# Children's height and weight in rural and urban populations in low-income and middle-income countries: a systematic analysis of population-representative data

**DOI:** 10.1016/S2214-109X(13)70109-8

**Published:** 2013-11

**Authors:** Christopher J Paciorek, Gretchen A Stevens, Mariel M Finucane, Majid Ezzati, on behalf of the Nutrition Impact Model Study Group (Child Growth)

**Affiliations:** a Department of Statistics, University of California, Berkeley, CA, USA; b Department of Health Statistics and Information Systems, World Health Organization, Geneva, Switzerland; c Gladstone Institutes, University of California, San Francisco, CA, USA; d MRC-PHE Centre for Environment and Health, Department of Epidemiology and Biostatistics, Imperial College London, London, UK

## Abstract

**Background:**

Urban living affects children's nutrition and growth, which are determinants of their survival, cognitive development, and lifelong health. Little is known about urban–rural differences in children's height and weight, and how these differences have changed over time. We aimed to investigate trends in children's height and weight in rural and urban settings in low-income and middle-income countries, and to assess changes in the urban–rural differentials in height and weight over time.

**Methods:**

We used comprehensive population-based data and a Bayesian hierarchical mixture model to estimate trends in children's height-for-age and weight-for-age *Z* scores by rural and urban place of residence, and changes in urban–rural differentials in height and weight *Z* scores, for 141 low-income and middle-income countries between 1985 and 2011. We also estimated the contribution of changes in rural and urban height and weight, and that of urbanisation, to the regional trends in these outcomes.

**Findings:**

Urban children are taller and heavier than their rural counterparts in almost all low-income and middle-income countries. The urban–rural differential is largest in Andean and central Latin America (eg, Peru, Honduras, Bolivia, and Guatemala); in some African countries such as Niger, Burundi, and Burkina Faso; and in Vietnam and China. It is smallest in southern and tropical Latin America (eg, Chile and Brazil). Urban children in China, Chile, and Jamaica are the tallest in low-income and middle-income countries, and children in rural areas of Burundi, Guatemala, and Niger the shortest, with the tallest and shortest more than 10 cm apart at age 5 years. The heaviest children live in cities in Georgia, Chile, and China, and the most underweight in rural areas of Timor-Leste, India, Niger, and Bangladesh. Between 1985 and 2011, the urban advantage in height fell in southern and tropical Latin America and south Asia, but changed little or not at all in most other regions. The urban–rural weight differential also decreased in southern and tropical Latin America, but increased in east and southeast Asia and worldwide, because weight gain of urban children outpaced that of rural children.

**Interpretation:**

Further improvement of child nutrition will require improved access to a stable and affordable food supply and health care for both rural and urban children, and closing of the the urban–rural gap in nutritional status.

**Funding:**

Bill & Melinda Gates Foundation, Grand Challenges Canada, UK Medical Research Council.

## Introduction

Restricted growth in young children is a risk factor for mortality from infectious diseases and for poor physical and cognitive development throughout life.^1^, ^2^, ^3^, ^4^ Analyses of trends in children's growth (and those of other global health indicators) have been done at the regional or at most the national level.^5^ National measurement is suitable for monitoring progress towards the Millennium Development Goals (MDGs), which are based on countries as reporting units.^5^ However, more than half of the world's population now lives in cities, compared with about a third in 1985. The urban share of population in developing regions ranges from 37% in sub-Saharan Africa to nearly 80% in Latin America and the Caribbean.^6^ In such an urbanising world, stratified rural and urban information is needed to formulate policies and programmes that improve nutrition, since the optimum (or even feasible) strategies are different for rural and urban populations, which differ in their local infrastructure, living environment, quantity and types of food available, source of income, and health-care access and quality.

Urban–rural differentials in children's anthropometric status have been reported in some countries at one point in time.^7^, ^8^, ^9^ Beyond these examples, little is known about the differences between the nutritional status of urban and rural children, and especially about how these differences have changed over time. We aimed to analyse trends in children's height and weight, which are anthropometric measures of their nutritional status and determinants of their survival, cognitive development, and lifelong health, by rural and urban place of residence, for 141 low-income and middle-income countries. We also investigated the urban–rural differentials in height and weight, and how they have changed between 1985 and 2011. Finally, we estimated the contribution of changes in children's heights and weights in rural and urban settings versus the contribution of urbanisation to the regional trends in these measures.

## Methods

### Data sources

We refer to the 141 countries in our analysis as the low-income and middle-income countries. They cover all geographical regions apart from Europe and the high-income countries in Asia-Pacific, Australasia, and North America. The countries were divided into seven geographical regions (appendix pp 1–2 lists the countries in each region). Some, such as Saudi Arabia and Kuwait, have a high per-person gross domestic product (GDP), but have demographic and epidemiological characteristics that are more similar to other low-income and middle-income countries in their region.

Because nutrition has a strong effect on children's growth, their nutritional status can be assessed from their height (or length) and weight relative to their age. Height-for-age and weight-for-age *Z* scores are measures of how a child's height and weight compare with a well-nourished reference population of the same age, by calculation of each child's relative position in the reference population distribution. We used the 2006 WHO growth standards as the reference population.^10^ One *Z* score for height is about 3·2 cm at age 2 years and 4·7 cm at age 5 years; one *Z* score for weight is about 1·4 kg at age 2 years and 2·6 kg at age 5 years.

We collated a comprehensive database of population-representative data for height and weight of children younger than 5 years. The database and the full data search process have been described in a previous report.^5^ Briefly, our data sources included: health examination, nutrition, and household surveys with anonymised individual records available through national and international agencies and through survey databases; summary statistics, including means and prevalences below specific thresholds, from the WHO database of child anthropometric indicators; and summary statistics not in the WHO database, extracted from reports by national and international agencies. For this work, we extended the database to include information by rural and urban place of residence. The list of data sources by country is provided in the appendix (pp 3–41). Rural and urban classifications were based on those of national statistical offices, which are generally used both for survey design and for dividing populations into rural or urban by the UN Population Division.

When individual-level data were available, we calculated *Z* scores on the basis of the WHO standards. Some summary statistical data, especially from older sources, were available to us only as summary statistics in relation to the 1977 US Centers for Disease Control and Prevention National Center for Health Statistics (NCHS) reference. To use a consistent reference population, we converted these summary statistics from the NCHS reference to the WHO standards, as described previously.^5^

### Statistical analysis

We used a Bayesian hierarchical mixture model to estimate distributions of height-for-age and weight-for-age *Z* scores by rural and urban place of residence for all 141 countries for every year from 1985 to 2011. The statistical model is an extension of the model used and described in detail previously.^5^ Here we extended the model to make separate estimates for rural and urban areas; the statistical details about the extension for subgroup analysis are reported elsewhere.^11^, ^12^ In the hierarchical model, estimates for each country-year were informed by data from that country-year itself, if available, and by data from other years in the same country and in other countries, especially those in the same region with data for similar time periods. The hierarchical model shares information to a greater extent when data are non-existent or weakly informative (eg, because they have a small sample size), and to a lesser extent in data-rich countries and regions.

We modelled trends over time as a linear trend plus a smooth non-linear trend. The estimates were also informed by time-varying covariates that help to predict height-for-age and weight-for-age *Z* scores, including maternal education, national income (natural logarithm of per-person GDP in inflation-adjusted international dollars), proportion of the population living in urban areas, and an aggregate metric of access to basic health care.^5^ Finally, the model accounted for the fact that data that did not cover the entire country and data that did not cover the complete 0–59 month age range might have had more variation relative to the true values than nationally representative data and data that covered the full range of ages.

The Bayesian mixture model uses a mixture (weighted average) of multiple normal densities to estimate the full distributions of height-for-age and weight-for-age *Z* scores, which can themselves be skewed. Here we used two five-component mixtures, one for rural children and another for urban children. This approach uses all data sources—those that separate rural and urban children, those in which data for rural and urban children are reported together, and those in which only one group was measured—to make separate estimates by rural or urban place of residence. The differences in distributions of height-for-age and weight-for-age *Z* scores between rural and urban children were allowed to vary by country and year. In years and countries for which separate data by place of residence were missing, the estimated difference was informed by other sources, especially those from the same region with data from similar time periods.

The uncertainties of our estimates incorporated sampling error in each data source; non-sampling error of national data (eg, because of issues with sample design and measurement); additional error associated with subnational data; uncertainty due to conversion from NCHS reference to WHO standards; and uncertainty due to estimates made by country and year for which data were missing altogether, when only summary statistics (rather than individual-level data) were available, or when data were not available separately by place of residence.

We fitted the Bayesian model using a Markov chain Monte Carlo algorithm and, after thinning the chains, obtained 2500 samples from the parameters' posterior, in turn used to obtain 2500 posterior samples of the population distributions of height-for-age and weight-for-age *Z* scores for rural and urban children for each country-year. With each of the 2500 sampled distributions we calculated the mean height-for-age and weight-for-age *Z* scores and the prevalences of stunting and underweight for each country-year, separately for rural and urban children. All reported credible intervals represent percentiles 2·5–97·5 of these 2500 draws.

We calculated distributions for regions, and for all 141 low-income and middle-income countries combined, as population-weighted averages of those of the constituent countries. We report the posterior probability (PP) that an estimated increase or decrease in the urban–rural differential represents a truly increasing or decreasing trend. The PP would be 0·50 when an increase is statistically indistinguishable from a decrease, and a larger PP suggests increased certainty. We also report the PP that an estimated difference between urban and rural children represents a true difference in the same direction as the point estimate.

Finally, we additively decomposed the changes in mean height and weight at the country, regional, and global level into those associated with change in rural children, those associated with change in urban children, and those associated with urbanisation (ie, an increase in the proportion of a country's population living in cities). The first two components are those represented by changes in mean height-for-age and weight-for-age *Z* scores in the rural and urban children, weighted by the proportion of children who lived in urban and rural areas, respectively, in 1985. The final component, the role of urbanisation, is the change in the proportion of children who were urban multiplied by the difference in *Z* scores between urban and rural children in 2011.

### Role of the funding sources

The sponsors of the study had no role in study design, data collection, data analysis, data interpretation, or writing of the report. CJP and GAS had access to all data sources. The corresponding author had final responsibility for the decision to submit for publication.

## Results

Our database included 673 population-based sources covering the period between 1985 and 2011, with a total of 8·6 million children whose heights and weights were measured. This database provided an average of 4·8 data sources per country, ranging between 1·2 per country in the small region of Oceania to 9·3 per country in south Asia. 568 (84%) data sources were national, with the other 16% being representative of at least the first administrative level (eg, province, canton, or state). 126 countries, which together had more than 99% of the total population of the 141 countries included in the study, had at least one data source; of these, 113 had at least two. At least one data source was available for every country in south Asia and sub-Saharan Africa, the regions with the highest prevalences of undernutrition.

**Figure 1:**
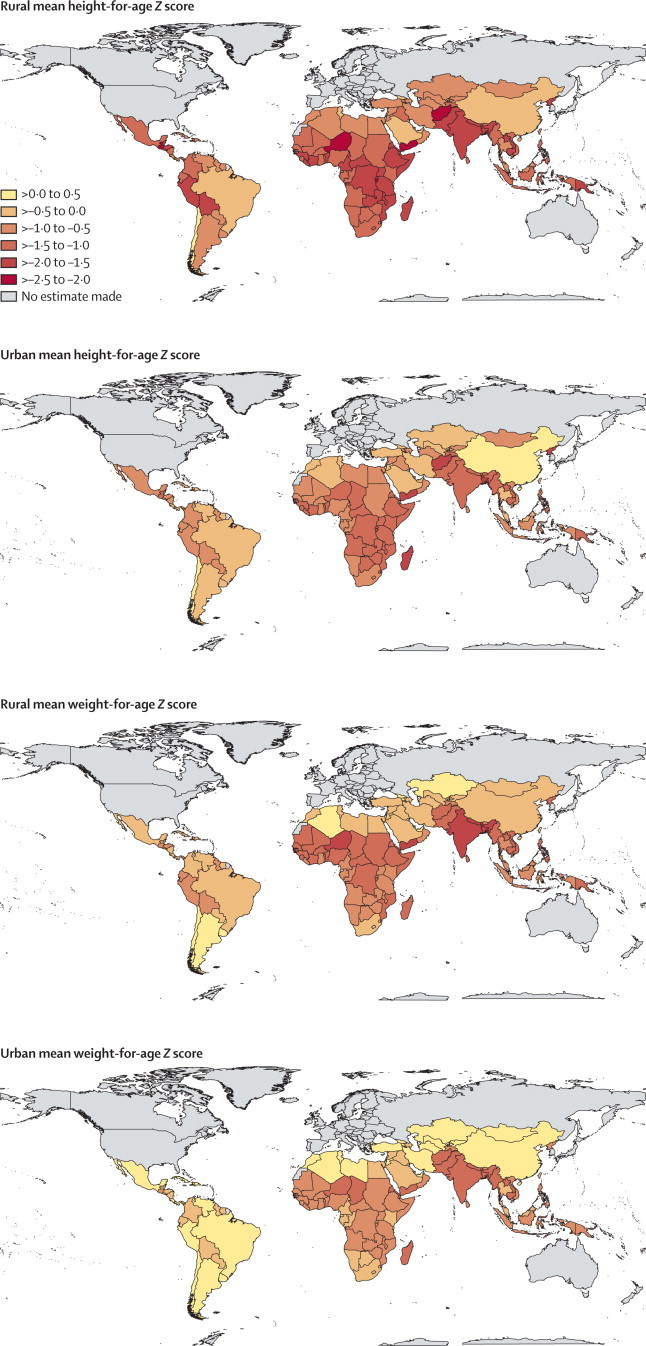
Children's mean height-for-age and weight-for-age *Z* scores in rural and urban areas of low-income and middle-income countries in 2011 A population with a mean height-for-age or weight-for-age *Z* score of zero would have the same average height or weight as a well-nourished WHO reference population.^10^ Negative numbers indicate countries that are on average shorter or more underweight than the reference population, whereas positive numbers indicate those that are taller or heavier than the reference population.

In 2011, children younger than 5 years living in cities in China, followed by Chile and Jamaica, were the tallest in low-income and middle-income countries, with mean height-for-age *Z* scores ranging from 0·07 to 0·22, and those in rural areas of Burundi, Guatemala, Niger, Yemen, and Afghanistan the shortest, all with mean height-for-age *Z* scores lower than −2·0 (figure 1). Mean height-for-age *Z* scores in the tallest and the shortest groups were more than 2·2 apart (equivalent to more than 10 cm at age 5 years). Whereas Chinese urban children were the tallest in low-income and middle-income countries, Chinese rural children's height was significantly lower than the WHO growth standard (−0·38; 95% credible interval −0·54 to −0·24), putting China as the ninth tallest out of 141 countries with respect to rural height.

In 2011, the heaviest children lived in cities in Georgia, followed by Chile and China, with mean weight-for-age *Z* scores ranging from 0·35 to 0·43, and the most underweight in rural areas of Timor-Leste, India, Niger, and Bangladesh, where weight-for-age *Z* scores were lower than −1·6. Mean weight-for-age *Z* scores in the heaviest and most underweight groups were more than 2·0 apart (equivalent to more than 5 kg at age 5 years). Urban children in 41 countries had mean weight-for-age *Z* scores greater than zero, whereas urban children in only four countries had height-for-age *Z* scores greater than zero. Even in rural areas, children in 14 countries had mean weight-for-age *Z* scores greater than the WHO growth standard, but in only three countries was the mean height-for-age *Z* score greater than the standard.

**Figure 2:**
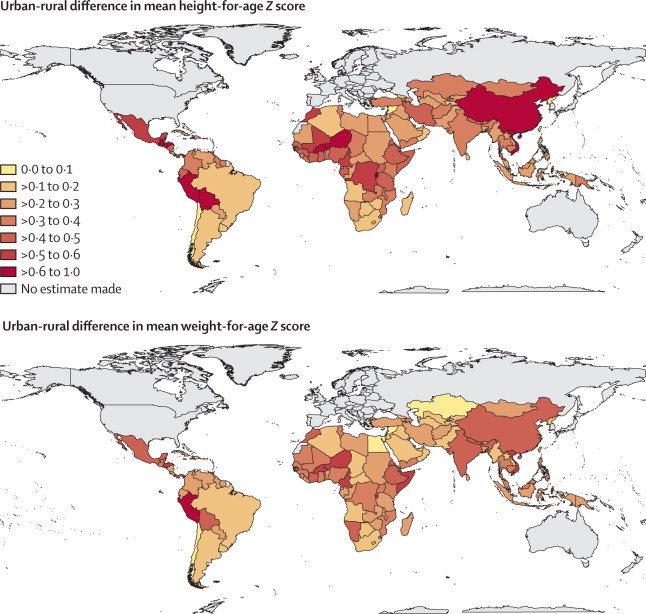
Urban advantage in children's height-for-age and weight-for-age *Z* scores, by country, in 2011 Larger numbers indicate greater differences in height or weight between urban and rural children. For height, one *Z* score is equivalent to about 3·2 cm of height at age 2 years and 4·7 cm at age 5 years. For weight, one *Z* score is equivalent to about 1·4 kg of weight at age 2 years and 2·6 kg at age 5 years. The appendix (pp 42–43) reports urban–rural differences in the prevalences of stunting and underweight.

In 2011, children who lived in cities were taller and heavier than their rural counterparts in almost all low-income and middle-income countries (figure 2). The urban advantage in children's height and weight was largest in Andean and central Latin America and the Caribbean (eg, Peru, Bolivia, Honduras, and Guatemala); in some African countries such as Niger, Burundi, and Burkina Faso; and in Vietnam and China. The urban–rural height-for-age *Z* score differential in these countries ranged between 0·6 and 0·9; the range for the urban–rural weight-for-age *Z* scores gap was between 0·4 and 0·7. Correspondingly, stunting and underweight were less prevalent among urban children than among rural children in these countries, by up to 30 percentage points (appendix pp 42–43). At the other extreme, rural and urban children's heights and weights differed very little in a handful of largely urban countries including Chile, the occupied Palestinian territory, Syria, and Jamaica.

**Figure 3:**
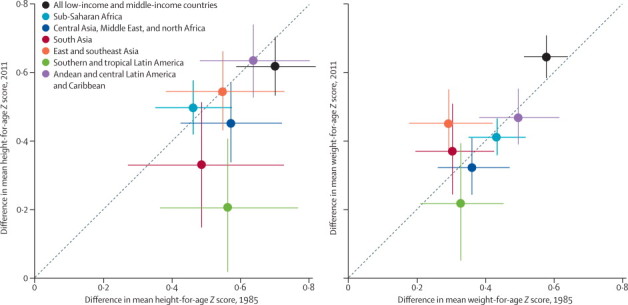
Change in urban advantage in children's height-for-age and weight-for-age *Z* scores between 1985 and 2011, by region A point above the diagonal line means a larger improvement in urban areas than in rural areas; a point below the diagonal line indicates the opposite. The vertical and horizontal error bars show the 95% credible intervals. The figure excludes Oceania because its large credible intervals would reduce the visibility of data from other regions. The urban–rural differential in height-for-age *Z* score in Oceania was 0·56 (0·11–1·05) in 1985 and 0·57 (0·24–0·91) in 2011. The urban–rural differential in weight-for-age *Z* score in Oceania was 0·38 (0·12–0·68) in 1985 and 0·37 (0·15–0·61) in 2011. The global advantage is larger than all region-specific advantages because regions such as southern and tropical Latin America where children are tallest and heaviest are also those that are more heavily urbanised, whereas regions with the shortest and most underweight child populations are those that are more rural, such as south Asia (ie, the global results encompass both within-region and between-region differences).

**Figure 4:**
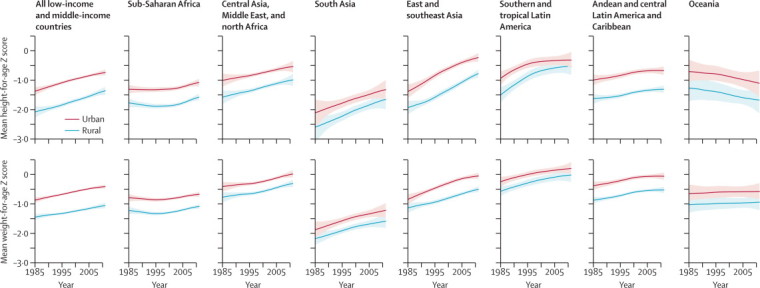
Trends in rural and urban mean height-for-age and weight-for-age *Z* score, by region The solid line represents the posterior mean and the shaded area the 95% uncertainty interval. The appendix reports trends in prevalences of stunting and underweight by region (pp 44–45), and trends in rural and urban mean height-for-age and weight-for-age *Z* scores and prevalences of stunting and underweight by country (pp 46–188).

Between 1985 and 2011, the urban advantage in height fell most substantially in southern and tropical Latin America (PP=0·99), followed by south Asia (PP=0·83) and a region that consisted of central Asia, the Middle East, and North Africa (PP=0·86; figure 3). In these regions, successive cohorts of rural children grew taller at a faster rate than those living in cities (figure 4). In other regions, rural and urban children's height improved at about the same rate, with virtually no change in the urban advantage over these 26 years.

At the country level, the urban–rural gap in height decreased in 100 countries and increased in 41 countries; in 34 of the countries in which the gap decreased, the PP that the change was a true reduction was at least 80%. The equalising trends were largest in Syria, Bangladesh, Thailand, Brazil, and Indonesia, where the urban–rural gap fell by a height-for-age *Z* score of 0·3 or more (appendix pp 46–188). The urban–rural height gap grew the most in Vietnam, Mali, Cameroon, and Niger. In Vietnam, urban children's growth seems to have outpaced that of rural children, who nonetheless grew taller during the period of analysis. In the three African countries, the heights of rural children worsened or stagnated, while urban children had modestly favourable trends.

Southern and tropical Latin America also saw a reduction in the urban–rural weight differential (figure 3; PP=0·83). By contrast, the urban–rural gap in weight increased in east and southeast Asia (PP=0·98), where urban children's weight gain outpaced that of rural children (figure 4; the increasing trend might have reversed in the last few years of the analysis). This faster pace of of weight gain by urban children was most pronounced in Vietnam, China, and Cambodia. The urban–rural weight gap might also have increased in south Asia (PP=0·74). Altogether, our best estimate was that the urban–rural gap in children's weight narrowed in 113 countries (19 of which had a PP of a truly shrinking gap of at least 80%) and widened in 28 countries.

**Figure 5:**
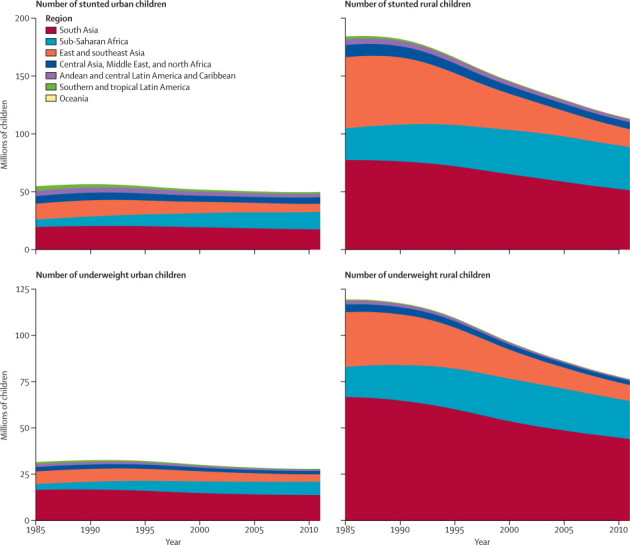
Number of stunted and underweight children, by region and rural or urban place of residence, 1985–2011 Stunted is defined as a height-for-age *Z* score lower than −2. Underweight is defined as weight-for-age *Z* score lower than −2.

The number of stunted children in in these 141 low-income and middle-income countries fell from 239 million (95% credible interval 220–258 million) in 1985 to 163 million (149–179 million) in 2011, while the number of underweight children fell from 151 million (137–165 million) to 105 million (92–119 million; figure 5). Almost the entire decline occurred in rural areas, with the number of undernourished children who live in cities changing little, because of the opposing effects of improvements in nutrition and urban population growth. In sub-Saharan Africa, where the urban population is growing faster than in any other region and where little gain was seen in children's height and weight (figure 4), the number of undernourished children in cities more than doubled over these 26 years. At the other extreme, the number of stunted and underweight children in rural east and southeast Asia fell by about three-quarters because of the combined trends of urbanisation and large improvements in height and weight. The improvements in this region were so substantial that even the number of undernourished children in cities fell by more than 40%, despite the increasing urban population. In 2011, 31% (95% credible interval 29–32) of the stunted children in low-income and middle-income countries and 27% (25–28) of those underweight lived in cities, compared with 23% (22–24) and 21% (20–22) in 1985. The proportion was roughly 80% in southern and tropical Latin America, the most urbanised region included in the study.

We have previously shown that, between 1985 and 2011, children's height and weight increased substantially in all regions apart from sub-Saharan Africa and Oceania.^5^ Of the increase in children's height in these 141 countries, 64% (95% credible interval 58–69) can be attributed to increases in rural children, 27% (23–31) to increases in urban children, and 9% (7–13) to urbanisation. Of the worldwide increase in children's weight, 55% (47–61) can be attributed to rural weight gain, 30% (27–34) to urban weight gain, and 15% (11–20%) to urbanisation. The relative contributions of these three components varied by region (table). In Asia, gains in rural height and weight drove overall improvements, because these countries were largely rural and because rural children steadily grew taller and heavier. By contrast, in Latin America and the Caribbean, urban gains were the main driver of the overall improvements.

**Table:** Contributions of rural improvement, urban improvement, and urbanisation to change in mean height-for-age and weight-for-age *Z* scores, by region

	**Height-for-age Z score**			**Weight-for-age *Z* score**		
	Rural component	Urban component	Urbanisation component	Rural component	Urban component	Urbanisation component
All 141 low-income and middle-income countries	0·64 (0·58 to 0·69)	0·27 (0·23 to 0·31)	0·09 (0·07 to 0·13)	0·55 (0·47 to 0·61)	0·30 (0·27 to 0·34)	0·15 (0·11 to 0·20)
Sub-Saharan Africa	0·55 (0·09 to 0·69)	0·22 (0·08 to 0·35)	0·23 (0·13 to 0·67)	0·52 (0·00 to 0·72)	0·15 (−0·10 to 0·28)	0·33 (0·14 to 1·03)
Central Asia, Middle East, and north Africa	0·51 (0·40 to 0·63)	0·42 (0·28 to 0·51)	0·07 (0·04 to 0·13)	0·48 (0·40 to 0·57)	0·46 (0·38 to 0·53)	0·06 (0·03 to 0·09)
South Asia	0·77 (0·69 to 0·86)	0·20 (0·11 to 0·27)	0·03 (0·01 to 0·06)	0·71 (0·61 to 0·78)	0·25 (0·18 to 0·32)	0·04 (0·02 to 0·09)
East and southeast Asia	0·68 (0·63 to 0·72)	0·23 (0·20 to 0·26)	0·09 (0·07 to 0·12)	0·61 (0·54 to 0·67)	0·26 (0·23 to 0·30)	0·12 (0·09 to 0·17)
Southern and tropical Latin America	0·37 (0·27 to 0·50)	0·59 (0·46 to 0·67)	0·04 (0·00 to 0·10)	0·31 (0·20 to 0·42)	0·63 (0·51 to 0·70)	0·07 (0·01 to 0·15)
Andean and central Latin America and Caribbean	0·32 (0·18 to 0·46)	0·49 (0·27 to 0·62)	0·20 (0·12 to 0·36)	0·35 (0·25 to 0·47)	0·51 (0·35 to 0·61)	0·14 (0·10 to 0·22)
Oceania	0·77 (0·09 to 1·52)	0·22 (−0·54 to 0·89)	0·01 (−0·09 to 0·12)	0·91 −0·72 to 2·16)	0·09 (−1·21 to 1·67)	−0·01 (−0·24 to 0·26)

Data are means of the posterior draws (95% credible intervals). The three components sum to 1·00. Any component can be negative or greater than 1·00 when one component causes an increase in height or weight in a particular region and another does the opposite.

## Discussion

Undernutrition restricts children's physical growth, which in turn increases the risk of dying from infectious diseases and adversely affects their cognitive development, school performance, and health in adulthood.^1^, ^2^, ^3^, ^4^, ^13^ In addition to being included in the MDGs, renewed attention to child nutrition and growth is evident through the UN Secretary General's *Every Woman Every Child* initiative and the *Scaling Up Nutrition* initiative. Priority setting and assessment of these activities have been based on national-level measurement and reporting, with little attention paid to how nutritional status differs between rural and urban populations. Our results show that, with the notable exception of southern and tropical Latin America, a substantial urban advantage in child nutrition has persisted or even grown in many countries and regions (panel). This urban advantage persists both where children's growth indicators have improved (eg, some countries in east and southeast Asia), and where they have stagnated (eg, parts of sub-Saharan Africa).

Panel **Research in context**

**Systematic review**

We searched PubMed for articles published between Jan 1, 1990, and Oct 11, 2012 using combinations of the keywords “underweight” or “stunting”, and “urban” or “rural”. The search was restricted to publications about low-income and middle-income countries or global analyses. Previous studies reported urban–rural differentials in children's anthropometric status at one point in time on the basis of data from household surveys, and in some cases analysed associations with household characteristics.^7^, ^8^, ^9^ Beyond these examples, little has been reported about the differences between urban and rural children with respect to nutritional status, and especially about how these differences have changed over time.

**Interpretation**

We analysed trends in children's height and weight *Z* scores, which are anthropometric measures of their nutritional status, by rural and urban place of residence for 141 low-income and middle-income countries. Urban children are taller and heavier than their rural counterparts in almost all low-income and middle-income countries, but the differential, and its trends over time, varied substantially across countries and regions. The urban–rural differential in children's height and weight is largest in Andean and central Latin America (eg, Peru, Honduras, Bolivia, and Guatemala); in some African countries such as Niger, Burundi, and Burkina Faso; and in Vietnam and China. It is smallest in southern and tropical Latin America (eg, Chile and Brazil). Between 1985 and 2011, the urban advantage in height fell in southern and tropical Latin America and south Asia, but changed little or not at all in most other regions. The urban–rural weight differential also decreased in southern and tropical Latin America, but increased in east and southeast Asia and worldwide, because weight gain of urban children outpaced that of rural children. The persistent urban–rural gap should be addressed in continuing and future efforts to improve child nutrition worldwide.

The main strength of our study is its novel scope. This is the first comprehensive global analysis of urban–rural differentials for a major health risk, and the first analysis of how the urban–rural gap has changed in the past quarter century. We accessed many data sources from different countries and regions. Our statistical model incorporated non-linear trends and took into account that some studies were nationally representative whereas others were subnational and hence could have had larger variability. We have also systematically estimated and reported the uncertainty of our estimates. Finally, we quantified the contributions of changes in rural and urban height and weight and of urbanisation to the global and regional trends in these outcomes.

The main limitation of our study is that, despite our wide-ranging data search and access (we obtained as many or more data sources, especially national data sources, per country as most previous analyses of risk factors by ourselves and others^11^, ^14^, ^15^, ^16^, ^17^), some countries still had little data available, leading to larger uncertainty in their estimates. This issue was especially pertinent in the small region of Oceania, where nutritional surveys are done less frequently than in other regions. Additionally, some older sources had used the NCHS reference to calculate height-for-age and weight-for-age *Z* scores, and hence had to be converted to the newer WHO standards for comparability, which also led to larger uncertainty.

Although our data sources largely used the rural and urban classification of national statistical offices, identical classification does not imply identical characteristics.^18^, ^19^ Cities and rural areas in different countries vary in their demographic characteristics (eg, population size or density), economic activity, administrative structures, services (eg, health care and education), infrastructure (roads, sanitation, water, energy, and communication), and environment (green space and air pollution).^19^ Some of these differences exist because countries themselves differ in every way—there is no typical city because there is no typical country. Others are a consequence of historical, administrative, or even political choices in the classification system.^18^, ^19^ To the extent that our findings are used for further investigation into the causes and determinants of urban–rural differentials in cross-country analyses, these differences should be taken into account by use of data for city characteristics; to the extent that they are used for measurement of inequality and for targeting efforts to reduce these inequalities, such heterogeneity matters less. This limitation also reveals the challenges of analysing subnational health patterns and inequalities in relation to socioeconomic (eg, education and wealth or income) and geographical factors.^20^ Such subnational analyses are needed, but will inevitably face challenges in terms of how to define population subgroups that are consistent over time and comparable across countries.

We could not separate to what extent urbanisation is caused by rural to urban migration versus differences in rural and urban fertility and mortality; the two mechanisms might have different effects on nutritional trends in rural and urban areas, and on the urban–rural gap. The most important driver of urban population growth at present is natural population growth in existing urban areas, accounting for about 60% of urban population growth.^21^ Longitudinal studies or linked data sources could be used to investigate whether migrants converge to the nutritional (and health) status of the existing urban population or whether they retain those of their communities of origin.^22^ Because urbanisation is a social process that generally improves standard of living and health status,^18^, ^23^, ^24^ convergence would seem to be the more likely possibility. Once again, this distinction, which is relevant for understanding the causes of the trends seen, matters less with respect to the measurement of subgroup inequality and the targeting of interventions. Finally, we did not measure social inequalities within urban or rural populations, which are large in some countries;^4^ these inequalities should be the subject of future analyses.

Children's growth is restricted when they do not receive sufficient nutritious foods or lose nutrients during sickness, both of which situations arise from a range of adverse proximal and distal social, environmental, nutritional, and health-care determinants.^4^, ^25^, ^26^ At the micro (household) level, urban advantage in children's nutrition seems to be partly associated with better economic status and higher maternal education in urban than in rural households.^27^ Community-level determinants are also important—these include sanitation infrastructure; the quantity, types, and price of available food; and physical and financial access to health care (and the quality of health care available).^28^

Cross-sectional household-level analyses do not reveal to what extent social and health policies have affected changes in urban–rural differentials. Even in countries with relatively rich data, identification of the independent contributions of specific determinants to the reported trends has been difficult,^29^, ^30^ perhaps because these determinants interact and are dynamic. Previous research nonetheless suggests that economic growth tends to improve child nutrition at the aggregate national level, but attention to income equity, policies and programmes that improve food production and food security, and investment in primary care are also necessary.^31^, ^32^, ^33^, ^34^, ^35^

Our findings for rural and urban differentials and trends emphasise the need to examine the role of equity in income, food security, and services not only in aggregate, but also with respect to how they affect nutrition in urban and rural populations separately. Specifically, the closing urban–rural gap in southern and tropical Latin America and the widening gap in some countries in east and southeast Asia both occurred as economies grew and the nutritional statuses of both rural and urban children showed substantial improvements; what differed between the two regions was the catching up versus falling behind of rural children relative to urban children during these advances. In Brazil, falling inequality in child nutrition has been traced to more equitable access to education, health care, clean water and sanitation, and reproductive health services (all despite large income inequality).^29^ Some of these services might have been less equally distributed in the fast-growing economies in east and southeast Asia where the urban–rural gap increased (at least until recently, as evidenced by the health-care reforms currently in progress in China).^36^

At the other extreme, the urban–rural gap also changed little or increased in most countries in sub-Saharan Africa, where there had been periods of macroeconomic stagnation or contraction. Previous research has implicated structural adjustment and trade policy reforms that accompanied macroeconomic shocks, and led to reduced spending on agriculture, food subsidies, and health care, as reasons for worsening nutritional status nationally, probably with larger adverse effects on poor rural children than on those living in cities.^37^, ^38^, ^39^, ^40^ In these countries, geographical inequality in economic adversity seems to have caused the persistent or increasing urban–rural height and weight differentials, mirroring the inequality in economic gain seen in parts of Asia.

Finally, we saw that in some Asian countries urban children outpaced their rural counterparts in weight gain, even though their heights grew at a similar pace. Urban children in 41 countries are on average heavier than the WHO growth standard; in eight countries they had mean weight-for-age *Z* scores of 0·25 or larger, whereas none of the 141 countries had achieved an equivalent mean height-for-age *Z* score. These results signify the need for strategies to curb the rising child obesity in an urbanising world, while reducing undernutrition.^4^

Closing the urban–rural gap seems to demand both policies that improve the economic status and food security of rural households, and more equitable access to interventions and services such as clean water and sanitation, complementary feeding with local foods, and case management of diarrhoea and other infectious diseases.^41^, ^42^ At the same time, an increasing share of undernourished children lives in cities. Despite having an advantage over their rural counterparts, these children are susceptible to environmental and economic shocks that affect food security and prices. Policies and programmes are needed to address poverty, environment, and nutrition in urban settings to parallel those that focus on rural areas.

## References

[1] Black RE, Allen LH, Bhutta ZA, for the Maternal and Child Undernutrition Study Group (2008). Maternal and child undernutrition: global and regional exposures and health consequences. Lancet.

[2] Grantham-McGregor S, Cheung YB, Cueto S, Glewwe P, Richter L, Strupp B (2007). Developmental potential in the first 5 years for children in developing countries. Lancet.

[3] Olofin I, McDonald CM, Ezzati M (2013). Associations of suboptimal growth with all-cause and cause-specific mortality in children under five years: a pooled analysis of ten prospective studies. PLoS One.

[4] Black RE, Victora CG, Walker SP (2013). Maternal and child undernutrition and overweight in low-income and middle-income countries. Lancet.

[5] Stevens GA, Finucane MM, Paciorek CJ, on behalf of Nutrition Impact Model Study Group (Child Growth) (2012). Trends in mild, moderate, and severe stunting and underweight, and progress towards MDG 1 in 141 developing countries: a systematic analysis of population representative data. Lancet.

[6] UN (2011). World urbanization prospects, the 2011 revision.

[7] Krumdiek CL (1971). The rural-to-urban malnutrition gradient: a key factor in the pathogenesis of urban slums. JAMA.

[8] Fox K, Heaton TB (2012). Child nutritional status by rural/urban residence: a cross-national analysis. J Rural Health.

[9] Smith LC, Ruel MT, Ndiaye A (2005). Why is child malnutrition lower in urban than in rural areas? Evidence from 36 developing countries. World Dev.

[10] WHO (2006). WHO child growth standards length/height-for-age, weight-for-age, weight-for-length, weight-for-height and body mass index-for-age: methods and development.

[11] Stevens GA, Finucane MM, De-Regil LM, on behalf of Nutrition Impact Model Study Group (Anaemia) (2013). Global, regional, and national trends in haemoglobin concentration and prevalence of total and severe anaemia in children and pregnant and non-pregnant women for 1995–2011: a systematic analysis of population-representative data. Lancet Glob Health.

[12] Finucane MM, Paciorek CJ, Stevens GA, Ezzati M Semiparametric Bayesian density estimation with disparate data sources: a meta-analysis of global childhood undernutrition. http://arxiv.org/pdf/1301.5390v1.pdf.

[13] McDonald CM, Olofin I, Flaxman S (2013). The effect of multiple anthropometric deficits on child mortality: meta-analysis of individual data in 10 prospective studies from developing countries. Am J Clin Nutr.

[14] Danaei G, Finucane MM, Lin JK, on behalf of the Global Burden of Metabolic Risk Factors of Chronic Diseases Collaborating Group (Blood Pressure) (2011). National, regional, and global trends in systolic blood pressure since 1980: systematic analysis of health examination surveys and epidemiological studies with 786 country-years and 5·4 million participants. Lancet.

[15] Danaei G, Finucane MM, Lu Y, on behalf of the Global Burden of Metabolic Risk Factors of Chronic Diseases Collaborating Group (Blood Glucose) (2011). National, regional, and global trends in fasting plasma glucose and diabetes prevalence since 1980: systematic analysis of health examination surveys and epidemiological studies with 370 country-years and 2·7 million participants. Lancet.

[16] Farzadfar F, Finucane MM, Danaei G, on behalf of the Global Burden of Metabolic Risk Factors of Chronic Diseases Collaborating Group (Cholesterol) (2011). National, regional, and global trends in serum total cholesterol since 1980: systematic analysis of health examination surveys and epidemiological studies with 321 country-years and 3·0 million participants. Lancet.

[17] Finucane MM, Stevens GA, Cowan MJ, on behalf of the Global Burden of Metabolic Risk Factor of Chronic Diseases Collaborating Group (Body Mass Index) (2011). National, regional, and global trends in body mass index since 1980: systematic analysis of health examination surveys and epidemiological studies with 960 country-years and 9·1 million participants. Lancet.

[18] Leon DA (2008). Cities, urbanization and health. Int J Epidemiol.

[19] Utzinger J, Keiser J (2006). Urbanization and tropical health—then and now. Ann Trop Med Parasitol.

[20] Di Cesare M, Khang YH, Asaria P, on behalf of *The Lancet* NCD Action Group (2013). Inequalities in non-communicable diseases and effective responses. Lancet.

[21] Montgomery MR, Stren R, Cohen B, Reed HE (2004). Cities transformed: demographic change and its implications in the developing world.

[22] Miranda JJ, Gilman RH, Garcia HH, Smeeth L (2009). The effect on cardiovascular risk factors of migration from rural to urban areas in Peru: PERU MIGRANT Study. BMC Cardiovasc Disord.

[23] Hall P (1998). Cities in civilization—culture, innovation, and urban order.

[24] Keyfitz N, Committee on Resources and Man of the Division of Earth Sciences, National Academy of Sciences–National Research Council (1969). United States and world populations. Resources and man—a study and recommendations.

[25] UNICEF (1990). Strategy for improved nutrition of children and women in developing countries.

[26] Scrimshaw NS, SanGiovanni JP (1997). Synergism of nutrition, infection, and immunity: an overview. Am J Clin Nutr.

[27] Garrett JL, Ruel MT (1999). Are determinants of rural and urban food security and nutritional status different? Some insights from Mozambique. World Dev.

[28] Rutherford ME, Mulholland K, Hill PC (2010). How access to health care relates to under-five mortality in sub-Saharan Africa: systematic review. Trop Med Int Health.

[29] Monteiro CA, Benicio MH, Conde WL (2010). Narrowing socioeconomic inequality in child stunting: the Brazilian experience, 1974–2007. Bull World Health Organ.

[30] von Braun J, Ruel M, Gulati A (2008). Accelerating progress toward reducing child malnutrition in India.

[31] Smith LC, Haddad L (2002). How potent is economic growth in reducing undernutrition? What are the pathways of impact? New cross-country evidence. Econ Dev Cult Change.

[32] Ravallion M (1990). Income effects on undernutrition. Econ Dev Cult Change.

[33] Anand S, Ravallion M (1993). Human-development in poor countries: on the role of private incomes and public services. J Econ Perspect.

[34] Subramanyam MA, Kawachi I, Berkman LF, Subramanian SV (2011). Is economic growth associated with reduction in child undernutrition in India?. PLoS Med.

[35] Haddad L, Alderman H, Appleton S, Song L, Yohannes Y (2003). Reducing child malnutrition: how far does income growth take us?. World Bank Econ Rev.

[36] Yip WC-M, Hsiao WC, Chen W, Hu S, Ma J, Maynard A (2012). Early appraisal of China's huge and complex health-care reforms. Lancet.

[37] Pongou R, Salomon JA, Ezzati M (2006). Health impacts of macroeconomic crises and policies: determinants of variation in childhood malnutrition trends in Cameroon. Int J Epidemiol.

[38] Cooper Weil D, Alicbusan A, Wilson J, Reich M, Bradley D (1990). The Impact of development policies on health: a review of the literature.

[39] Sundberg S (2009). Agriculture, poverty and growth in Africa: linkages and policy challenges. CAB Rev: Perspect Agric Vet Sci Nutr Nat Resour.

[40] World Bank (2008). World development report 2008: agriculture for development.

[41] Sanchez PA, Swaminathan MS (2005). Hunger in Africa: the link between unhealthy people and unhealthy soils. Lancet.

[42] Bhutta ZA, Das JK, Rizvi A (2013). Evidence-based interventions for improvement of maternal and child nutrition: what can be done and at what cost?. Lancet.

