# Supplementary material for: Children's height and weight in rural and urban populations in low-income and middle-income countries: a systematic analysis of population-representative data
Source: Lancet Glob Health. 2013 Nov;1(5):e300–9. doi: 10.1016/S2214-109X(13)70109-8 (PMC4547325; doi:10.1016/S2214-109X(13)70109-8)

THE LANCET Global Health

Supplementary appendix

This appendix formed part of the original submission and has been peer reviewed. We post it as supplied by the authors.

Supplement to: Paciorek CJ, Stevens GA, Finucane MM, Majid Ezzati, on behalf of the Nutrition Impact Model Study Group (Child Growth). Children's height and weight in rural and urban populations in low-income and middle-income countries: a systematic analysis of population-representative data. *Lancet Glob Health* 2013; **1:** e300–09.

Appendix Table 1. List of countries and territories in each region.

**Region**	**Countries**
East and Southeast Asia	Cambodia, China, China (Hong Kong SAR), China (Macao SAR), Democratic People's Republic of Korea, Indonesia, Lao People's Democratic Republic, Malaysia, Maldives, Myanmar, Philippines, Sri Lanka, Taiwan, Thailand, Timor-Leste, Viet Nam
Oceania	Fiji, Kiribati, Marshall Islands, Micronesia (Federated States of), Papua New Guinea, Samoa, Solomon Islands, Tonga, Vanuatu
South Asia	Afghanistan, Bangladesh, Bhutan, India, Nepal, Pakistan
Central Asia, Middle East, and North Africa	Algeria, Armenia, Azerbaijan, Bahrain, Egypt, Georgia, Iran (Islamic Republic of), Iraq, Jordan, Kazakhstan, Kuwait, Kyrgyzstan, Lebanon, Libyan Arab Jamahiriya, Mongolia, Morocco, Occupied Palestinian Territory, Oman, Qatar, Saudi Arabia, Syrian Arab Republic, Tajikistan, Tunisia, Turkey, Turkmenistan, United Arab Emirates, Uzbekistan, Yemen
Sub-Saharan Africa	Angola, Benin, Botswana, Burkina Faso, Burundi, Cameroon, Cape Verde, Central African Republic, Chad, Comoros, Congo, Côte d'Ivoire, Democratic Republic of the Congo, Djibouti, Equatorial Guinea, Eritrea, Ethiopia, Gabon, Gambia, Ghana, Guinea, Guinea-Bissau, Kenya, Lesotho, Liberia, Madagascar, Malawi, Mali, Mauritania, Mauritius, Mozambique, Namibia, Niger, Nigeria, Rwanda, Senegal, Seychelles, Sierra Leone, Somalia, South Africa, Sudan, Swaziland, São Tomé and Príncipe, Togo, Uganda, United Republic of Tanzania, Zambia, Zimbabwe
Andean and Central Latin America and Caribbean	Antigua and Barbuda, Bahamas, Barbados, Belize, Bermuda, Bolivia, Colombia, Costa Rica, Cuba, Dominica, Dominican Republic, Ecuador, El Salvador, Grenada, Guatemala, Guyana, Haiti, Honduras, Jamaica, Mexico, Nicaragua, Panama, Peru, Puerto Rico, Saint Lucia, Saint Vincent and the Grenadines, Suriname, Trinidad and Tobago, Venezuela (Bolivarian Republic of)
Southern and Tropical Latin America	Argentina, Brazil, Chile, Paraguay, Uruguay

Appendix Table 2. Data sources used in the analysis and their characteristics.

Country	Year	Age range	Administrative level	Sample size	Indicators	Notes*	Survey (when individual level data available) or source (when summary statistics available)
Afghanistan	1995	0-59.99	Regional	1674	HAZ, WAZ	3,5	Kakar F and Kakar SR. Indicators of child morbidity and mortality in three Afghan provinces. The Research and Advisory Council of Afghanistan and UNICEF Aghanistan Program Office. Kabul, Afghanistan, 1996.
Afghanistan	1997	6-59.99	National	4846	HAZ, WAZ	3,5	Afghanistan 1997 multiple indicator baseline (MICS). Report to UNICEF. Acapulco: Centro de Investigacion de Enfermedades Tropicales (CIET), 1998 (and additional analysis).
Afghanistan	2000	0-59.99	Regional (rural)	638	HAZ, WAZ	3,5	Afghanistan MICS2 Steering Committee. 2000 Afghanistan multiple indicator cluster survey (MICS2) Vol.1: Situation analysis of children and women in the east of Afghanistan. ICONS, SHAIP Islamabad, Deutsche Gesellschaft für Technische Zusammenarbeit GmbH, UNICEF Afghanistan, 2001.
Afghanistan	2000	6-59.99	First admin level	5499	HAZ, WAZ	3,5	Lukmanji Z and Hamidzai A. Nutrition survey of children under five years: Province of Badakshan, Afghanistan, January 2000. FOCUS Humanitarian Assistance Europe Foundation and Department of Health, Province of Badakshan, Afghanistan. FOCUS Europa Foundation, May 2000.
Afghanistan	2002	0-59.99	First admin level	676	HAZ, WAZ	3,5	Tohill UR. GOAL Samangan Province, nutrition survey, May - June 2002. GOAL report No. 2. GOAL Afghanistan, 2002.
Afghanistan	2002	0-59.99	First admin level	717	HAZ, WAZ	3,5	Tohill UR. GOAL Jawzjan Province, nutrition survey, April 2002. GOAL report No. 1. GOAL Afghanistan, 2002.
Afghanistan	2002	0-59.99	First admin level	545	HAZ, WAZ	3,5	Woodruff BA, Reynolds M, Tchibindat F, Ahimana C. Nutrition and health survey, Badghis Province, Afghanistan, February - March 2002. UNICEF and U.S. Centers for Disease Control and Prevention (CDC), 2002.
Afghanistan	2004	6-59.99	National	946	HAZ, WAZ	2	Ministry of Public Health (Afghanistan), UNICEF, CDC, National Institute for Research on Food and Nutrition (Italy), Tufts University. Summary report of the national nutrition survey, 2004. Kabul, Islamic Republic of Afghanistan: Ministry of Public Health and UNICEF, 2005 (and additional analysis).
Algeria	1992	0-59.99	National	4207	HAZ, WAZ	1	PAPCHILD Algeria
Algeria	1995	0-59.99	National	3824	HAZ, WAZ	2	Ministère de la Santé et de la Population. Enquête nationale sur les objectifs de la mi-decennie, "MDG Algerie", 1995. Alger, Algerie, 1996 (and additional analysis).
Algeria	2000	0-59.99	National	4178	HAZ, WAZ	2	Minstère de la Santé et de la Population. Enquête nationale sur les objectives de la fin décennie santé mère et enfant EDG Algérie 2000 (MICS). Institut National de Santé Publique. République Algérienne Démocratique et Populaire, 2001 (and additional analysis).
Algeria	2002	0-59.99	National	4419	HAZ, WAZ	2	Ministère de la Santé, de la Population et de la Réforme Hospitalière et l'Office National des Statistiques. Enquête Algérienne sur la santé de la famille-2002: Rapport principal. Alger, Algérie: Agence Nationale de la Documentation en Santé, 2004 (and additional analysis).

Algeria	2005	0-59.99	National	13975	HAZ, WAZ	2	Ministère de la Santé de la Population et de la Réforme Hospitalière, Office National des Statistiques. Suivi de la situation des enfant et des femmes. Enquête nationale à indicateurs multiples: Rapport principal. MICS3. République Algérienne Démocratique et Populaire, Décembre 2008 (and additional analysis).
Angola	1996	6-59.99	National	3016	HAZ, WAZ	2	Inquerito de indicadores multiplos (MICS) 1996. Instituto Nacional de Estatistica - Gabinete de Monitorizaçao das Condiçoes de Vida da Populaçao. Luanda, Angola, 1999 (and additional analysis).
Angola	2001	0-59.99	National	5106	HAZ, WAZ	1	MICS
Angola	2007	6-59.99	National	10224	HAZ, WAZ	3	Ministerio da Saude. Relatorio do inquerito sobre a nutriçao em Angola 2007. Luanda, Republica de Angola: Ministerio da Saude, Direcçao nacional de Saude Publica, 2008.
Argentina	1985-1986	9-23.99	First admin level	430	HAZ, WAZ	3,5	Calvo EB, Islam J, Gnazzo N, Ibanez M, de Martinez CB, de Vacaliuc RS et al. [Encuesta nutricional en ninos de 2 anos de la provincia de Misiones. I. Indicadores antropometricos.] Archivos Argentinos de Pediatria 1987;85:247-269.
Argentina	1994	0-59.99	Regional (urban)	3967	HAZ, WAZ	2,5	Calvo EB. Encuesta antropometrica de niños menores de 6 años. En: Infancia y condiciones de vida. Encuesta especial para el diagnostico y la evaluacion de las metas sociales. Publicacion INDEC, Buenos Aires, 1995 (and additional analysis).
Argentina	1994	0-59.99	National	5296	HAZ, WAZ	3,5	Lejarraga H, Krupitzky S, Gimenez E, Diament N, Kelmansky A, Tibaldi F, et al. The organisation of a national survey for evaluating child psychomotor development in Argentina. Paediatric and Perinatal Epidemiology 1997;11:359-373 (and additional analysis).
Argentina	2004-2005	6-59.99	National	44731	HAZ, WAZ	2	Durán P, Mangialavori G, Biglieri A, Kogan L, Gilardon EA. Nutrition status in Argentinean children 6 to 72 months old. Results from the National Nutrition and Health Survey (ENNyS). Archivos Argentinos de Pediatria 2009;107:397-404 (and additional analysis).
Armenia	1998	0-59.99	National	3241	HAZ, WAZ	2	The health and nutritional status of children an women in Armenia. National Institute of Nutrition - Italy, 1998 (and additional analysis).
Armenia	2000	0-59.99	National	1508	HAZ, WAZ	1	DHS
Armenia	2005	0-59.99	National	1231	HAZ, WAZ	1	DHS
Armenia	2010	0-59.99	National	1305	HAZ, WAZ	1	DHS
Azerbaijan	1996	6-59.99	National	500	HAZ, WAZ	3,5	Branca F, Burkholder B, Hamel M, Parvanta I, Robertson A. Health and nutrition survey of internally displaced and resident population of Azerbaijan - April 1996. Baku, Azerbaijan, 1996 (and additional analysis).
Azerbaijan	2000	0-59.99	National	1716	HAZ, WAZ	1	MICS
Azerbaijan	2001	3-59.99	National	2426	HAZ, WAZ	3,5	Adventist Development and Relief Agency, Azerbaijan Ministry of Health, State Committee of Statistics, Mercy Corps [Baku, Azerbaijan], DRH/CDC, USAID, UNFPA, UNHCR. Reproductive health survey Azerbaijan, 2001: Final report. Serbanescu F, Morris L, Rahimova S, Stupp P, eds. Atlanta, GA: US Department of Health and Human Services, CDC, 2003 (and additional analysis).

Azerbaijan	2006	0-59.99	National	1925	HAZ, WAZ	1	DHS
Bahrain	1989	0-59.99	National	2033	HAZ, WAZ	3,5	Ministry of Health. Bahrain child health survey 1989. Manama, Bahrain, 1992.
Bahrain	1995	0-59.99	National	673	HAZ, WAZ	3,5	Naseeb T and Farid SM. Bahrain family health survey 1995: principal report. Manama, Bahrain: Ministry of Health, 2000.
Bangladesh	1985-1986	6-59.99	National	2675	HAZ, WAZ	3,5	Government of the Peoples's Republic of Bangladesh. Report of the child nutrition status module, Bangladesh household expenditure survey 1985-86. Bangladesh bureau of statistics. Dhaka, Bangladesh, 1987 (and additional analysis).
Bangladesh	1989-1990	6-59.99	National	1914	HAZ, WAZ	3,5	Government of the People's Republic of Bangladesh. Report of the child nutrition status survey 1989-90. Bangladesh bureau of statistics. Dhaka, Bangladesh, 1991 (and additional analysis).
Bangladesh	1991	6-59.99	Regional (rural)	32493	HAZ, WAZ	2	Helen Keller International (HKI) Bangladesh. Nutritional Surveillance Project 1991: data on rural national (using the WHO Child Growth Standards). Unpublished estimates. Dhaka, Bangladesh: HKI and Institute of Public Health Nutrition, 2008.
Bangladesh	1992	6-59.99	Regional (rural)	36997	HAZ, WAZ	2	Helen Keller International (HKI) Bangladesh. Nutritional Surveillance Project 1992: data on rural national (using the WHO Child Growth Standards). Unpublished estimates. Dhaka, Bangladesh: HKI and Institute of Public Health Nutrition, 2008.
Bangladesh	1993	6-59.99	Regional (rural)	42826	HAZ, WAZ	2	Helen Keller International (HKI) Bangladesh. Nutritional Surveillance Project 1993: data on rural national (using the WHO Child Growth Standards). Unpublished estimates. Dhaka, Bangladesh: HKI and Institute of Public Health Nutrition, 2008.
Bangladesh	1994	6-59.99	Regional (rural)	63753	HAZ, WAZ	2	Helen Keller International (HKI) Bangladesh. Nutritional Surveillance Project 1994: data on rural national (using the WHO Child Growth Standards). Unpublished estimates. Dhaka, Bangladesh: HKI and Institute of Public Health Nutrition, 2008.
Bangladesh	1995	6-59.99	Regional (rural)	87051	HAZ, WAZ	2	Helen Keller International (HKI) Bangladesh. Nutritional Surveillance Project 1995: data on rural national (using the WHO Child Growth Standards). Unpublished estimates. Dhaka, Bangladesh: HKI and Institute of Public Health Nutrition, 2008.
Bangladesh	1996-1997	0-59.99	National	4672	HAZ, WAZ	1	DHS
Bangladesh	1996	6-59.99	Regional (rural)	81067	HAZ, WAZ	2	Helen Keller International (HKI) Bangladesh. Nutritional Surveillance Project 1996: data on rural national (using the WHO Child Growth Standards). Unpublished estimates. Dhaka, Bangladesh: HKI and Institute of Public Health Nutrition, 2008.
Bangladesh	1998	6-59.99	National (rural)	49496	HAZ, WAZ	2	Helen Keller International (HKI) Bangladesh. Nutritional Surveillance Project 1998: data on rural national (using the WHO Child Growth Standards). Unpublished estimates. Dhaka, Bangladesh: HKI and Institute of Public Health Nutrition, 2008.

Bangladesh	1999	6-59.99	National (rural)	49374	HAZ, WAZ	2	Helen Keller International (HKI) Bangladesh. Nutritional Surveillance Project 1999: data on rural national (using the WHO Child Growth Standards). Unpublished estimates. Dhaka, Bangladesh: HKI and Institute of Public Health Nutrition, 2008.
Bangladesh	1999-2000	0-59.99	National	5302	HAZ, WAZ	1	DHS
Bangladesh	2000	0-59.99	National (rural)	63632	HAZ, WAZ	2	Helen Keller International (HKI) Bangladesh. Nutritional Surveillance Project 2000: data on rural national (using the WHO Child Growth Standards). Unpublished estimates. Dhaka, Bangladesh: HKI and Institute of Public Health Nutrition, 2008.
Bangladesh	2001	0-59.99	National (rural)	63445	HAZ, WAZ	2	Helen Keller International (HKI) Bangladesh. Nutritional Surveillance Project 2001: data on rural national (using the WHO Child Growth Standards). Unpublished estimates. Dhaka, Bangladesh: HKI and Institute of Public Health Nutrition, 2008.
Bangladesh	2002	0-59.99	National (rural)	53314	HAZ, WAZ	2	Helen Keller International (HKI) Bangladesh. Nutritional Surveillance Project 2002: data on rural national (using the WHO Child Growth Standards). Unpublished estimates. Dhaka, Bangladesh: HKI and Institute of Public Health Nutrition, 2008.
Bangladesh	2003	0-59.99	National (rural)	86998	HAZ, WAZ	2	Helen Keller International (HKI) Bangladesh. Nutritional Surveillance Project 2003: data on rural national (using the WHO Child Growth Standards). Unpublished estimates. Dhaka, Bangladesh: HKI and Institute of Public Health Nutrition, 2008.
Bangladesh	2004	0-59.99	National (rural)	73690	HAZ, WAZ	2	Helen Keller International (HKI) Bangladesh. Nutritional Surveillance Project 2004: data on rural national (using the WHO Child Growth Standards). Unpublished estimates. Dhaka, Bangladesh: HKI and Institute of Public Health Nutrition, 2008.
Bangladesh	2004	0-59.99	National	5895	HAZ, WAZ	1	DHS
Bangladesh	2005	0-59.99	National (rural)	74758	HAZ, WAZ	2	Helen Keller International (HKI) and Institute of Public Health Nutrition. Nutritional Surveillance Project 2005: Rural data (using the WHO Child Growth Standards). Unpublished estimates. Dhakar, Bangladesh: HKI and Institute of Public Health Nutrition, 2007.
Bangladesh	2006	0-59.99	National (rural)	24302	HAZ, WAZ	2	Helen Keller International (HKI) Bangladesh. Nutritional Surveillance Project 2006: data on rural national data (using the WHO Child Growth Standards). Unpublished estimates. Dhaka, Bangladesh: HKI and Institute of Public Health Nutrition, 2008 (and additional analysis).
Bangladesh	2007	0-59.99	National	5263	HAZ, WAZ	1	DHS
Belize	1992	0-59.99	National	8516	WAZ	3,5	Ministry of Health. Assessment of the food, nutrition and health situation of Belize. INCAP Publication DC1/002. Kingston: Institute of Nutrition of Central America and Panama, 1992 (and additional analysis).
Belize	2006	0-59.99	National	686	HAZ, WAZ	1	MICS
Benin	1996	0-35.99	National	2521	HAZ, WAZ	1	DHS
Benin	2001	0-59.99	National	4374	HAZ, WAZ	1	DHS
Benin	2006	0-59.99	National	12149	HAZ, WAZ	1	DHS

Bhutan	1999	6-59.99	National	2996	HAZ, WAZ	2	Ministry of Health and Education. National anthropometric survey of under five children in Bhutan. Division of Health Services. Thimpu, Bhutan, 1999 (and additional analysis).
Bhutan	2008	6-59.99	National	2348	HAZ, WAZ	2	Ministry of Health, National Statistics Bureau and Centre for Research Initiative. National nutrition, infant & young child feeding survey 2008. Thimphu, Bhutan: Ministry of Health and UNICEF, 2009 (and additional analysis).
Bhutan	2010	0-59.99	National	6071	HAZ, WAZ	2	National Statistics Bureau (NSB). Bhutan multiple indicator cluster survey (BMIS) 2010. Thimphu, Bhutan: NSB, May 2011.
Bolivia	1989	3-35.99	National	2603	HAZ, WAZ	1	DHS
Bolivia	1990	0-59.99	First admin level	89490	WAZ	3,5	Ministerio de Prevision Social y Salud Publica. Santa Cruz: estado nutricional de la poblacion menor de cinco anos. Boletin de Sistema de Vigilancia Epidemiologica Nutricional 1991;5(3):1-3.
Bolivia	1993-1994	0-35.99	National	2867	HAZ, WAZ	1	DHS
Bolivia	1996	0-59.99	National	1302	HAZ, WAZ	3,5	Gutierrez Sardan M. Encuesta nacional de multiples indicadores 1996 (MICS). La Paz: Ministerio de Desarrollo Humano, enero 1997 (and additional analysis).
Bolivia	1998	0-59.99	National	6139	HAZ, WAZ	1	DHS
Bolivia	2003-2004	0-59.99	National	9091	HAZ, WAZ	1	DHS
Bolivia	2008	0-59.99	National	7672	HAZ, WAZ	1	DHS
Botswana	1996	0-59.99	National	NR	HAZ, WAZ	3,5,7	The 1996 Botswana family health survey III. Gaborone: Central Statistics Office, 1999 (and additional analysis).
Botswana	2000	0-59.99	National	2880	HAZ, WAZ	2	Charumbira Multiple indicator survey (MIS) 2000. Full report. Gaborone, Botswana, 2001 (and additional analysis).
Botswana	2007-2008	0-59.99	National	2623	HAZ, WAZ	2	2007 Botswana family health survey IV report. Gaborone, Botswana: CSO, 2009 (and additional analysis by UNICEF).
Brazil	1986	0-59.99	National	1167	HAZ, WAZ	1	DHS
Brazil	1987	0-35.99	First admin level	4349	HAZ, WAZ	2,5	Saude e nutricao das criancas nordestinas; pesquisas estaduais 1987-92. UNICEF/Universidade Federal de Pelotas. Brasilia: UNICEF, 1995.
Brazil	1989	0-59.99	First admin level	1093	HAZ, WAZ	2,5	Saude e nutricao das criancas nordestinas; pesquisas estaduais 1987-92. UNICEF/Universidade Federal de Pelotas. Brasilia: UNICEF, 1995.
Brazil	1989	0-59.99	First admin level	1038	HAZ, WAZ	2,5	Saude e nutricao das criancas nordestinas; pesquisas estaduais 1987-92. UNICEF/Universidade Federal de Pelotas. Brasilia: UNICEF, 1995.
Brazil	1989	0-59.99	National	7487	HAZ, WAZ	1	PNSN 1989
Brazil	1990	0-35.99	First admin level	2770	HAZ, WAZ	2,5	Saude e nutricao das criancas nordestinas; pesquisas estaduais 1987-92. UNICEF/Universidade Federal de Pelotas. Brasilia: UNICEF, 1995.
Brazil	1991	0-59.99	First admin level	1262	HAZ, WAZ	2,5	Saude e nutricao das criancas nordestinas; pesquisas estaduais 1987-92. UNICEF/Universidade Federal de Pelotas. Brasilia: UNICEF, 1995.
Brazil	1991	0-59.99	First admin level	1244	HAZ, WAZ	2,5	Saude e nutricao das criancas nordestinas; pesquisas estaduais 1987-92. UNICEF/Universidade Federal de Pelotas. Brasilia: UNICEF, 1995.
Brazil	1991	0-59.99	First admin level	916	HAZ, WAZ	2,5	Saude e nutricao das criancas nordestinas; pesquisas estaduais 1987-92. UNICEF/Universidade Federal de Pelotas. Brasilia: UNICEF, 1995.

Brazil	1991	0-59.99	First admin level	1026	HAZ, WAZ	2,5	Saude e nutricao das criancas nordestinas; pesquisas estaduais 1987-92. UNICEF/Universidade Federal de Pelotas. Brasilia: UNICEF, 1995.
Brazil	1996	0-59.99	National	4045	HAZ, WAZ	1	DHS
Brazil	2002-2003	0-59.99	National	17107	WAZ	2	Laboratório de Avaliaçao Nutricional de Populaçoes, Universidade de Sao Paulo (LANPOP-USP). Pesquisa de orçamentos familiares (POF - 2002-03). Rio de Janeiro, Brazil, 2007 (http://www.ibge.gov.br/home/estatistica/populacao/condicaodevida/pof/2003medidas/default.shtm).
Brazil	2006-2007	0-59.99	National	4415	HAZ, WAZ	2	Centro Brasileiro de Análise e Planejamento [CEBRAP]. Pesquisa nacional de demografia e saúde da criança e da mulher - PNDS 2006. Relatório da pesquisa. Sao Paulo: CEBRAP, 2008 (http://bvsms.saude.gov.br/bvs/pnds/index.php and additional analysis).
Burkina Faso	1987	12-47.99	Regional	606	WAZ	3,5	Ouedraogo NA. Analyse de la situation nutritionnelle des populations du Burkina Faso. Conference Internationale sur la Nutrition. Rome: FAO/WHO, 1992.
Burkina Faso	1992-1993	0-59.99	National	4358	HAZ, WAZ	1	DHS
Burkina Faso	1998-1999	0-59.99	National	4515	HAZ, WAZ	1	DHS
Burkina Faso	2003	0-59.99	National	8007	HAZ, WAZ	1	DHS
Burkina Faso	2006	0-59.99	National	4680	HAZ, WAZ	1	MICS
Burkina Faso	2009	0-59.99	National	15318	HAZ, WAZ	3	Ministry of Health. Enquête nutritionnelle nationale 2009. Ouagadougou, Burkina Faso: Direction de la Nutrition, 2009 (and additional analysis).
Burkina Faso	2011	0-59.99	National	6994	HAZ, WAZ	2	Institut National de la Statistique et de la Demographie, Measure DHS. Burkina Faso Enquete Demographique et de la Sante et a Indicateurs Multiples 2010: Rapport preliminaire. Ouagadougou, Burkina Faso and Calverton, Maryland, USA: Institut National de la Statistique et de la Demographie, and ICF Macro, 2011.
Burundi	1987	3-35.99	National	1896	HAZ, WAZ	1	DHS
Burundi	2000	6-59.99	National	2586	HAZ, WAZ	1	MICS
Burundi	2005	0-59.99	National	7065	HAZ, WAZ	3,5	Rapport de l'enquête nationale de nutrition de la population, 2005. Bujumbura, Burundi, October 2006 (and additional analysis).
Burundi	2010	0-59.99	National	3590	HAZ, WAZ	3	Institut de Statistiques et d'Etudes Economiques du Burundi, Institud National de Sante Publique, Measure DHS. Burundi Enquete Demographique et de la Sante 2010: Rapport preliminaire. Calverton, Maryland, USA: Institut de Statistiques et d'Etudes Economiques du Burundi, Institud National de Sante Publique, and ICF Macro, 2011.
Cambodia	1996	0-59.99	National	5773	HAZ, WAZ	2	Ministry of Planning. Socio economic survey of Cambodia 1996: Volume 1, summary results. National Institute of Statistics. Phnom Penh, Cambodia, 1997 (and additional analysis).

Cambodia	2000	0-59.99	National (rural)	19556	HAZ, WAZ	2,5	Helen Keller International, Cambodia. Initial findings from the 2000 Cambodia national micronutrient survey: supporting doc. to the micronutrient workshop on February 20, 2001, Phnom Penh, Kingdom of Cambodia. Helen Keller Worldwide, Phnom Penh, Cambodia, 2001 (and additional analysis).
Cambodia	2000	0-59.99	National	3467	HAZ, WAZ	1	DHS
Cambodia	2005-2006	0-59.99	National	3566	HAZ, WAZ	1	DHS
Cambodia	2008	0-59.99	National	7018	HAZ, WAZ	2	National Institute of Statistics, Ministry of Planning and UNICEF Cambodia. Cambodia anthropometrics survey 2008. Phnom Penh, Cambodia: National Institute of Statistics, Ministry of Planning and UNICEF Cambodia, 2009 (and additional analysis).
Cambodia	2010	0-59.99	National	3675	HAZ, WAZ	1	DHS
Cameroon	1990	0-59.99	First admin level	2010	HAZ, WAZ	2	Mendoza Aldana J, Piechulek H. Situation nutritionnelle des enfants de 0 à 59 mois en zone urbaine et rurale du Cameroun. Bulletin of the World Health Organization 1992;70:725-732.
Cameroon	1991	0-59.99	National	2615	HAZ, WAZ	1	DHS
Cameroon	1998	0-35.99	National	1789	HAZ, WAZ	1	DHS
Cameroon	2004	0-59.99	National	3189	HAZ, WAZ	1	DHS
Cameroon	2006	0-59.99	National	6117	HAZ, WAZ	2	Institut National de la Statistique et UNICEF. Cameroun: Suivi de la situation des enfants et des femmes. Enquête par grappee à indicateurs multiples 2006. Rapport principal. Yaoundé, Cameroun: Institut National de la Statistique et UNICEF, 2008 (and additional analysis).
Cameroon	2011	0-59.99	National	5860	HAZ, WAZ	3	Institut National de la Statistique, Ministere de l'Economie, Ministere de la Sante Publique, Measure DHS. Cameroun Enquete Demographique et de la Sante et a Indicateurs Multiples 2011: Rapport preliminaire. Yaounde, Cameroun and Calverton, Maryland, USA: Institut National de la Statistique, Ministere de l'Economie, Ministere de la Sante Publique, and ICF Macro, 2011.
Cape Verde	1994	0-59.99	National	1610	HAZ, WAZ	3,5	Ferreira Medina JB, Skard T, Sobhy S, America Ungaretti M. A saude das crianças menores de cinco anos em Cabo Verde. Ministério de Saude e Promoçao Social e UNICEF. Cabo Verde, 1996 (and additional analysis).
Central African Republic	1994-1995	0-35.99	National	2327	HAZ, WAZ	1	DHS
Central African Republic	1995	0-59.99	National	2225	HAZ, WAZ	3,5	Ministère de la Santé Publique et de la Population. Etat nutritionnel de la population. Rapport préliminaire de l'enquête de nutrition mai-juillet 1995. Bangui, République Centrafricaine, 1995 (and additional analysis).
Central African Republic	2000	0-59.99	National	12683	HAZ, WAZ	1	MICS
Central African Republic	2006	0-59.99	National	8562	HAZ, WAZ	1	MICS
Chad	1996-1997	0-59.99	National	5607	HAZ, WAZ	1	DHS
Chad	2000	0-59.99	National	5184	HAZ, WAZ	1	MICS
Chad	2004	0-59.99	National	4329	HAZ, WAZ	1	DHS

Chile	2001	0-59.99	National	1002454	HAZ, WAZ	3,4,5	Ministerio de Salud, Departamento de Estadisticas e Informacion. National health service system. Santiago, Chile, 2002 (and additional analysis).
Chile	2002	0-59.99	National	1022552	HAZ, WAZ	3,4,5	Ministerio de Salud, Departamento de Estadisticas e Informacion. National health service system. Santiago, Chile, 2003 (and additional analysis).
Chile	2007	0-47.99	National	604485	HAZ, WAZ	3,4	Atalah E. Ministerio de Salud. National Health Service System. Santiago, Chile, December 2007 (www.minsal.cl).
Chile	2008	0-47.99	National	665636	HAZ, WAZ	3,4	Atalah E. Ministerio de Salud. National Health Service System. Santiago, Chile, December 2008 (www.minsal.cl).
China	1987	0-59.99	Regional	76130	HAZ, WAZ	3,5	Institute of Nutrition and Food Hygiene. The third national growth and development survey of children in China, 1987 (and additional analysis).
China	1989	0-59.99	Regional	5744	HAZ, WAZ	1	CHNS 1989
China	1990	0-59.99	Regional	4332	HAZ, WAZ	2	Chen Chunming, He Wu, Wang Yuying. Nutritional status of children aged 0-5 years old in China (1990) - National surveillance system in 7 provinces. Beijing, China: Chinese Center for Disease Control and Prevention, 2010.
China	1991	2-59.99	Regional	964	HAZ, WAZ	1	CHNS 1991
China	1992	0-59.99	National	5535	HAZ, WAZ	2	Ge Keyou. The dietary and nutritional status of Chinese population (1992 national nutrition survey). Beijing: Institute of Nutrition and Food Hygiene, 1995 (and additional analysis).
China	1992	0-59.99	National (rural)	140994	HAZ, WAZ	2,5	Nutritional status of children - 1992 Child Survey. State Statistic Bureau. Presented at 7th Asian Congress of Nutrition. Beijing, China, 1995.
China	1992	0-59.99	National (urban)	38711	HAZ, WAZ	2,5	Nutritional status of children - 1992 Child Survey. State Statistic Bureau. Presented at 7th Asian Congress of Nutrition. Beijing, China, 1995.
China	1993	1-59.99	Regional	611	HAZ, WAZ	1	CHNS 1993
China	1995	0-59.99	Regional	2832	HAZ, WAZ	2	Chen Chunming, He Wu, Wang Yuying. Nutritional status of children aged 0-5 years old in China (1995) - National surveillance system in 7 provinces. Beijing, China: Chinese Center for Disease Control and Prevention, 2010.
China	1997	0-59.99	Regional	409	HAZ, WAZ	1	CHNS 1997
China	1998	0-59.99	National	13838	HAZ, WAZ	2	Chen Chunming, He Wu, Wang Yuying. Nutritional status of children aged 0-5 years old in China (1998) - National (40 nutrition surveillance sites from 26 provinces). Beijing, China: Chinese Center for Disease Control and Prevention, 2010.
China	1999	0-35.99	First admin level	1497	WAZ	3,5	Dang S, Yan H, Yamamoto S, Wang X, Zeng L. Poor nutritional status of younger Tibetan children living at high altitudes. European Journal of Clinical Nutrition 2004;58:938-46.
China	2000	2-59.99	Regional	373	HAZ, WAZ	1	CHNS 2000
China	2000	0-59.99	National	16460	HAZ, WAZ	2	Chen Chunming, He Wu, Wang Yuying. Nutritional status of children aged 0-5 years old in China (2000) - National (40 nutrition surveillance sites from 26 provinces). Beijing, China: Chinese Center for Disease Control and Prevention, 2010.
China	2002	0-59.99	National	16564	HAZ, WAZ	2	Yang X, Wang Z, He Y, Yu W, Hu Y, Zhai F. [Trends and prevalence of malnutrition among Chinese children under five years old.] Acta Nutrimenta Sinica 2005;25:185-88 (and additional analysis).
China	2004	1-59.99	Regional	345	HAZ, WAZ	1	CHNS 2004

China	2005	0-59.99	National	15987	HAZ, WAZ	2	Chen Chunming, He Wu, Wang Yuying. Nutritional status of children aged 0-5 years old in China (2005) - National (40 nutrition surveillance sites from 26 provinces). Beijing, China: Chinese Center for Disease Control and Prevention, 2010.
China	2006	0-59.99	Regional	336	HAZ, WAZ	1	CHNS 2006
China	2008	0-59.99	National (rural)	10726	HAZ, WAZ	2	Chen Chunming, He Wu, Wang Yuying. Nutritional status of children aged 0-5 years old in China (2008) - National (26 nutrition surveillance sites from rural areas). Beijing, China: Chinese Center for Disease Control and Prevention, 2010.
China	2009	0-59.99	National (rural)	10635	HAZ, WAZ	2	Chen Chunming et al. Nutrition and rapid economic development - 2010 research report on nutrition policy in China. Beijing, China: Chinese Center for Disease Control and Prevention, 2010.
China	2010	0-59.99	National	15399	HAZ, WAZ	2	Nutritional status of children aged 0-5 years old in China (2010) - National (38 nutrition surveillance sites from 25 provinces). Beijing, China: Chinese Center for Disease Control and Prevention, 2012 (and additional analysis).
China, Hong Kong SAR	1993	0-59.99	National	9654	HAZ, WAZ	2,5	Leung SSF. Growth standards for Hong Kong: a territory wide survey in 1993. Hong Kong: The Chinese University of Hong Kong, 1995.
Colombia	1986	3-35.99	National	1321	HAZ, WAZ	1	DHS
Colombia	1989	0-59.99	National	1973	HAZ, WAZ	3,5	Mora JO, de Paredes B, de Navarro L, Rodriguez E. Consistent improvement in the nutritional status of Colombian children between 1965 and 1989. Bulletin of PAHO 1992;26:1-13 (and additional analysis).
Colombia	1995	0-59.99	National	4495	HAZ, WAZ	1	DHS
Colombia	2000	0-59.99	National	4170	HAZ, WAZ	1	DHS
Colombia	2004-2005	0-59.99	National	12370	HAZ, WAZ	1	DHS
Colombia	2009-2010	0-59.99	National	15907	HAZ, WAZ	1	DHS
Comoros	1991-1992	0-23.99	National	1954	HAZ, WAZ	3,5	Ministère de la Santé Publique et de la Population. Rapport sur l'état nutritionnel et les facteurs impliqués chez les enfants de moins de deux ans en République Fédérale Islamique des Comores 1991. Direction de la Santé Familiale. Comores, 1995 (and additional analysis).
Comoros	1996	0-35.99	National	949	HAZ, WAZ	1	DHS
Comoros	2000	0-59.99	National	3233	HAZ, WAZ	1	MICS
Congo	1987	0-59.99	National (rural)	2429	HAZ, WAZ	3,5	Cornu A, Delpeuch F, Simondon F, Tchibindat F, Faucon LD, Massamba JP et al. Enquête nationale sur l'état nutritionnel des enfants d'age prescolaire au Congo. Collection Etudes et Theses. Paris: ORSTOM, Institut Français de Recherche Scientifique pour le Développement en Coopération, 1990 (and additional analysis).
Congo	2005	0-59.99	National	3844	HAZ, WAZ	1	DHS
Costa Rica	2008-2009	0-59.99	National	351	HAZ, WAZ	3,8	Ministerio de Salud. Encuesta nacional de nutricion 2008-2009. San Jose, Costa Rica, 2011.
Cuba	2000	0-59.99	National	1571	HAZ, WAZ	3,5	Encuesta de agrupacion de indicadores multiples: Informe final, diciembre del 2000 (MICS2). La Habana, Cuba, 2000 (and additional analysis).
Côte d'Ivoire	1986	0-59.99	National	1947	HAZ, WAZ	3,5	Sahn DE. Malnutrition in Côte d'Ivoire, prevalence and determinants. Working paper No. 4. Washington D.C.: The World Bank, 1990 (and additional analysis).

Côte d'Ivoire	1994	0-35.99	National	3398	HAZ, WAZ	1	DHS
Côte d'Ivoire	1998-1999	0-59.99	National	1529	HAZ, WAZ	1	DHS
Côte d'Ivoire	2006	0-59.99	National	8241	HAZ, WAZ	1	MICS
Côte d'Ivoire	2007	6-59.99	National	854	HAZ, WAZ	2	Tschannen AB, Rohner F, Gohou V, Bosso E, Malan A. Evaluation des carences en vitamine A et fer en Côte d'Ivoire (Rapport final). Ministère de la Santé et de l'Hygiène Publique et Helen Keller Int., Abidjan, Côte d'Ivoire, 2009 (and additional analysis).
Dem. People's Republic of Korea	1997	0-59.99	Regional	2275	HAZ	2,4,5	Katona Apte J, Mokdad A. Malnutrition of children in the Democratic People's Republic of North Korea. Journal of Nutrition 1998;128:1315-1319.
Dem. People's Republic of Korea	1998	6-59.99	Regional	1263	HAZ, WAZ	3,5	Nutrition survey of the Democratic People's Republic of Korea. Report by the EU, UNICEF and WFP of a study undertaken in collaboration with the Government to DPRK (Internet, 7 January 1999 at http://www.wfp.org/OP/Countries/dprk/nutrion_survey.html).
Dem. People's Republic of Korea	2000	0-59.99	National	4175	HAZ, WAZ	3,5	Central Bureau of Statistics [DPRK]. Report of the second multiple indicator cluster survey 2000, DPRK (MICS). Pyongyang, Democratic People's Republic of Korea, 2000.
Dem. People's Republic of Korea	2002	0-59.99	National	5297	HAZ, WAZ	2	Central Bureau of Statistics (D.P.R. Korea). Nutrition assessment 2002 D.P.R. Korea. Pyongyang: Government of D.P.R. Korea, United Nations Children's Fund and World Food Programme, 2003 (and additional analysis).
Dem. People's Republic of Korea	2009	0-59.99	National	2172	HAZ, WAZ	2	Central Bureau of Statistics (CBS), Institute of Children's Nutrition, UNICEF. The Democratic People's Republic of Korea (DPR Korea) multiple indicator cluster survey 2009 (MICS4). Final Report. Pyongyang, DPR Korea: CBS and UNICEF, 2010.
Democratic Republic of the Congo	1995	0-59.99	National	4362	HAZ, WAZ	3,5	Ministère du Plan et Reconstruction Nationale. Enquête nationale sur la situation des enfants et des femmes au Zaire en 1995. Kinshasa, Zaire, 1996 (and additional analysis).
Democratic Republic of the Congo	2001	0-59.99	National	9279	HAZ, WAZ	1	MICS
Democratic Republic of the Congo	2007	0-59.99	National	3215	HAZ, WAZ	1	DHS
Democratic Republic of the Congo	2010	0-59.99	National	10568	HAZ, WAZ	2	Ministry of Planning National Institute of Statistics in Collaboration with the United Nations Children's Fund Institut National de la Statistique et Fonds des Nations Unies pour l'Enfance, Enquete par Grappes a Indicateurs Multiples en Republique Democratique du Congo (MICS-RDS 2010), Rapport Final, Mai 2011.
Djibouti	1989	0-59.99	National	3750	HAZ, WAZ	3,5	Ministere de la Sante Publique et des Affaires Sociales. Enquete couverture vaccinale malnutrition. Republique de Djibouti, Djibouti: Ministere de la Sante Publique et des Affaires Sociales,1990 (and additional analysis).

Djibouti	1996	0-59.99	National	NR	HAZ, WAZ	3,5,7	Enquête djiboutienne auprès des ménages indicateurs sociaux (EDAM-IS 1996). Ministère du Commerce et du Tourisme, Direction Nationale de la Statistique. Djibouti ville, République de Djibouti, 1997 (and additional analysis).
Djibouti	2002	0-59.99	National	2289	HAZ, WAZ	2	Department of Statistics and Demographic Studies, Ministry of Health and Pan Arab Project for Family Health. Enquête djiboutienne sur la santé de la famille (EDSF/PAPFAM) 2002, PAPFAM Rapport final. Djibouti, 2004 (and additional analysis).
Djibouti	2006	0-59.99	National	1732	HAZ, WAZ	1	MICS
Dominican Republic	1986	6-35.99	National	1926	HAZ, WAZ	1	DHS
Dominican Republic	1991	0-59.99	National	3167	HAZ, WAZ	1	DHS
Dominican Republic	1996	0-59.99	National	3719	HAZ, WAZ	1	DHS
Dominican Republic	2000	0-59.99	National	1862	HAZ, WAZ	1	MICS
Dominican Republic	2002	0-59.99	National	9245	HAZ, WAZ	1	DHS
Dominican Republic	2006	0-59.99	National	3782	HAZ, WAZ	3,5	Oficina Nacional de Estadistica (ONE). Encuesta nacional de hogares de proposito multiples (ENHOGAR 2006): Informe general. Santo Domingo, Republica Dominicana: ONE, 2008 (and additional analysis).
Dominican Republic	2007	0-59.99	National	9212	HAZ, WAZ	1	DHS
Ecuador	1986	0-59.99	National	7798	HAZ, WAZ	3,5	Freire W, Dirren H, Mora J, Arenales P, Granda E, Breilh J, et al. [Diagnostico de la situacion alimentaria, nutricional y de salud de la poblacion ecuatoriana menor de cinco años. Quito: Ministerio de Salud Publica y Consejo Nacional de Desarrollo, 1988 (and additional analysis).
Ecuador	1998	0-59.99	National	2998	HAZ, WAZ	3,5	Larrea C, Freire WB, Lutter C. Equidad desde el principio - situacion nutricional de los niños ecuatorianos. Encuesta de condiciones de vida, 1998. Organizacion Panamericana de la Salud y Ministerio de Salud Publica, Ecuador. Washington, D.C.: OPS, 2001 (and additional analysis).
Ecuador	2004	0-59.99	National	5134	HAZ, WAZ	2	Centro de Estudios de Poblacion y Desarrollo Social (CEPAR) et al. Encuesta demografía y de salud materna e infantil, ENDEMAIN 2004: Informe final. Quito, Ecuador: CEPAR, 2005 (and additional analysis).
Egypt	1988-1989	3-35.99	National	2022	HAZ, WAZ	1	DHS
Egypt	1991	0-59.99	National	3446	HAZ, WAZ	1	PAPCHILD Egypt
Egypt	1992-1993	0-59.99	National	7174	HAZ, WAZ	1	DHS
Egypt	1995-1996	0-59.99	National	10037	HAZ, WAZ	1	DHS
Egypt	1996	0-59.99	National	1629	HAZ, WAZ	3,5	El Tawila S. Child well-being in Egypt: results of Egypt's multiple indicator cluster survey (MICS). Cairo: Social Research Centre, American University, 1997.
Egypt	1997-1998	0-59.99	National	3328	HAZ, WAZ	3,5	Egypt demographic and health survey 1997. Demographic and Health Surveys. El-Zanaty and Associates, Cairo, Egypt, 1998 (and additional analysis).
Egypt	1998	0-59.99	National	3997	HAZ, WAZ	3,5	El-Zanaty et al. Egypt demographic and health survey 1998. Demographic and Health Surveys. Cairo, Egypt, 1999 (and additional analysis).

Egypt	2000	0-59.99	National	10210	HAZ	1	DHS
Egypt	2003	0-59.99	National	6019	HAZ, WAZ	1	DHS
Egypt	2005	0-59.99	National	12199	HAZ, WAZ	1	DHS
Egypt	2008	0-59.99	National	9345	HAZ, WAZ	1	DHS
El Salvador	1988	0-59.99	National	2002	HAZ, WAZ	2	Evaluacion de la situacion alimentaria nutricional en El Salvador (ESANES-88). Ministerio de Salud Publica y Asistencia Social. San Salvador, El Salvador, 1990 (and additional analysis).
El Salvador	1993	0-59.99	National	3598	HAZ, WAZ	2	Salvadoran Demographic Association. National family health survey 1993 (FESAL-93). San Salvador: Government of El Salvador, 1994 (and additional analysis).
El Salvador	1994	0-59.99	Regional	545	HAZ, WAZ	3,5	Ministerio de Salud. Informe final: Linea basal de la evaluacion programa nacional de educacion nutricional. San Salvador, El Salvador, 1995.
El Salvador	1998	3-59.99	National	6624	HAZ, WAZ	1	RHS El Salvador 1998
El Salvador	2002-2003	0-59.99	National	5250	HAZ, WAZ	1	RHS El Salvador 2002-2003
El Salvador	2008	0-59.99	National	4611	HAZ, WAZ	1	RHS El Salvador 2008
Equatorial Guinea	1992	0-59.99	Regional	1252	HAZ, WAZ	3,5	Ministère de la Santé de Guinée Equatoriale. Evaluation de l'état nutritionnel et de la mortalité infantile dans la région continentale de la Guinée Equatoriale. Document technique No 840/SG/DSP. Yaounde: Organisation de Cooperation pour la lutte contre les Endemes en Afrique Centrale, 1993.
Equatorial Guinea	1997	0-59.99	National	412	HAZ, WAZ	2	Custodio E, Descalzo MA, Roche J, Molina L, Sánchez I, Lwanga M et al. The economic and nutrition transition in Equatorial Guinea coincided with a double burden of over- and under nutrition. Economics and Human Biology 2010;8:80-87 (and additional analysis).
Equatorial Guinea	2000	0-59.99	National	2025	HAZ, WAZ	1	MICS
Equatorial Guinea	2004	0-59.99	National	553	HAZ, WAZ	2	Custodio E, Descalzo MA, Roche J, Sánchez I, Molina L, Lwanga M et al. Nutritional status and its correlates in Equatorial Guinean preschool children: Results from a nationally representative survey. Food and Nutrition Bulletin 2008;29:49-58 (and additional analysis).
Eritrea	1993	6-59.99	National	NR	HAZ, WAZ	3,5,7	Ministry of Finance and Development. Children and women in Eritrea: 1994. Government of the State of Eritrea/UNICEF Situation Analysis. Asmara, Eritrea, 1994 (and additional analysis).
Eritrea	1995-1996	0-35.99	National	2372	HAZ, WAZ	2	National Statistics Office [Eritrea] and Macro International Inc. Eritrea demographic and health survey 1995. Demographic and Health Surveys. Calverton, Maryland: National Statistics Office and Macro International Inc, 1997 (and additional analysis).
Eritrea	2002	0-59.99	National	5707	HAZ, WAZ	2	National Statistics and Evaluation Office (NSEO) [Eritrea] and ORC Macro. Eritrea demographic and health survey 2002. Demographic and Health Surveys. Calverton, Maryland, USA: National Statistics amd Evaluation Office and ORC Macro, 2003 (and additional analysis).
Ethiopia	2000	0-59.99	National	8686	HAZ, WAZ	1	DHS
Ethiopia	2005	0-59.99	National	3819	HAZ, WAZ	1	DHS
Ethiopia	2011	0-59.99	National	9553	HAZ, WAZ	1	DHS
Fiji	1993	0-59.99	National	618	HAZ, WAZ	3,5	1993 National nutrition survey - main report. Suva: National Food and Nutrition Committee, 1995 (and additional analysis).

Gabon	2000-2001	0-59.99	National	3428	HAZ, WAZ	1	DHS
Gambia	1996	0-59.99	National	2401	HAZ, WAZ	3,5	Central Statistics Department [The Gambia], et al. Report of the progress of the mid-decade goals in the Gambia (MICS), 1996. Banjul: The Republic of The Gambia and UNICEF, 1998 (and additional analysis).
Gambia	2000	0-59.99	National	2711	HAZ, WAZ	1	MICS
Gambia	2005-2006	0-59.99	National	6424	HAZ, WAZ	2	Gambia Bureau of Statistics (GBoS). The Gambia Multiple Indicator Cluster Survey 2005/2006 Report. Banjul, The Gambia: GBoS, 2007 (and additional analysis).
Georgia	1999	0-59.99	National	3434	HAZ, WAZ	3,5	Georgia multiple indicator cluster survey 1999 (MICS). Tibilisi, Georgia, 2000 (and additional analysis).
Georgia	2000	0-59.99	Regional	3938	HAZ, WAZ	2	Safe the Children, USAID, NCDC, and CDC. Nutritional status of children less than five years of age in six drought-affected regions of Georgia 2000-2001: Final report. Tibilisi, Georgia: d&p studio, 2002 (and additional analysis).
Georgia	2005	0-59.99	National	1826	HAZ, WAZ	1	MICS
Georgia	2009	0-59.99	National	3020	HAZ, WAZ	3	Georgia National Center for Disease Control and Public Health (NCDC&PH), UNICEF. Report of the Georgia national nutrition survey (GNNS) 2009. Tbilisi, Georgia: NCDC&PH and UNICEF, 2010.
Ghana	1987-1988	0-59.99	National	2949	HAZ, WAZ	3,5	Alderman H. Nutritional status in Ghana and its determinants. Living Standards Survey, Ghana 1987-88. Working paper No. 3. Washington, D.C.: The World Bank, 1989 (and additional analysis).
Ghana	1988	3-35.99	National	1967	HAZ, WAZ	1	DHS
Ghana	1993-1994	0-35.99	National	1873	HAZ, WAZ	1	DHS
Ghana	1998-1999	0-59.99	National	2741	HAZ, WAZ	1	DHS
Ghana	2003	0-59.99	National	3072	HAZ, WAZ	1	DHS
Ghana	2006	0-59.99	National	3324	HAZ, WAZ	1	MICS
Ghana	2008	0-59.99	National	2367	HAZ, WAZ	1	DHS
Guatemala	1987	3-35.99	National	2218	HAZ, WAZ	1	DHS
Guatemala	1995	0-59.99	National	8446	HAZ, WAZ	1	DHS
Guatemala	1998-1999	0-59.99	National	3862	HAZ, WAZ	1	DHS
Guatemala	2000	0-59.99	National	5415	HAZ, WAZ	3,5	Marini A and Gragnolati M. Malnutrition and poverty in Guatemala. Policy Research Working Paper 2967. The World Bank, Latin America and the Caribbean Region, Human Development Sector Unit, 2003 (and additional analysis).
Guatemala	2002	1-59.99	National	6363	HAZ, WAZ	1	RHS Guatemala 2002
Guatemala	2008-2009	0-59.99	National	10221	HAZ, WAZ	1	RHS Guatemala 2008-2009
Guinea	1990	0-59.99	Regional (rural)	1115	HAZ, WAZ	2,5	Mock NB, Magnani RJ, Abdoh AA, Kondé MK. Intra-household correlations in maternal-child nutritional status in rural Guinea: implications for programme-screening strategies. Bulletin of the World Health Organization 1994;72:119-127.
Guinea	1994-1995	3-59.99	National	3542	HAZ, WAZ	3,5	Enquête intégrale sur les conditions de vie des ménages avec module budget et consommation (EIBC) 1994-95. Ministère du Plan et de la Coopération, Direction Nationale de la Statistique. Conakry, République de Guinée, 1998.
Guinea	1999	0-59.99	National	4264	HAZ, WAZ	1	DHS

Guinea	2000	6-59.99	National	1457	HAZ, WAZ	3	Ministère de la Santé Publique, Direction Nationale de la Santé Publique, Section Alimentation-Nutri Enquête nationale sur l'anémie ferriprive en Guinée (rapport final:Version 3.0). Institut de Nutrition et de Santé de l'Enfant, République de Guinée, 2001.
Guinea	2005	0-59.99	National	2615	HAZ, WAZ	1	DHS
Guinea	2007-2008	0-59.99	National	11781	HAZ, WAZ	2	Ministère de l'Economie des Finances et du Plan, UNICEF, PAM and Direction Nationale de la Statistique. Enquête nationale sur l'état nutritionnel et le suivi des principaux indicateurs de survie de l'enfant. Rapport provisoire, Mai 2008 (30/12/09 http://ochaonline.un.org/CoordinationIASC/Securitealimentairenutrition/tabid/5651/language/fr-FR/Default.aspx).
Guinea-Bissau	2000	0-59.99	National	5358	HAZ, WAZ	1	MICS
Guinea-Bissau	2006	0-59.99	National	5291	HAZ, WAZ	2	Ministère de l`Economie - Secrétariat d`Etat du Plan et à l`Intégration Régionale. Enquête par grappes à indicateurs multiples, Guinée-Bissau, 2006, Rapport final. Bissau, Guinée-Bissau : Ministère de l`Economie - Secrétariat d`Etat du Plan et à l`Intégration Régionale, 2006 (and additional analysis).
Guinea-Bissau	2008	0-59.99	National	2710	HAZ, WAZ	3	Gouvernement de la Guinée Bissau et UNICEF. Enquête nutritionnel SMART. Evaluation de la situation nutritionnelle en Guinée Bissau: Rapport final, décembre 2008. (30/12/09 http://ochaonline.un.org/CoordinationIASC/Securitealimentairenutrition/tabid/5651/language/fr-FR/Default.aspx).
Guyana	1993	0-59.99	National	581	WAZ	3,5	Guyana Bureau of Statistics. Household income and expenditure survey 1993 and Guyana living standards measurement survey. In: Guyana strategies for reducing poverty. Report No. 12861-GUA. Washington: The World Bank, 1994:50-67 (and additional analysis).
Guyana	2000	0-59.99	National	2491	HAZ, WAZ	1	MICS
Guyana	2006-2007	0-59.99	National	2285	HAZ, WAZ	2	Bureau of Statistics and UNICEF. Guyana multiple indicator cluster survey 2006, final report. Georgetown, Guyana: Bureau of Statistics and UNICEF, 2008 (and additional analysis).
Guyana	2009	0-59.99	National	1551	HAZ, WAZ	1	DHS
Haiti	1990	3-59.99	Regional	967	HAZ, WAZ	3,5	Centres for Disease Control. Nutritional assessment of children in drought-affected areas - Haiti, 1990. Morbidity and Mortality Weekly Report 1991;40:222-225.
Haiti	1990	3-59.99	National	1843	HAZ, WAZ	3,5	Ministry of Public Health and Population. Haiti's nutrition situation in 1990: A report based on anthropometric data of the 1990 nutrition surveys. Port-au-Prince, Haiti, 1993 (and additional analysis).
Haiti	1994-1995	0-59.99	National	2734	HAZ, WAZ	1	DHS
Haiti	1995	0-59.99	First admin level	397	HAZ, WAZ	2,5,8	Nutritional surveillance for programme planning. Haiti 1995 nutrition surveys by department. Port-au-Prince, Haiti, 1997.
Haiti	1995	0-59.99	First admin level	385	HAZ, WAZ	2,5,8	Nutritional surveillance for programme planning. Haiti 1995 nutrition surveys by department. Port-au-Prince, Haiti, 1997.
Haiti	1995	0-59.99	First admin level	391	HAZ, WAZ	2,5,8	Nutritional surveillance for programme planning. Haiti 1995 nutrition surveys by department. Port-au-Prince, Haiti, 1997.

Haiti	1995	0-59.99	First admin level	394	HAZ, WAZ	2,5,8	Nutritional surveillance for programme planning. Haiti 1995 nutrition surveys by department. Port-au-Prince, Haiti, 1997.
Haiti	1995	0-59.99	First admin level	398	HAZ, WAZ	2,5,8	Nutritional surveillance for programme planning. Haiti 1995 nutrition surveys by department. Port-au-Prince, Haiti, 1997.
Haiti	1995	0-59.99	First admin level	392	HAZ, WAZ	2,5,8	Nutritional surveillance for programme planning. Haiti 1995 nutrition surveys by department. Port-au-Prince, Haiti, 1997.
Haiti	1995	0-59.99	First admin level	389	HAZ, WAZ	2,5,8	Nutritional surveillance for programme planning. Haiti 1995 nutrition surveys by department. Port-au-Prince, Haiti, 1997.
Haiti	1995	0-59.99	First admin level	395	HAZ, WAZ	2,5,8	Nutritional surveillance for programme planning. Haiti 1995 nutrition surveys by department. Port-au-Prince, Haiti, 1997.
Haiti	1995	0-59.99	First admin level	388	HAZ, WAZ	2,5,8	Nutritional surveillance for programme planning. Haiti 1995 nutrition surveys by department. Port-au-Prince, Haiti, 1997.
Haiti	1995	0-59.99	First admin level	396	HAZ, WAZ	2,5,8	Nutritional surveillance for programme planning. Haiti 1995 nutrition surveys by department. Port-au-Prince, Haiti, 1997.
Haiti	2000	0-59.99	National	5502	HAZ, WAZ	1	DHS
Haiti	2005-2006	0-59.99	National	2524	HAZ, WAZ	1	DHS
Honduras	1987	0-59.99	National	3244	HAZ, WAZ	3,5	Barahona F, Soto RJ, Tronconi E, Maradiaga A, O'Connor G, Corrales G. Encuesta nacional de nutricion, Honduras, 1987. Cuadros de frequencias por regiones de salud y nacionales. Ministerio de Salud Publica. Tegucigalpa, Republica de Honduras, 1988 (and additional analysis).
Honduras	1991-1992	0-59.99	National	5961	HAZ, WAZ	3,5	Ministerio de Salud Publica. Encuesta nacional de epidemiologia y salud familiar (ENESF), 1991/92. Tegucigalpa, Republica de Honduras, 1993 (and additional analysis).
Honduras	1993-1994	0-59.99	National	1875	HAZ, WAZ	3,5	National survey of socio-economic indicators 1993/94. Tegucigalpa, Republica de Honduras, 1996 (and additional analysis).
Honduras	1996	12-59.99	National	1455	HAZ, WAZ	2	Ministry of Health. National micronutrient survey Honduras 1996. Tegucigalpa, Republic of Honduras, 1997 (and additional analysis).
Honduras	2001	1-59.99	National	5624	HAZ, WAZ	1	RHS Honduras 2001
Honduras	2005-2006	0-59.99	National	9200	HAZ, WAZ	1	DHS
India	1988-1990	0-59.99	Regional	13548	HAZ, WAZ	3,5	National Institute of Nutrition. National Nutrition Monitoring Bureau, 1988-90 (8 States pooled data). Hyderabad, India; 1993 (data reanalyzed for WHO).
India	1991-1992	0-59.99	Regional (rural)	2948	HAZ, WAZ	3,5	National Institute of Nutrition. National Nutrition Monitoring Bureau, 1991-92 (8 States pooled data). Hyderabad, India; 1993 (data reanalyzed for WHO).
India	1992-1993	0-47.99	National	26565	HAZ, WAZ	1	DHS
India	1992-1993	0-35.99	First admin level (rural)	2010	HAZ, WAZ	3,5	Abel R and Sampathkumar V. Tamil Nadu nutritional survey comparing children aged 0-3 years withthe NCHS/CDC reference population. Indian Journal of Pediatrics 1998;65:565-572.
India	1995-1996	12-59.99	Regional	46457	HAZ, WAZ	3,5	Government of India. India nutrition profile. Department of Women and Child Development. Ministry of Human Resource Development. New Delhi, India, 1998.

India	1996-1997	0-59.99	Regional (rural)	22959	HAZ, WAZ	3,5	Vijayaraghavan K, Hanumantha Rao D. Diet and nutrition situation in rural India. Indian Journal of Medical Research 1998;108:243-253 (and additional analysis).
India	1998-2000	0-35.99	National	24605	HAZ, WAZ	1	DHS
India	2004-2005	0-59.99	Regional (rural)	5837	HAZ, WAZ	3	National Nutrition Monitoring Bureau. Diet & nutritional status of population and prevalence of hypertension among adults in rural areas. NNMB Technical Report No. 24. Hyderabad, India: National Institute of Nutrition, Indian Council of Medical Research, 2006.
India	2005-2006	0-59.99	National	40951	HAZ, WAZ	1	DHS
Indonesia	1987	0-59.99	National	28169	WAZ	3,5	National socioeconomic survey 1987 (SUSENAS-1987). Central Bureau of Statistics. Jakarta, Indonesia, 1992 (and additional analysis).
Indonesia	1989	0-59.99	National	14101	WAZ	2	Atmarita, Jahari AB, Latief D, Soekirman, Tilden RL. The effect of economic crisis on the nutritional status of Indonesian pre-school children. Gizi Indonesia 2000;33-41 (and additional analysis), and http://www.gizi.net, accessed 18/12/03.
Indonesia	1992	0-59.99	National	33742	WAZ	2	Atmarita, Jahari AB, Latief D, Soekirman, Tilden RL. The effect of economic crisis on the nutritional status of Indonesian pre-school children. Gizi Indonesia 2000;33-41 (and additional analysis), and http://www.gizi.net, accessed 18/12/03.
Indonesia	1991-1993	0-59.99	Regional (rural)	3338	HAZ, WAZ	2,5	Sastroamidjodjo S, Gross R, Schultink W. SEAMEO-GTZ combined nutrition surveys. Jakarta: SEAMO-TROPMED-GTZ, 1994.
Indonesia	1993	0-59.99	Regional	2410	HAZ, WAZ	1	IFLS 1
Indonesia	1995	0-59.99	National	9227	HAZ, WAZ	3,5	Indonesia multiple indicator cluster survey (MICS) 1995. Jakarta: UNICEF, 1997 (preliminary results provided by the Centers for Disease Control and Prevention; and additional analysis).
Indonesia	1995	0-59.99	National	26073	WAZ	2	National socioeconomic survey (SUSENAS) 1995. Central Bureau of Statistics. Jakarta, Indonesia, 1998 (and additional analysis).
Indonesia	1997	0-59.99	Regional	2522	HAZ, WAZ	1	IFLS 2
Indonesia	1998	0-59.99	National	25505	WAZ	2	Atmarita, Jahari AB, Latief D, Soekirman, Tilden RL. The effect of economic crisis on the nutritional status of Indonesian pre-school children. Gizi Indonesia 2000;33-41 (and additional analysis), and http://www.gizi.net, accessed 18/12/03.
Indonesia	1999	0-59.99	First admin level (rural)	6898	HAZ, WAZ	2,5	Nutrition and health surveillance system (NNS) - Monitoring the economic crisis: Impact and Transition, 1998-2000. Jakarta: Helen Keller International/Indonesia, 2000 (and additional analysis: rural areas in Central Java).
Indonesia	1999	0-59.99	National	78849	WAZ	2	Atmarita, Jahari AB, Latief D, Soekirman, Tilden RL. The effect of economic crisis on the nutritional status of Indonesian pre-school children. Gizi Indonesia 2000;33-41 (and additional analysis), and http://www.gizi.net, accessed 18/12/03.
Indonesia	1999	0-59.99	First admin level (rural)	5723	HAZ, WAZ	2,5	Nutrition and health surveillance system (NNS) - Monitoring the economic crisis: Impact and Transition, 1998-2000. Jakarta: Helen Keller International/Indonesia, 2000 (and additional analysis: rural areas in West Sumatra).

Indonesia	1999-2000	0-59.99	First admin level (rural)	3502	HAZ, WAZ	2,5	Nutrition and health surveillance system (NNS) - Monitoring the economic crisis: Impact and Transition, 1998-2000. Jakarta: Helen Keller International/Indonesia, 2000 (and additional analysis: rural areas of East Java).
Indonesia	1999-2000	0-59.99	First admin level (rural)	4705	HAZ, WAZ	2,5	Nutrition and health surveillance system (NNS) - Monitoring the economic crisis: Impact and Transition, 1998-2000. Jakarta: Helen Keller International/Indonesia, 2000 (and additional analysis: rural areas in West Java).
Indonesia	2000	0-59.99	National	106147	HAZ, WAZ	2	New Insight on the Health & Nutrition Situation in Indonesia through Data Sharing: Annual Report 2000-2001. Summary: Second Annual Report of the Nutrition and Health Surveillance System in Indonesia with data from the period 2000-2001, http://www.hki.org/research/nutrition_surveillance.html (and additional analysis).
Indonesia	2000	0-59.99	Regional	3673	HAZ, WAZ	1	IFLS 3
Indonesia	2000	0-59.99	National	70591	WAZ	2	Atmarita, Jahari AB, Latief D, Soekirman, Tilden RL. The effect of economic crisis on the nutritional status of Indonesian pre-school children. Gizi Indonesia 2000;33-41 (and additional analysis), and http://www.gizi.net, accessed 18/12/03.
Indonesia	2001	0-59.99	National	11678	WAZ	2	Atmarita, Tilden R, Noor Nastry Nur, Ascobat Gani, Widjajanto RM. Indonesian nutritional status of children 1989-2005: Poverty and household food security, dietary diversity and infection: which is the most important risk? Gizi Indonesia 2005;28: (and additional analysis).
Indonesia	2001	0-59.99	National	96417	HAZ, WAZ	2	New Insight on the Health & Nutrition Situation in Indonesia through Data Sharing: Annual Report 2000-2001. Summary: Second Annual Report of the Nutrition and Health Surveillance System in Indonesia with data from the period 2000-2001, http://www.hki.org/research/nutrition_surveillance.html (and additional analysis).
Indonesia	2002	0-59.99	National	74360	WAZ	2	Atmarita, Tilden R, Noor Nastry Nur, Ascobat Gani, Widjajanto RM. Indonesian nutritional status of children 1989-2005: Poverty and household food security, dietary diversity and infection: which is the most important risk? Gizi Indonesia 2005;28: (and additional analysis).
Indonesia	2003	0-59.99	National	77110	WAZ	2	National socioeconomic survey 2003 (SUSENAS-2003). Central Bureau of Statistics. Jakarta, Indonesia, 2006 (and additional analysis).
Indonesia	2004	0-59.99	National	3116	HAZ, WAZ	2	Ministry of Health. National Institute of Health Research and Development, 2005. National Household Health Survey (SKRT) 2004, Volume 2: Community Health Status in Indonesia. Jakarta, Indonesia, 2007 (and additional analysis).
Indonesia	2005	0-59.99	National	94652	WAZ	2	National socioeconomic survey 2005 (SUSENAS-2005). Central Bureau of Statistics. Jakarta, Indonesia, 2006 (and additional analysis).

Indonesia	2007	0-59.99	National	77808	HAZ, WAZ	2	Ministry of Health and National Institute of Health Research and Development. Basic Health Survey, Riskesdas, 2007. Results to be presented at the ICN in Bangkok, October 2009: "Changes in malnutrition from 1989 to 2007 in Indonesia" by Ita Atmarita, Ministry of Health.
Indonesia	2007	0-59.99	Regional	4541	HAZ, WAZ	1	IFLS 4
Iran (Islamic Republic of)	1995	0-59.99	National	11139	HAZ, WAZ	3,5	Undersecretary for Public Affairs, Ministry of Health and Medical Education. Cluster survey for evaluation of mid decade goal indicators (MICS). Theran, Islamic Republic of Iran, April 1996 (and additional analysis).
Iran (Islamic Republic of)	1998	0-59.99	National	2536	HAZ, WAZ	3,5	The nutritional status of children, October-November 1998 (ANIS). Teheran: Ministry of Health and Medical Education and UNICEF, 2000 (and additional analysis).
Iran (Islamic Republic of)	2001	13-28.99	National	8432	HAZ, WAZ	1	Iran Micronutrient Survey 2001
Iraq	1991	0-59.99	Regional	680	HAZ, WAZ	3,5	Field JO, Russell RM. Nutrition mission to Iraq for UNICEF. Nutrition Reviews 1992;50:41-46.
Iraq	1991	0-59.99	National	2565	HAZ, WAZ	3,5	International Study Team. Infant and child mortality and nutritional status of Iraqi children after the Gulf conflict: Results of a community-based study. Center for Population and Development Studies, Harvard University, Cambridge, MA, USA; 1992 (and additional analysis).
Iraq	1996	0-59.99	Regional	6392	HAZ, WAZ	3,5	The 1996 multiple indicator cluster survey: a survey to assess the situation of children and women in Iraq (MICS). Central Statistical Organizations and UNICEF. Baghdad, Iraq 1997.
Iraq	1997	0-11.99	Regional	3102	HAZ, WAZ	3,4,5	Ministry of Health and UNICEF/Iraq. Nutritional status survey of infants in Iraq (excluding the Autonomous Northern Governorates) - final report. Baghdad, Iraq, 1997.
Iraq	1997	0-59.99	Regional	2248	HAZ, WAZ	3,5	Ministry of Health and Social Welfare and UNICEF/Iraq. Nutritional status of children under five in the Autonomous Northern Region, Iraq. Baghdad, Iraq, 1998 (pre-final draft).
Iraq	1998	0-11.99	Regional	3622	HAZ, WAZ	3,4,5	Nutritional status survey of infants in Iraq (15 governorates in the South/Centre). Baghdad, Iraq, 1998.
Iraq	1998	0-59.99	Regional	13892	HAZ, WAZ	3,4,5	Nutritional status survey of infants in Iraq (15 governorates in the South/Centre). Baghdad, Iraq, 1998.
Iraq	1999	0-59.99	Regional	13738	HAZ, WAZ	3,4,5	Ministry of Health and UNICEF/Iraq. Nutrition survey in primary health centres on polio national immunization days in centre/south Iraq (final draft). Baghdad, Iraq, 1999.
Iraq	1999	0-23.99	Regional	6414	HAZ, WAZ	3,4,5	Ministry of Health and UNICEF. Nutrition survey of children under two attending rotine immunization sessions at primary health care centres in Iraq. Baghdad, Iraq, 1999.
Iraq	2000	0-59.99	National	13861	HAZ, WAZ	1	MICS

Iraq	2002	0-59.99	Regional	24489	HAZ, WAZ	3,5	Ministry of Health, Directorate of Preventive Health, Nutrition Research Institute, Ministry of Planning, Central Statistical Organisation and UNICEF. Integrated nutritional status survey of under five years and breastfeeding/ complementary feeding practices of under two years in South/Center Iraq. Baghdad, Iraq: UNICEF, 2002.
Iraq	2003	0-59.99	National	NR	HAZ, WAZ	3,5,7	Baseline food security analysis in Iraq. Baghdad, Iraq: WFP Iraq Country Office, September 2004 (and additional analysis).
Iraq	2004	0-59.99	National	16464	HAZ, WAZ	3,5	Iraq living conditions survey 2004. Volume 1: Tabulation report. Baghdad, Iraq: Central Organization for Statistics and Information Technology, Ministry of Planning and Development Cooperation, 2005 (and additional analysis).
Iraq	2006	0-59.99	National	16309	HAZ, WAZ	2	Central Organization for Statistics & Information Technology and Kurdistan Regional Statistical Office. Iraq multiple indicator cluster survey 2006, Final report. Iraq, 2007 (and additional analysis).
Jamaica	1989	0-59.99	National	860	HAZ, WAZ	3,5	Jamaica Living Standards and Measurement Survey 1989. Kingston: The World Bank, 1990 (and additional analysis).
Jamaica	1991	0-59.99	National	358	HAZ, WAZ	3,5,8	Jamaica survey of living conditions - report 1991. Kingston: The Planning Institute and the Statistical Institute of Jamaica, 1992 (and additional analysis).
Jamaica	1992	0-59.99	National	1327	HAZ, WAZ	3,5	Jamaica survey of living conditions 1992. Kingston: The Planning Institute and the Statistical Institute of Jamaica, 1994 (and additional analysis).
Jamaica	1993	0-59.99	National	663	HAZ, WAZ	3,5	Jamaica survey of living conditions 1993. Kingston: The Planning Institute and the Statistical Institute of Jamaica, 1995 (and additional analysis).
Jamaica	1994	0-59.99	National	982	HAZ, WAZ	2	Jamaica survey of living conditions, 1994. Kingston: The Planning Institute and the Statistical Institute of Jamaica, 1996 (and additional analysis).
Jamaica	1995	0-59.99	National	959	HAZ, WAZ	2	Jamaica survey of living conditions, 1995. Kingston: The Planning Institute and the Statistical Institute of Jamaica, 1997 (and additional analysis).
Jamaica	1996	0-59.99	National	1001	HAZ, WAZ	2	Jamaica survey of living conditions, 1996. Kingston: The Planning Institute and the Statistical Institute of Jamaica, 1997 (and additional analysis).
Jamaica	1997	0-59.99	National	931	HAZ, WAZ	2	Jamaica survey of living conditions, 1997. Kingston: The Planning Institute and the Statistical Institute of Jamaica, 1998 (and additional analysis).
Jamaica	1998	0-59.99	National	3142	HAZ, WAZ	2	Jamaica survey of living conditions, 1998. Kingston: The Planning Institute and the Statistical Institute of Jamaica, 1999 (and additional analysis).
Jamaica	1999	0-59.99	National	810	HAZ, WAZ	2	Jamaica survey of living conditions, 1999. Kingston: The Planning Institute and the Statistical Institute of Jamaica, 2000 (and additional analysis).
Jamaica	2000	0-59.99	National	770	HAZ, WAZ	2	Jamaica survey of living conditions data set 2000. The Planning Institute and the Statistical Institute of Jamaica, 2001 (and additional analysis).
Jamaica	2001	0-59.99	National	631	HAZ, WAZ	2	Jamaica survey of living conditions data set 2001. The Planning Institute and the Statistical Institute of Jamaica, 2002 (and additional analysis).

Jamaica	2002	0-59.99	National	3275	HAZ, WAZ	2	Jamaica survey of living conditions data set 2002. The Planning Institute and the Statistical Institute of Jamaica, 2003 (and additional analysis).
Jamaica	2004	0-59.99	National	793	HAZ, WAZ	2	Jamaica survey of living conditions data set 2004. The Planning Institute and the Statistical Institute of Jamaica, 2005 (and additional analysis).
Jamaica	2006	0-59.99	National	454	HAZ, WAZ	3,5	Jamaica survey of living conditions data set 2006. The Planning Institute and the Statistical Institute of Jamaica, 2009 (and additional analysis).
Jamaica	2007	0-59.99	National	500	HAZ, WAZ	3,5	Jamaica survey of living conditions data set 2007. The Planning Institute and the Statistical Institute of Jamaica, 2009 (and additional analysis).
Jordan	1990	0-59.99	National	6596	HAZ, WAZ	1	DHS
Jordan	1991	0-59.99	National	8113	HAZ, WAZ	3,5	Ministry of Health. Assessment of the nutritional status of preschool children in Jordan. Amman: Department of Statistics, 1993.
Jordan	1997	0-59.99	National	5578	HAZ, WAZ	1	DHS
Jordan	2002	0-59.99	National	4858	HAZ, WAZ	1	DHS
Jordan	2007	0-59.99	National	4371	HAZ, WAZ	1	DHS
Jordan	2009	0-59.99	National	4354	HAZ, WAZ	1	DHS
Kazakhstan	1995	0-35.99	National	733	HAZ, WAZ	1	DHS
Kazakhstan	1999	0-59.99	National	565	HAZ, WAZ	1	DHS
Kazakhstan	2006	0-59.99	National	4227	HAZ, WAZ	1	MICS
Kenya	1987	6-59.99	National (rural)	6957	HAZ	2,5	Fourth Rural Child Nutrition Survey, 1987. Central Bureau of Statistics, Ministry of Planning and National Development. Republic of Kenya, Nairobi; 1991.
Kenya	1993	0-59.99	National	4880	HAZ, WAZ	1	DHS
Kenya	1994	6-59.99	National	8944	HAZ, WAZ	3,5	Central Bureau of Statistics. Fifth child nutrition survey, 1994. Welfare monitoring survey. Nairobi, Kenya, 1995.
Kenya	1998	0-35.99	National	2887	HAZ, WAZ	1	DHS
Kenya	2000	0-59.99	National	6091	HAZ, WAZ	1	MICS
Kenya	2003	0-59.99	National	4689	HAZ, WAZ	1	DHS
Kenya	2005-2006	6-59.99	National	9985	HAZ, WAZ	3,5	Kenya integrated household budget survey (KIHBS), 2006/06: Revised edition, basic report. Nairobi, Kenya 2006 (and additional analysis).
Kenya	2008-2009	0-59.99	National	5058	HAZ, WAZ	1	DHS
Kiribati	1985	0-59.99	National	2941	HAZ, WAZ	3,5	Ministry of Health and Family Planning. National nutrition survey, 1985. Government of Kiribati. South Tarawa, Republic of Kiribati, 1990 (draft version; and additional analysis).
Kuwait	1994-1995	6-59.99	National	1280	HAZ, WAZ	3,5	Amine EK, Al-Awadi FA. Nutritional status survey of preschool children in Kuwait. Eastern Mediterranean Health Journal 1996;2:386-394.
Kuwait	1996-1997	0-59.99	National	12376	HAZ, WAZ	3,4,5	Kuwait national nutritional surveillance system. Nutrition unit, Ministry of Health. Al Shaab, Kuwait, 1998 (and additional analysis).
Kuwait	2001	0-59.99	National	4878	HAZ, WAZ	2,4	Administration of Food and Nutrition, Ministry of Health. Kuwait nutrition surveillance system, 2005 report: 2001-2005 trends. Kuwait, State of Kuwait: Administration of Food and Nutrition, Ministry of Health, 2006 (and additional analysis).
Kuwait	2002	0-59.99	National	3842	HAZ, WAZ	2,4	Administration of Food and Nutrition, Ministry of Health. Kuwait nutrition surveillance system, 2005 report: 2001-2005 trends. Kuwait, State of Kuwait: Administration of Food and Nutrition, Ministry of Health, 2006 (and additional analysis).

Kuwait	2003	0-59.99	National	4308	HAZ, WAZ	2,4	Administration of Food and Nutrition, Ministry of Health. Kuwait nutrition surveillance system, 2005 report: 2001-2005 trends. Kuwait, State of Kuwait: Administration of Food and Nutrition, Ministry of Health, 2006 (and additional analysis).
Kuwait	2004	0-59.99	National	4381	HAZ, WAZ	2,4	Administration of Food and Nutrition, Ministry of Health. Kuwait nutrition surveillance system, 2005 report: 2001-2005 trends. Kuwait, State of Kuwait: Administration of Food and Nutrition, Ministry of Health, 2006 (and additional analysis).
Kuwait	2005	0-59.99	National	5601	HAZ, WAZ	2,4	Administration of Food and Nutrition, Ministry of Health. Kuwait nutrition surveillance system, 2005 report: 2001-2005 trends. Kuwait, State of Kuwait: Administration of Food and Nutrition, Ministry of Health, 2006 (and additional analysis).
Kuwait	2006	0-59.99	National	3422	HAZ, WAZ	2,4	Administration of Food and Nutrition, Ministry of Health. Kuwait nutrition surveillance system: 2006-2009 trends. Kuwait, State of Kuwait: Administration of Food and Nutrition, Ministry of Health, 2010 (and additional analysis).
Kuwait	2007	0-59.99	National	3949	HAZ, WAZ	2,4	Administration of Food and Nutrition, Ministry of Health. Kuwait nutrition surveillance system: 2006-2009 trends. Kuwait, State of Kuwait: Administration of Food and Nutrition, Ministry of Health, 2010 (and additional analysis).
Kuwait	2008	0-59.99	National	4199	HAZ, WAZ	2,4	Administration of Food and Nutrition, Ministry of Health. Kuwait nutrition surveillance system: 2006-2009 trends. Kuwait, State of Kuwait: Administration of Food and Nutrition, Ministry of Health, 2010 (and additional analysis).
Kuwait	2009	0-59.99	National	4099	HAZ, WAZ	2,4	Administration of Food and Nutrition, Ministry of Health. Kuwait nutrition surveillance system: 2006-2009 trends. Kuwait, State of Kuwait: Administration of Food and Nutrition, Ministry of Health, 2010 (and additional analysis).
Kyrgyzstan	1997	0-35.99	National	963	HAZ, WAZ	1	DHS
Kyrgyzstan	2005-2006	0-59.99	National	2875	HAZ, WAZ	1	MICS
Lao People's Democratic Republic	1986	0-59.99	Regional	6967	HAZ	3,5	Vijayaraghavan K. A nutritional surveillance report for the Lao People's Democratic Republic. Assignment Report (WP)NUT/LAO/NUT/001-E. Geneva: World Health Organization, 1988.
Lao People's Democratic Republic	1993	0-59.99	National	1365	HAZ, WAZ	3,5	Ministry of Public Health. Women and children in the Lao People's Democratic Republic. Results from the LAO social indicator survey (LSIS). Vientiane: Mother and Child Institute, 1994 (and additional analysis).
Lao People's Democratic Republic	1994	0-59.99	National	2950	HAZ, WAZ	3,5	Diagnostic de la situation nutritionnel et consommationn alimentaire au Laos. Rapport complet de l'étude sur l'état nutritionnel de la population Laotienne. ESNA: TCP/LAO/2354. Rome: Food and Agriculture Organization, 1995 (and additional analysis).
Lao People's Democratic Republic	2000	0-59.99	National	1444	HAZ, WAZ	1	MICS

Lao People's Democratic Republic	2006	0-59.99	National	3976	HAZ, WAZ	1	MICS
Lebanon	1996	0-59.99	National	1782	HAZ, WAZ	1	PAPCHILD Lebanon
Lebanon	2004	0-59.99	National	1129	HAZ, WAZ	2	The Arab League and the Repubic of Lebanon. Lebanon family health survey 2004: Principal report. Tutelian M, Khayyat M , Monem AA, eds. The Pan Arab Project for Family Health, 2006 (additional analysis conducted by PAPFAM).
Lesotho	1992	0-59.99	National	4687	HAZ, WAZ	3,5	Ministries of Health and Agriculture. National nutrition survey report, May-June 1992. Maseru, Kingdom of Lesotho, 1992 (and additional analysis).
Lesotho	1994	24-59.99	National	449	HAZ, WAZ	3,5	Ministry of Health. National survey on iodine, vitamin A and iron status of women and children in Lesotho. Maseru, Lesotho, 1994.
Lesotho	1996	0-59.99	National	2823	HAZ, WAZ	3,5	Spring CA. Mid-decade goals: progress towards the world summit, May 1996 (MICS). Maseru: Bureau of Statistics and UNICEF, 1996.
Lesotho	2000	0-59.99	National	3042	HAZ, WAZ	1	MICS
Lesotho	2004-2005	0-59.99	National	1353	HAZ, WAZ	1	DHS
Lesotho	2009-2010	0-59.99	National	1600	HAZ, WAZ	1	DHS
Liberia	1999-2000	0-59.99	National	4702	HAZ, WAZ	2	Ministry of Health and Social Welfare, UNICEF, Christian Health Association of Liberia. Liberia national nutrition survey 1999-2000. Monrovia, Liberia, 2001 (and additional analysis).
Liberia	2006-2007	0-59.99	National	4311	HAZ, WAZ	1	DHS
Libyan Arab Jamahiriya	1995	0-59.99	National	4440	HAZ, WAZ	1	PAPCHILD Libyan Arab Jamahiriya
Libyan Arab Jamahiriya	2007	0-59.99	National	10723	HAZ, WAZ	2	National Center for Infectious and Chronic Disease Control [Jamahiriya] and Pan-Arab Project for Family Health. National Libyan family health survey. PAPFAM surveys. Cairo: The league of Arab States, 2008 (and additional analysis conducted by PAPFAM).
Madagascar	1985	0-59.99	Regional	1000	HAZ, WAZ	3,5	Ministère de la Recherche Scientifique et Technologique pour le Developpement. Synthèse des données existantes sur l'état nutritionnel à Madagascar. Antananarivo, Madagascar, 1990.
Madagascar	1986	0-59.99	Regional	1000	HAZ, WAZ	3,5	Ministère de la Recherche Scientifique et Technologique pour le Developpement. Synthèse des données existantes sur l'état nutritionnel à Madagascar. Antananarivo, Madagascar, 1990.
Madagascar	1992	0-59.99	National	4116	HAZ, WAZ	1	DHS
Madagascar	1993-1994	3-59.99	National	3131	HAZ, WAZ	3,5	Institut National de la Statistique. Enquête permanente aupres des menages - rapport principal, décembre 1995. Antananarivo, Madagascar, 1995 (and additional analysis).
Madagascar	1995	0-59.99	National	5049	HAZ, WAZ	3,5	Institut National de la Statistique et UNICEF. Enquête par grappes a indicateurs multiples [multiple indicators cluster survey (MICS)], Madagascar 1995 (rapport préliminaires). Antananarivo, Madagascar: Institut National de la Statistique et UNICEF, 1996.
Madagascar	1997	0-35.99	National	2989	HAZ, WAZ	1	DHS
Madagascar	2003-2004	0-59.99	National	4406	HAZ, WAZ	1	DHS

Madagascar	2008-2009	0-59.99	National	4840	HAZ, WAZ	1	DHS
Malawi	1992	0-59.99	National	3177	HAZ, WAZ	1	DHS
Malawi	1995	6-59.99	National	3654	HAZ, WAZ	3,5	Ministry of Economic Planning and Development. Malawi social indicators survey 1995. MICS surveys. National Statistical Office and the Centre for Social Research. Lilongwe, Malawi, 1996 (and additional analysis).
Malawi	1997-1998	6-59.99	National	639	HAZ, WAZ	3,5	Government of Malawi. A relative profile of poverty in Malawi, 1998: A quintile-based poverty analysis of the Malawi integrated household survey, 1997-98. Poverty Monitoring System. Copied from www.nso.malawi.net; accessed 26/05/03 (and additional analysis).
Malawi	2000	0-59.99	National	9083	HAZ, WAZ	1	DHS
Malawi	2004-2005	0-59.99	National	7967	HAZ, WAZ	1	DHS
Malawi	2006	0-59.99	National	21207	HAZ, WAZ	1	MICS
Malawi	2009	6-59.99	National	981	HAZ, WAZ	3	UNICEF and CDC. The national micronutrient survey 2009. Lilongwe, Malawi: Ministry of Health, UNICEF, Irish Aid and CDC, 2011.
Malawi	2010	0-59.99	National	4527	HAZ, WAZ	1	DHS
Malaysia	1990	0-59.99	First admin level (rural)	641	HAZ	3,5	Kiyu A, Teo B, Hardin S, Ong F. Nutritional status of children in rural Sarawak, Malaysia. Southeast Asian Journal of Tropical Medicine and Public Health 1991;22:211-215.
Malaysia	1999	0-59.99	National	5108	HAZ, WAZ	3,5	Somsiah Parman. A study of malnutrition in under five children in Malaysia. Kuala Lumpur, Malaysia: Ministry of Health, 2000 (and additional analysis).
Malaysia	2006	0-59.99	National	5546	HAZ, WAZ	3	Khor GL, Noor Safiza MN, Jamalludin AB, Jamaiyah H, Geeta A, Kee CC et al. Nutritional status of children below five years in Malaysia: Anthropometric analyses from the third national health and morbidity survey III (NHMS, 2006). Malaysian Journal of Nutrition 2009;15:121-36.
Maldives	1994	0-59.99	National	1995	HAZ, WAZ	2	Ministry of Health and Welfare. Nutritional status and child feeding practices of Maldivian children. Department of Public Health. Male, Maldives, 1994.
Maldives	1995	0-59.99	National	798	HAZ, WAZ	3,5	Maldives multiple indicator survey report (MICS). United Nations Children's Fund. Malé, Maldives, June 1996 (and additional analysis).
Maldives	1997-1998	0-59.99	National	1512	HAZ, WAZ	2	Ministry of Planning and National Development and United Nations Development Programme. Vulnerability and poverty assessment 1998. Male', Republic of Maldives, 1999 (and additional analysis).
Maldives	2001	0-59.99	National	746	HAZ, WAZ	2	Damodar Sahu. Multiple indicator cluster survey (MICS 2) Maldives (draft). Ministry of Health. Male, Republic of Maldives, 2001 (and additional analysis).
Mali	1987	3-35.99	National	1521	HAZ, WAZ	1	DHS
Mali	1990	6-59.99	Regional (rural)	1171	HAZ, WAZ	2,5	La situation alimentaire et nutritionnelle au niveau des ménages et le role des femmes ... au Gourma et à Koutiala. Rapport d'Etape, Sécurité Alimentaire/Femmes, Centre National de la Recherche Scientifique et Technologique, Bamako, Mali, 1992.

Mali	1990	6-59.99	Regional (rural)	331	HAZ, WAZ	2,5,8	La situation alimentaire et nutritionnelle au niveau des ménages et le role des femmes ... au Gourma et à Koutiala. Rapport d'Etape, Sécurité Alimentaire/Femmes, Centre National de la Recherche Scientifique et Technologique, Bamako, Mali, 1992.
Mali	1990	6-59.99	Regional (rural)	842	HAZ, WAZ	2,5,8	La situation alimentaire et nutritionnelle au niveau des ménages et le role des femmes ... au Gourma et à Koutiala. Rapport d'Etape, Sécurité Alimentaire/Femmes, Centre National de la Recherche Scientifique et Technologique, Bamako, Mali, 1992.
Mali	1991	6-59.99	Regional (rural)	502	HAZ, WAZ	2,5	La situation alimentaire et nutritionnelle au niveau des ménages et le role des femmes ... au Gourma et à Koutiala. Rapport d'Etape, Sécurité Alimentaire/Femmes, Centre National de la Recherche Scientifique et Technologique, Bamako, Mali, 1992.
Mali	1992	12-59.99	Regional (rural)	145	HAZ, WAZ	2,5,8	La situation alimentaire et nutritionnelle au niveau des ménages et le role des femmes ... au Gourma et à Koutiala. Rapport d'Etape, Sécurité Alimentaire/Femmes, Centre National de la Recherche Scientifique et Technologique, Bamako, Mali, 1992.
Mali	1995-1996	0-35.99	National	4634	HAZ, WAZ	1	DHS
Mali	1996	6-59.99	National	NR	HAZ, WAZ	3,5,7	Enquête à indicateurs multiples au Mali (EIM) 1996 (MICS). Rapport d'Analyse. Direction Nationale de la Statistique et de l'Informatique (DNSI). Bamako, Mali, 1996 (and additional analysis).
Mali	2001	0-59.99	National	9291	HAZ, WAZ	1	DHS
Mali	2006	0-59.99	National	10746	HAZ, WAZ	1	DHS
Mauritania	1988	0-59.99	National	931	HAZ	3,5	Elder JA. The socio-economic determinants of nutritional status among children under five in Mauritania. Social Dimensions of Adjustment Surveys. Washington D.C.: The World Bank, 1990 (and additional analysis).
Mauritania	1990	0-59.99	National	3613	HAZ, WAZ	1	PAPCHILD Mauritania
Mauritania	1995-1996	0-59.99	National	3733	HAZ, WAZ	3,5	Ministère du Plan, Direction des Ressources Humaines. Enquête nationale sur les indicateurs des objectifs à mi-terme en Mauritanie (MICS). Nouakchott, République Islamique de Mauritanie, 1996 (and additional analysis).
Mauritania	1996	0-59.99	Regional (rural)	1351	HAZ, WAZ	3,5	Ministère de la Santé et des Affaires Sociales. Resultats de l'enquête nutritionnelle dans les moughataas de: Amourj (Hodh el Chargui), Koboni (Hodh el Gharbi), Bababe (Brakna). Nouakchott, Rebublique Islamique de Mauritanie, 1996.
Mauritania	2000-2001	0-59.99	National	4297	HAZ, WAZ	2	Office National de la Statistique (ONS) [Mauritania] et ORC Macro. Enquête démographique et de santé Mauritanie 2000-2001. Demographic and Health Surveys. Calverton, Maryland, USA: ONS et ORC Macro, 2001 (and additional analysis).
Mauritania	2007	0-59.99	National	7891	HAZ, WAZ	1	MICS
Mauritania	2008	0-59.99	National	6338	HAZ, WAZ	2	ANED, Office National de la Statistique (ONS) et UNICEF. Enquête rapide nationale sur la nutrition et survie de l'enfant en Mauritanie: Rapport final. Nouakchott, Mauritanie: ANED, ONS et UNICEF, 2008 (and additional analysis).

Mauritius	1985	0-59.99	National	2430	HAZ, WAZ	3,5	Ministry of Health. Mauritius national nutrition survey 1985: summary report. Evaluation and Nutrition Unit. Port Louis, Mauritius, 1988 (and additional analysis).
Mauritius	1995	0-59.99	National	1537	HAZ, WAZ	3,5	Ministry of Health. A survey on nutrition in Mauritius and Rodrigues, 1995 (final report). Port Louis, Mauritius, 1996 (and additional analysis).
Mexico	1988	0-59.99	National	7422	HAZ, WAZ	3,5	Sepulveda AJ, Lezana MA, Tapia Conyer R, Valdespino IL, Madrigal H, Kumate J. Estado nutricional de preescolares y las mujeres en Mexico: resultados de una encuesta probabilistica nacional. Gaceta Medica de Mexico 1990;126:207-226 (and additional analysis).
Mexico	1989	0-59.99	National (rural)	14932	HAZ, WAZ	2	Encuesta nacional de alimentacion en el medio rural, 1989 (ENAL). Instituto de la Nutricion 'Salvador Zubiran'. Comision Nacional de Alimentacion. Division de Nutricion, INNSZ. Tlalpan, Mexico, D.F, 1990 (and additional analysis).
Mexico	1991	12-59.99	First admin level	935	WAZ	3,5	Hernandez Martinez E, Roldan Fernandez SG. [Prevalence of malnutrition in preschool children in Tabasco, Mexico.] Salud Publica de Mexico 1995;37:211-218.
Mexico	1995	0-59.99	First admin level (urban)	1093	HAZ, WAZ	3,5	Encuesta urbana de alimentacion y nutricion en la zona metropolitana de la ciudad de Mexico, ENURBAL 1995. Instituto Nacional de la Nutricion ' Salvador Zubiran' . Mexico, 1998.
Mexico	1996	0-59.99	National (rural)	31601	HAZ, WAZ	3,5	Avila Curiel A, Shamah Levy T, Galindo Gomez C, Rodriguez Hernandez G, Barragan Heredia LM. La desnutricion infantil en el medio rural mexicano. Salud Publica de Mexico 1998;40:150-160.
Mexico	1999	0-59.99	National	7618	HAZ, WAZ	1	Nutrition 1999
Mexico	2005-2006	1-59.99	National	7953	HAZ, WAZ	1	ENSANUT 2005-2006
Micronesia (Fed. States of)	2000	24-59.99	Regional	485	HAZ	3,5	Vitamin A deficiency among children--Federated States of Micronesia, 2000. Morbidity and Mortality Weekly Report 2001;50(24):509-12.
Mongolia	1992	0-47.99	National	1679	HAZ, WAZ	3,5	Kachondham Y. Report of a consultancy on the Mongolian Child Nutrition Survey. Institute of Nutrition. Nakornpathom, Thailand, 1992 (and additional analysis).
Mongolia	1997	0-59.99	Regional	439	HAZ, WAZ	2	Nutrition Research Centre. Mongolia nutrition research centre survey 1997. Ulaanbaatar, Mongolia, 1999 (and additional analysis).
Mongolia	1999	0-59.99	National	4146	HAZ, WAZ	2	Kachondham Y. Report on the 2nd national child and nutrition survey, Mongolia 1999. Institute of Nutrition and Faculty of Medicine, Ramathibodi Hospital, Mahidol University, Thailand, 2000 (and additional analysis).
Mongolia	2000	0-59.99	National	5811	HAZ, WAZ	1	MICS
Mongolia	2004	6-59.99	National	1247	HAZ, WAZ	2	Nutrition Research Center, Ministry of Health (Mongolia) and Unicef -Mongolia. Nutritional status of Mongolian children and women: 3rd National nutrition survey report. Ulaanbaatar, Mongolia, 2006.
Mongolia	2005	0-59.99	National	3275	HAZ, WAZ	1	MICS
Mongolia	2010	0-59.99	National	3956	HAZ, WAZ	3	Multiple Indicator Cluster Survey 2010. Summary Report. Unlaanbaatar, 2011.
Morocco	1987	0-59.99	National	5289	HAZ, WAZ	1	DHS
Morocco	1992	0-59.99	National	4512	HAZ, WAZ	1	DHS
Morocco	1997	0-59.99	National	3587	HAZ, WAZ	1	PAPCHILD Morocco
Morocco	2003-2004	0-59.99	National	5315	HAZ, WAZ	1	DHS

Mozambique	1995	0-59.99	National	4586	HAZ, WAZ	3,5	Government of Mozambique and United Nations Children's Fund. Multiple indicator cluster survey Mozambique - 1995. MICS Surveys. Ministry of Planning and Finance. Maputo, Mozambique, 1996 (and additional analysis).
Mozambique	1997	0-35.99	National	3357	HAZ, WAZ	1	DHS
Mozambique	2000-2001	0-59.99	National	NR	HAZ, WAZ	3,5,7	Questionario de indicadores basicos de bem-estar (QUIBB): Relatorio final. Maputo, Moçambique: Instituto Nacional de Estadisticas, 2001 (and additional analysis).
Mozambique	2002	6-59.99	Regional	4832	HAZ	3,5	Mozambique Comite de Analise de Vulnerabilidade (VAC). Emergency vulnerability report: November-December 2002 (final report). Maputo, Mozambique: VAC, 2003.
Mozambique	2003-2004	0-59.99	National	7980	HAZ, WAZ	1	DHS
Mozambique	2008	0-59.99	National	10595	HAZ, WAZ	1	MICS
Myanmar	1990	0-35.99	National	5899	WAZ	3,5	Ministry of Health. Nutrition Situation of Myanmar Children. Preliminary report of the national nutrition survey 1990. Rangoon, Myanmar, 1991 (and additional analysis).
Myanmar	1991	0-35.99	National	5540	HAZ, WAZ	3,5	Ministry of Health. Nutrition situation of Myanmar children. Report of the National Nutrition Survey 1991. Ragoon, Myanmar, 1994 (and additional analysis).
Myanmar	1994	0-35.99	National	5994	HAZ, WAZ	3,5	Department of Health. National nutrition survey, 1994. National Nutrition Centre. Yangon, Myanmar, 1995 (and additional analysis).
Myanmar	1995	0-59.99	National	19908	WAZ	3,5	Ministry of Health. Monitoring progress toward the goals of the World Summit for Children through multiple indicator cluster survey (MICS). Yangon, Myanmar, 1995 (and additional analysis).
Myanmar	1997	0-35.99	National	4894	HAZ, WAZ	3,5	Ministry of Health. National nutrition survey 1997. National Nutrition Centre. Yangon, Myanmar, 2000 (and additional analysis).
Myanmar	2000	0-59.99	National	8140	HAZ, WAZ	1	MICS
Myanmar	2003	0-59.99	National	8451	HAZ, WAZ	2	Department of Health Planning, Ministry of Health and UNICEF (Myanmar). Multiple indicator cluster survey 2003 (MICS). Yangon, Myanmar, 2004 (and additional analysis).
Namibia	1992	0-59.99	National	2534	HAZ, WAZ	1	DHS
Namibia	2000	0-59.99	National	2913	HAZ, WAZ	1	DHS
Namibia	2006-2007	0-59.99	National	3668	HAZ, WAZ	1	DHS
Nepal	1995	6-35.99	National	6781	HAZ, WAZ	3,5	National Planning Commission. Nepal multiple indicator surveillance: cycle I, Jan to March 1995 health and nutrition - final report (MICS). Kathmandu, Nepal, 1996 (and additional analysis).
Nepal	1995-1996	6-35.99	National (rural)	696	HAZ, WAZ	2,5	Hotchkiss DR, Mock NB, Seiber EE. The effect of the health care supply environment on children's nutritional status in rural Nepal. Journal of biosocial Science 2002;34:173-92.
Nepal	1996	6-35.99	National	5525	HAZ, WAZ	2,5	National Planning Commission. Early childhood feeding nutrition and development. Nepal multiple indicator surveillance - fourth cycle (MICS). His Majesty's Government, Kathmandu, Nepal, 1997 (and additional analysis).
Nepal	1996	0-35.99	National	3700	HAZ, WAZ	1	DHS

Nepal	1997-1998	6-59.99	National	17471	HAZ, WAZ	2	Nepal micronutrient status survey 1998. Kathmandu, Nepal: Ministry of Health, Child Health Division, HMG/N, New ERA, Micronutrient Initiative, UNICEF Nepal and WHO, 2000 (and additional analysis).
Nepal	2001	0-59.99	National	6143	HAZ, WAZ	1	DHS
Nepal	2006	0-59.99	National	5206	HAZ, WAZ	1	DHS
Nepal	2011	0-59.99	National	2324	HAZ, WAZ	1	DHS
Nicaragua	1988	0-59.99	First admin level	2822	HAZ, WAZ	3,5	Ministerio de Salud, Centro de Investigacion y Estudios de la Salud. Enfoque de riesgo y estado nutricional de los ninos menores de 5 anos en la region III, 1988. Managua, Nicaragua; 1988.
Nicaragua	1993	0-59.99	National	3347	HAZ, WAZ	2	Nicaragua 1993 living standards measurement survey (LSMS). Washington, D.C.: The World Bank, 1997 (and additional analysis).
Nicaragua	1997-1998	0-59.99	National	6793	HAZ, WAZ	1	DHS
Nicaragua	1998	0-59.99	National	2787	HAZ, WAZ	2	Instituto Nacional de Estadisticas y Censos [Nicaragua] and the World Bank. Encuesta nacional de hogares sobre medicion de niveles de vida, 1998 (2nd Living Standard Measurements Survey). Managua, Nicaragua, 1999 (and additional analysis).
Nicaragua	2001	0-59.99	National	5839	HAZ, WAZ	1	DHS
Nicaragua	2003-2005	6-59.99	National	1494	HAZ, WAZ	2	Ministra de Salud, MINSA y CDC. Sistema integrado de vigilancia de interventiones nutricionales (SIVIN): Informe de progreso, Nicaragua 2003-05. Managua, Nicaragua 2008 (and additional analysis).
Nicaragua	2006-2007	0-59.99	National	6538	HAZ, WAZ	1	RHS Nicaragua 2006-2007
Niger	1985	0-59.99	National	1672	HAZ, WAZ	3,5	Ministère de la Santé Publique et des Affaires Sociales. Enquête nationale sur la morbidité et la mortalité, rapport No 1. Cellule de Planification. Niamey, République de Niger, 1985 (and additional analysis).
Niger	1987	0-59.99	Regional	3115	HAZ	3,5	Quelin G, Pecoul B, Amadou B, Baker S. [Facteurs de risque de la malnutrition chez les enfants de 0-59 mois dans deux arrondissements au Niger.] Medicine Tropicale 1991;51:335-342.
Niger	1992	0-59.99	National	4643	HAZ, WAZ	1	DHS
Niger	1998	0-35.99	National	3870	HAZ, WAZ	1	DHS
Niger	2000	0-59.99	National	4768	HAZ, WAZ	1	MICS
Niger	2006	0-59.99	National	3671	HAZ, WAZ	1	DHS
Nigeria	1986	6-35.99	First admin level	1464	HAZ, WAZ	2,5	Ministry of Health. Ondo State, Nigeria, demographic and health survey 1986. Demographic and Health Surveys. Medical/Preventive Health Division, Akure, Ondo State, Nigeria, 1989 (and additional analysis).
Nigeria	1990	0-59.99	National	5750	HAZ, WAZ	1	DHS
Nigeria	1999	0-59.99	National	8617	HAZ, WAZ	3,5	Federal Office of Statistics [Nigeria] and UNICEF. Multiple indicator cluster survey (1999) Nigera (MICS). Lagos: Federal Office of Statistics and UNICEF, December 14, 2000.
Nigeria	2001	0-59.99	National	4954	HAZ, WAZ	3,5	Maziya-Dixon B, Akinyele IO, Oguntona EB, Nokoe S, Sanusi RA, Harris E. Nigeria food consumption survey 2001-2003: Summary. Ibadan, Nigeria: International Institute of Tropical Agriculture, 2004.
Nigeria	2003	0-59.99	National	4349	HAZ, WAZ	1	DHS
Nigeria	2007	0-59.99	National	14423	HAZ, WAZ	1	MICS
Nigeria	2008	0-59.99	National	18712	HAZ, WAZ	1	DHS

Occupied Palestinian Territory	1995	0-59.99	First admin level	1500	HAZ, WAZ	3,5	Kumar B. Assessment of the nutritional status of children under 5 in the Gaza strip. Bethlehem: Terre des Hommes Palestine, 1995.
Occupied Palestinian Territory	1996	0-59.99	National	4451	HAZ, WAZ	3,5	The health survey in the West Bank and Gaza Strip: main findings (MICS). Palestinian Central Bureau of Statistics. Ramallah, Palestine, 1996 (and additional analysis).
Occupied Palestinian Territory	2006-2007	0-59.99	National	9364	HAZ, WAZ	2	Palestinian Central Bureau of Statistics. Palestinian family health survey, 2006: Final report. Ramallah, Palestine, 2007 (additional analysis conducted by PAPFAM).
Oman	1991	12-59.99	National	764	HAZ, WAZ	3,5	Musaiger OA. Ministry of Health. National Nutrition Survey of the Sultanate of Oman. UNICEF Muscat, Oman, 1993 (and additional analysis).
Oman	1994-1995	0-47.99	National	639	HAZ, WAZ	2	Ministry of Health. National study on the prevalence of vitamin A deficiency (VAD) among children 6 months to 7 years. Muscat, Sultanate of Oman, 1995 (and additional analysis).
Oman	1999	0-59.99	National	14076	HAZ, WAZ	2	Alasfoor D, Mohammed AJ. Implications of the use of the new WHO growth charts on the interpretation of malnutrition and obesity in infants and young children in Oman. Eastern Mediterranean Health Journal 2009;15:890-8 (and additional analysis).
Oman	2009	0-59.99	National	8105	HAZ, WAZ	2	Alasfoor D, Al Sayed M, AlShamakhi S, Al Ghammari I. Second national PEM survey 2009. Ministry of Health. Muscat, Oman, 2011.
Pakistan	1985-1987	6-59.99	National	6707	HAZ, WAZ	3,5	Government of Pakistan. National nutrition survey 1985-87 report. National Institute of Health, Nutrition Division. Islamabad, Pakistan, 1988 (and additional analysis).
Pakistan	1990-1991	0-59.99	National	4152	HAZ, WAZ	1	DHS
Pakistan	1990-1994	0-59.99	National	3240	HAZ, WAZ	2	Nuruddin R and Azam I. National health survey of Pakistan (NHSP, 1990-94): Health profile of the people of Pakistan. Islamabad, Pakistan, 1998 (and additional analysis).
Pakistan	1995	0-59.99	National	7368	WAZ	3,5	Ministry of Health. Multiple indicator cluster survey of Pakistan, 1995. MICS surveys. Government of Pakistan. Islamabad, Pakistan, 1996 (and additional analysis).
Pakistan	1998	6-35.99	First admin level	4932	HAZ, WAZ	3,5	CIETinternational, Sindh Bureau of Statistics, Government of Sindh, UNICEF Sindh. The bond of care: Technical Report, Sindh Province 1998. Karachi, Sindh Province: CIETinternational, 1999.
Pakistan	2001	6-59.99	National	9174	HAZ, WAZ	1	Pakistan National Nutrition Survey 2001
Pakistan	2011	0-59.99	National	28087	HAZ, WAZ	1	Pakistan Nutritional Survey 2011
Panama	1992	12-59.99	National	1389	HAZ, WAZ	3,5	Ministerio de Salud. Encuesta nacional de Vitamina A, 1992. Departamento de Nutricion y Dietetica. Panama, Republica de Panama, 1992.
Panama	1997	0-59.99	National	2289	HAZ, WAZ	2	Ministerio de Planificacion y Politica Economica. Encuesta de niveles de vida. 1997 Panama living standards survey (LSMS). Ciudad de Panama, Republica de Panama, 1998 (and additional analysis).

Papua New Guinea	1986-1987	0-59.99	National (urban)	530	HAZ, WAZ	3,5	Jenkins C, Zemel B. Ancient diversity and contemporary change in the growth patterns of Papua New Guinea children. 59th Annual Meeting of the American Association of Physical Anthropologists. Miami, Florida, 1990.
Papua New Guinea	2005	6-59.99	National	924	HAZ, WAZ	2	Department of Health of Papua New Guinea, UNICEF Papua New Guinea, University of Papua New Guinea, US Centers of Disease Control and Prevention. Papua New Guinea national micronutrient survey 2005: Final report. Port Moresby, Papua New Guinea, 2009 (and additional analysis).
Paraguay	1990	0-59.99	National	3605	HAZ, WAZ	1	DHS
Paraguay	2005	0-59.99	National	1700	HAZ, WAZ	2	Sanabria MC. Informe final de consultoria: Analisis de la situacion de salud infantil y antropometria en menores de 5 años. Paraguay EPH 2005. Asuncion, Paraguay: PNUD Paraguay, 2006.
Peru	1991-1992	0-59.99	National	7653	HAZ, WAZ	1	DHS
Peru	1994	0-59.99	National	2070	HAZ, WAZ	1	ENNIV 1994
Peru	1996	0-59.99	National	14781	HAZ, WAZ	1	DHS
Peru	2000	0-59.99	National	1835	HAZ, WAZ	1	ENNIV 2000
Peru	2000	0-59.99	National	11515	HAZ, WAZ	1	DHS
Peru	2003-2005	0-59.99	National	2290	HAZ, WAZ	1	DHS
Peru	2006-2008	0-59.99	National	8075	HAZ, WAZ	1	DHS
Peru	2009	0-59.99	National	9183	HAZ, WAZ	3	Instituto Nacional de Estadística e Informática (INEI), Agencia de los Estados Unidos Para el Desarrollo Internacional (USAID) y ORC Macro. Encuesta demografica y de salud familiar. Informe principal: ENDES continua 2009. Demographic and Health Surveys. Lima, Peru: INEI, USAID y ORC Macro, 2009.
Peru	2010	0-59.99	National	8668	HAZ, WAZ	3	Instituto Nacional de Estadística e Informática (INEI), Agencia de los Estados Unidos Para el Desarrollo Internacional (USAID) y ORC Macro. Encuesta demografica y de salud familiar. Informe principal: ENDES continua 2010. Demographic and Health Surveys. Lima, Peru: INEI, USAID y ORC Macro, 2009.
Philippines	1987	0-59.99	National	2250	HAZ, WAZ	3,5	Departement of Science and Technology. Third National Nutrition Survey Philippines, 1987. Food and Nutrition Research Institute. Manila, Philippines; 1991 (and additional analysis).
Philippines	1989-1990	0-59.99	National	5629	HAZ, WAZ	3,5	National Economics and Statistics Section. Regional Updating of Nutritional Status of Filipino Children, 1989-90. Food and Nutrition Research Institute. Manila, Philippines; 1991 (and additional analysis).
Philippines	1992	0-59.99	National	5858	HAZ, WAZ	3,5	Department of Science and Technology. The 1992 regional nutrition survey. Food and Nutrition Research Institute. Manila, Philippines; 1994 (and additional analysis).
Philippines	1993	0-59.99	National	4229	HAZ, WAZ	3,5	Department of Science and Technology. The fourth national nutrition survey: Philippines 1993. Food and Nutrition Institute. Manila, Philippines; 1995 (and additional analysis).
Philippines	1998	0-59.99	National	24308	HAZ, WAZ	3,5	Food and Nutrition Research Institute. The 5th national nutrition survey. Philippine nutrition: Facts & figures. Taguig, Metro Manila, Philipines: UNICEF, 2001 (and additional analysis).

Philippines	2003	0-59.99	National	3499	HAZ, WAZ	2	Sixth National Nutrition Survey: Philippines, 2003. Food and Nutrition Research Institute, 2004.
Philippines	2008	0-59.99	National	18403	HAZ, WAZ	3	Food and Nutrition Research Institute, Department of Science and Technology (FNRI-DOST). 7th national nutrition survey. Manila, Philippines: FNRI-DOST, 2010 (and additional analysis).
Rwanda	1991-1992	0-59.99	National (rural)	1939	HAZ, WAZ	2,5	Ministère de l'Agriculture et de l'Elevage. Statut nutritionnel et sécurité alimentaire au Rwanda: résultats de l'enquête nationale sur la nutrition et la sécurité alimentaire des enfants de 0 à 5 ans et leurs mères. Division des Statistiques Agricoles. Kigali, République Rwandaise, 1992.
Rwanda	1992	0-59.99	National	4280	HAZ, WAZ	1	DHS
Rwanda	1992	0-59.99	National (rural)	1639	HAZ, WAZ	2,5	Ministère de l'Agriculture et de l'Elévage. Statut nutritionnel et sécurité alimentaire au Rwanda: résultats de l'enquête nationale sur la nutrition et la sécurité alimentaire des enfants de 0 à 5 ans et leurs mères. Division des Statistiques Agricoles. Kigali, République Rwandaise; Juin 1993.
Rwanda	1996	0-59.99	National (rural)	1115	HAZ, WAZ	3,5	Ministère de la Santé. National nutrition survey of women and children in Rwanda in 1996. Kigali, République Rwandaise, 1997 (and additional analysis).
Rwanda	2000	0-59.99	National	6014	HAZ, WAZ	1	DHS
Rwanda	2000	0-59.99	National	2700	HAZ, WAZ	1	MICS
Rwanda	2005	0-59.99	National	3614	HAZ, WAZ	1	DHS
Rwanda	2010	0-59.99	National	4050	HAZ, WAZ	1	DHS
Samoa	1999	0-59.99	National	1107	HAZ, WAZ	3,5	Mackerras D, Kiernan DM. Samoa national nutrition survey 1999. Part 3: Child growth, diet, contact with the health system and interview with carers. Technical report. Apia, Samoa: Nutrition Centre, 2003 (and additional analysis).
Saudi Arabia	1994	0-59.99	National	23821	HAZ, WAZ	3,5	Al-Mazrou YY, Al-Amoud MM, El-Gizouli SE, Khoja T, Al-Turki K, Tantawi N, Khalil MK, Aziz KM. Comparison of the growth standards between Saudi and American children aged 0-5 years. Saudi Medical Journal 2003;24:598-602 [Erratum Saudi Medical Journal 2003;24:1032] (and additional analysis).
Saudi Arabia	2004-2005	0-59.99	National	15601	HAZ, WAZ	2	El-Mouzan MI, Al-Herbish AS, Al-Salloum AA, Qurachi MM, Al-Omar AA. Growth charts for Saudi children and adolescents. Saudi Medical Journal 2007;28:1555-68 (and additional analysis).
Senegal	1986	6-35.99	National	637	HAZ, WAZ	1	DHS
Senegal	1991-1992	0-35.99	National	NR	HAZ, WAZ	3,5,7	Senegal Bureau of Statistics. Social Dimensions of Adjustment Household Priority Survey 1991-92. New York: The World Bank, 1993 (and additional analysis).
Senegal	1992-1993	0-59.99	National	4401	HAZ, WAZ	1	DHS
Senegal	1996	0-59.99	National	NR	HAZ, WAZ	3,5,7	Evaluation des objectives intermédiares (MICS). Dakar: UNICEF, September 1996 (and additional analysis).
Senegal	2000	0-59.99	National	8434	HAZ, WAZ	1	MICS
Senegal	2005	0-59.99	National	2860	HAZ, WAZ	1	DHS
Senegal	2010-2011	0-59.99	National	3701	HAZ, WAZ	1	DHS
Seychelles	1987-1988	0-59.99	National	836	HAZ, WAZ	3,5	Ministry of Health. Nutritional status of Seychellois children (unpublished data). Victoria, Seychelles, 1989 (and additional analysis).

Sierra Leone	1989	0-59.99	National	4424	HAZ, WAZ	3,5	Ministry of Health. The Republic of Sierra Leone National Nutrition Survey. Freetown, Sierra Leone; 1990.
Sierra Leone	1990	0-59.99	National	4595	HAZ, WAZ	3,5	Ministry of Health. The Republic of Sierra Leone National Nutrition Survey. Freetown, Sierra Leone; 1990.
Sierra Leone	2000	0-59.99	National	2224	HAZ, WAZ	1	MICS
Sierra Leone	2005	0-59.99	National	5103	HAZ, WAZ	2	Statistics Sierra Leone and UNICEF-Sierra Leone. Sierra Leone multiple indicator cluster survey 2005: Final Report. Freetown, Sierra Leone: Statistics Sierra Leone and UNICEF-Sierra Leone, 2007 (and additional analysis).
Sierra Leone	2008	0-59.99	National	1995	HAZ, WAZ	1	DHS
Solomon Islands	1989	0-59.99	National	3980	HAZ, WAZ	2	Solomon Islands national nutrition survey 1989. Honiara, Solomon Islands, 1990 (and additional analysis).
Solomon Islands	2006-2007	0-59.99	National	2029	HAZ, WAZ	3	National Statistics Office (SISO), SPC and Macro International Inc. Solomon Islands 2006-2007 demographic and health survey. DHS. Noumea, New Caledonia: SISO, SPC and Macro International Inc., 2007 (accessed 22/12/09 http://www.spc.int/prism/Country/SB/Stats/Publication/DHS07/report/SI-DHS-REPORT_TOC_Summary.pdf)
Somalia	2000	0-59.99	National	3582	HAZ, WAZ	3,5	UNICEF Somalia. Somalia end-decade multiple indicator cluster survey. Full technical report. Nairobi: UNICEF Somilia, 2001 (and additional analysis).
Somalia	2006	0-59.99	National	5513	HAZ, WAZ	1	MICS
South Africa	1986	0-59.99	National (rural)	1745	HAZ, WAZ	3,5	First RHOSA nutrition survey, anthropometric assessment of nutritional status in black under-fives in rural R.S.A. Epidemiological comments Vol 14 No 3. Pretoria: Department of National Health and Population Development, 1987.
South Africa	1993-1994	6-59.99	National	3689	HAZ	2,5	South Africans rich and poor: baseline household statistics. Project for statistics on living standards and development. Cape Town, 1994 (and additional analysis).
South Africa	1994-1995	6-59.99	National	9807	HAZ, WAZ	3,5	The South African Vitamin A Consultative Group. Children aged 6 to 71 months in South Africa, 1994: their anthropometric, vitamin A, iron and immunisation coverage status. Johannesburg, South Africa, 1995 (and additional analysis).
South Africa	1999	12-59.99	National	1556	HAZ, WAZ	3,5	Labadarios D, Steyn NP, Maunder E, MacIntryre U, Gericke G, Swart R, et al. The National Food Consumption Survey (NFCS): South Africa, 1999. Public Health Nutrition 2005;8:533-43 (and additional analysis).
South Africa	2003-2004	0-59.99	National	1310	HAZ, WAZ	1	DHS
South Africa	2005	12-35.99	National	846	HAZ, WAZ	3,5	Department of Health, Republic of South Africa. National Food Consumption Survey - Fortification Baseline: Stellenbosch, South Africa: Directorate: Nutrition, Department of Health. 2005
South Africa	2008	0-59.99	National	2079	HAZ, WAZ	3	Ardington C, Case A. National income dynamics study (NIDS). Health: Analysis of the NIDS wave 1 dataset. Discussion paper no. 2. South Africa: The Presidency Republic of South Africa and SALDRU, 2009. http://www.nids.uct.ac.za/home/ accessed 15 April 2011.
Sri Lanka	1987	3-35.99	National	1999	HAZ, WAZ	1	DHS

Sri Lanka	1993	3-59.99	National	3068	HAZ, WAZ	3,5	Department of Census and Statistics, Ministry of Finance, Planning, Ethnic Affairs and National Inte Sri Lanka demographic and health survey 1993. Colombo, Sri Lanka, 1995.
Sri Lanka	1995	3-59.99	National	2782	HAZ, WAZ	3,5	Ramanujam P and Nestel P. Preliminary report on the fourth national nutrition and health survey July - August, 1995. The Ceylon Journal of Medical Science 1997;40:13-24.
Sri Lanka	1995-1996	6-59.99	National	2304	HAZ, WAZ	3,5	Medical Research Institute of the Ministry of Health and Indigenous Medicine. Vitamin A deficiency status of children in Sri Lanka, 1995/1996: A survey report. Colombo, Sri Lanka, 1998.
Sri Lanka	2000	3-59.99	National	2512	HAZ, WAZ	2	Department of Census and Statistics and Ministry of Health, Nutrition and Welfare. Sri Lanka demographic and health survey 2000. Colombo, Sri Lanka, 2001 (and additional analysis).
Sri Lanka	2001	6-59.99	National	1716	HAZ, WAZ	3,5	Medical Research Institute of the Department of Health Services, Ministry of Health, Nutrition and Welfare. Assessment of anaemia status in Sri Lanka, 2001: A survey report. Colombo, Sri Lanka, 2003.
Sri Lanka	2006-2007	0-59.99	National	6649	HAZ, WAZ	3	Department of Census and Statistics, Ministry of Health Care and Nutrition. Sri Lanka demographic and health survey 2006/07: Preliminary report and selected tables from the chapters of the final report (I:\UnitData\SURVEILLANCE\GDCGM\data sets\Sri Lanka\DHS_MoH 2006_07_WHO\DHS_web_links.htm, accessed 03/07/09).
Sri Lanka	2009	0-59.99	National	2589	HAZ, WAZ	2	Jayatissa R. Nutrition and food security survey 2009. Colombo, Sri Lanka: Medical Research Institute, 2010.
Sudan	1993	0-59.99	Regional	3418	HAZ, WAZ	1	PAPCHILD Sudan
Sudan	1995	0-59.99	Regional	3454	HAZ, WAZ	2	Federal Ministry of Health, WHO, Ministries of Health/Nutrition Departments of 6 states. Comprehensive nutrition survey. National Nutrition Department. Khartoum, Sudan, 1997 (and additional analysis).
Sudan	2000	0-59.99	Regional	18234	HAZ, WAZ	1	SudanNorthMICS2
Sudan	2000	1-59.99	Regional (urban)	877	HAZ, WAZ	1	MICS
Sudan	2006	0-59.99	National	19801	HAZ, WAZ	2	Government of National Unity and Government of Southern Sudan. Sudan household health survey (SHHS) - 2006. Khartoum and Juba: Government of National Unity, Government of Southern Sudan, December 2007 (additional analysis conducted by PAPFAM, June 2009).
Sudan	2010	0-59.99	National	NR	HAZ, WAZ	3,7	National Ministry of Health and Central Bureau of Statistics, Sudan Sudan Household Health Survey Second Round 2010 Summary Report, August 2011
Suriname	2000	0-59.99	National	1411	HAZ, WAZ	1	MICS
Suriname	2006	0-59.99	National	1992	HAZ, WAZ	2	General Bureau of Statistics, Ministry of Planning and Development Cooperation and Ministry of Social Affairs and Housing. Suriname multiple indicator cluster survey 2006, Final Report (MICS3). Paramaribo, Suriname, 2009 (and additional analysis).
Swaziland	2000	0-59.99	National	3200	HAZ, WAZ	1	MICS
Swaziland	2006-2007	0-59.99	National	2027	HAZ, WAZ	1	DHS

Swaziland	2008	0-59.99	National	3823	HAZ, WAZ	3	Ministry of Health, National Nutrition Council. Swaziland national nutrition survey report (November 2008). Mbabane, Swaziland: Ministry of Health, 2009.
Swaziland	2010	0-59.99	National	2555	HAZ, WAZ	2	Central Statistical Office, Kingdom of Swaziland Multiple Indicator Cluster Survey 2010 Preliminary Report, Central Statistical Office, Kingdom of Swaziland
Syrian Arab Republic	1993	0-59.99	National	3959	HAZ, WAZ	1	PAPCHILD Syrian Arab Republic
Syrian Arab Republic	1995	0-59.99	National	2425	HAZ, WAZ	3,5	Prime Minister's Council. Multiple indicator cluster survey in the Syrian Arab Republic (MICS). Central Bureau of Statistics. Damascus, The Syrian Arab Republic, 1996.
Syrian Arab Republic	2000	0-59.99	National	6262	HAZ, WAZ	3,5	Council of Ministers, Central Office of Statistics, Arab Republic of Syria, and UNICEF [Multiple Indicator Cluster Survey II (MICS II) concerning Child Health and Welfare. Main report.] Damascus, Syrian Arab Republic: UNICEF, 2002 (and additional analysis).
Syrian Arab Republic	2001	0-59.99	National	6367	HAZ, WAZ	2	League of Arab States (PAPFAM) and Syrian Arab Republic, Office of the Prime Minister, Central Bureau of Statistics. The family health survey in the Syrian Arab Republic. Principal Report. Cairo: The League of Arab States, 2002 (and additional analysis).
Syrian Arab Republic	2006	0-59.99	National	9906	HAZ, WAZ	1	MICS
Syrian Arab Republic	2009	0-59.99	National	14392	HAZ, WAZ	2	League of Arab States and Syrian Arab Republic. Family health survey of the Arab Republic of Syria 2009: Principal report (PAPFAM). Cairo: The League of Arab States, 2011 (and additional analysis).
São Tomé and Príncipe	1986	0-59.99	National	2155	HAZ, WAZ	3,5	Ministerio de Saude. Estado nutricional e cobertura vacinal des criancas menoras de 5 anos na. Seccao de Nutricao. Sao Tome, Republica Democratica de Sao Tome e Principe, 1986 (and additional analysis).
São Tomé and Príncipe	2000	0-59.99	National	1677	HAZ, WAZ	1	MICS
São Tomé and Príncipe	2006	0-59.99	National	2848	HAZ, WAZ	3,5	Institute of National Statistics (INE). Democratic Republic of Sao Tome e Principe 2006 multiple indicator cluster survey (MICS3): Final report. Sao Tome: INE and UNICEF, 2007.
São Tomé and Príncipe	2008-2009	0-59.99	National	1430	HAZ, WAZ	1	DHS
Tajikistan	1999	6-59.99	National	3599	HAZ	3,5	McBurney R and Mason F. National nutrition survey, Tajikistan (September/October 1999). London, UK: Action Against Hunger UK, 2000 (and additional analysis).
Tajikistan	2000	6-59.99	National	5657	HAZ	3,5	McLachlan E. National nutrition survey of Tajikistan (September/October 2000). London, UK: Action Against Hunger UK, 2001 (and additional analysis).
Tajikistan	2001	6-59.99	National	3704	HAZ	3,5	Walters T, Brown R. Representative national nutrition survey Tajikistan (Sughd, RRS, Kouliab and Kurgan Teppe regions), October/November 2001. London, UK: Action Against Hunger UK, 2002 (and additional analysis).

Tajikistan	2002	6-59.99	National	4543	HAZ	3,5	Moloney G and Brown R. National nutrition survey Tajikistan, May/June 2002. Action against Hunger and European Community Humanitarian Office, 2002 (and additional analysis).
Tajikistan	2003	6-59.99	National	4654	HAZ	3,5	Baronina E, Brasell-Jones T, Petersen J, Purves M. National nutrition and water & sanitation survey, Tajikistan, October 2003. Dushanbe, Tajikistan: Action Against Hunger (lead agency nutrition) and Mercy Corps (lead agency wat/san), 2004 (and additional analysis).
Tajikistan	2005	0-59.99	National	4065	HAZ, WAZ	1	MICS
Tajikistan	2007	0-59.99	National	2544	HAZ, WAZ	3	State Committee on Statistics [Republic of Tajikistan] and UNICEF. Tajikistan living standards measurement survey 2007 (TLSS): Indicators at a glance. Dushanbe, Republic of Tajikistan: State Committee on Statistics and UNICEF, 2009 (www.tojikinfo.tj/en/.../UNICEF%20TLSS%20Report%20Eng.pdf, accessed 09/06/2011).
Thailand	1987	3-35.99	National	1848	HAZ, WAZ	1	DHS
Thailand	1993	0-59.99	National	11748	HAZ, WAZ	3,5	Kitvorapat W, Chaotilittakul N, Sinawat S, Wanaratana L. Random survey on nutritional status of children of ages under five. Thailand Journal of Health Promotion and Environmental Health 1996;19:57-66 (and additional analysis).
Thailand	1995	0-59.99	National	4178	HAZ, WAZ	3,5	Ministry of Public Health. The fourth national nutrition survey of Thailand 1995. Department of Health. Bangkok, Thailand 1998 (and additional analysis).
Thailand	1997	12-59.99	National	2599	HAZ, WAZ	1	NHES 2
Thailand	2005-2006	0-59.99	National	9002	HAZ, WAZ	1	MICS
Timor-Leste	2002	0-59.99	National	4133	HAZ, WAZ	2	Multiple indicator cluster survey (MICS - 2002). UNICEF, Dili, Timor-Leste, 2003 (and additional analysis).
Timor-Leste	2003	0-59.99	National	5255	HAZ, WAZ	3,5	Ministry of Health (MOH) Timor Leste, University of Newcastle, Australian National University and ACIL. Timor Leste 2003 demographic and health survey. Newcastle, NSW, Australia: MOH and University of Newcastle, 2003 (and additional analysis).
Timor-Leste	2009-2010	0-59.99	Regional	6975	HAZ, WAZ	1,6	DHS
Togo	1988	6-59.99	National	2866	HAZ, WAZ	3,5	Ministère de la Santé Publique, Ministère du Plan et des Mines, Ministère du Développement Rural. Politique nationale d'alimentation et de nutrition. Document de synthese. Lomé, Republique Togolaise, 1989 (and additional analysis).
Togo	1988	0-35.99	National	1656	HAZ, WAZ	1	DHS
Togo	1996	0-59.99	National	3761	HAZ, WAZ	3,5	Enquête national sur la situation des enfants au Togo en 1995 (MICS -Togo -96). Lomé, République Togolaise: Ministère du Plan et de l'Amenagement du Territoire et UNICEF, September 1996.
Togo	1998	0-35.99	National	3614	HAZ, WAZ	1	DHS
Togo	2006	0-59.99	National	3581	HAZ, WAZ	2	Direction Generale de la Statistique et de la Comptabilité Nationale and UNICEF. Résultats de l'enquête nationale à indicateurs multiples, Togo 2006. Rapport final, aôut 2007. http://www.childinfo.org/mics3_surveys.html, accessed 8 January 2009 (and additional analysis).

Togo	2008	0-59.99	National	3204	HAZ, WAZ	3	Ministère de la Santé et UNICEF. Rapport d'enquête nationale nutrition et survie des enfants de 0 à 59 mois, pratique d'alimentation de nourrisson et du jeune enfants. SMART. Togo, décembre 2008 (30/12/09 http://ochaonline.un.org/CoordinationIASC/Securitealimentairenutrition/tabid/5651/language/fr-FR/Default.aspx).
Togo	2010	0-59.99	National	4752	HAZ, WAZ	3	Direction Generale de la Statistique et de la Comptabilite Nationale, Togo Togo. Enquete par grappes a indicateurs multiple (MICS) 2010: Resultats preliminaires, Mai 2011, Direction Generale de la Statistique et de la Comptabilite Nationale
Tonga	1986	0-59.99	National	1094	HAZ	3,5	Maclean E, Badcock J, Bach F. The 1986 national nutrition survey of the Kingdom of Tonga. Technical report National Food and Nutrition Committee. Nuku'alofa: Government of the Kingdom of Tonga, 1987 (and additional analysis).
Trinidad and Tobago	1987	3-35.99	National	838	HAZ, WAZ	1	DHS
Trinidad and Tobago	2000	0-59.99	National	780	HAZ, WAZ	2	Multiple indicator cluster survey Trinidad and Tobago: Full report. UNICEF website http://www.childinfo.org/MICS2/newreports/trinidad/trinidad.htm, accessed 17/11/2003 (and additional analysis).
Tunisia	1988	3-35.99	National	2007	HAZ, WAZ	1	DHS
Tunisia	1993-1994	0-59.99	National	3030	HAZ, WAZ	1	PAPCHILD Tunisia
Tunisia	1996-1997	0-59.99	National	891	HAZ, WAZ	3,5	Ministère de la Santé Publique, Institut National de Nutrition et de Technologie Alimentaire. Enquête nationale 1996-1997. Evaluation de l'état nutritionnel de la population Tunisienne: Rapport national. Tunis, Tunisia: Sotepa Grafic, 1998 (and additional analysis).
Tunisia	2000	0-59.99	National	10310	HAZ, WAZ	3,5	Tunisia multiple indicator cluster survey II 2000 (MICS II). Tunis, Tunisia: 2000 (and additional analysis).
Tunisia	2006	0-59.99	National	2843	HAZ, WAZ	2	Ministère de la Santé Publique, Office National de la Famille et de la Population et UNICEF. Enquête sur la santé et le bien être de la mère et l'enfant: MICS 3. Tunis, Tunisia, 2008 (and additional analysis conducted by PAPFAM).
Turkey	1993	0-59.99	National	3134	HAZ, WAZ	1	DHS
Turkey	1995	0-59.99	National	2871	WAZ	3,5	Ministry of Health. Multiple indicator cluster survey in Turkey 1995. Ankara: National Bureau of Statistics, 1996 (and additional analysis).
Turkey	1998	0-59.99	National	2774	HAZ, WAZ	1	DHS
Turkey	2003-2004	0-59.99	National	4005	HAZ, WAZ	1	DHS
Turkmenistan	2000	0-59.99	National	2928	HAZ, WAZ	3,5	Gurbansoltan Eje Clinical Research Center for MCH (GECRCMCH), Ministry of Health & Medical Industry. Turkmenistan demographic and health survey 2000. Demographic and Health Surveys. Calverton, Maryland, USA: GECRCMCH and ORC Macro, 2001(and additional analysis).
Uganda	1988-1989	0-59.99	National	3649	HAZ, WAZ	1	DHS
Uganda	1995	0-47.99	National	4540	HAZ, WAZ	1	DHS
Uganda	2000-2001	0-59.99	National	5114	HAZ, WAZ	1	DHS
Uganda	2006	0-59.99	National	2354	HAZ, WAZ	1	DHS

United Republic of Tanzania	1991	0-59.99	First admin level	3446	HAZ, WAZ	3,5	Mpanju WFK, Msamanga GI, Gerverdinck IHA, Kabalimu TK, Kawau FMN, Rongo LMB, et al. Assessment of nutritional status and associated factors of under-fives in Dar es Salaam region from 9-23 September 1991. Institute of Public Health, Muhimbili University College of Health Sciences. Dar es Salaam, Tanzania; 1992.
United Republic of Tanzania	1991-1992	0-59.99	National	6311	HAZ, WAZ	1	DHS
United Republic of Tanzania	1996	0-59.99	First admin level	1654	HAZ, WAZ	3,5	Monitoring progress towards the goals of the world summit for children through multiple indicator cluster survey, 1996 (MICS). Bureau of Statistics. Dar es Salaam, United Republic of Tanzania, 1996.
United Republic of Tanzania	1996	0-59.99	National	5298	HAZ, WAZ	1	DHS
United Republic of Tanzania	1996	0-59.99	Regional	3939	HAZ, WAZ	3,5	Monitoring progress towards the goals of the world summit for children through multiple indicator cluster survey, 1996 (MICS). Bureau of Statistics. Dar es Salaam, United Republic of Tanzania, 1996.
United Republic of Tanzania	1999	0-59.99	National	2510	HAZ, WAZ	1	DHS
United Republic of Tanzania	2004-2005	0-59.99	National	7114	HAZ, WAZ	1	DHS
United Republic of Tanzania	2009-2010	0-59.99	National	6740	HAZ, WAZ	1	DHS
Uzbekistan	1996	0-35.99	National	935	HAZ, WAZ	1	DHS
Uzbekistan	2002	0-59.99	National	2525	HAZ, WAZ	2	Analytical and Information Center, Ministry of Health of the Republic of Uzbekistan, State Department of Statistics, Ministry of Macroeconomics and Statistics [Uzbekistan], and ORC Macro. Uzbekistan health examination survey 2002. Demographic and Health Surveys. Calverton, Maryland, USA: Analytical and Information Center, State Department of Statistics, and ORC Macro, 2004.
Uzbekistan	2006	0-59.99	National	4704	HAZ, WAZ	1	MICS
Vanuatu	1996	0-59.99	National	1297	HAZ, WAZ	3,5	Department of Health [Vanuatu] and Australian Agency for International Development (AusAID). Report of the second national nutrition survey 1996. Government of the Republic of Vanuatu, Department of Health and AusAID, 1998 (and additional analysis).
Vanuatu	2007	0-59.99	National	1215	HAZ, WAZ	1	MICS
Venezuela (Bolivarian Republic of)	1987	0-59.99	National	18023	HAZ, WAZ	3,5	Proyecto Venezuela 1987. Caracas: Centro de estudios sobre crecimiento y desarrollo de la poblacion venezolana, 1995 (and additional analysis).
Viet Nam	1987-1989	0-59.99	National	7044	HAZ, WAZ	3,5	Ministry of Health. Report on re-analyzed data collected by the General Nutrition Survey 1987-89. Department of Planning. Hanoi, Viet Nam, 1991 (and additional analysis).
Viet Nam	1992-1993	0-59.99	National	2833	HAZ, WAZ	2	Viet Nam living standards survey 1992-93 (VNLSS). Washington, D.C.: The World Bank, 1998 (and additional analysis).

Viet Nam	1994	0-59.99	National	37764	HAZ, WAZ	3,5	Bloem MW, Gorstein J. Viet Nam: Xerophthalmia free; 1994 national Vitamin A deficiency and protein-energy malnutrition prevalence survey. Consultancy report 5-17 March 1995. National Institute of Nutrition. Hanoi, Viet Nam, 1995 (and additional analysis).
Viet Nam	1997-1998	0-59.99	National	2848	HAZ, WAZ	1	Vietnam Living Standards Survey, 1997-98
Viet Nam	1998	0-59.99	National	12919	HAZ, WAZ	3,5	Dibley MJ, Khoi HH, Khan NC, Tam NC, Tuyen LD, Do TT, Mai LB. National protein energy malnutrition survey, Viet Nam 1998. National Institute of Nutrition, Hanoi, Viet Nam and Centre for Clinical Epidemiology & Biostatistics, Newcastle, Australia, 1999 (and additional analysis).
Viet Nam	1999	0-59.99	National	1541	HAZ, WAZ	2	Tuyen le D. Annual national nutrition monitoring. Nutrition Surveillance Department. Hanoi, Vietnam: National Institute of Nutrition, 2009 (and additional analysis).
Viet Nam	1999	0-59.99	National	93469	HAZ, WAZ	3,5	Khoi HH, Khan NC, Tuyen LD, Ngu T, Xuan TT. 1999 Viet Nam - child nutrition situation. The national goal for child malnutrition control. Hanoi: Medical Publishing House, 2000 (and additional analysis).
Viet Nam	2000	0-59.99	National	2983	HAZ, WAZ	1	MICS
Viet Nam	2000	0-59.99	National	1579	HAZ, WAZ	2	Tuyen le D. Annual national nutrition monitoring. Nutrition Surveillance Department. Hanoi, Vietnam: National Institute of Nutrition, 2009 (and additional analysis).
Viet Nam	2000	0-59.99	National	94469	HAZ, WAZ	3,5	National Institute of Nutrition and General Statistical Office. 2000 - Vietnam child an mother nutrition situation. Hanoi: Medical Publishing House, 2001 (and additional analysis).
Viet Nam	2001-2002	0-59.99	National	11145	HAZ, WAZ	1	VNHS 2002
Viet Nam	2002	0-59.99	National	1531	HAZ, WAZ	2	Tuyen le D. Annual national nutrition monitoring. Nutrition Surveillance Department. Hanoi, Vietnam: National Institute of Nutrition, 2009 (and additional analysis).
Viet Nam	2003	0-59.99	National	1457	HAZ, WAZ	2	Tuyen le D. Annual national nutrition monitoring. Nutrition Surveillance Department. Hanoi, Vietnam: National Institute of Nutrition, 2009 (and additional analysis).
Viet Nam	2004	0-59.99	National	1499	HAZ, WAZ	2	Tuyen le D. Annual national nutrition monitoring. Nutrition Surveillance Department. Hanoi, Vietnam: National Institute of Nutrition, 2009 (and additional analysis).
Viet Nam	2005	0-59.99	National	1493	HAZ, WAZ	2	Tuyen le D. Annual national nutrition monitoring. Nutrition Surveillance Department. Hanoi, Vietnam: National Institute of Nutrition, 2009 (and additional analysis).
Viet Nam	2006	0-59.99	National	1532	HAZ, WAZ	2	Tuyen le D. Annual national nutrition monitoring. Nutrition Surveillance Department. Hanoi, Vietnam: National Institute of Nutrition, 2009 (and additional analysis).
Viet Nam	2006	0-59.99	National	2680	HAZ, WAZ	3	National household living standard survey 2006 (VHLSS2006). Results reported in Viet Nam multiple indicator cluster survey 2006 - MICS3. Ha Noi, Viet Nam, 2007 (accessed 3 July 2008 http://www.childinfo.org/files/MICS3_Vietnam_FinalReport_2006.pdf).

Viet Nam	2007	0-59.99	National	1507	HAZ, WAZ	2	Tuyen le D. Annual national nutrition monitoring. Nutrition Surveillance Department. Hanoi, Vietnam: National Institute of Nutrition, 2009 (and additional analysis).
Viet Nam	2008	0-59.99	National	1483	HAZ, WAZ	2	Tuyen le D. Annual national nutrition monitoring. Nutrition Surveillance Department. Hanoi, Vietnam: National Institute of Nutrition, 2009 (and additional analysis).
Yemen	1991	0-59.99	National	2520	HAZ, WAZ	1	PAPCHILD Yemen
Yemen	1996	0-59.99	National	3833	HAZ, WAZ	3,5	Yemen multiple indicator cluster survey (March 1996): Final results. Ministry of Planning and Development. Sanaa, Republic of Yemen, 1996 (and additional analysis).
Yemen	1997	0-59.99	National	10793	HAZ, WAZ	2	Yemen demographic and maternal and child health survey 1997. Demographic and Health Surveys. Central Statistical Organization. Sana'a, Yemen, 1998 (and additional analysis).
Yemen	2003	0-59.99	National	12364	HAZ, WAZ	2	The Republic of Yemen Ministry of Health & Population, Central Statistical Organization an League of Arab States. The Yemen family health survey: Principal report. Pan Arab Project for Family Health. Cairo, Egypt: The Republic of Yemen Ministry of Health & Population, Central Statistical Organization an League of Arab States, 2004 (and additional analysis).
Yemen	2005-2006	0-59.99	National	12704	HAZ, WAZ	1	Yemen Household Budget Survey 2005-2006
Zambia	1990	6-59.99	National (rural)	2000	HAZ, WAZ	3,5	Cogill B, Zaza M. Report of the Nutrition Module as Part of the Crop Forecasting Survey - Rural Zambia 1990. Ministry of Health. Lusaka, Zambia; 1990.
Zambia	1992	0-59.99	National	4888	HAZ, WAZ	1	DHS
Zambia	1995	0-59.99	National	NR	HAZ	3,5,7	Food Security, Health and Nutrition Information System, National Commission for Development Planning, Central Statistics Office. Zambia's children in 1995: Key results of a survey to monitor progress towards goals for children (MICS2). Lusaka, Zambia: Government of the Republic of the Zambia, 1997 (and additional analysis).
Zambia	1996-1997	0-59.99	National	5470	HAZ, WAZ	1	DHS
Zambia	2001-2002	0-59.99	National	5394	HAZ, WAZ	1	DHS
Zambia	2004-2005	0-59.99	National	6865	HAZ, WAZ	1	Living Conditions Monitoring Survey IV
Zambia	2007	0-59.99	National	5063	HAZ, WAZ	1	DHS
Zimbabwe	1988-1989	3-59.99	National	2448	HAZ, WAZ	1	DHS
Zimbabwe	1994	0-35.99	National	2081	HAZ, WAZ	1	DHS
Zimbabwe	1999	0-59.99	National	2610	HAZ, WAZ	1	DHS
Zimbabwe	2005-2006	0-59.99	National	3878	HAZ, WAZ	1	DHS
Zimbabwe	2010-2011	0-59.99	National	4276	HAZ, WAZ	1	DHS

*1=individual-level data; 2=means and prevalences; 3=only prevalences; 4=surveillance data; 5=estimate converted from NCHS reference to WHO standards; 6=data collection in Aileu province deemed unreliable, province excluded and study marked as regional; 7=sample size not reported, sample size assumed to be 400; 8=included in analysis because total sample size (including other age groups or regions) exceeds 400.

Appendix Figure 1. Urban-rural differences in prevalences of stunting and underweight (in percentage points). A negative number indicates higher prevalence in rural children than in urban children; a positive number indicates the opposite.

(a) urban−rural difference in stunting

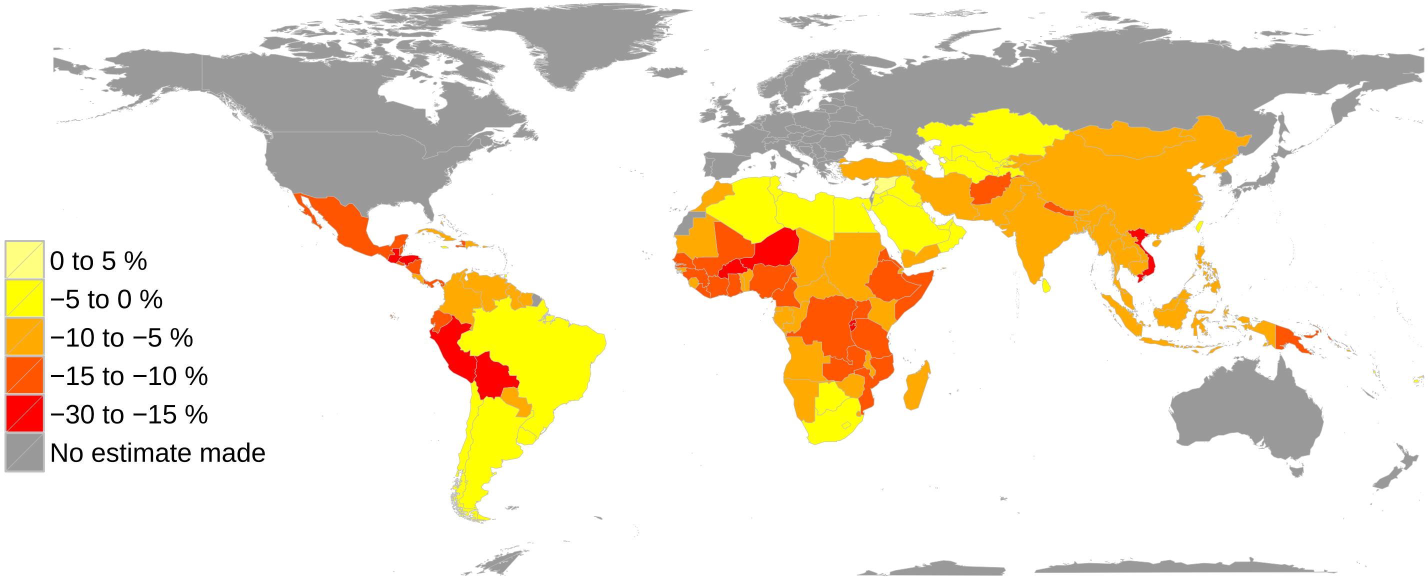

(b) urban−rural difference in underweight

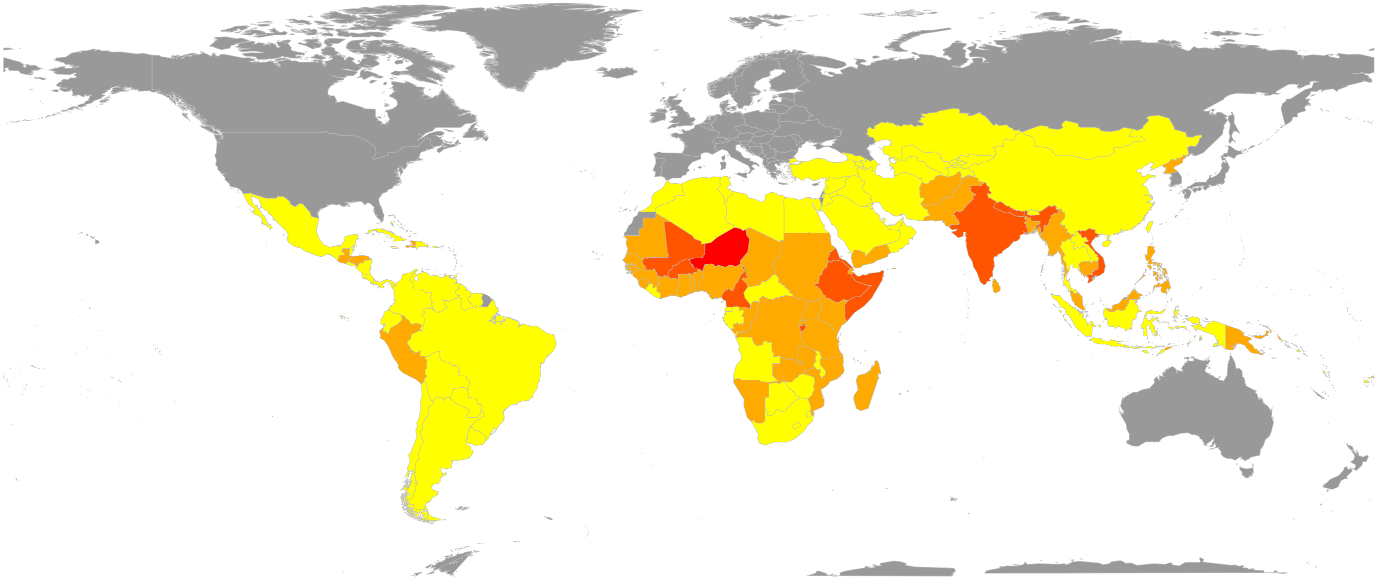

Appendix Figure 2. Trends in rural (blue) and urban (orange) prevalences of stunting and underweight by region.

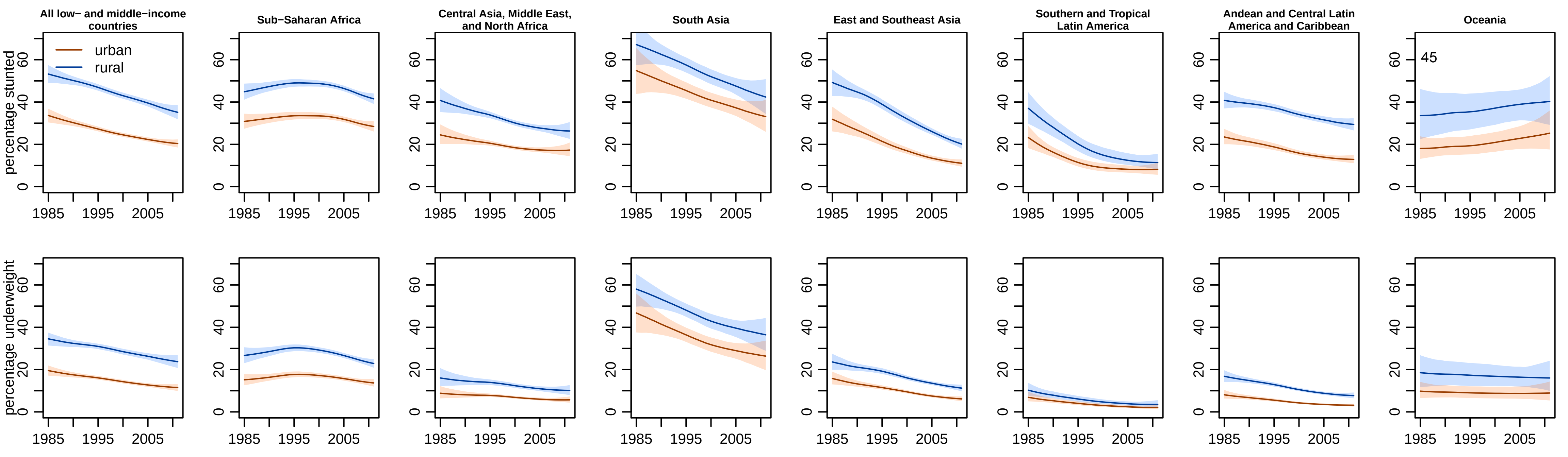

All low− and middle−income countries
Sub−Saharan Africa
Central Asia, Middle East, and North Africa
South Asia
East and Southeast Asia
Southern and Tropical Latin America
Andean and Central Latin America and Caribbean
Oceania
urban
rural
45
percentage stunted
percentage underweight
0
20
40
60
1985
1995
2005

Appendix Figure 3. Trends in rural and urban mean HAZ and WAZ and prevalences of stunting and underweight by country. Shaded areas show the 95% uncertainty interval of the trend. The points show original data, with the legend for symbols and colours shown on the first page.

Note: Some data sources did not report standard deviation or standard error. For these data sources, the figure shows confidence intervals calculated using the estimated standard deviation (or variance) from the model fit.

Nationally representative
Regional or first administrative unit

Rural
Urban
Mixed Rural/Urban

Covers defined age range
Does not cover defined age range

Uncertainty based on modelled variance
Beyond the y−axis limits

Afghanistan

South Asia Region

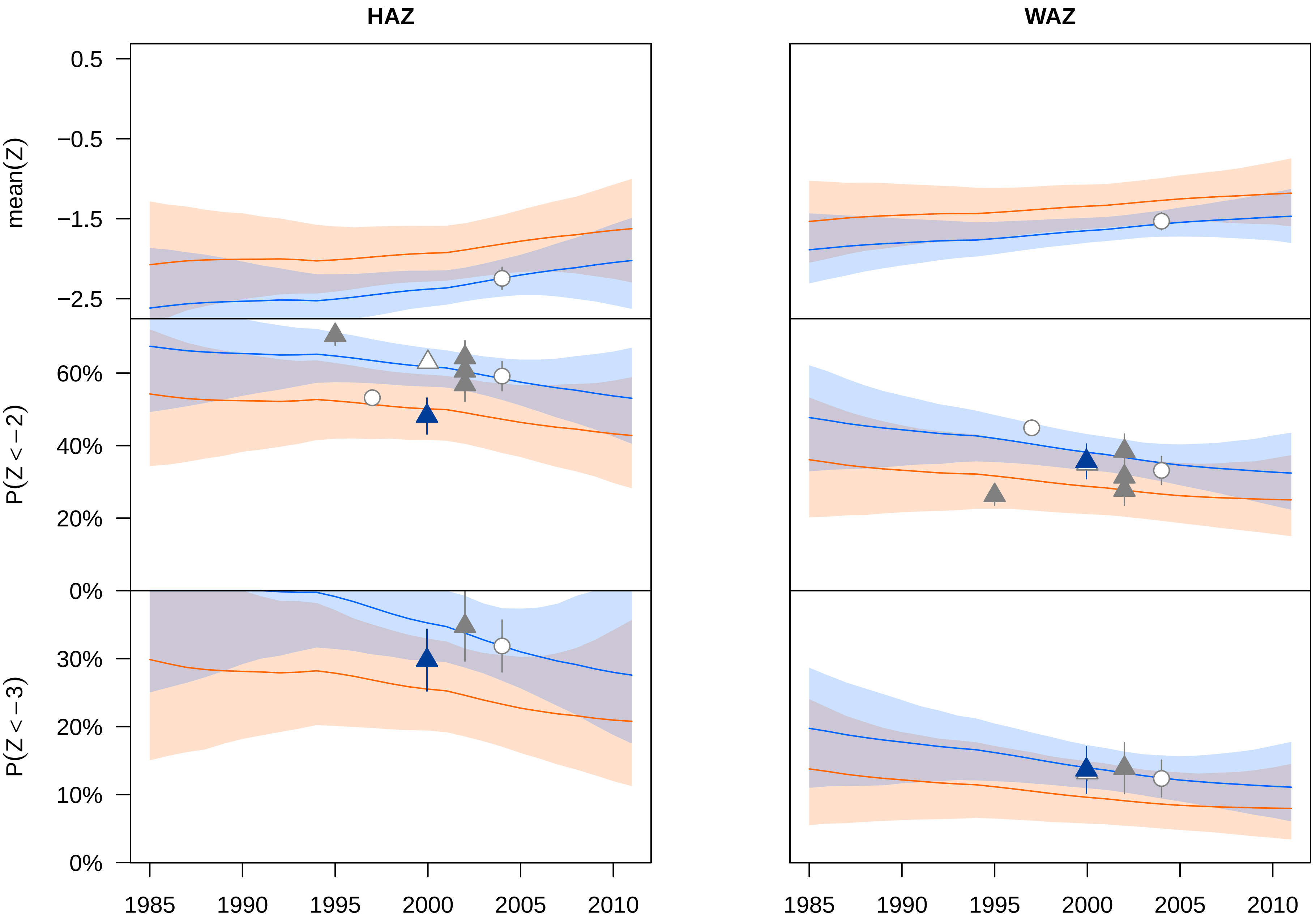

Algeria

Central Asia, Middle East, and North Africa Region

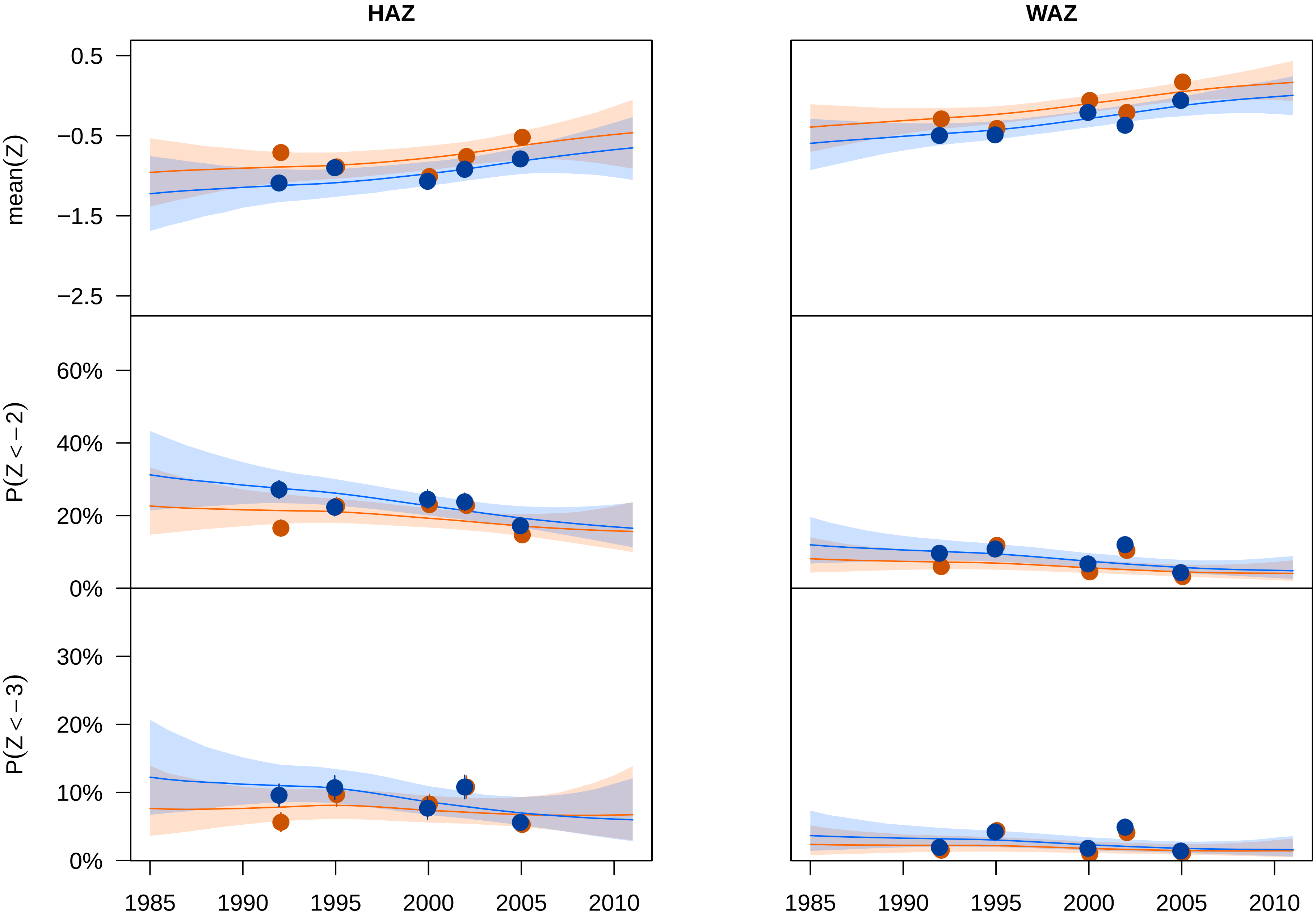

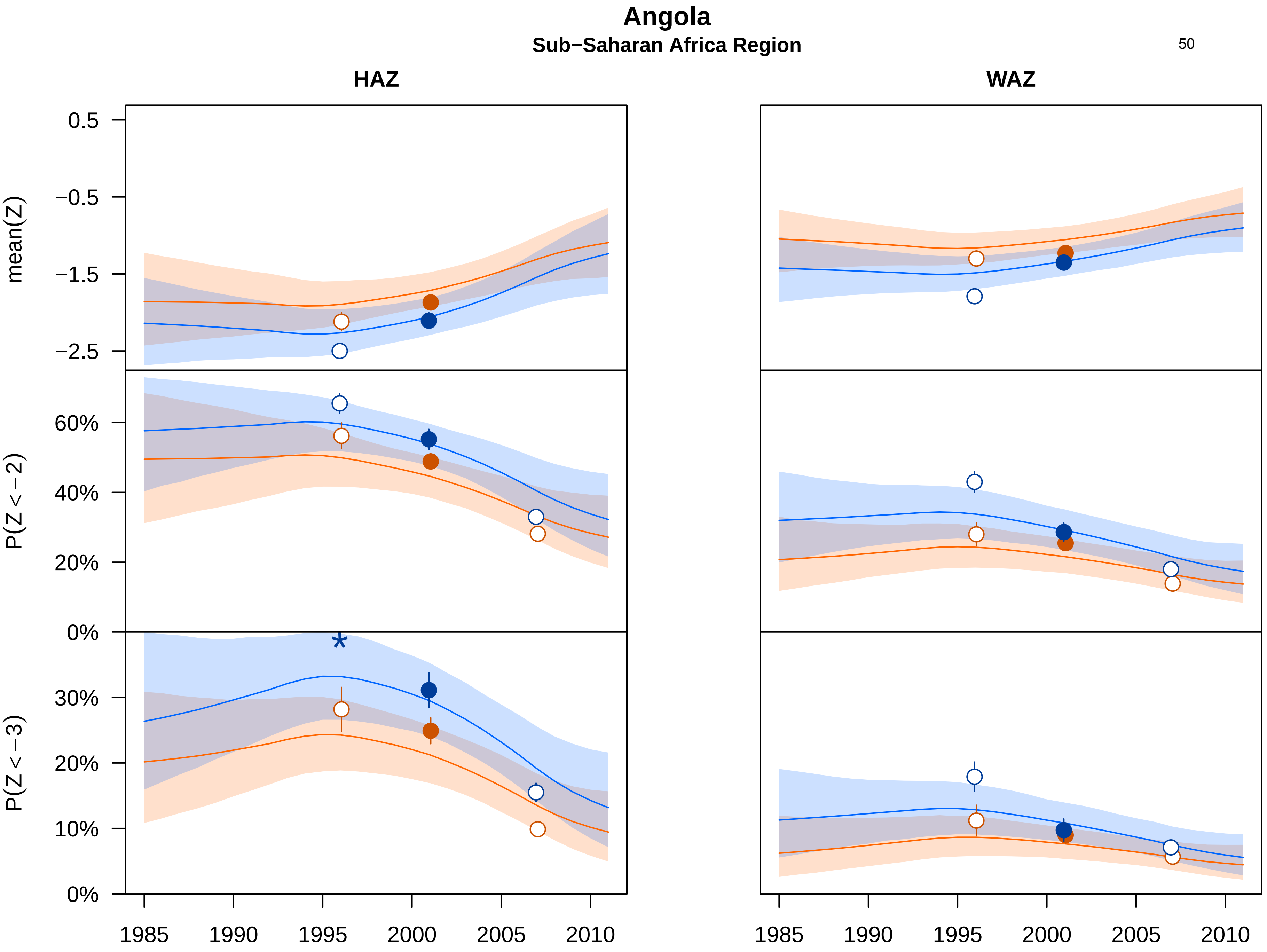
Angola
Sub−Saharan Africa Region
HAZ
WAZ
mean(Z)
P(Z < −2)
P(Z < −3)
0.5
−0.5
−1.5
−2.5
60%
40%
20%
0%
30%
20%
10%
0%
1985
1990
1995
2000
2005
2010

Antigua and Barbuda

Andean and Central Latin America and Caribbean Region

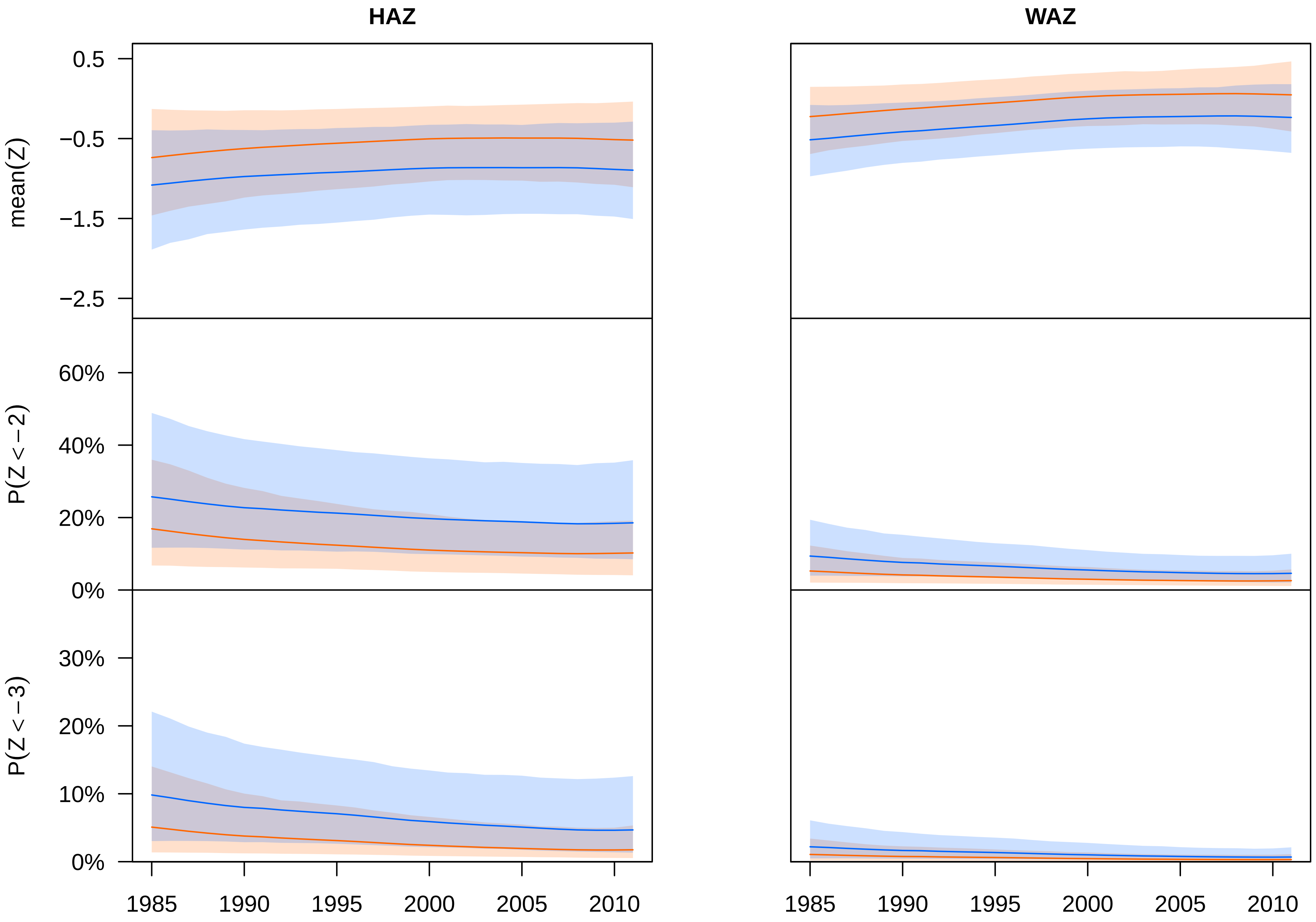

Argentina

Southern and Tropical Latin America Region

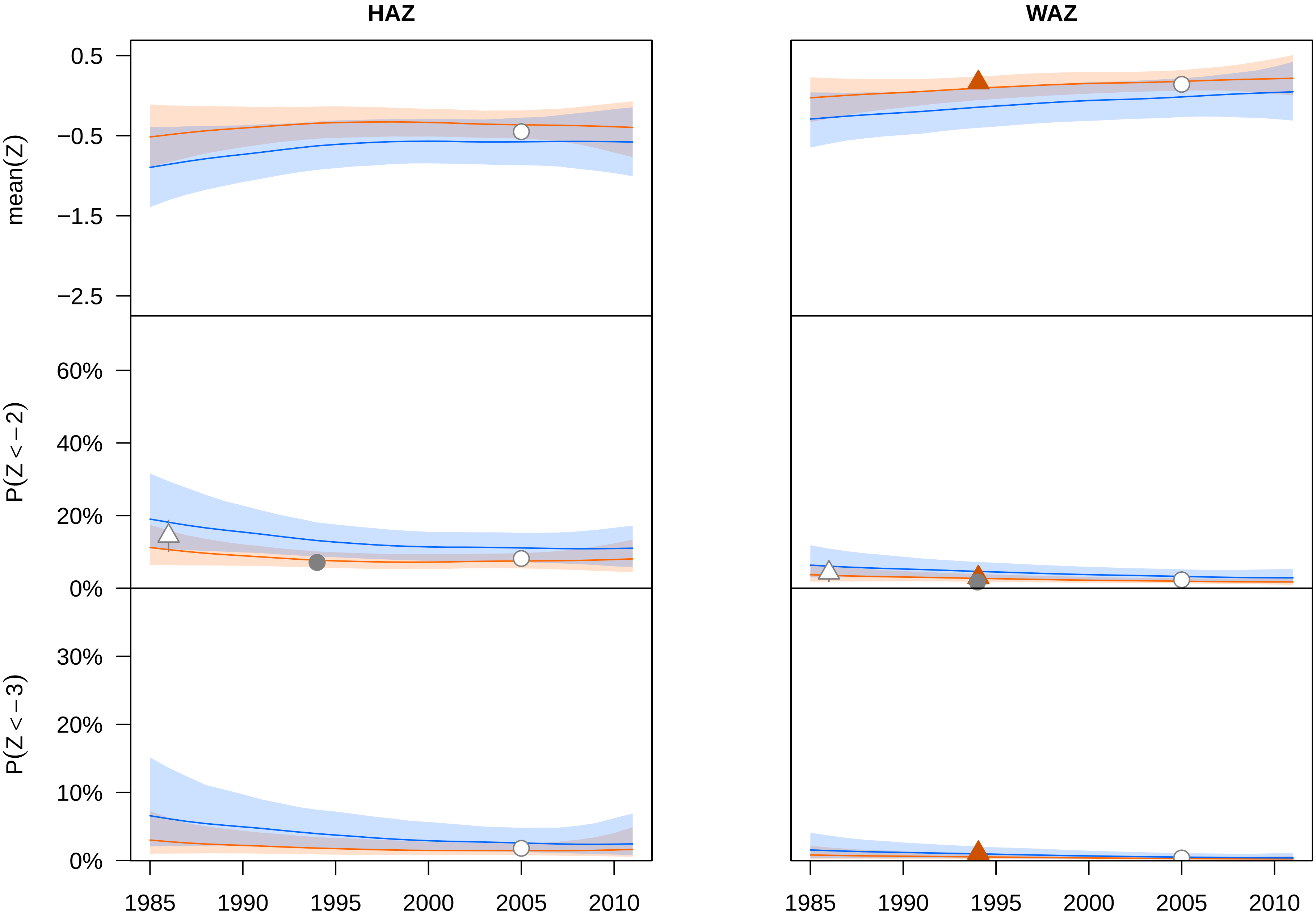

Armenia

Central Asia, Middle East, and North Africa Region

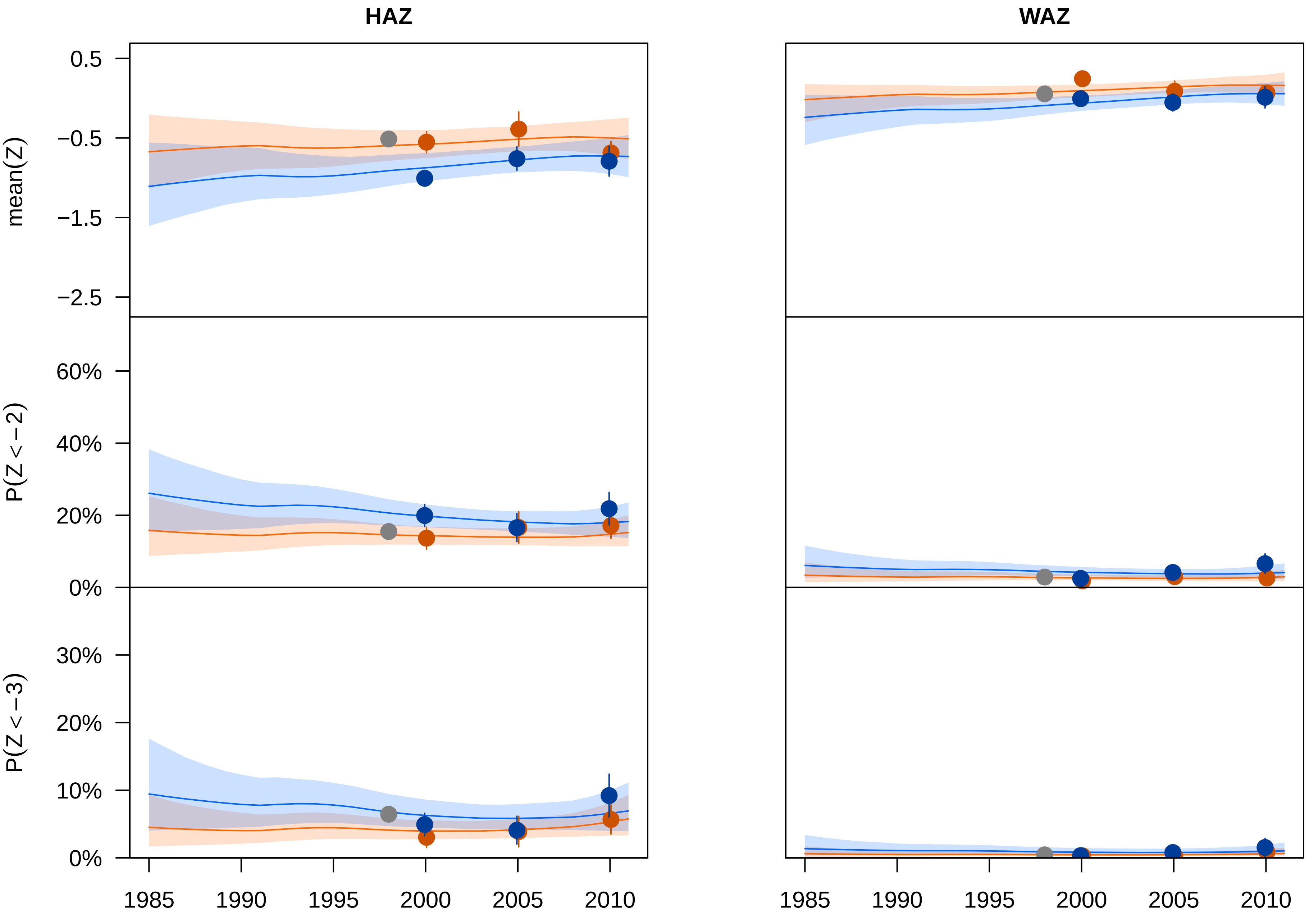

Azerbaijan

Central Asia, Middle East, and North Africa Region

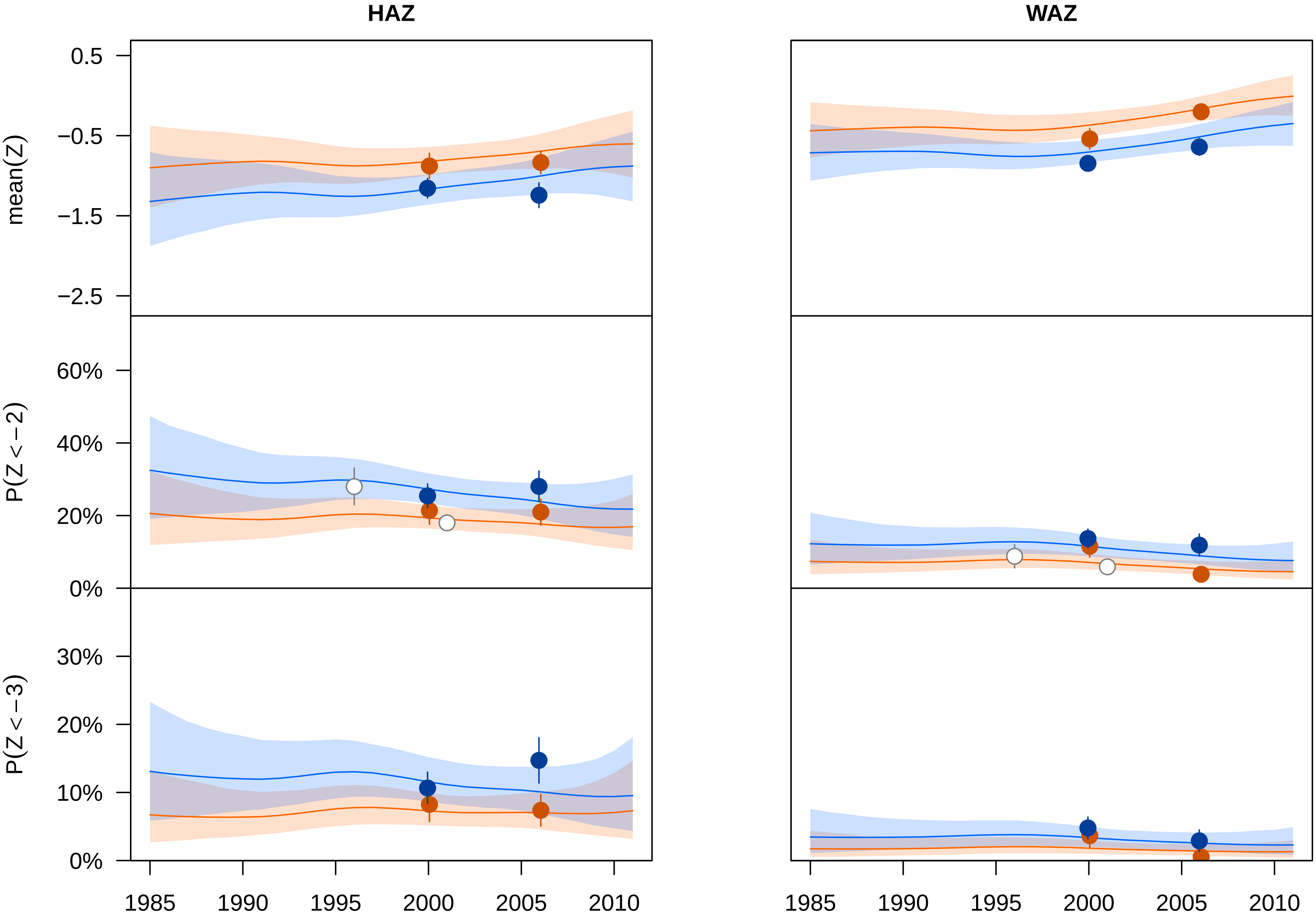

Bahamas

Andean and Central Latin America and Caribbean Region

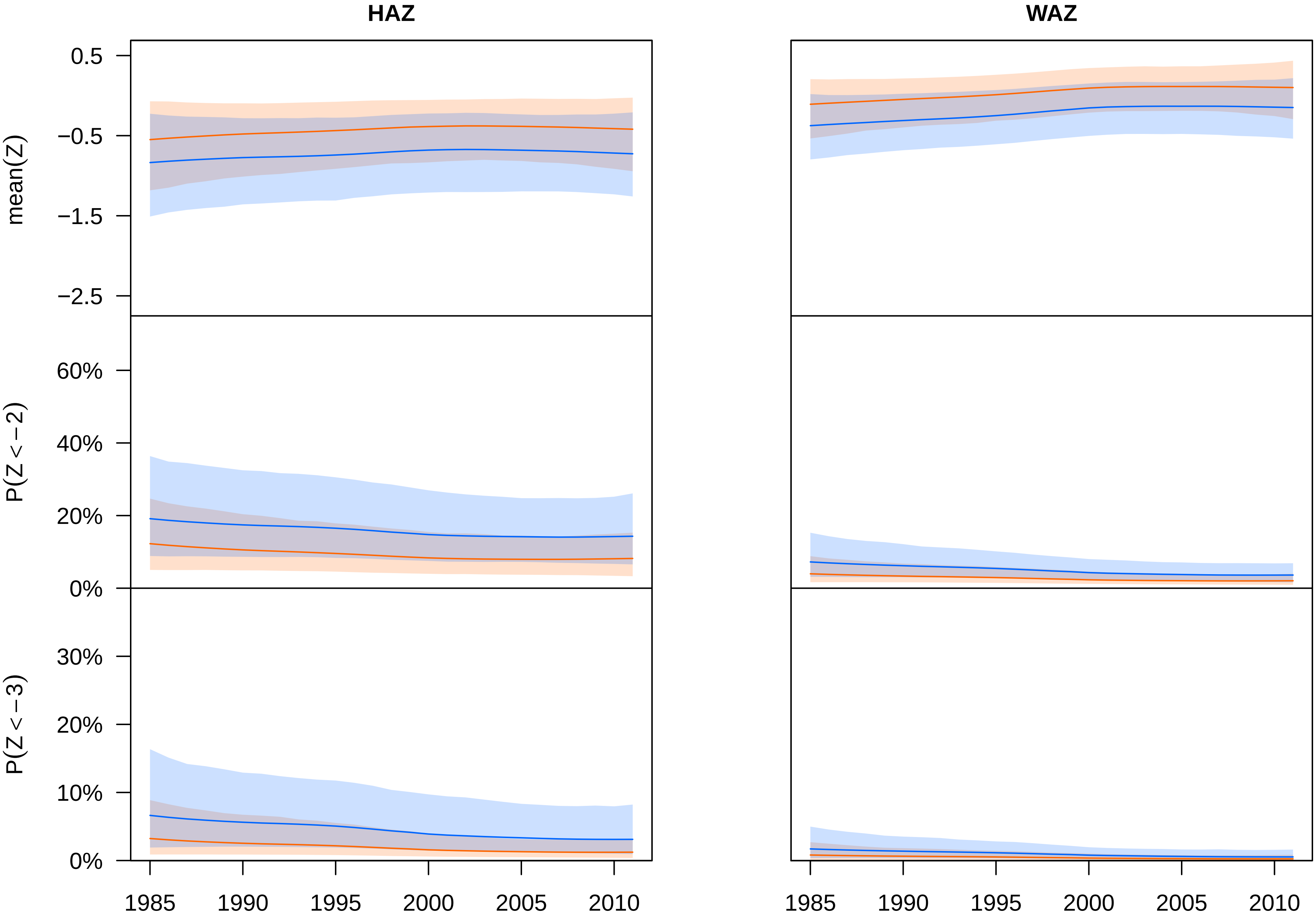

Bahrain

Central Asia, Middle East, and North Africa Region

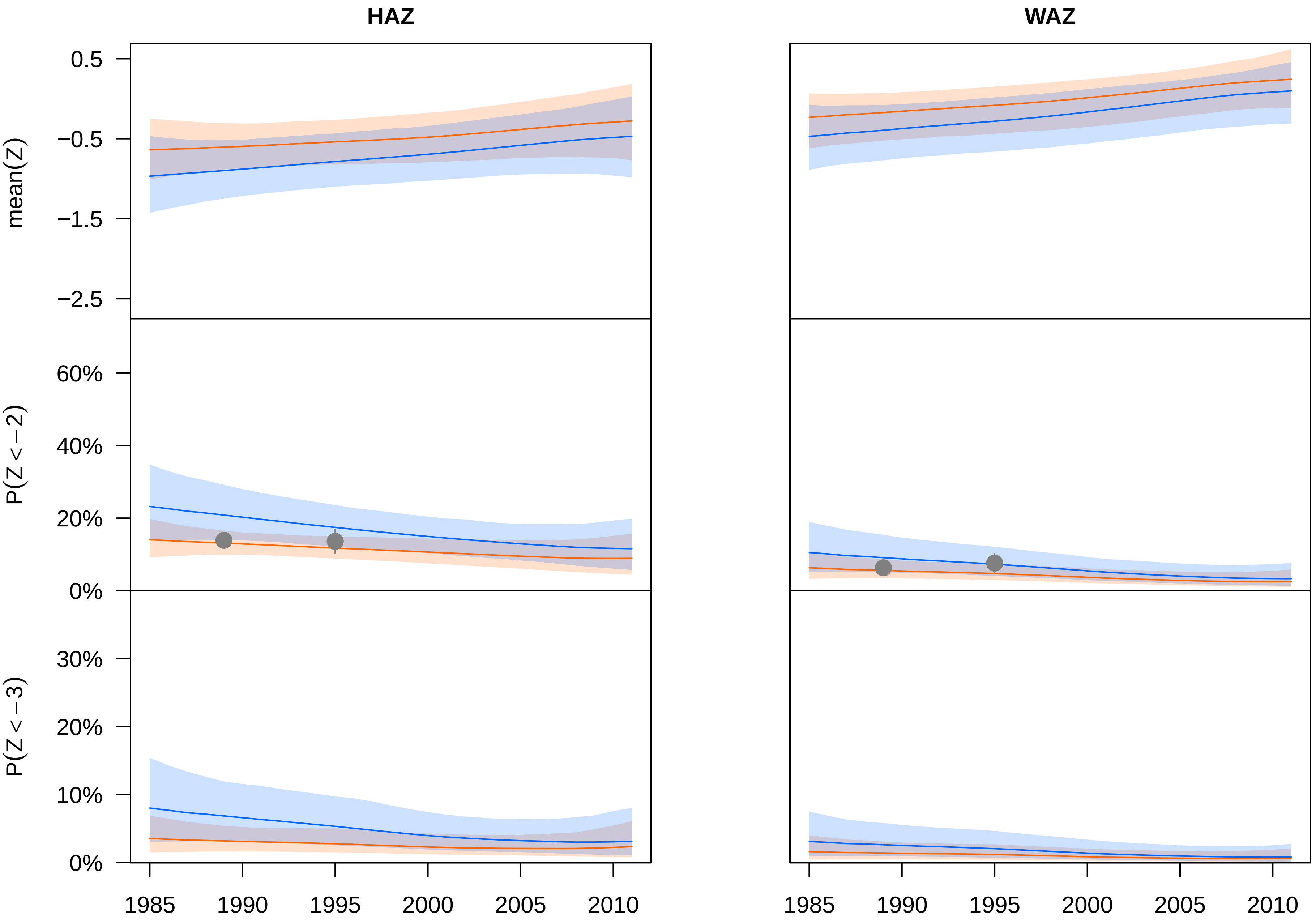

Bangladesh

South Asia Region

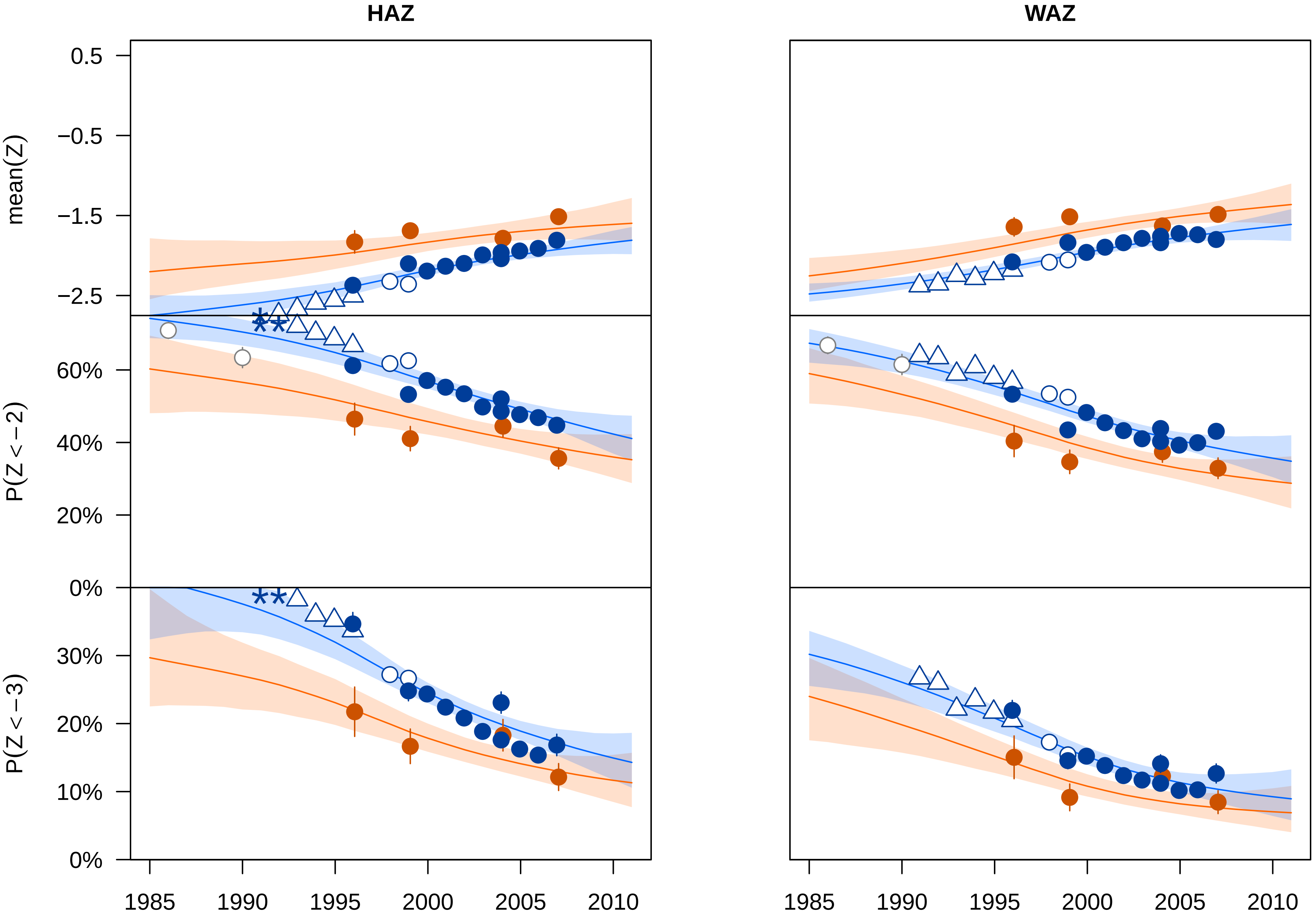

Barbados

Andean and Central Latin America and Caribbean Region

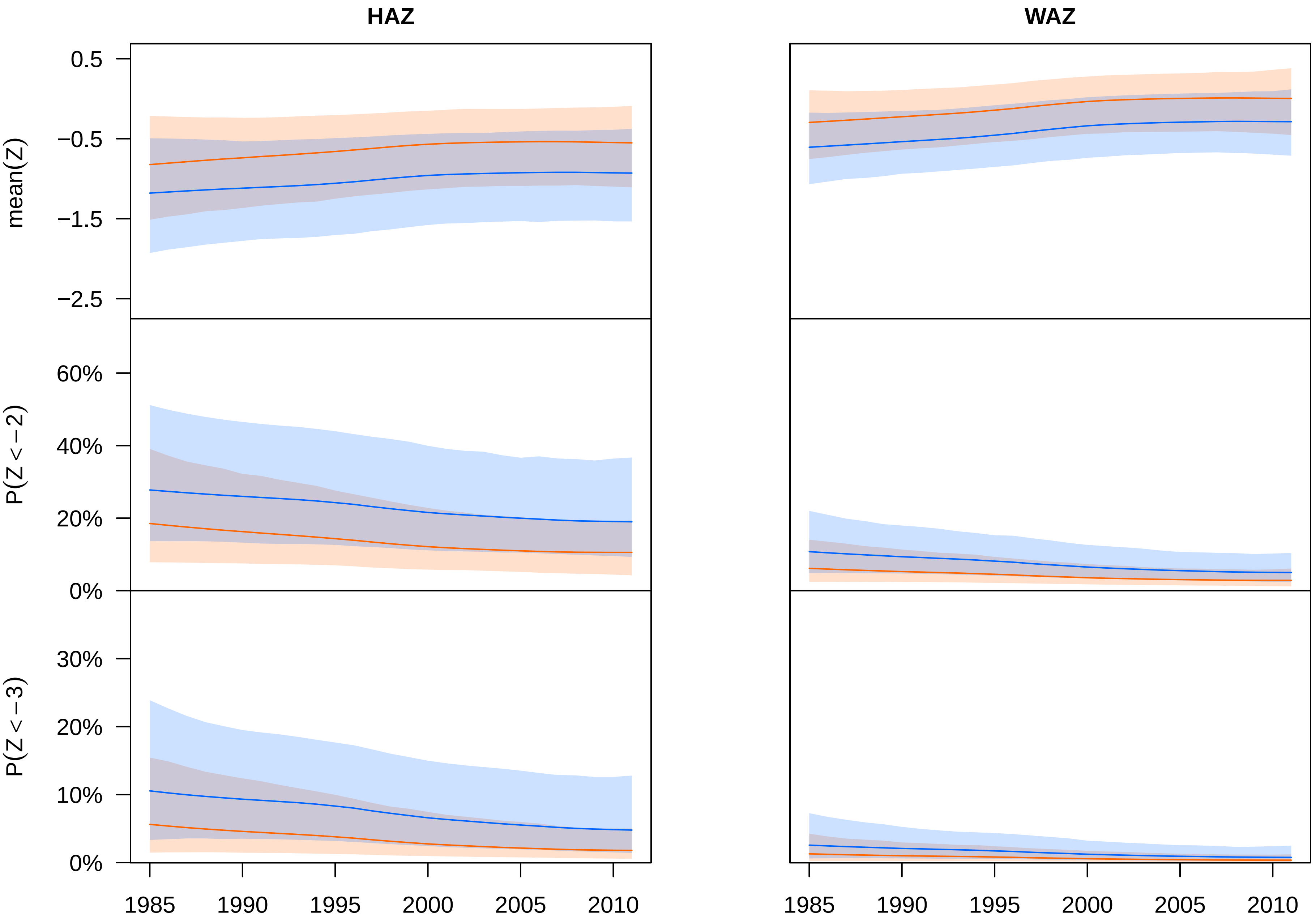

Belize

Andean and Central Latin America and Caribbean Region

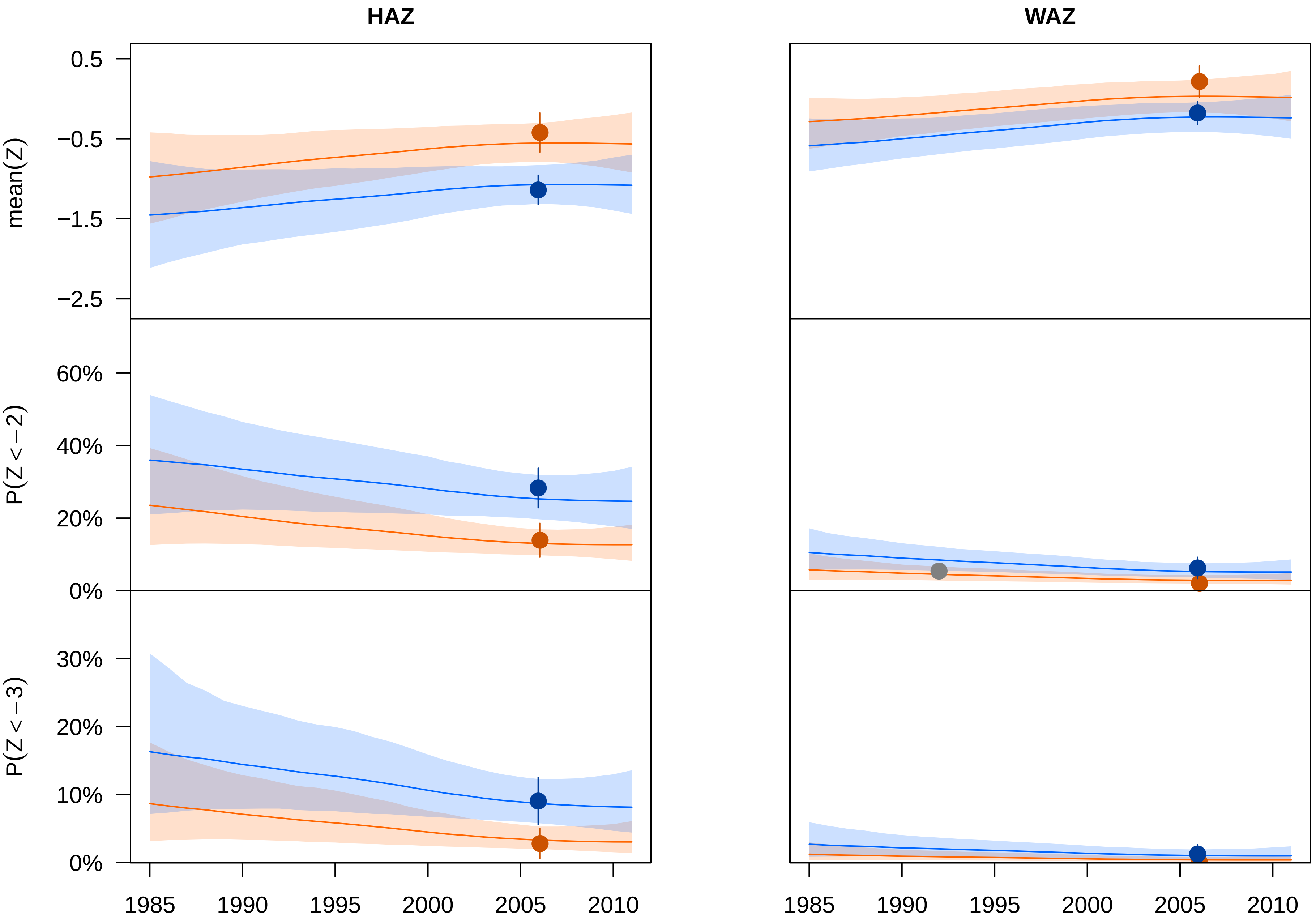

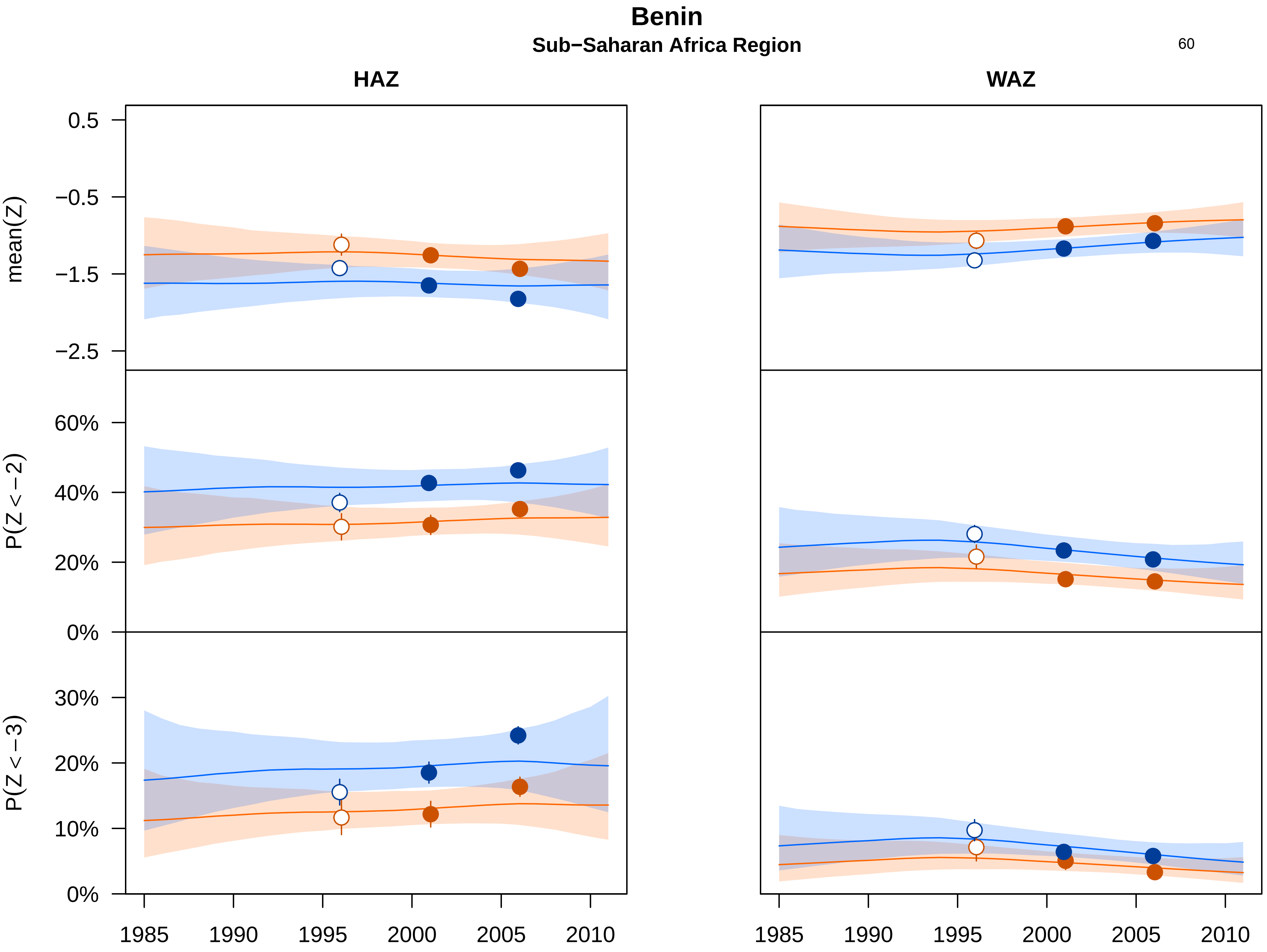
Benin
Sub−Saharan Africa Region
HAZ
WAZ
mean(Z)
P(Z < −2)
P(Z < −3)
0.5
−0.5
−1.5
−2.5
60%
40%
20%
0%
30%
20%
10%
0%
1985
1990
1995
2000
2005
2010

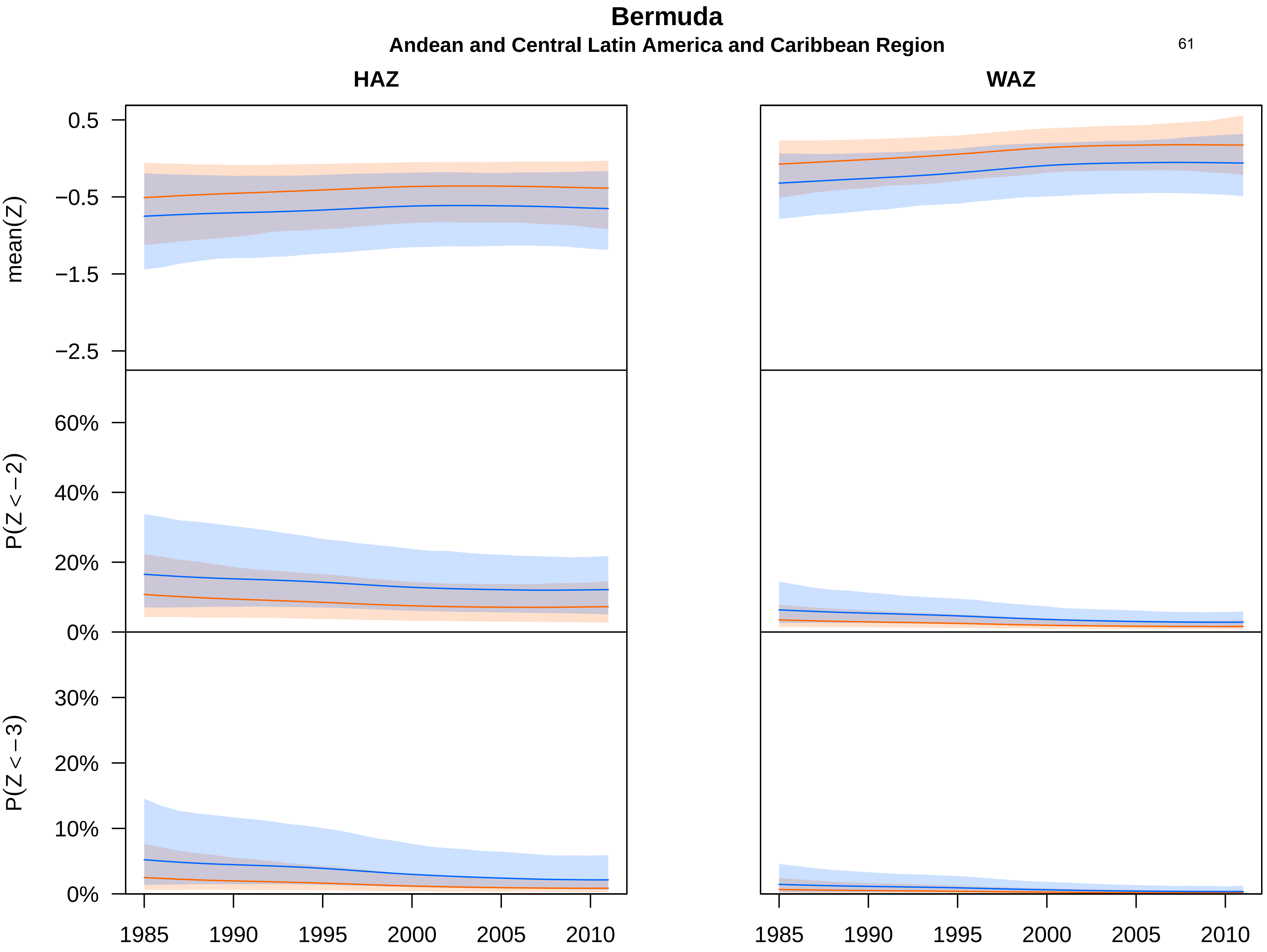
Bermuda
Andean and Central Latin America and Caribbean Region
HAZ
WAZ
mean(Z)
P(Z < −2)
P(Z < −3)
0.5
−0.5
−1.5
−2.5
60%
40%
20%
0%
30%
20%
10%
0%
1985
1990
1995
2000
2005
2010

Bhutan

South Asia Region

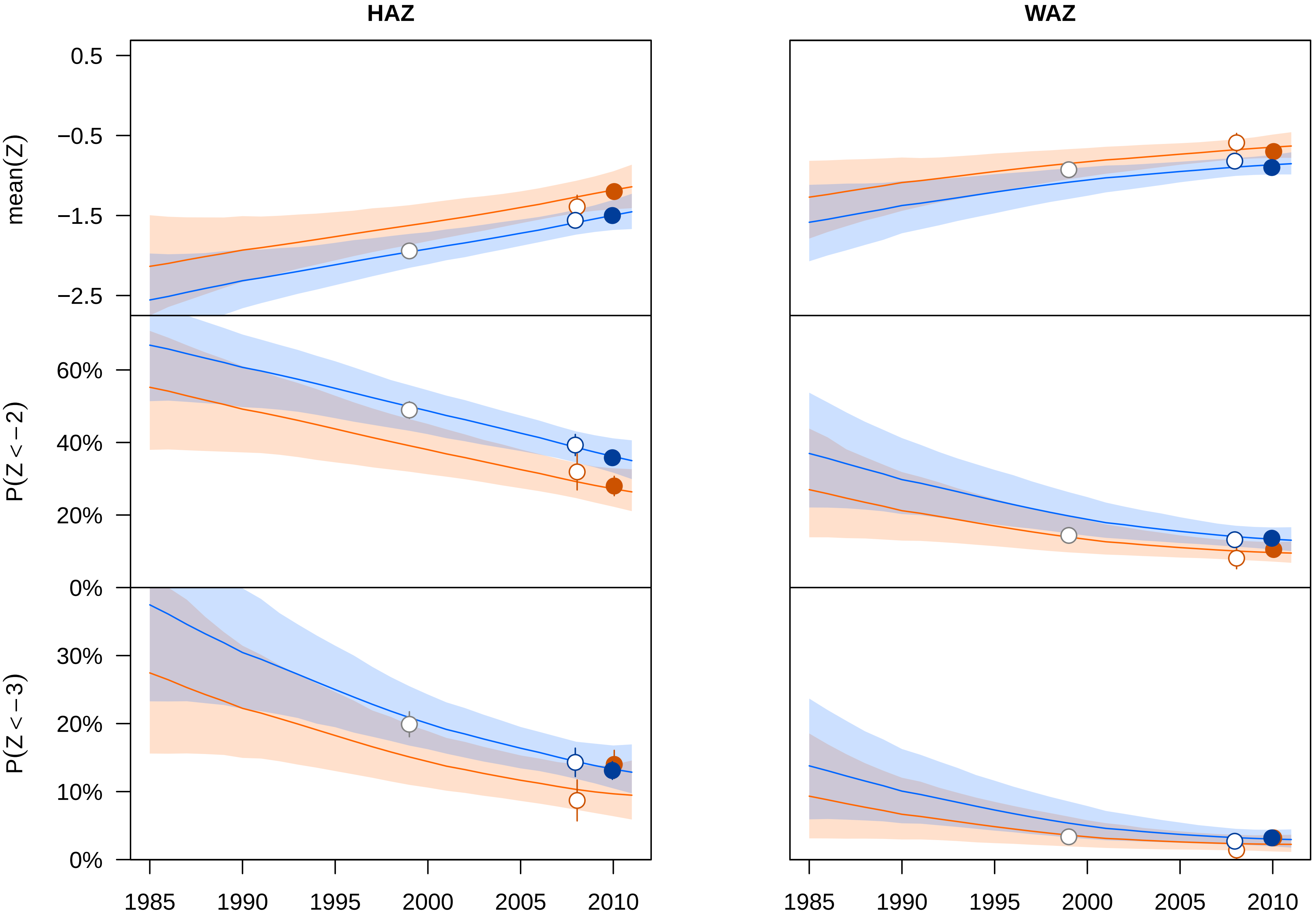

Bolivia

Andean and Central Latin America and Caribbean Region

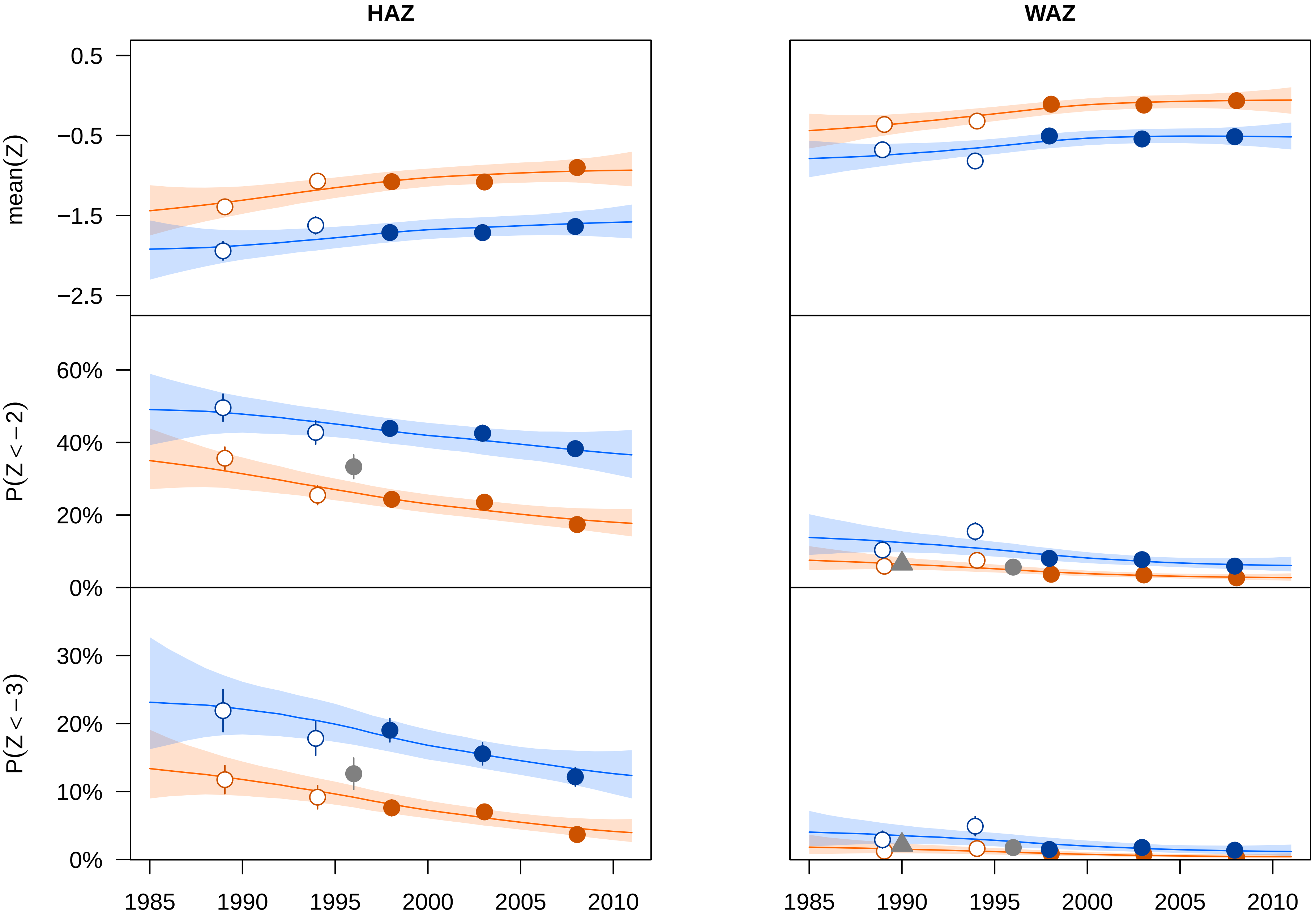

Botswana

Sub−Saharan Africa Region

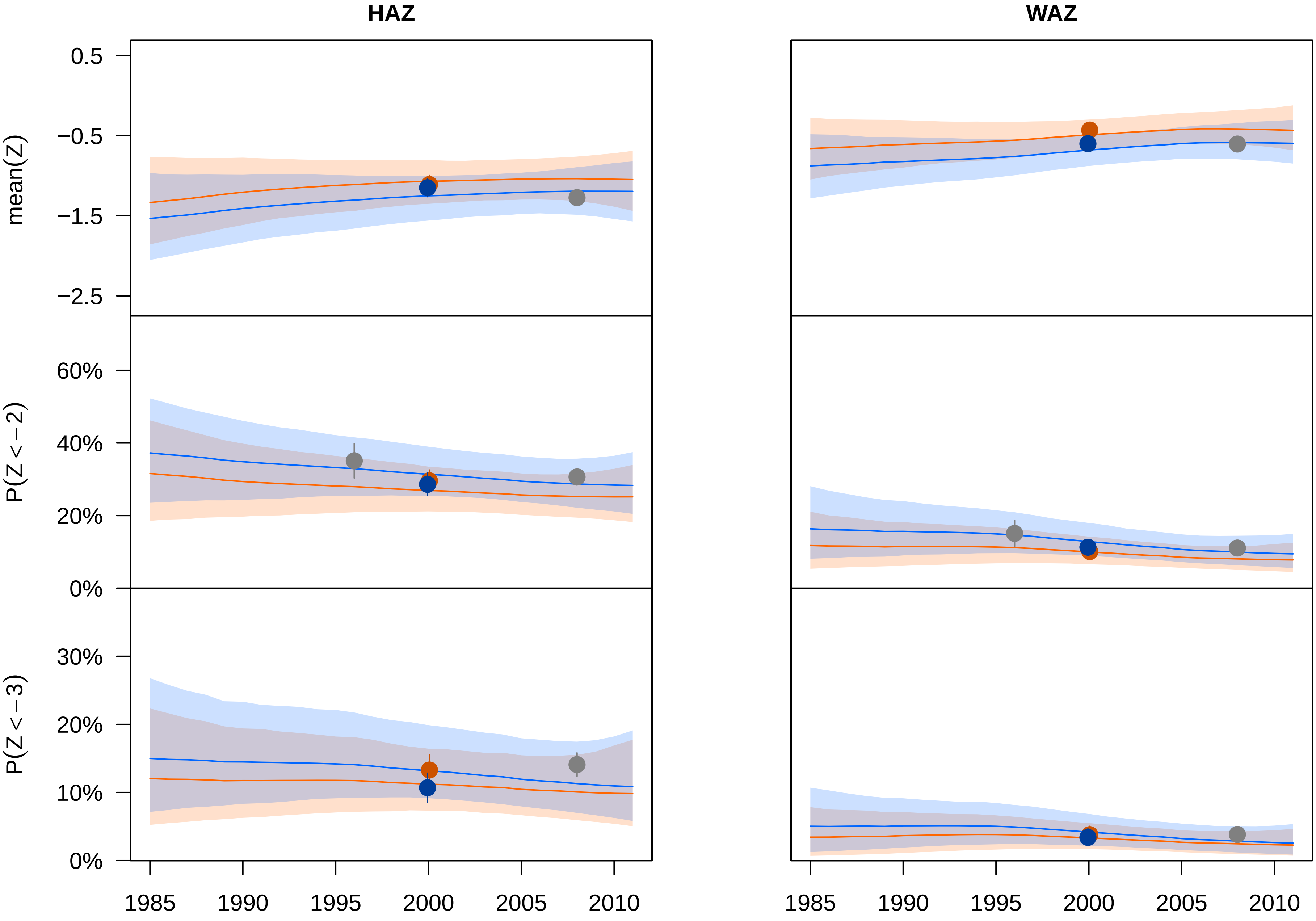

Brazil

Southern and Tropical Latin America Region

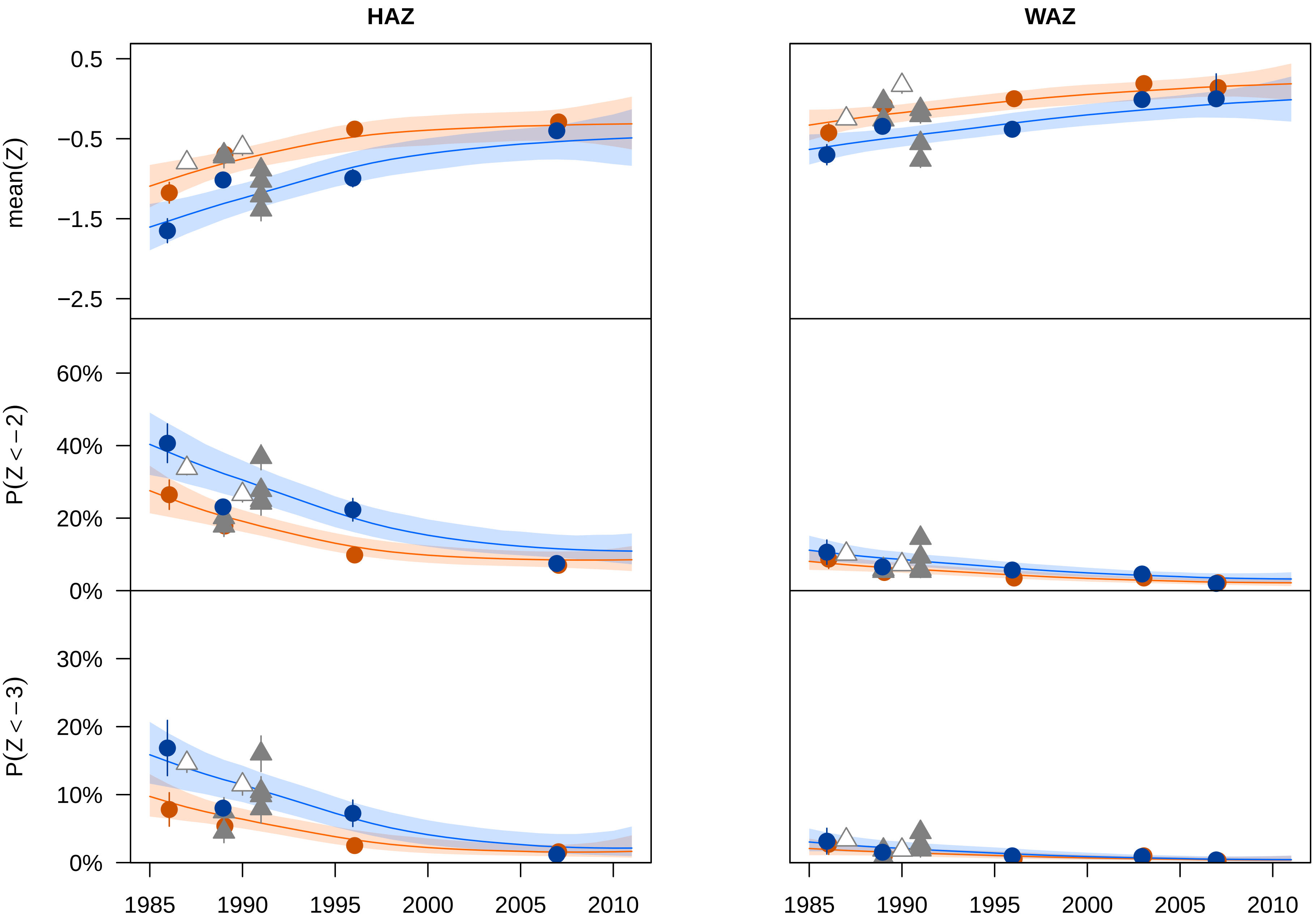

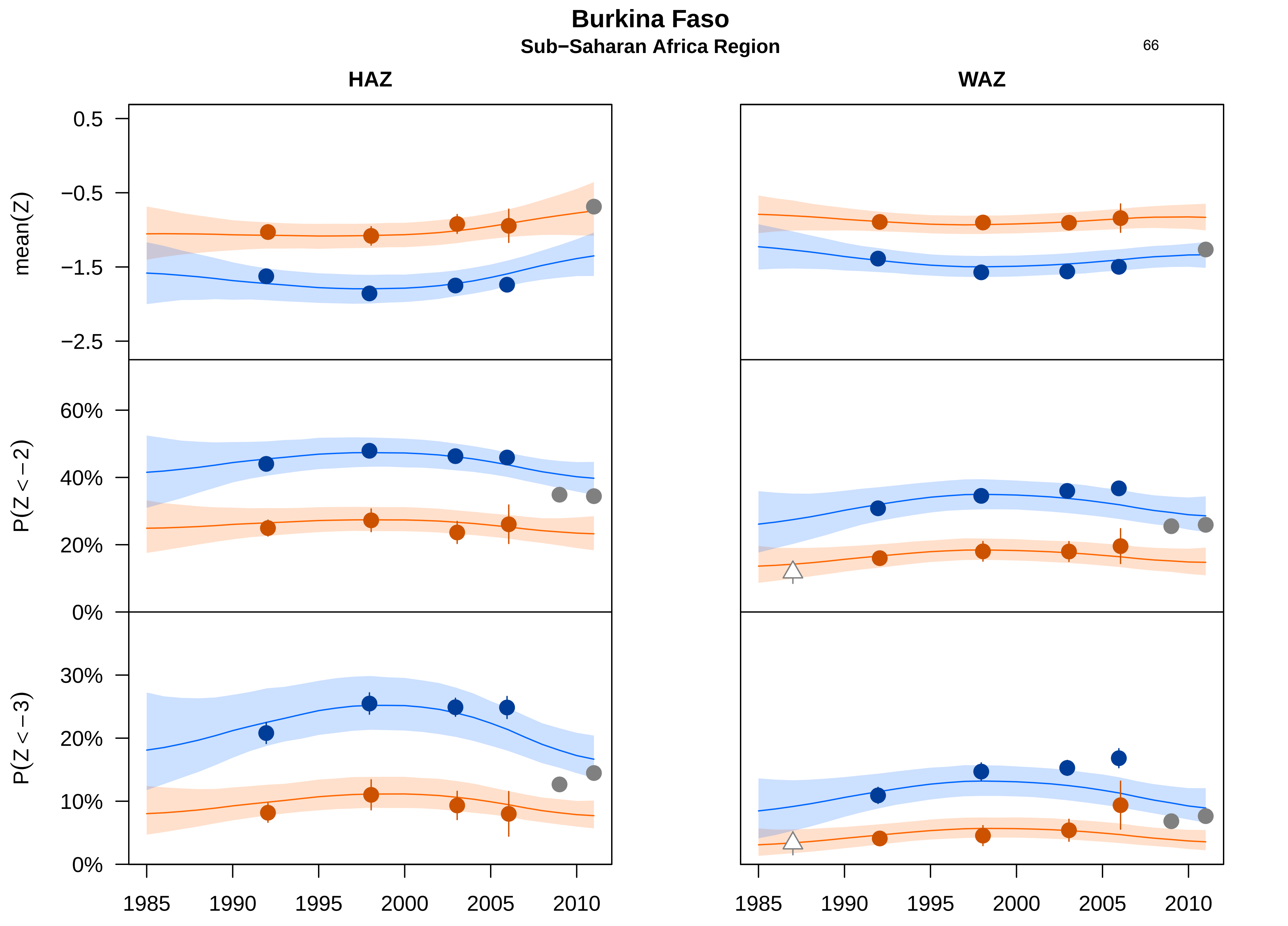

Burkina Faso
Sub−Saharan Africa Region
66
HAZ
WAZ
mean(Z)
P(Z < −2)
P(Z < −3)
0.5
−0.5
−1.5
−2.5
60%
40%
20%
0%
30%
20%
10%
0%
1985
1990
1995
2000
2005
2010

Burundi

Sub−Saharan Africa Region

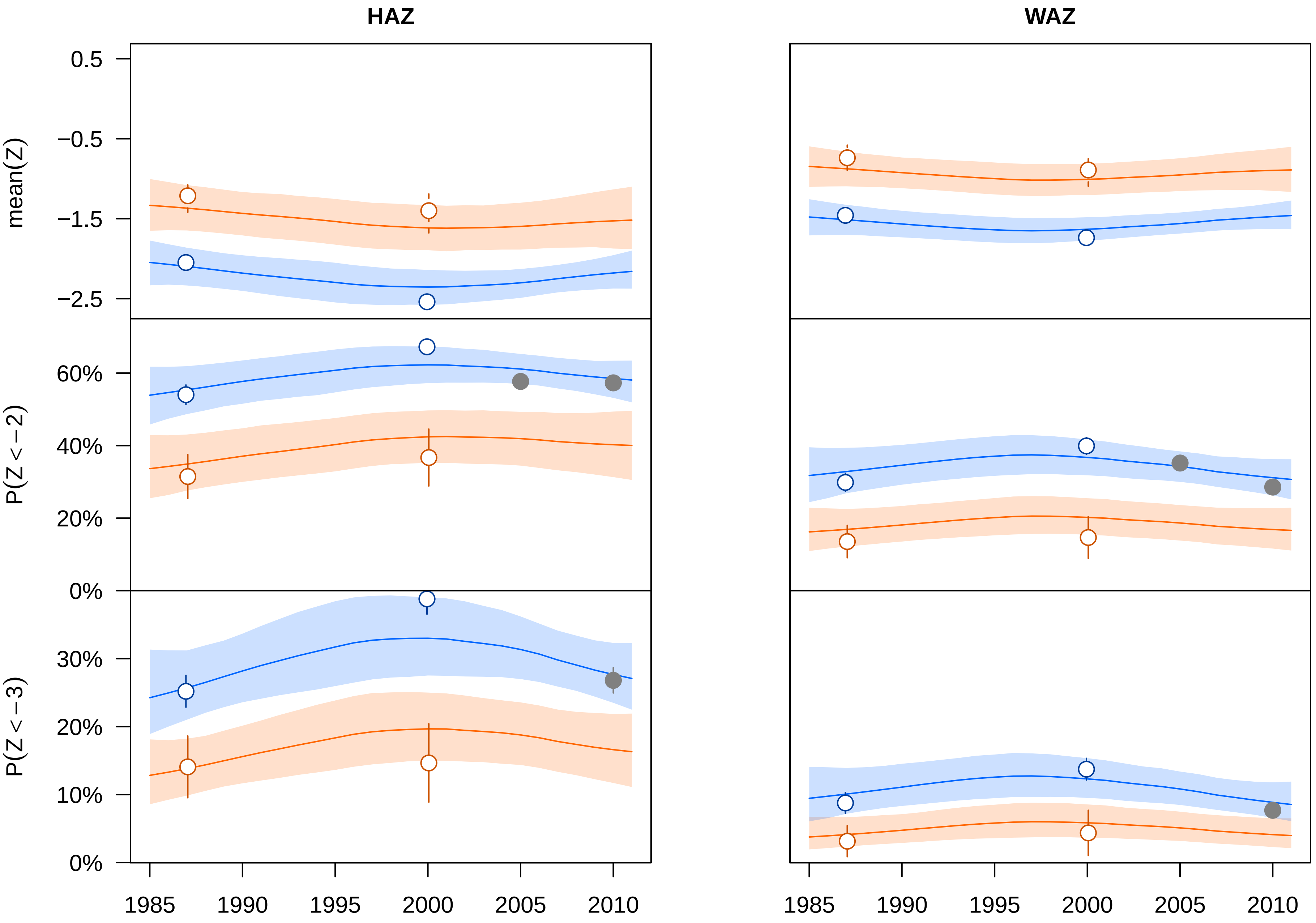

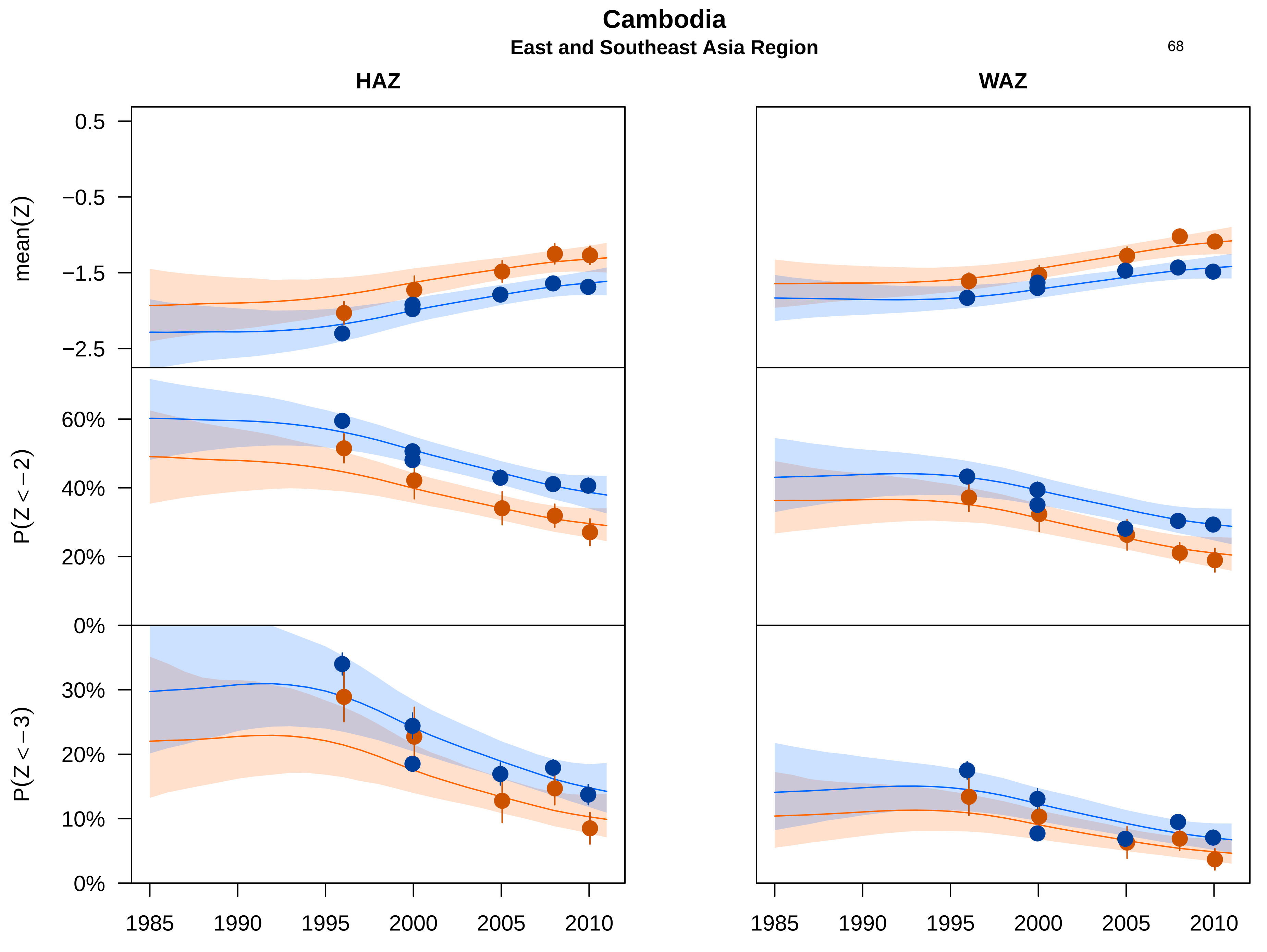
Cambodia
East and Southeast Asia Region
HAZ
WAZ
mean(Z)
P(Z < −2)
P(Z < −3)
0.5
−0.5
−1.5
−2.5
60%
40%
20%
0%
30%
20%
10%
0%
1985
1990
1995
2000
2005
2010

Cameroon

Sub−Saharan Africa Region

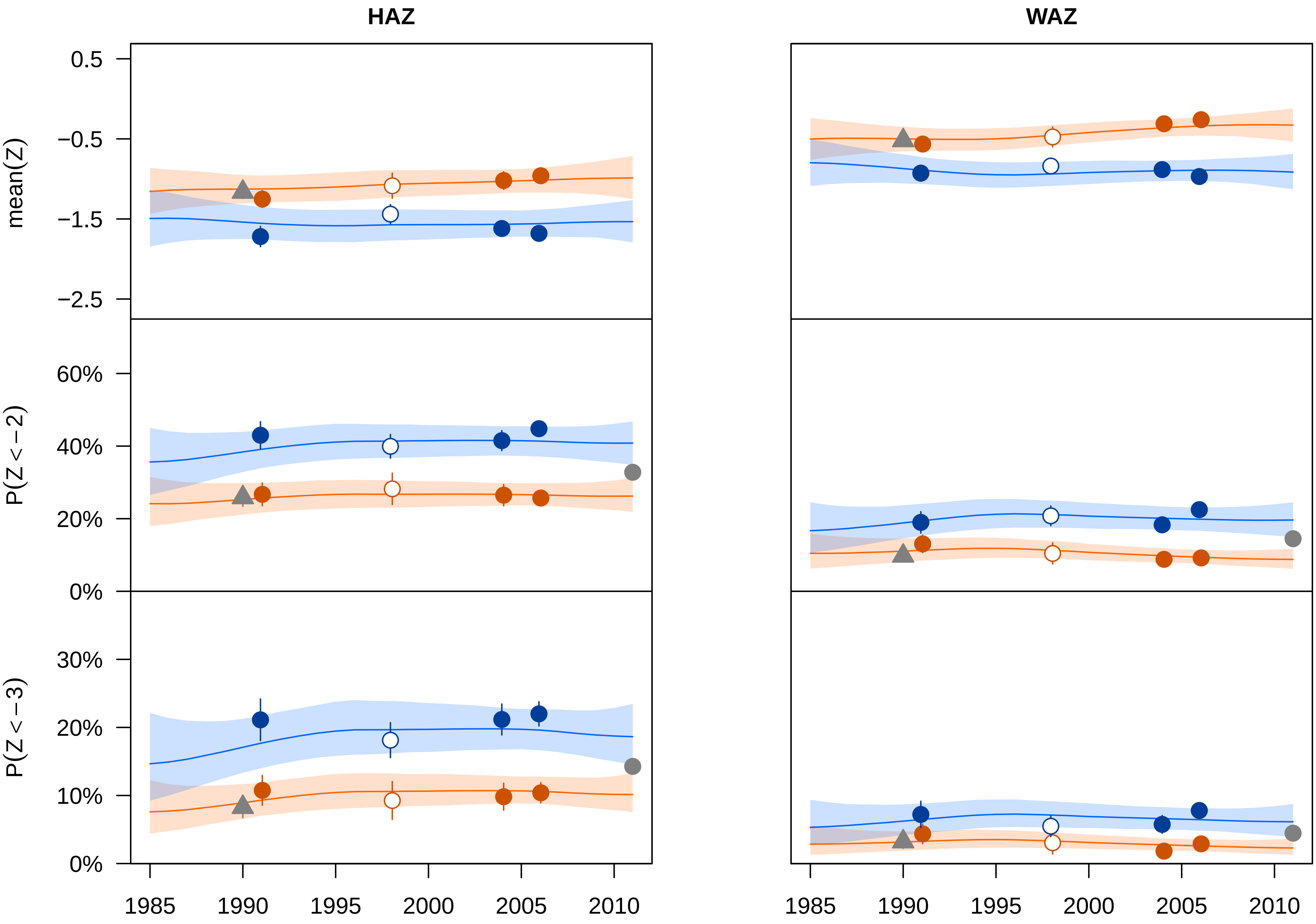

Cape Verde

Sub−Saharan Africa Region

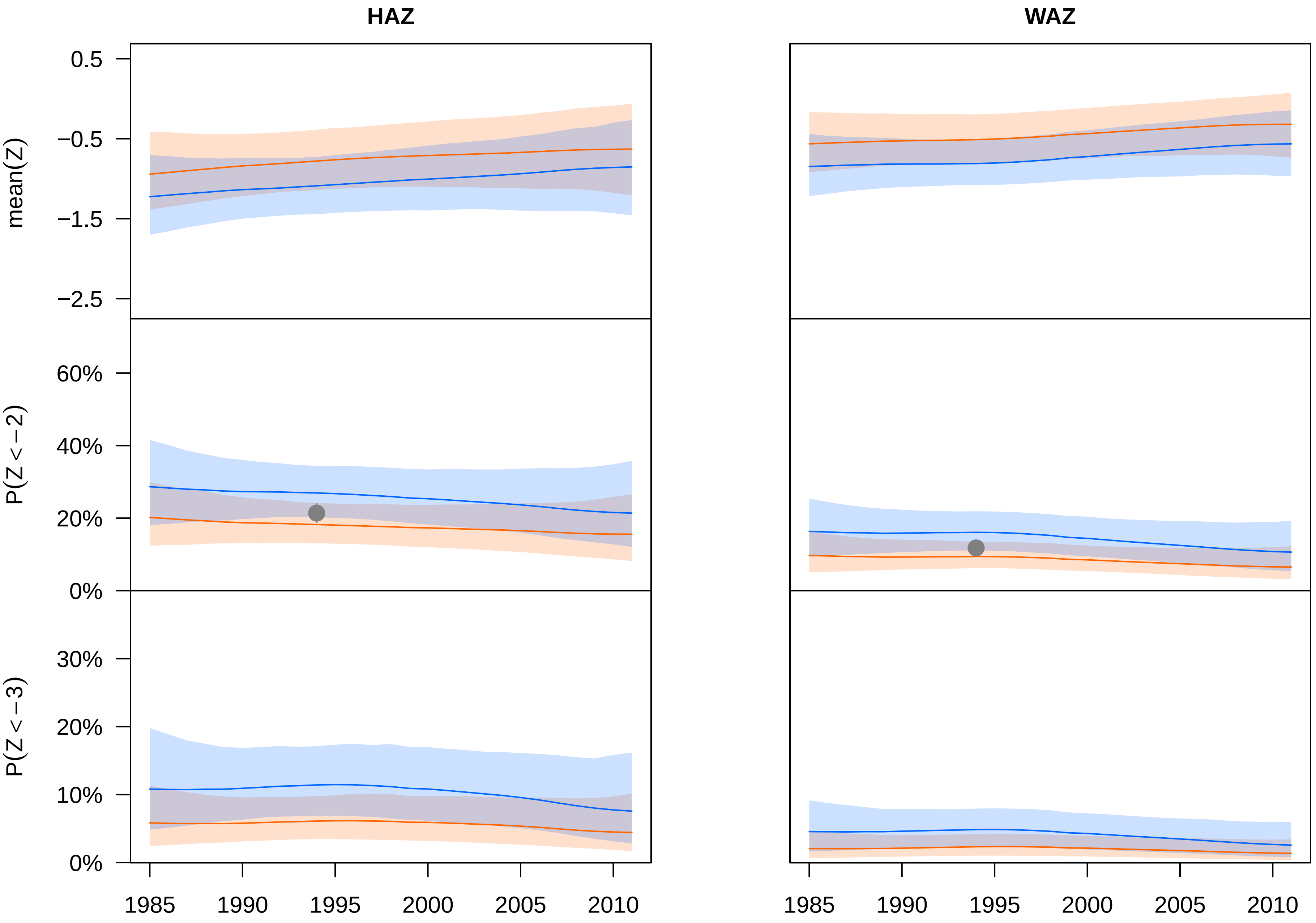

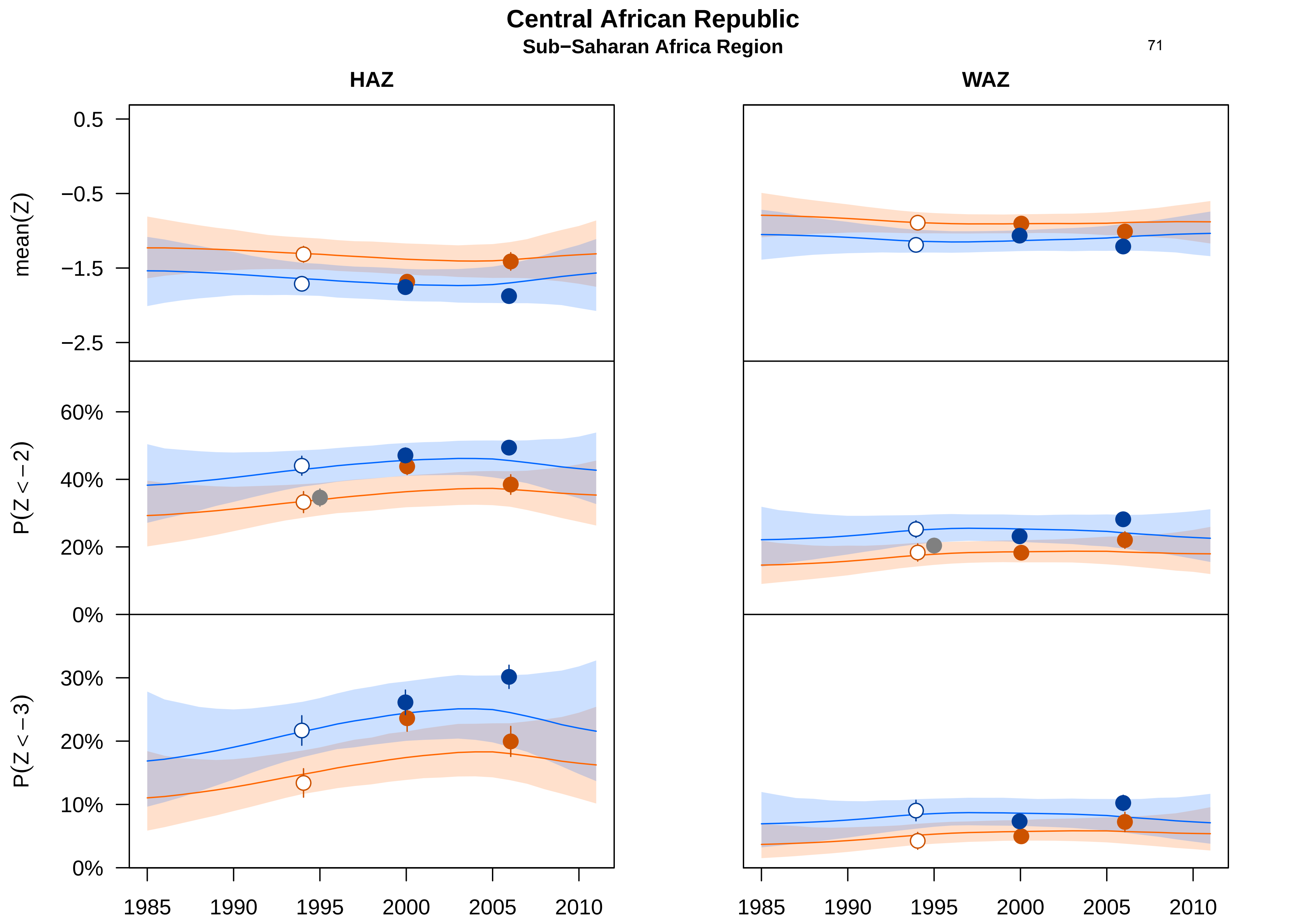
Central African Republic
Sub−Saharan Africa Region
HAZ
WAZ
mean(Z)
P(Z < −2)
P(Z < −3)
0.5
−0.5
−1.5
−2.5
60%
40%
20%
0%
30%
20%
10%
0%
1985
1990
1995
2000
2005
2010

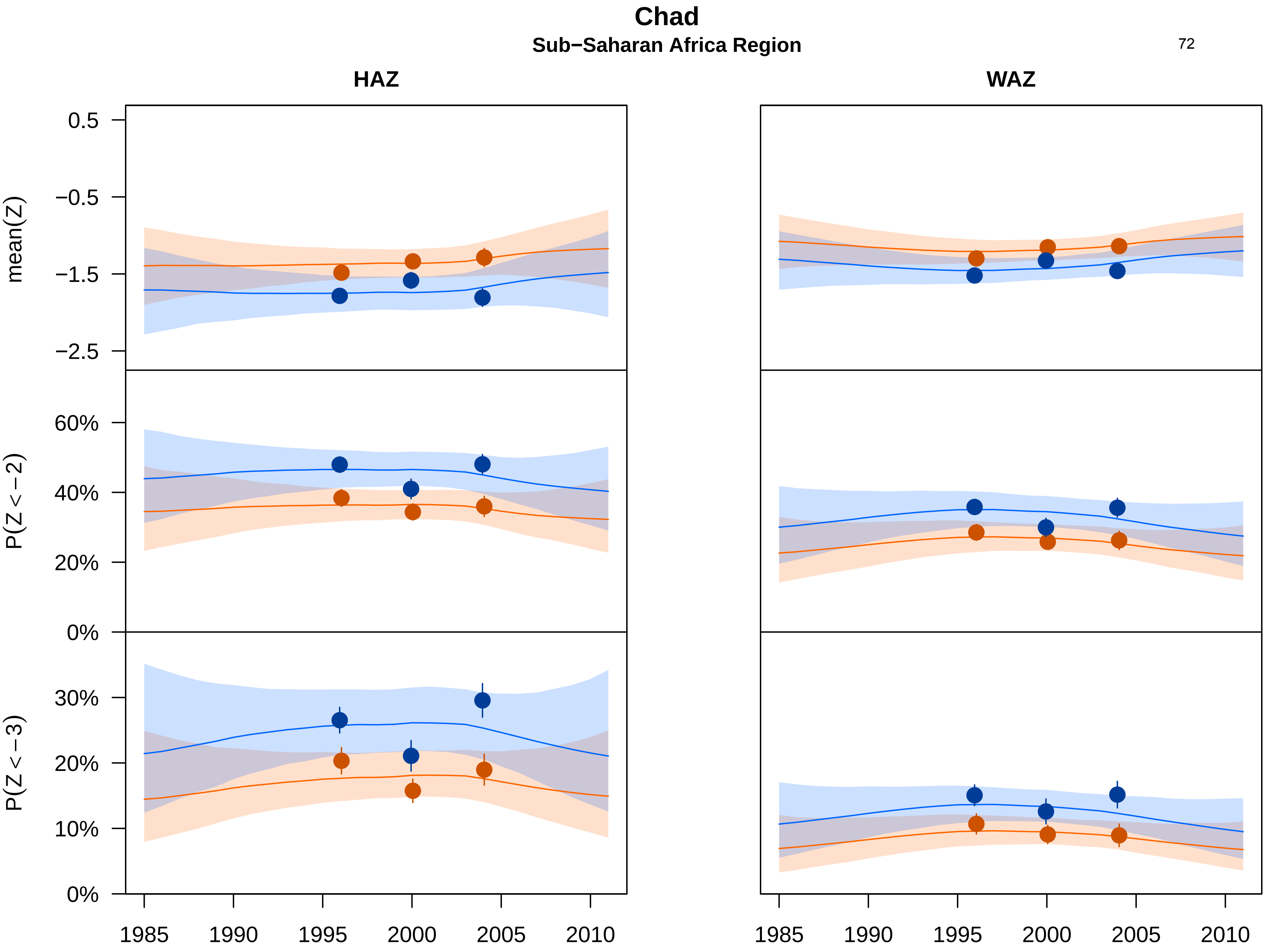
Chad
Sub−Saharan Africa Region
HAZ
WAZ
mean(Z)
P(Z < −2)
P(Z < −3)
0.5
−0.5
−1.5
−2.5
60%
40%
20%
0%
30%
20%
10%
0%
1985
1990
1995
2000
2005
2010

Chile

Southern and Tropical Latin America Region

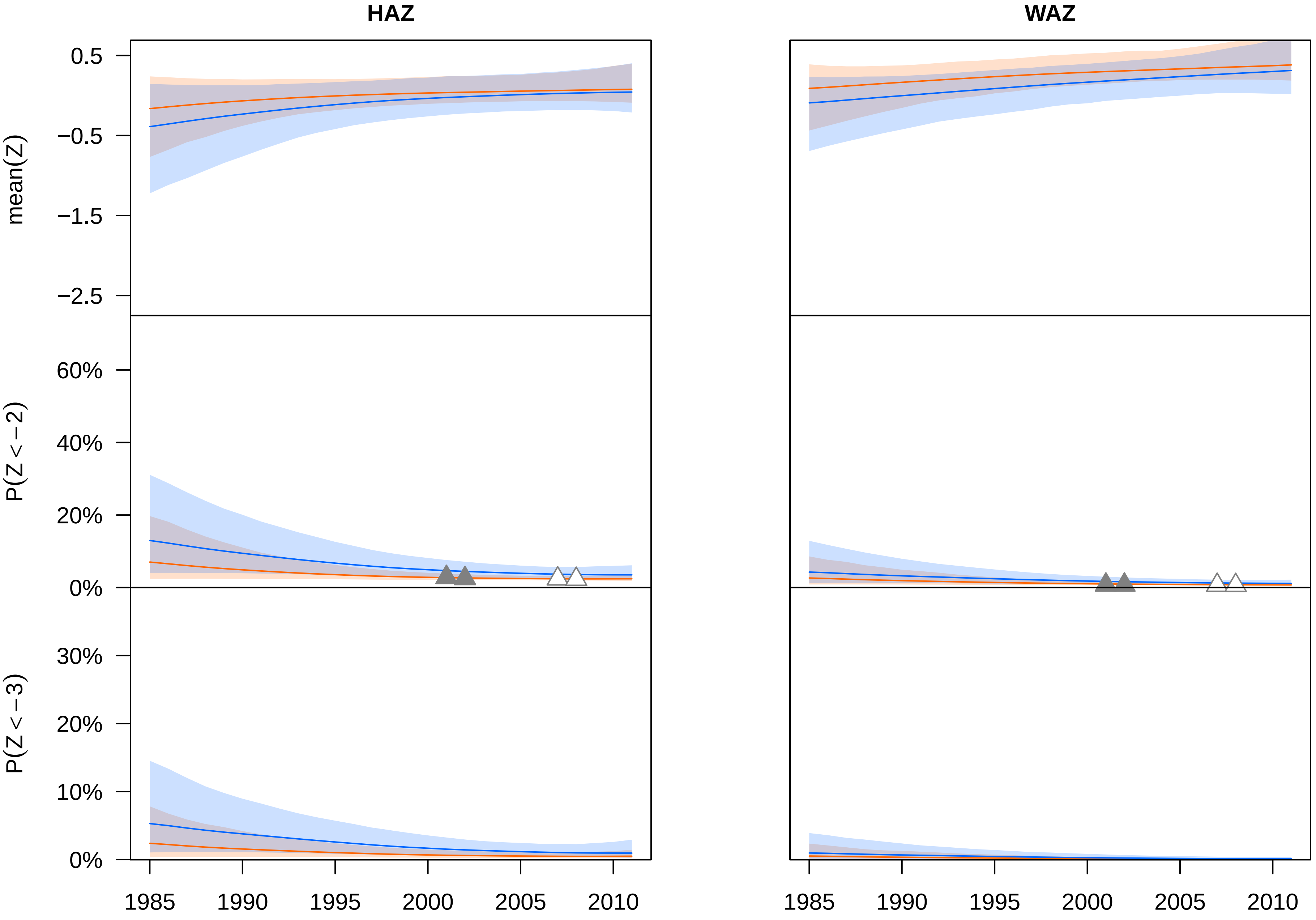

China

East and Southeast Asia Region

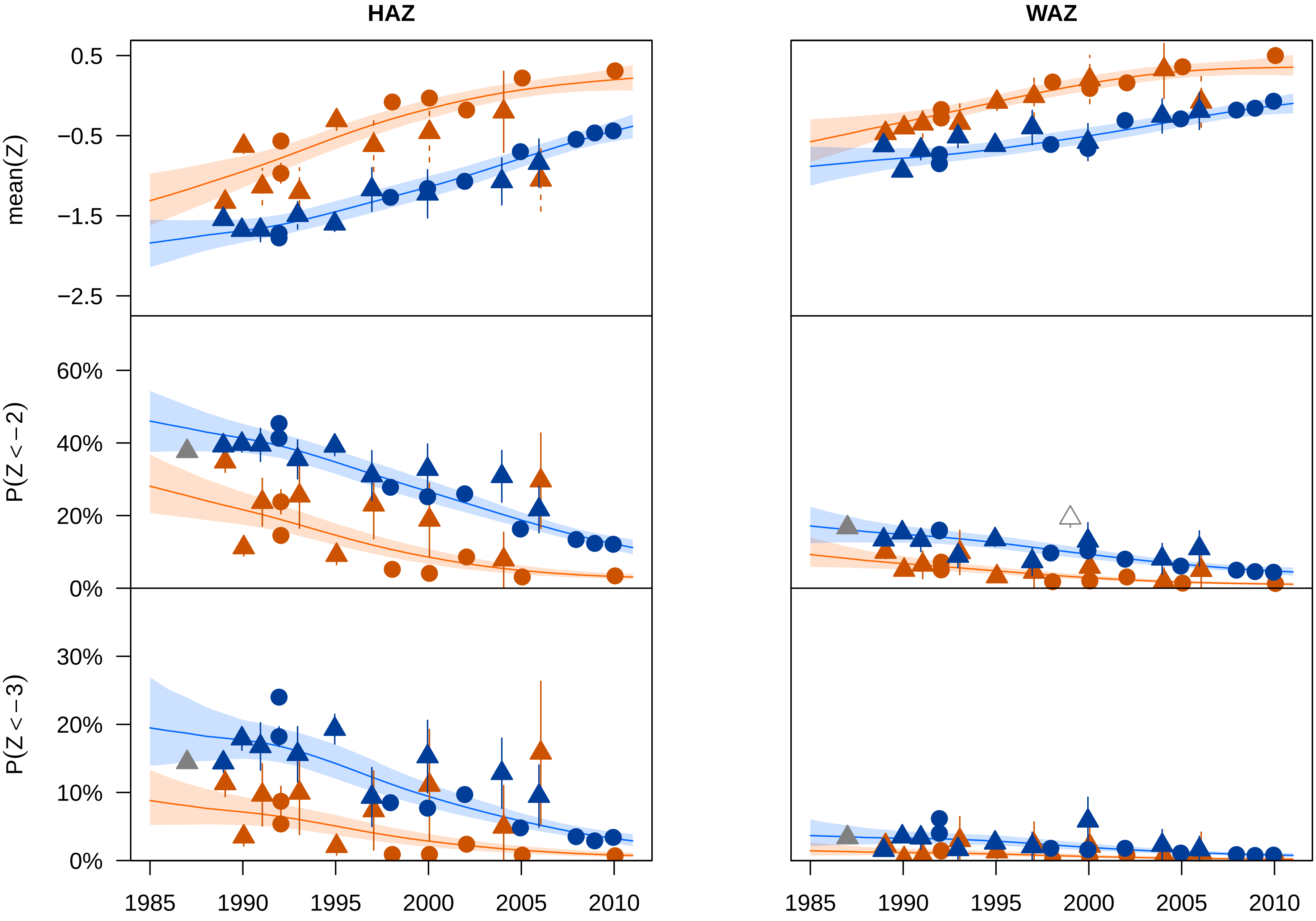

China (Hong Kong SAR)

East and Southeast Asia Region

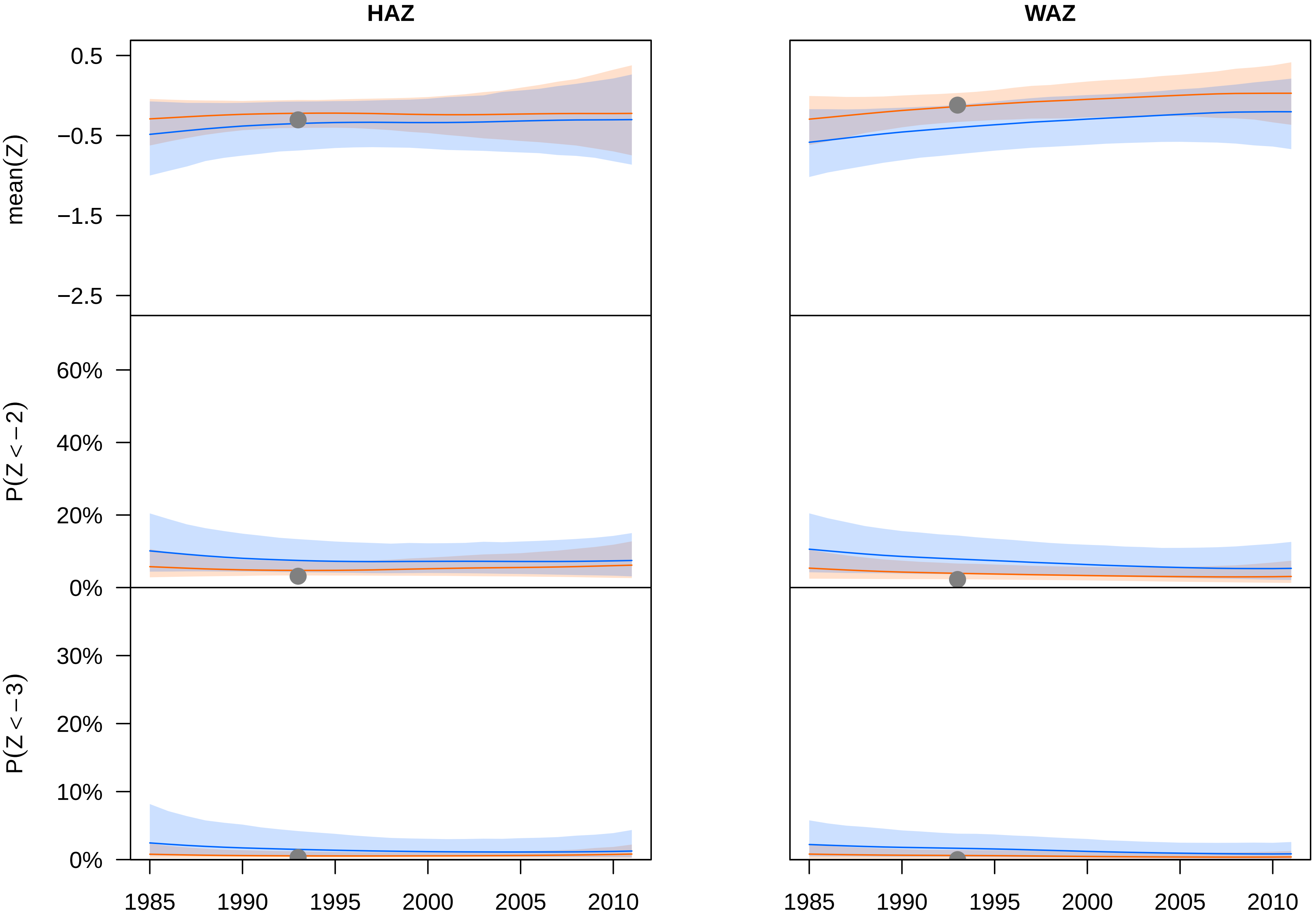

China (Macao SAR)

East and Southeast Asia Region

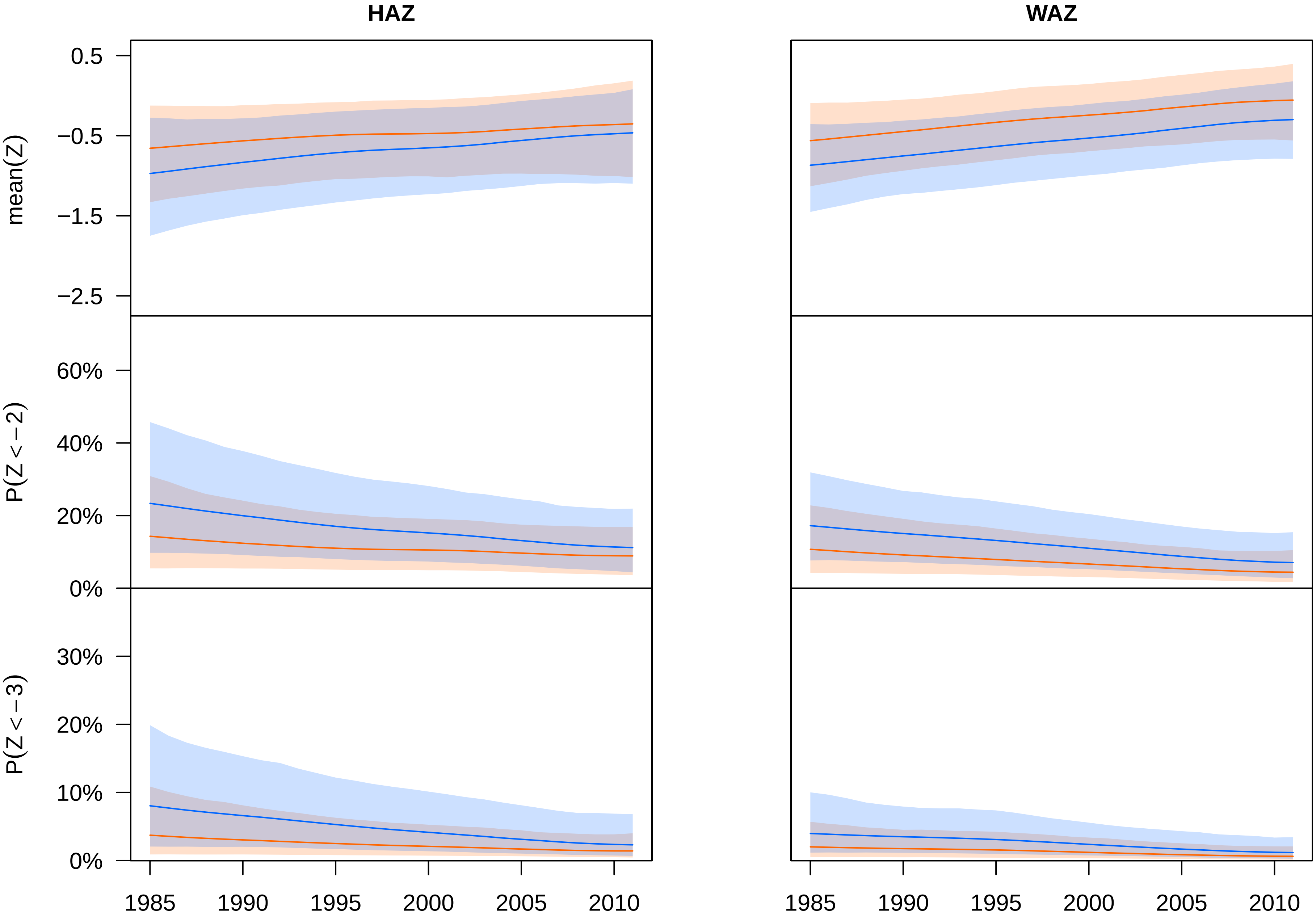

Colombia

Andean and Central Latin America and Caribbean Region

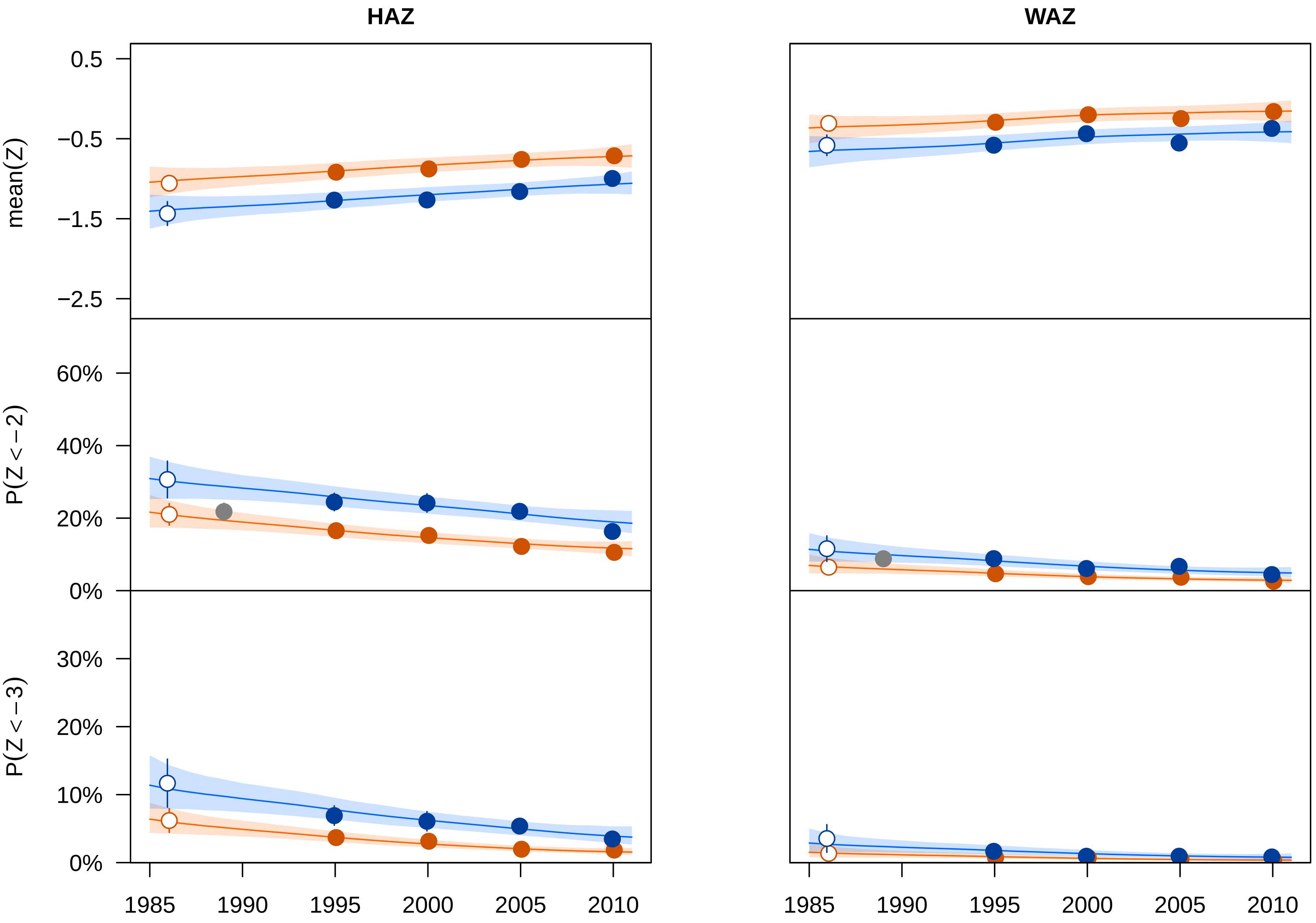

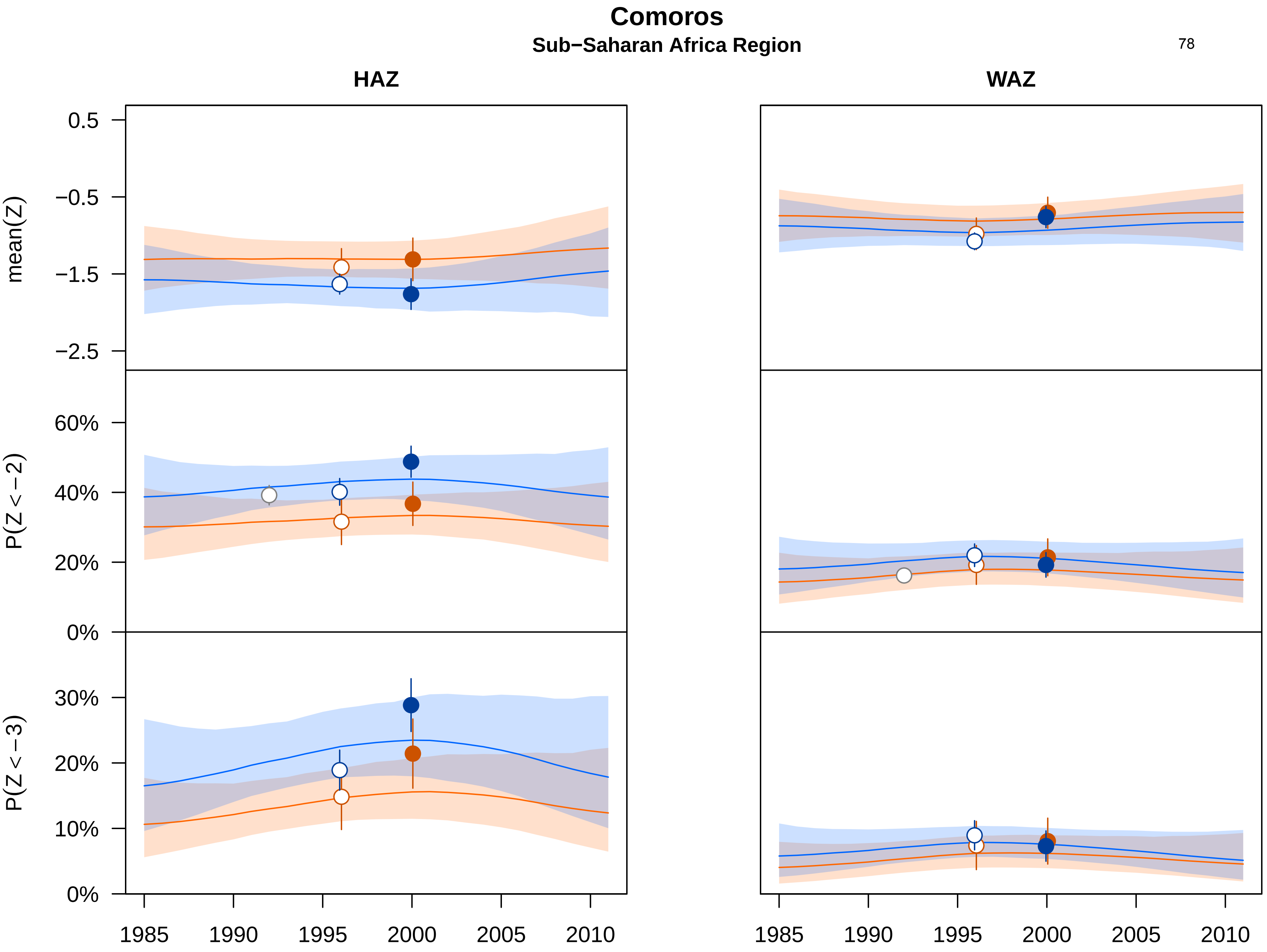
Comoros
Sub−Saharan Africa Region
HAZ
WAZ
mean(Z)
P(Z < −2)
P(Z < −3)
0.5
−0.5
−1.5
−2.5
60%
40%
20%
0%
30%
20%
10%
0%
1985
1990
1995
2000
2005
2010

Congo

Sub−Saharan Africa Region

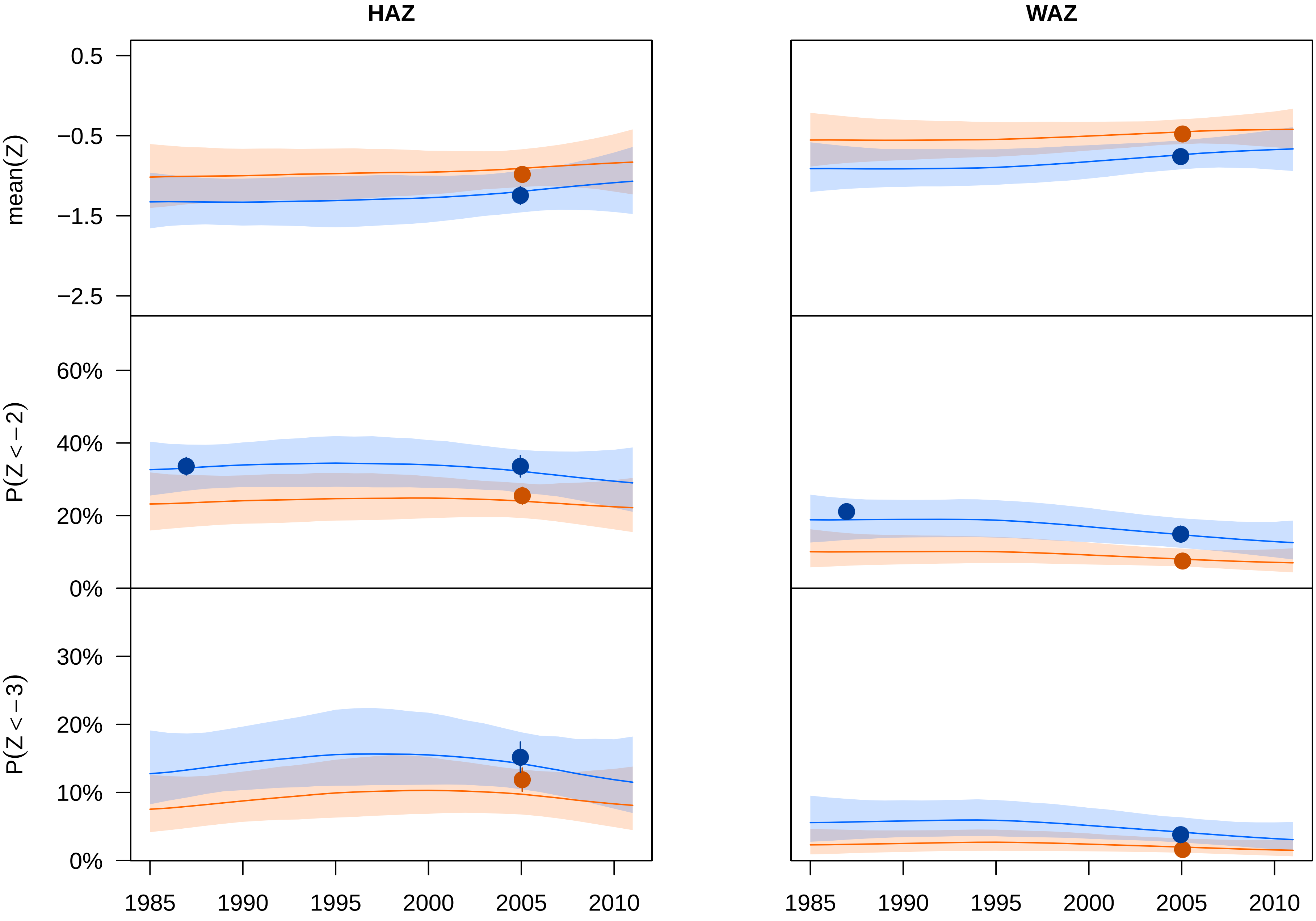

Costa Rica

Andean and Central Latin America and Caribbean Region

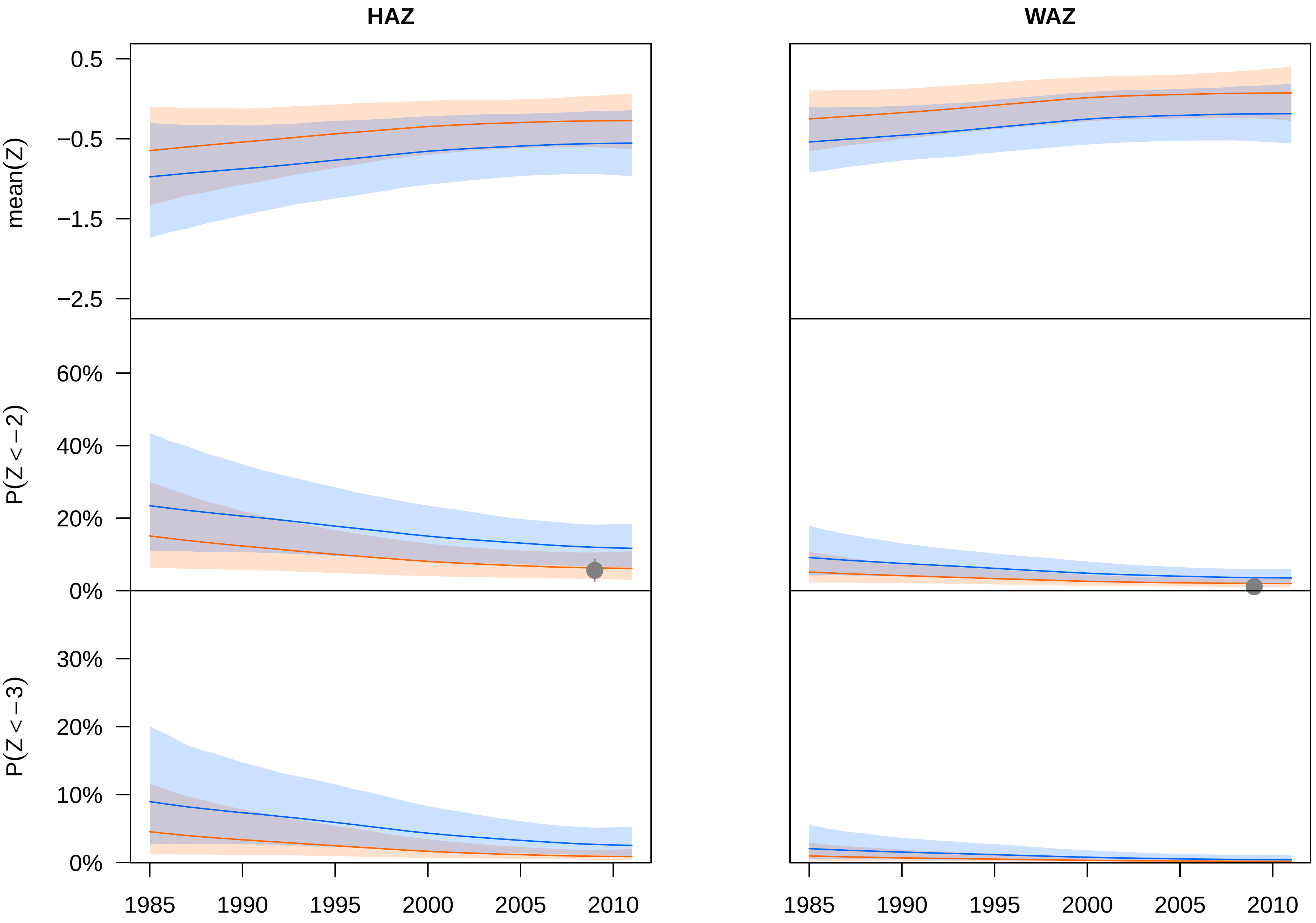

Côte d'Ivoire

Sub−Saharan Africa Region

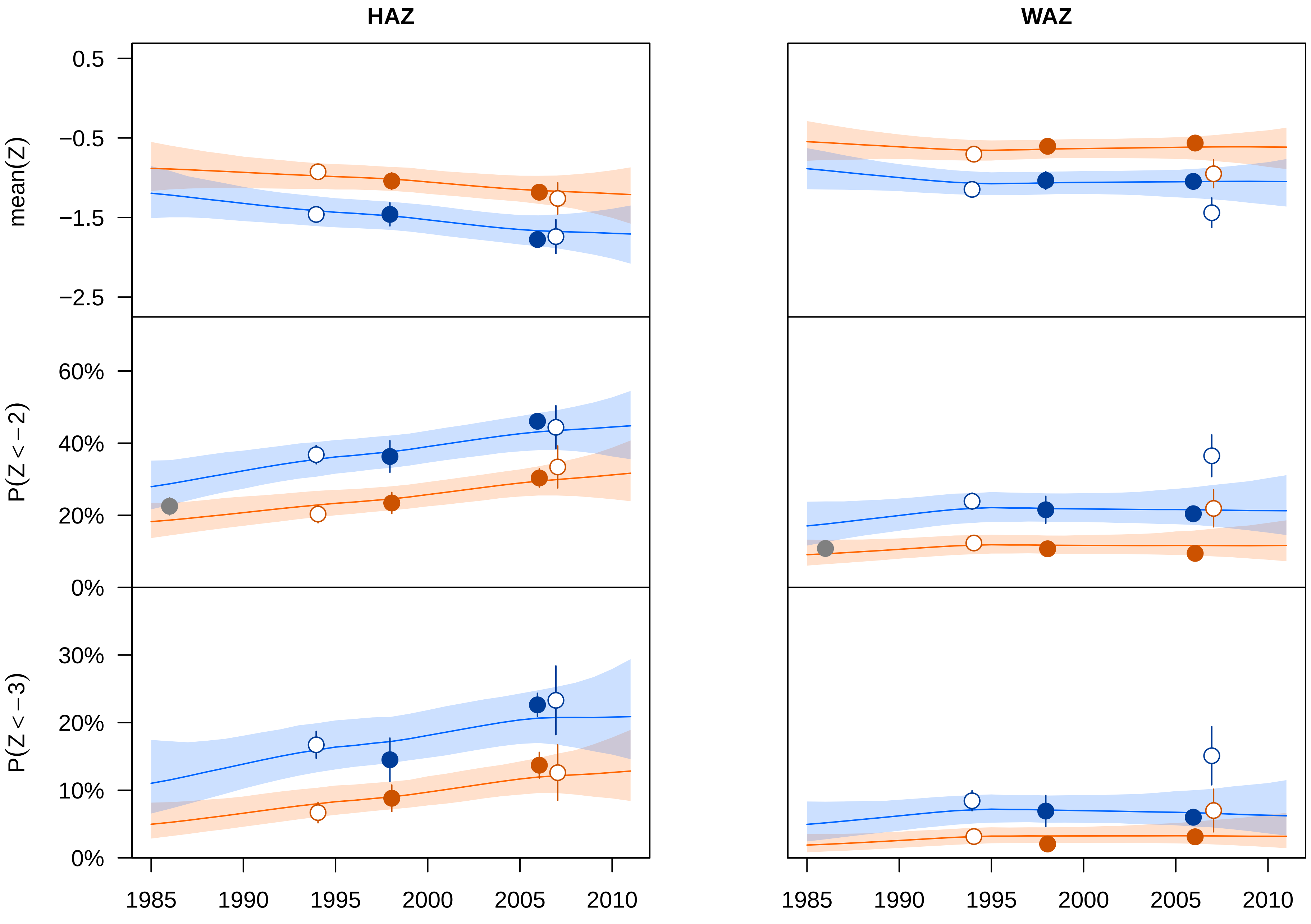

Cuba

Andean and Central Latin America and Caribbean Region

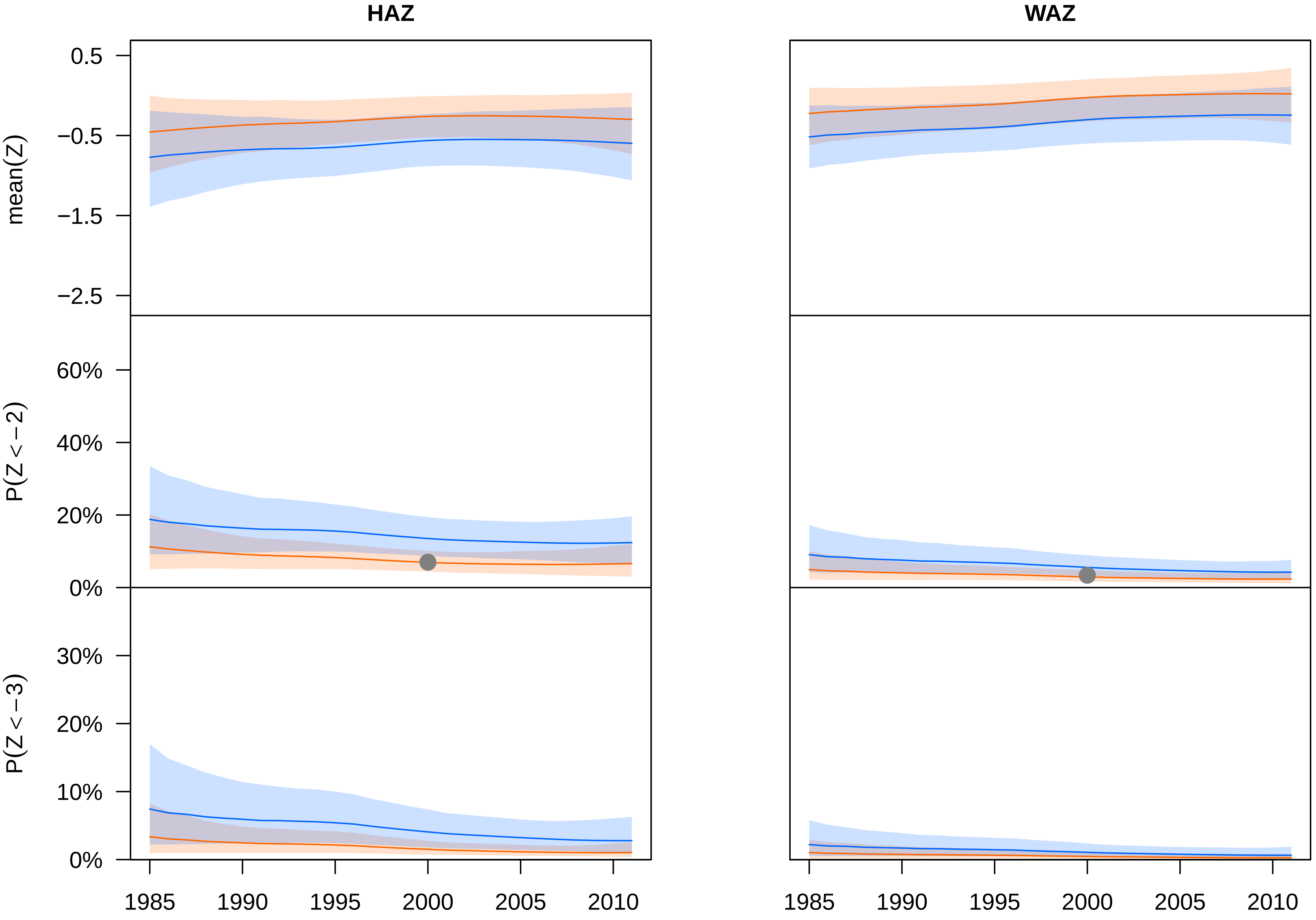

Democratic People's Republic of Korea

East and Southeast Asia Region

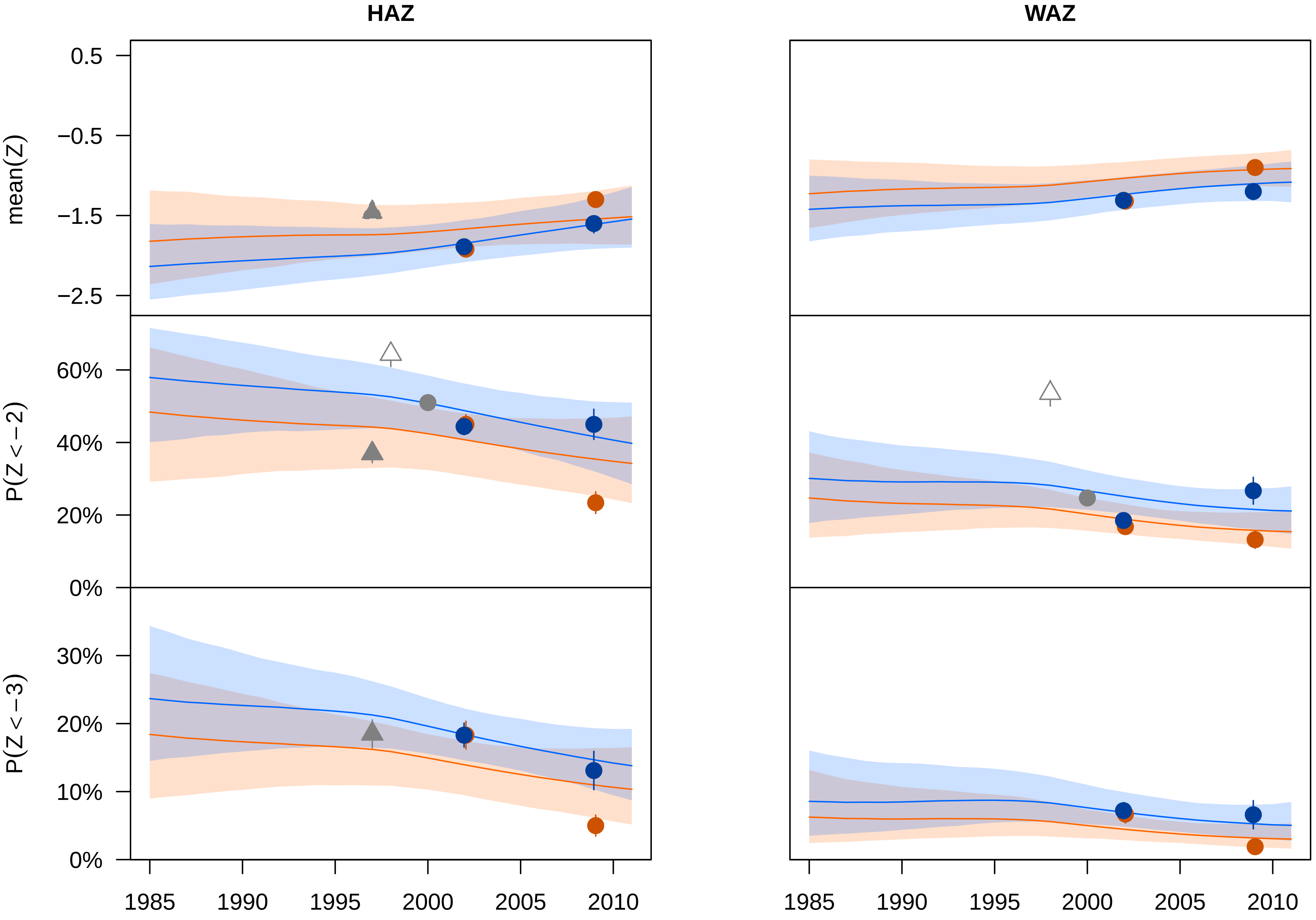

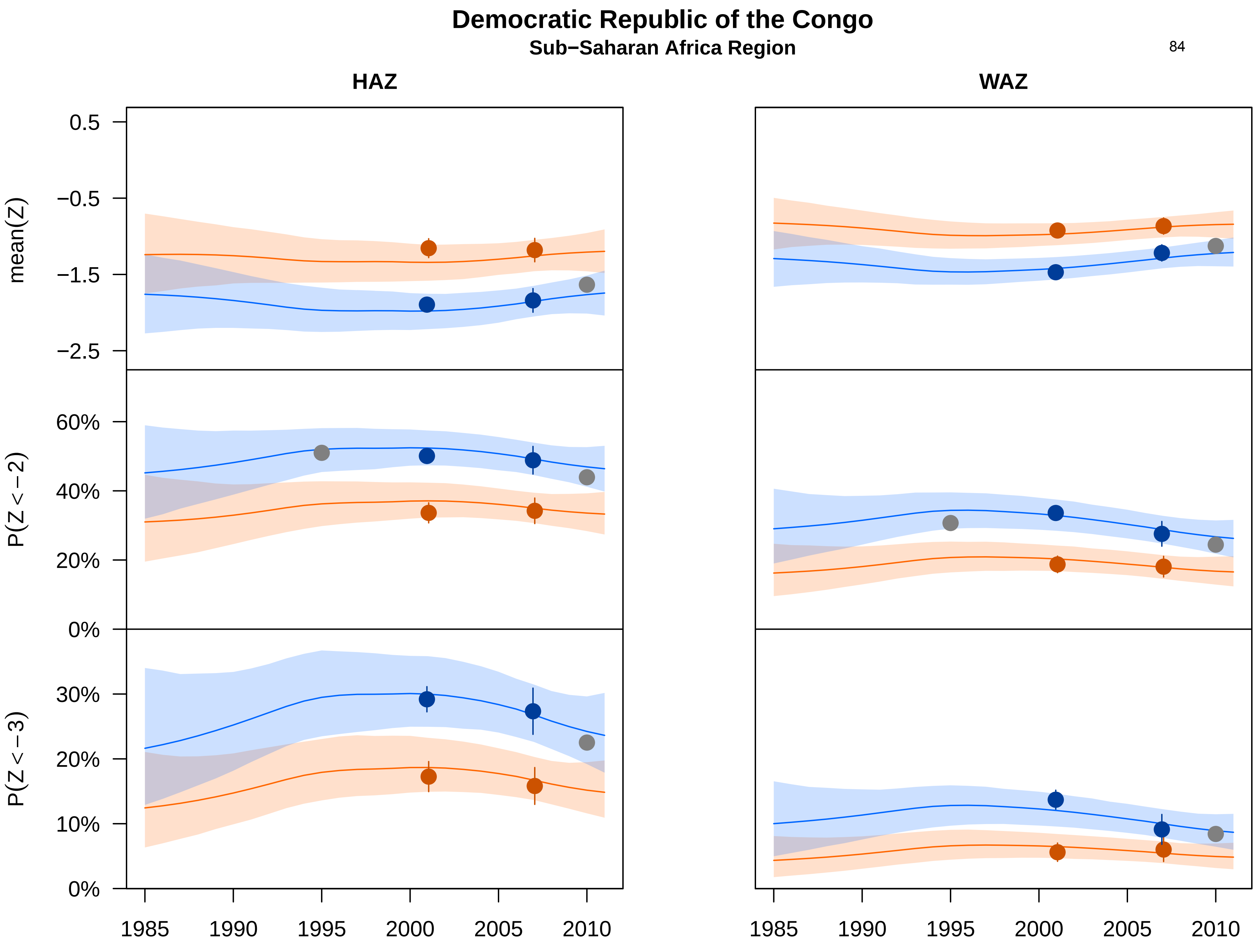
Democratic Republic of the Congo
Sub−Saharan Africa Region
HAZ
WAZ
mean(Z)
P(Z < −2)
P(Z < −3)
0.5
−0.5
−1.5
−2.5
60%
40%
20%
0%
30%
20%
10%
0%
1985
1990
1995
2000
2005
2010

Djibouti

Sub−Saharan Africa Region

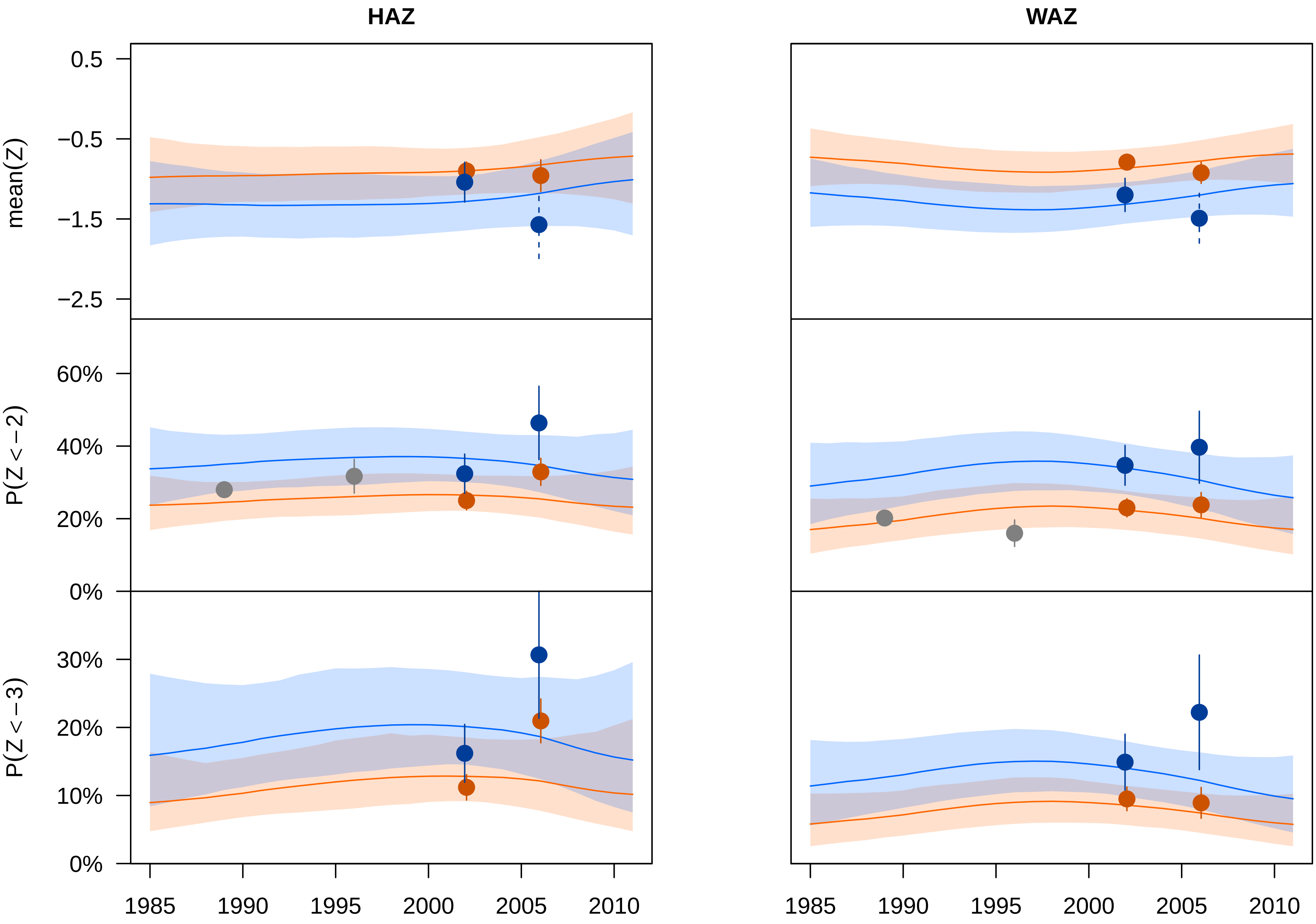

Dominica

Andean and Central Latin America and Caribbean Region

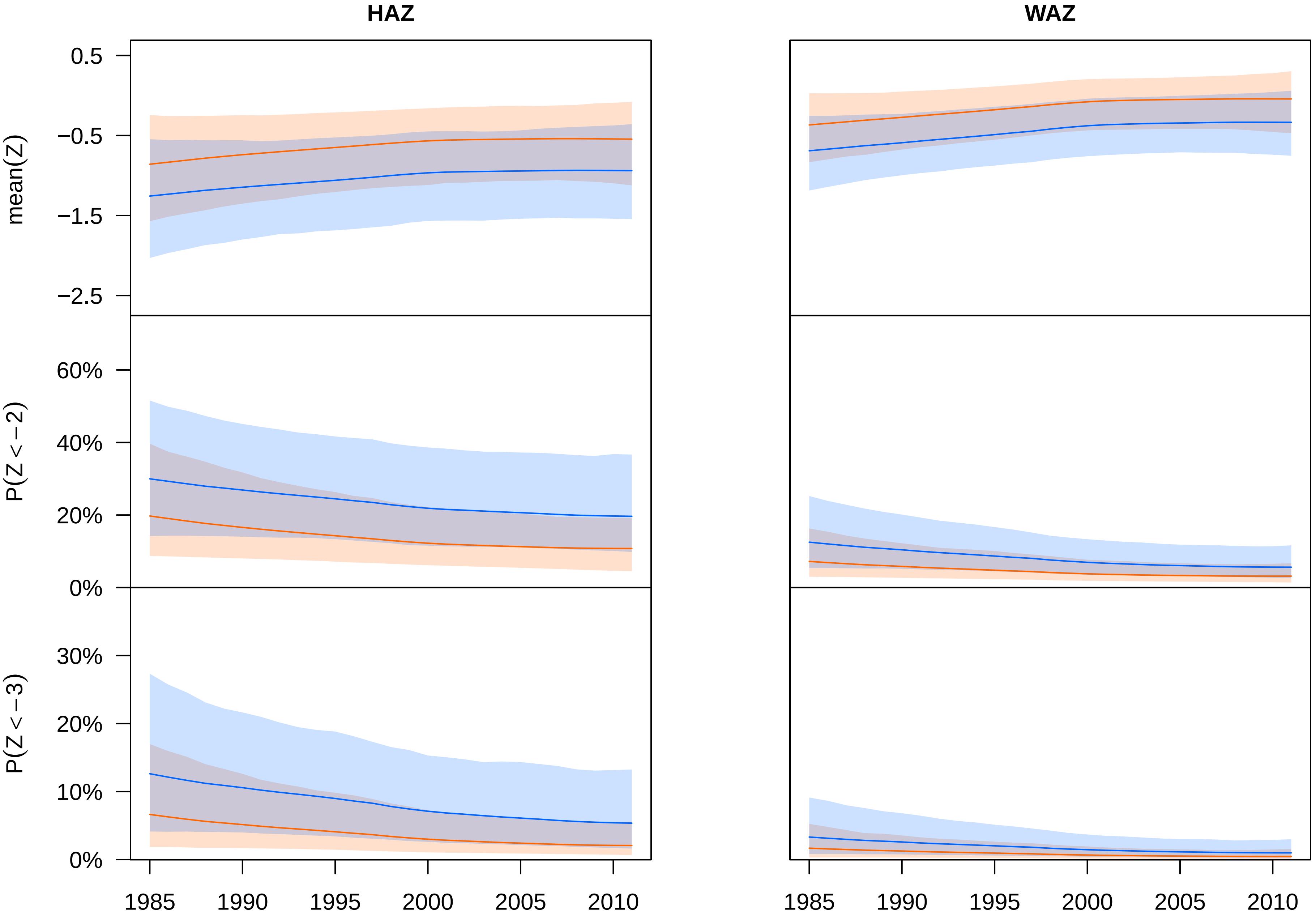

Dominican Republic

Andean and Central Latin America and Caribbean Region

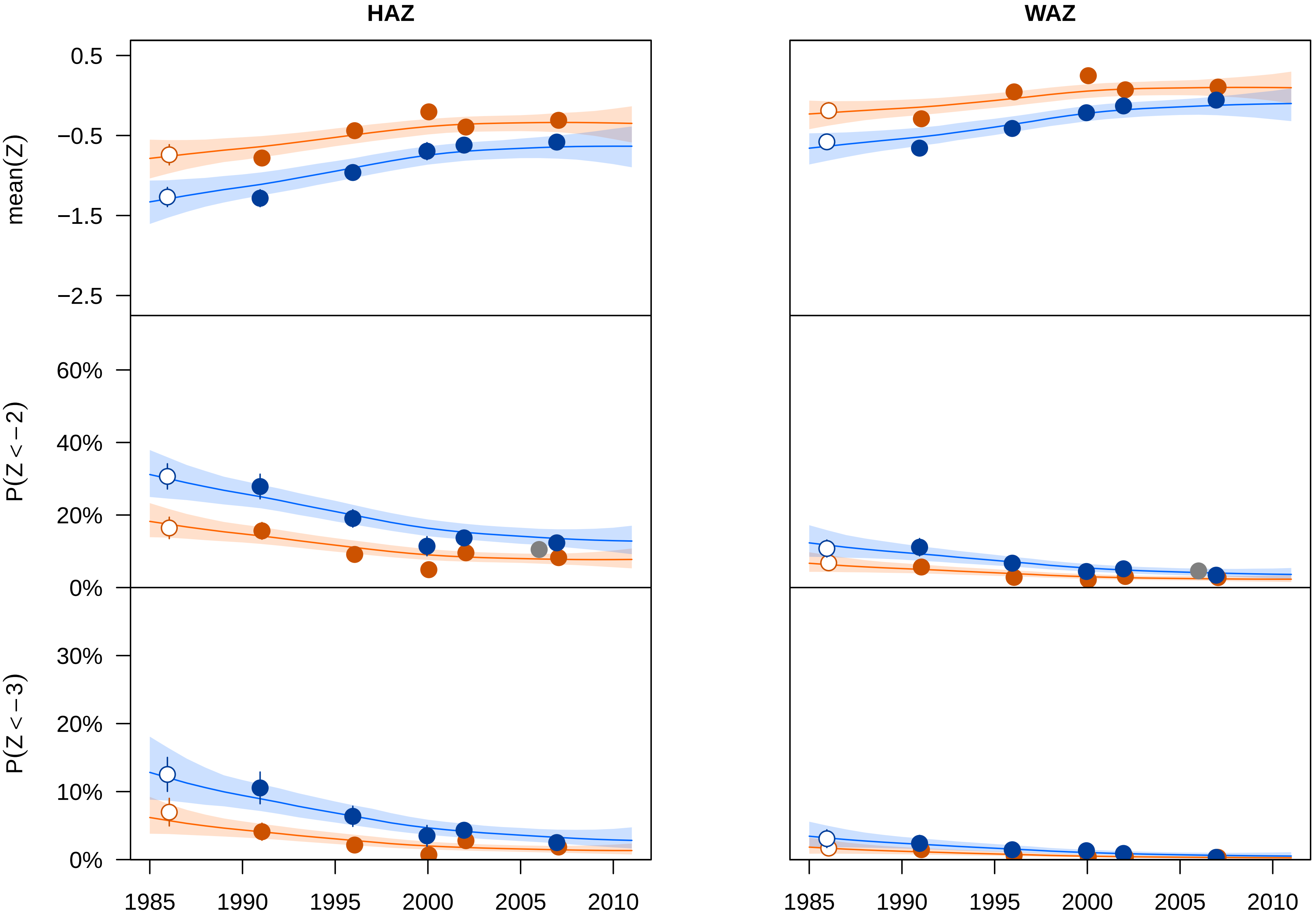

Ecuador

Andean and Central Latin America and Caribbean Region

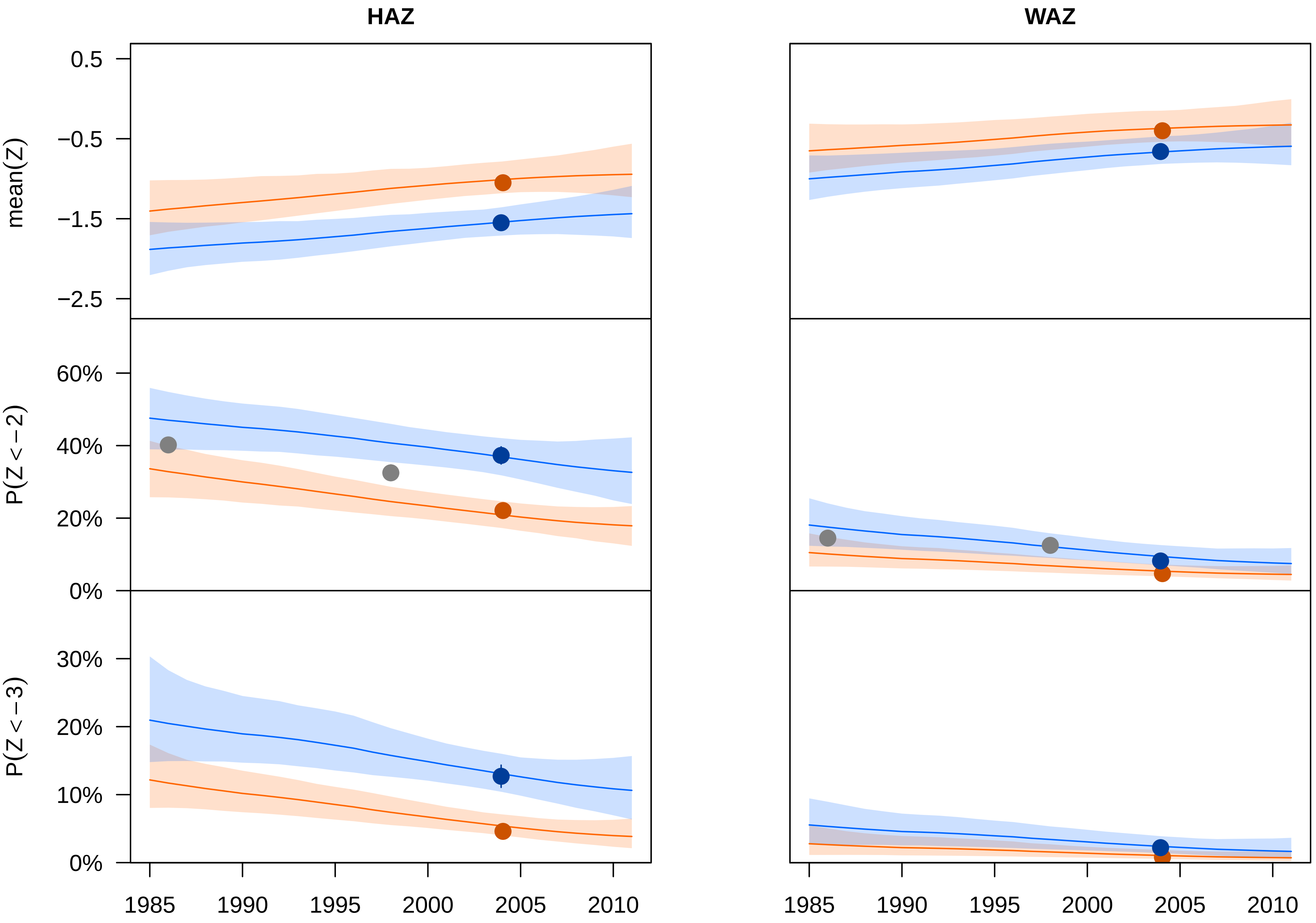

Egypt

Central Asia, Middle East, and North Africa Region

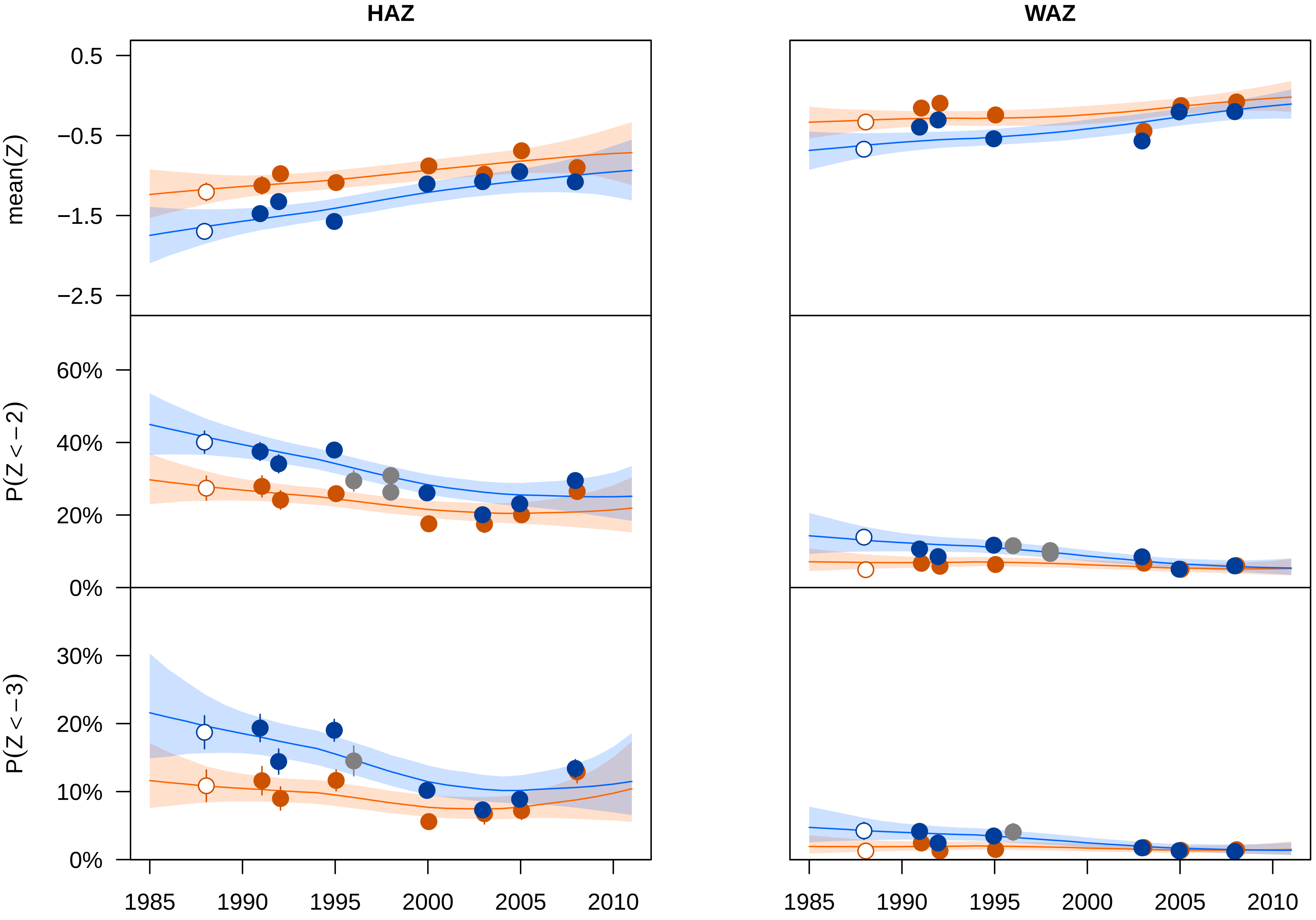

El Salvador

Andean and Central Latin America and Caribbean Region

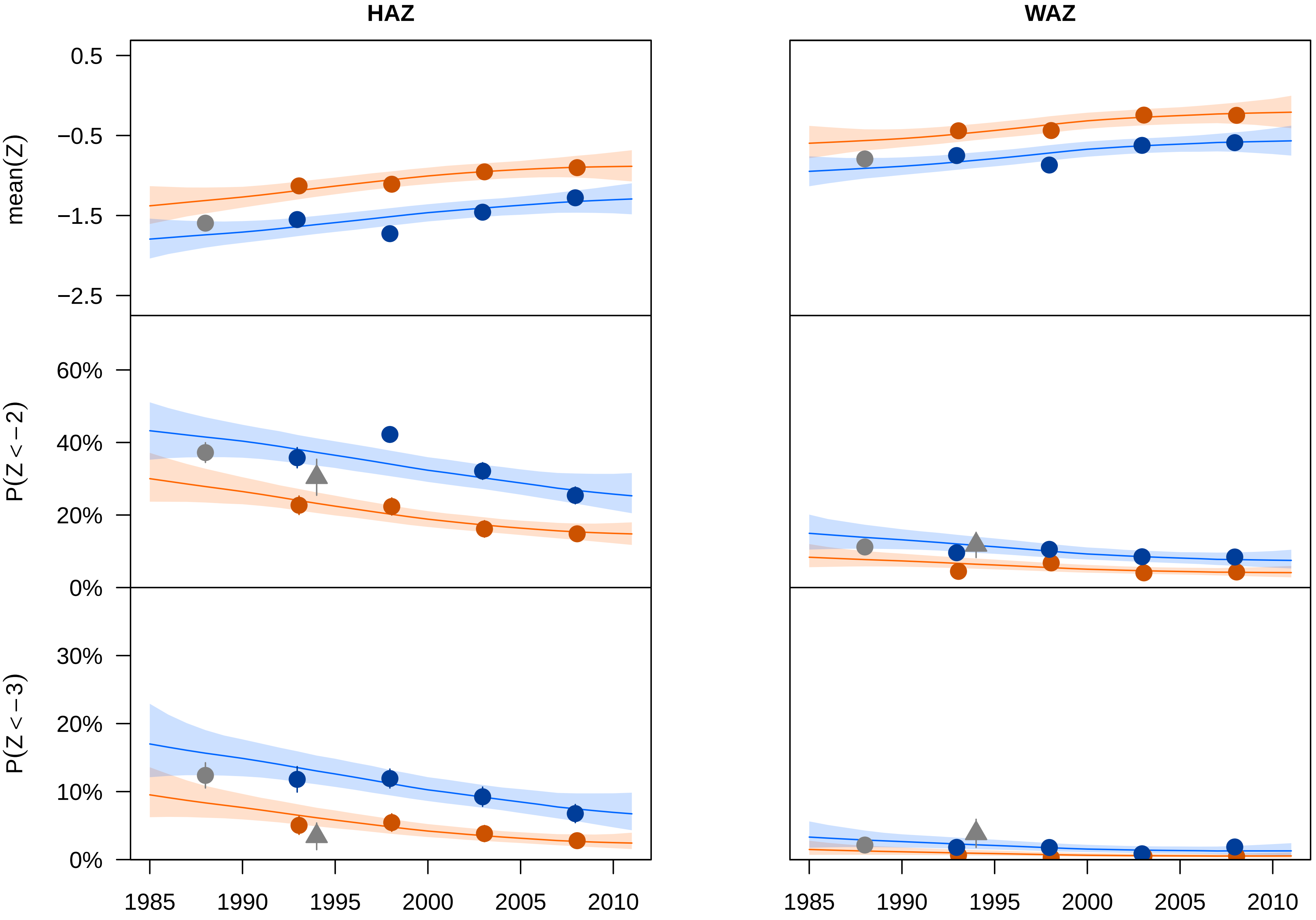

Equatorial Guinea

Sub−Saharan Africa Region

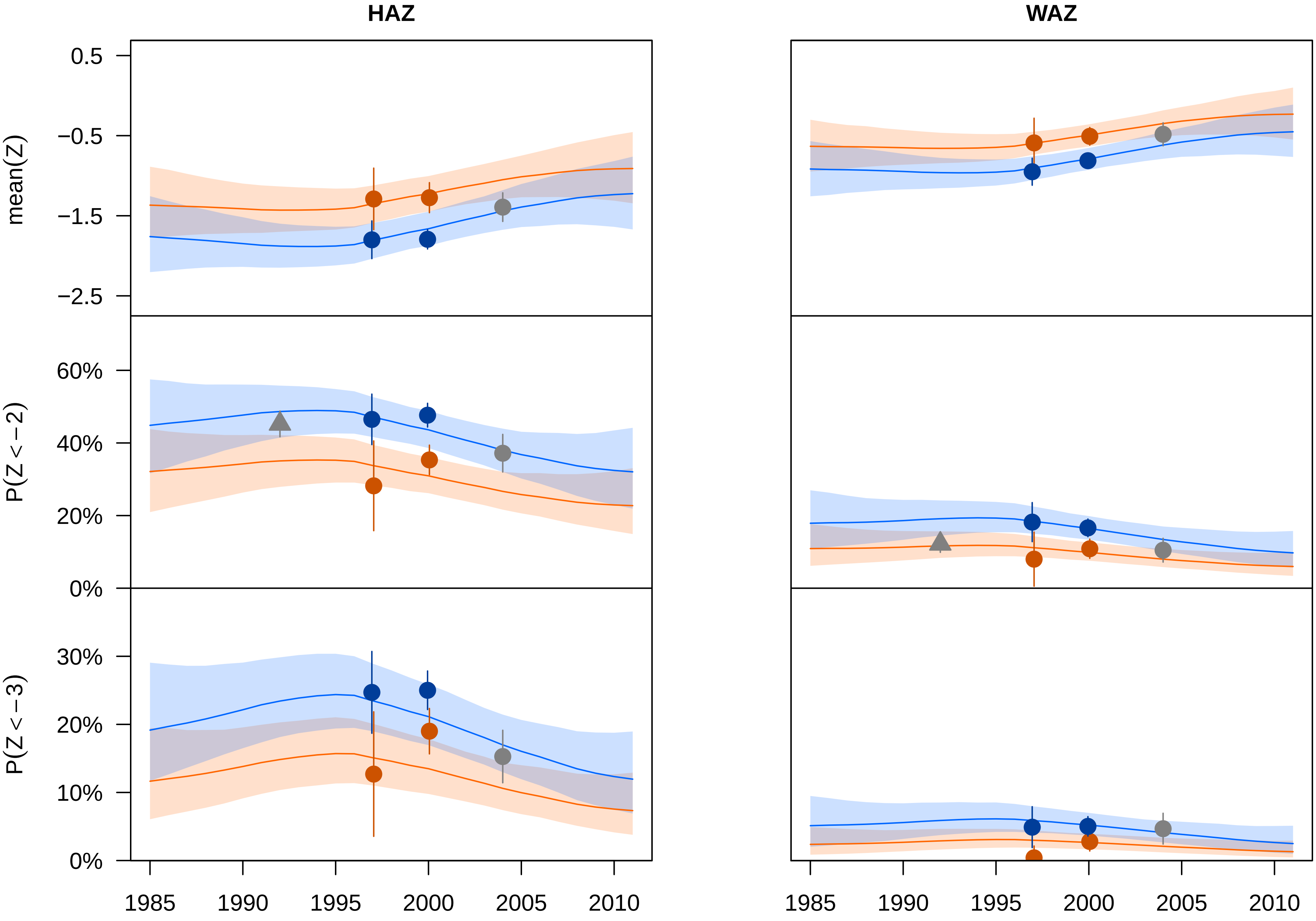

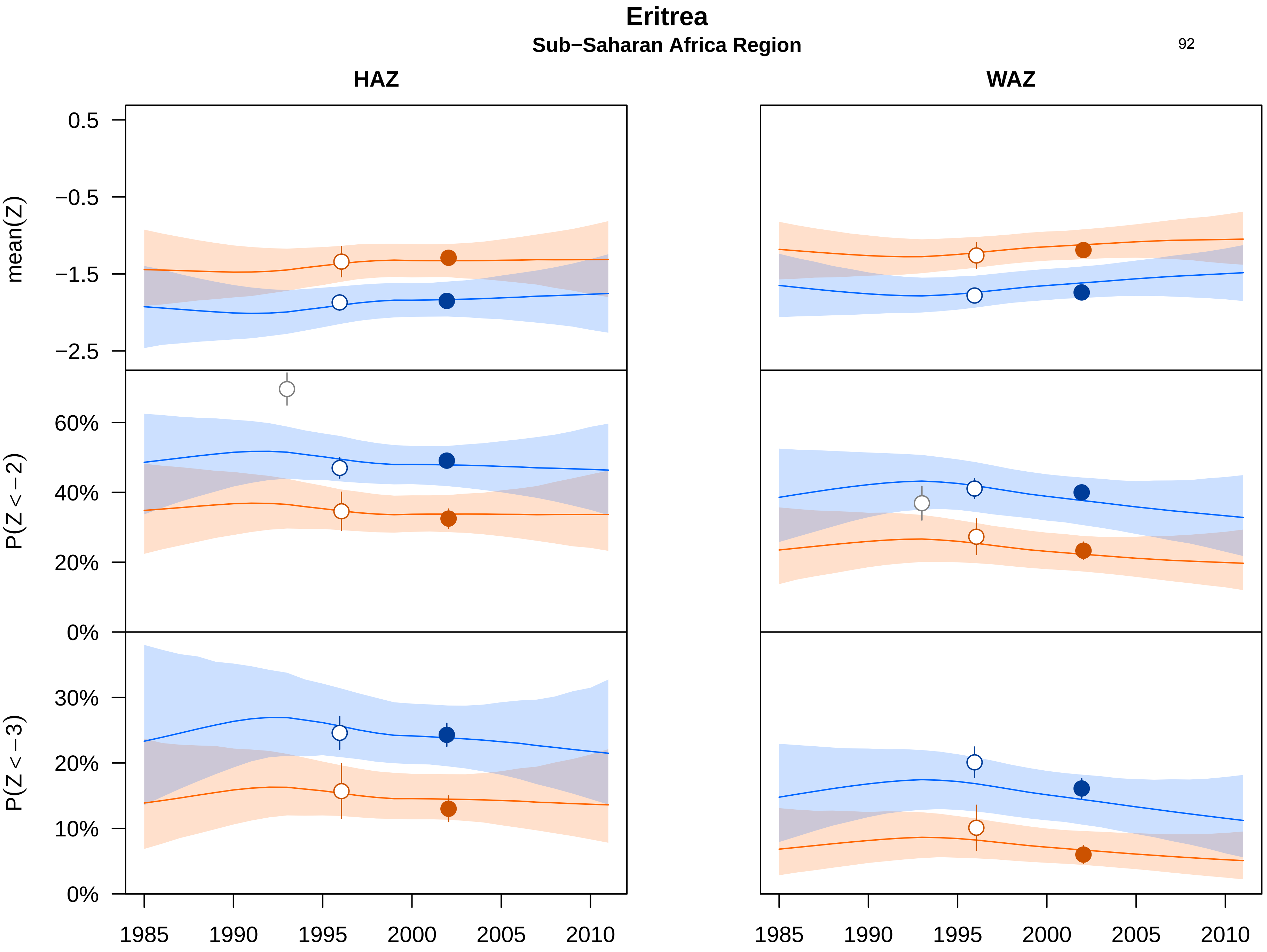
Eritrea
Sub−Saharan Africa Region
HAZ
WAZ
mean(Z)
P(Z < −2)
P(Z < −3)
0.5
−0.5
−1.5
−2.5
60%
40%
20%
0%
30%
20%
10%
0%
1985
1990
1995
2000
2005
2010

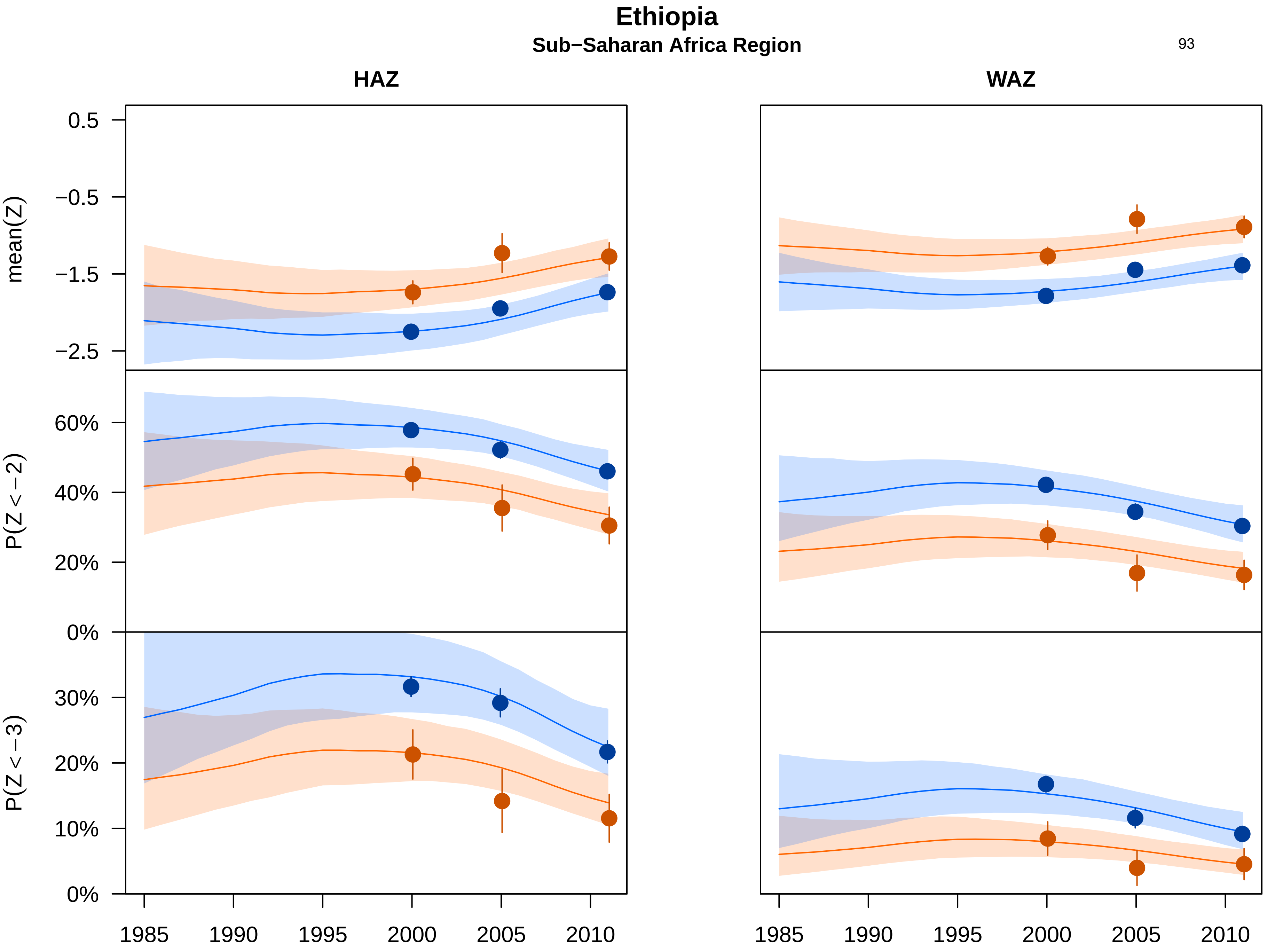
Ethiopia
Sub−Saharan Africa Region
HAZ
WAZ
mean(Z)
P(Z < −2)
P(Z < −3)
0.5
−0.5
−1.5
−2.5
60%
40%
20%
0%
30%
20%
10%
0%
1985
1990
1995
2000
2005
2010

Fiji

Oceania Region

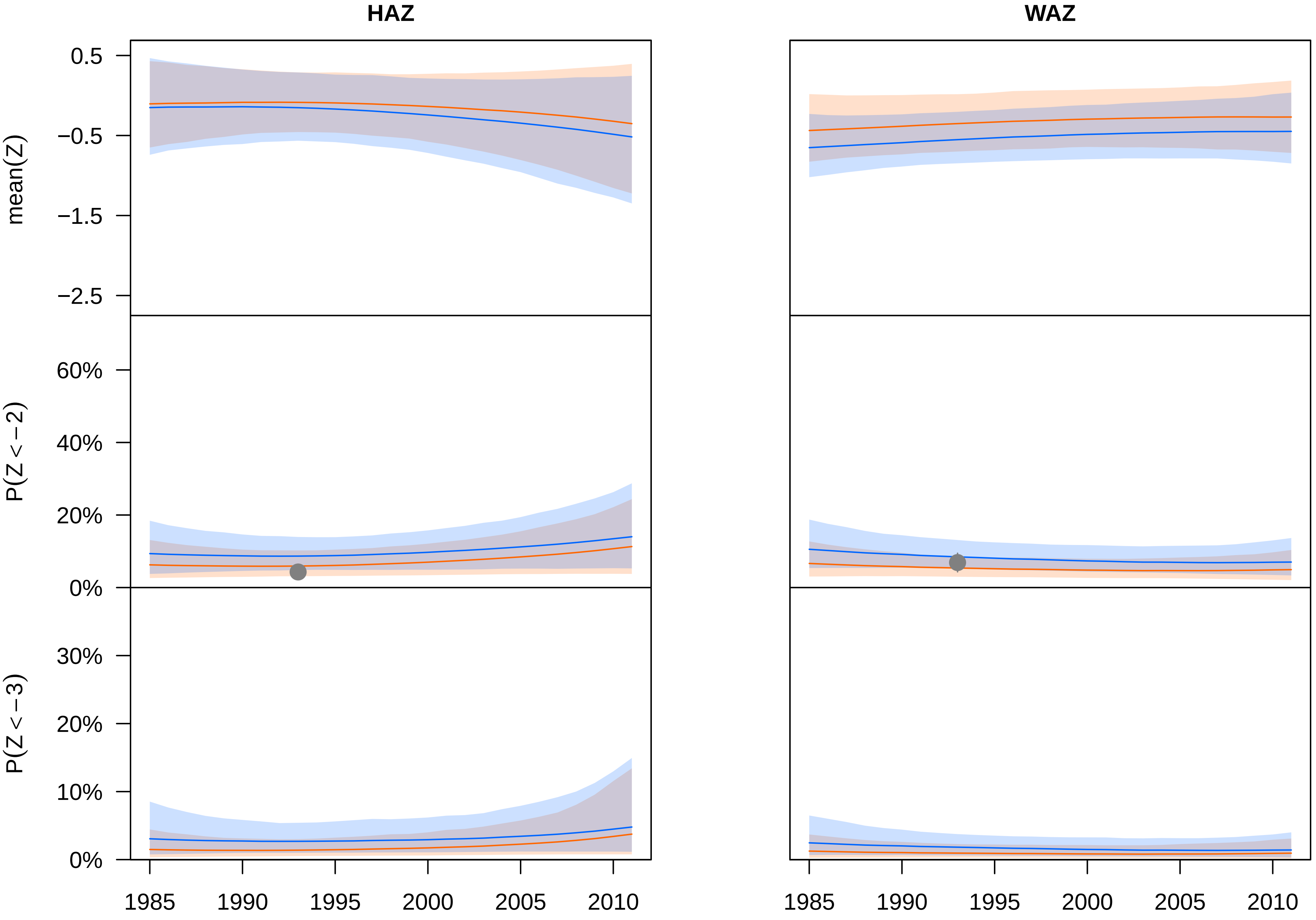

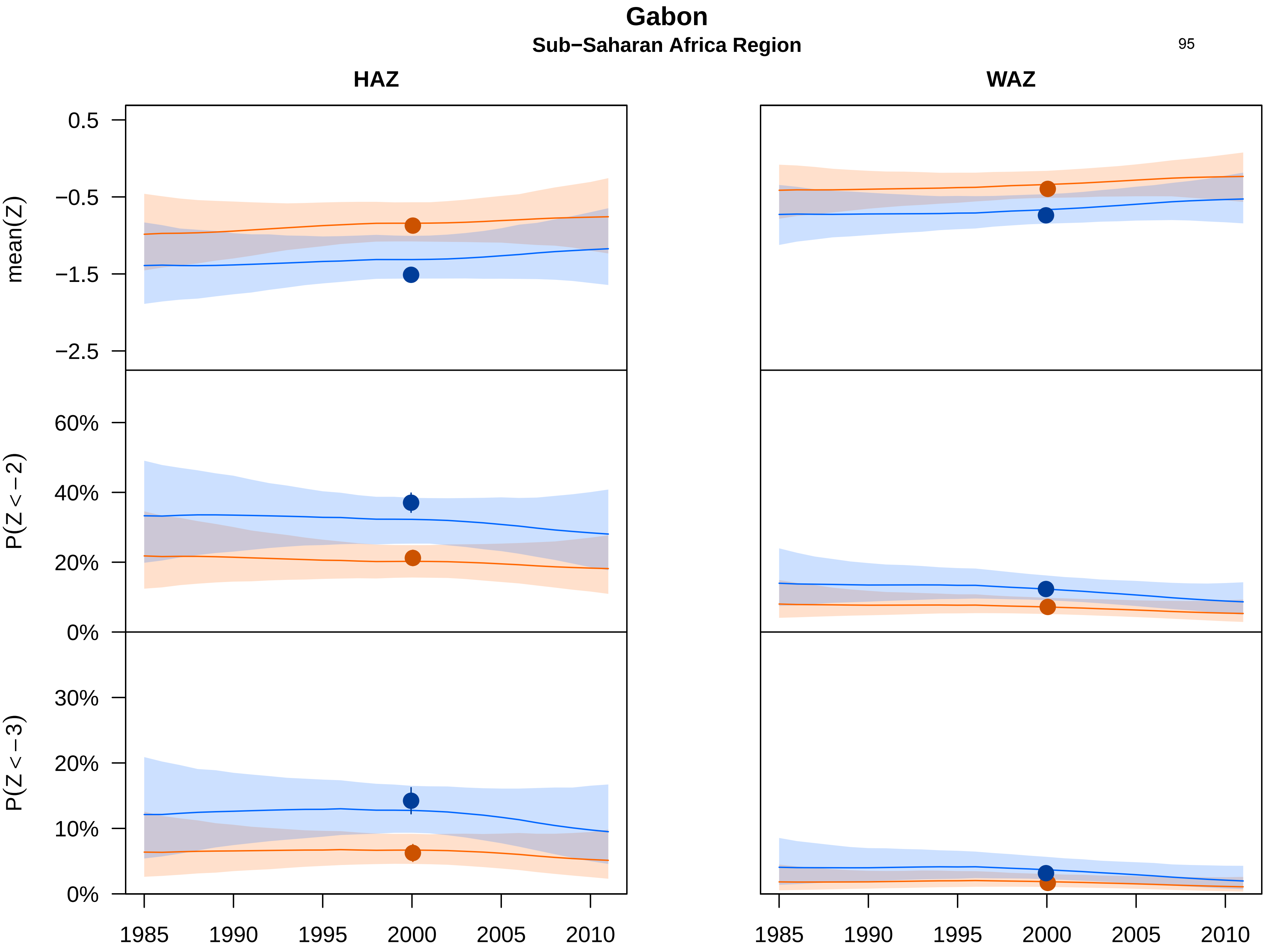
Gabon
Sub−Saharan Africa Region
HAZ
WAZ
mean(Z)
P(Z < −2)
P(Z < −3)
0.5
−0.5
−1.5
−2.5
60%
40%
20%
0%
30%
20%
10%
0%
1985
1990
1995
2000
2005
2010

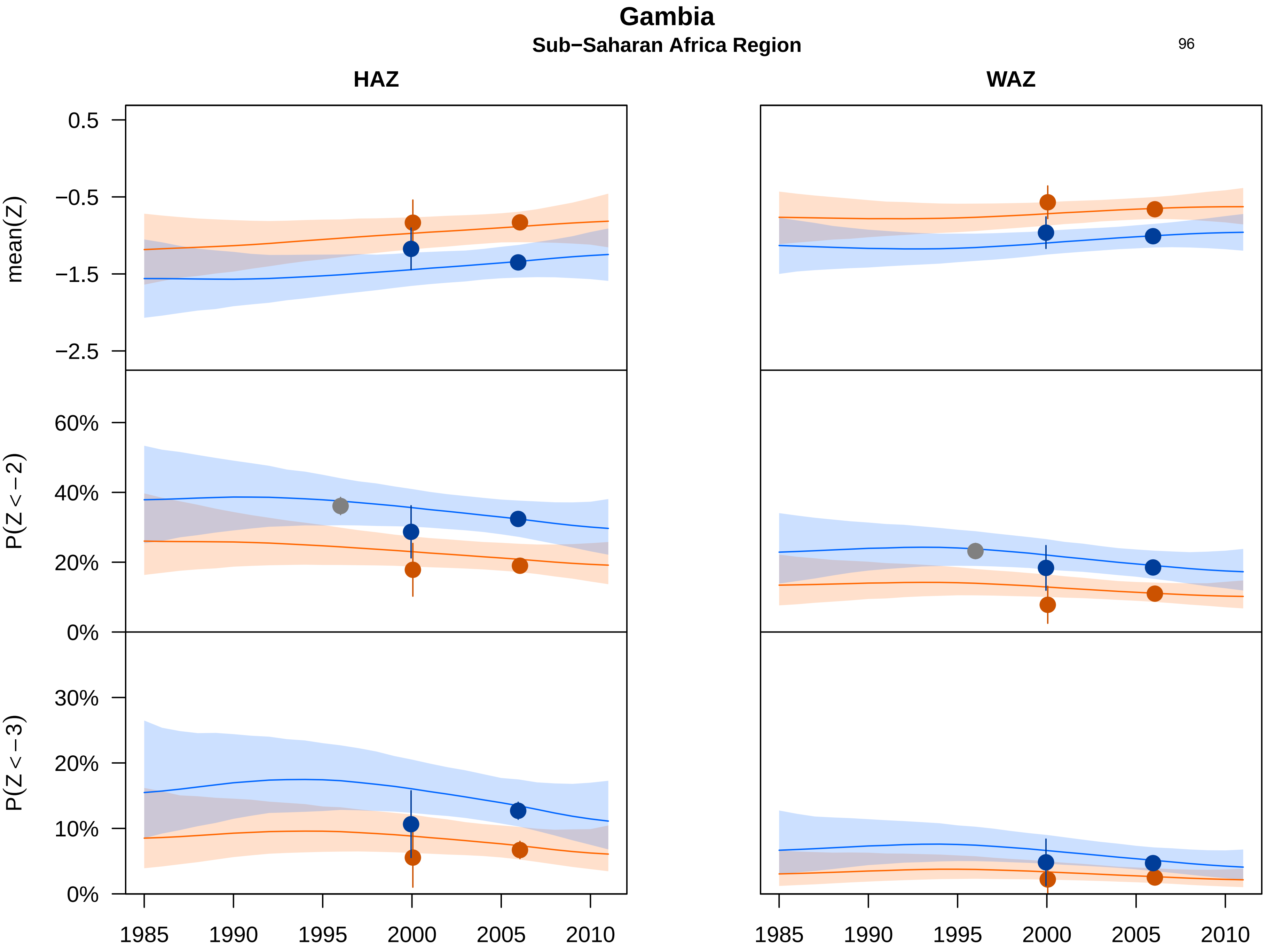
Gambia
Sub−Saharan Africa Region
HAZ
WAZ
mean(Z)
P(Z < −2)
P(Z < −3)
0.5
−0.5
−1.5
−2.5
60%
40%
20%
0%
30%
20%
10%
0%
1985
1990
1995
2000
2005
2010

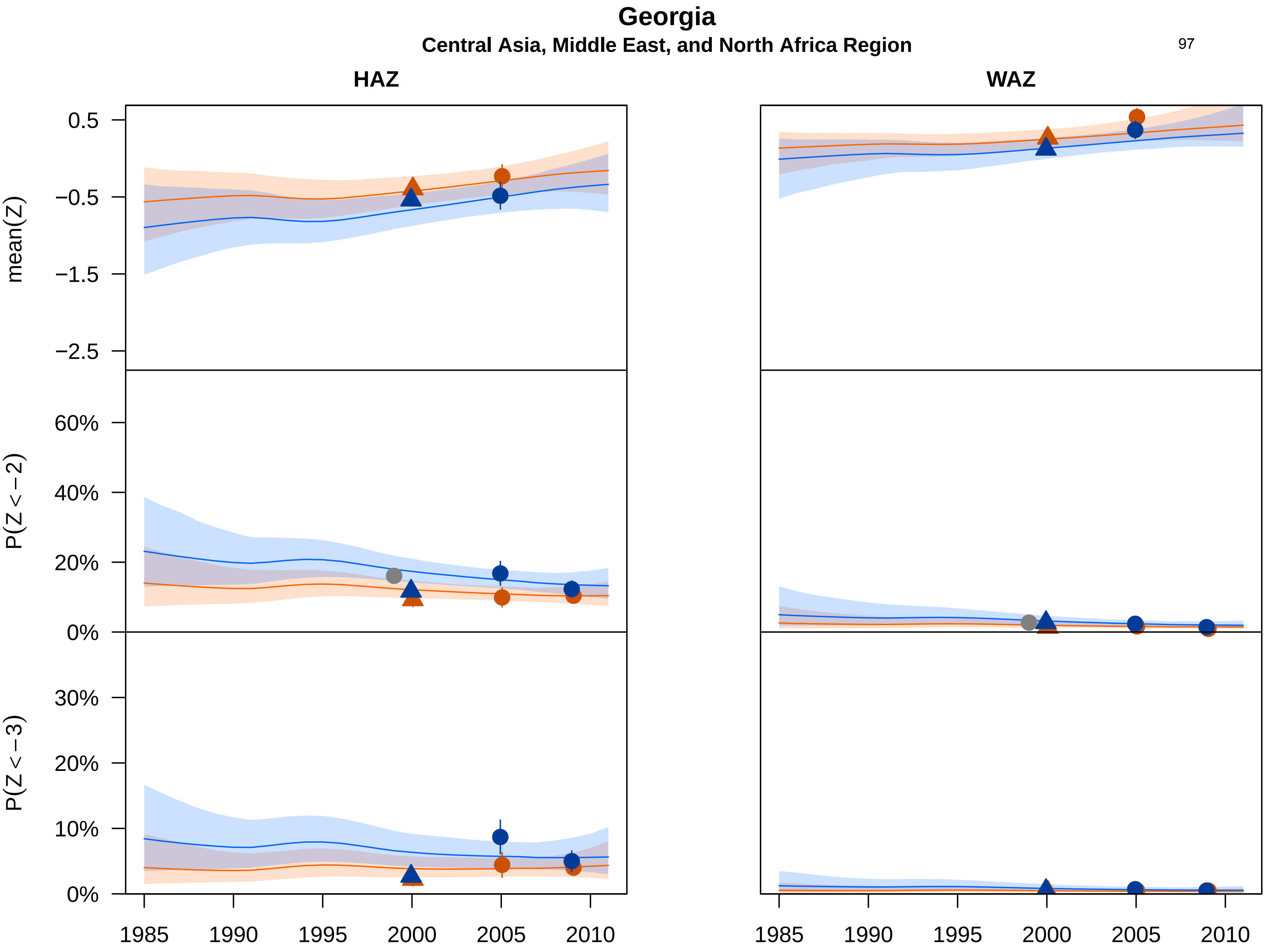
Georgia
Central Asia, Middle East, and North Africa Region
HAZ
WAZ
mean(Z)
P(Z < −2)
P(Z < −3)
0.5
−0.5
−1.5
−2.5
60%
40%
20%
0%
30%
20%
10%
0%
1985
1990
1995
2000
2005
2010

Ghana

Sub−Saharan Africa Region

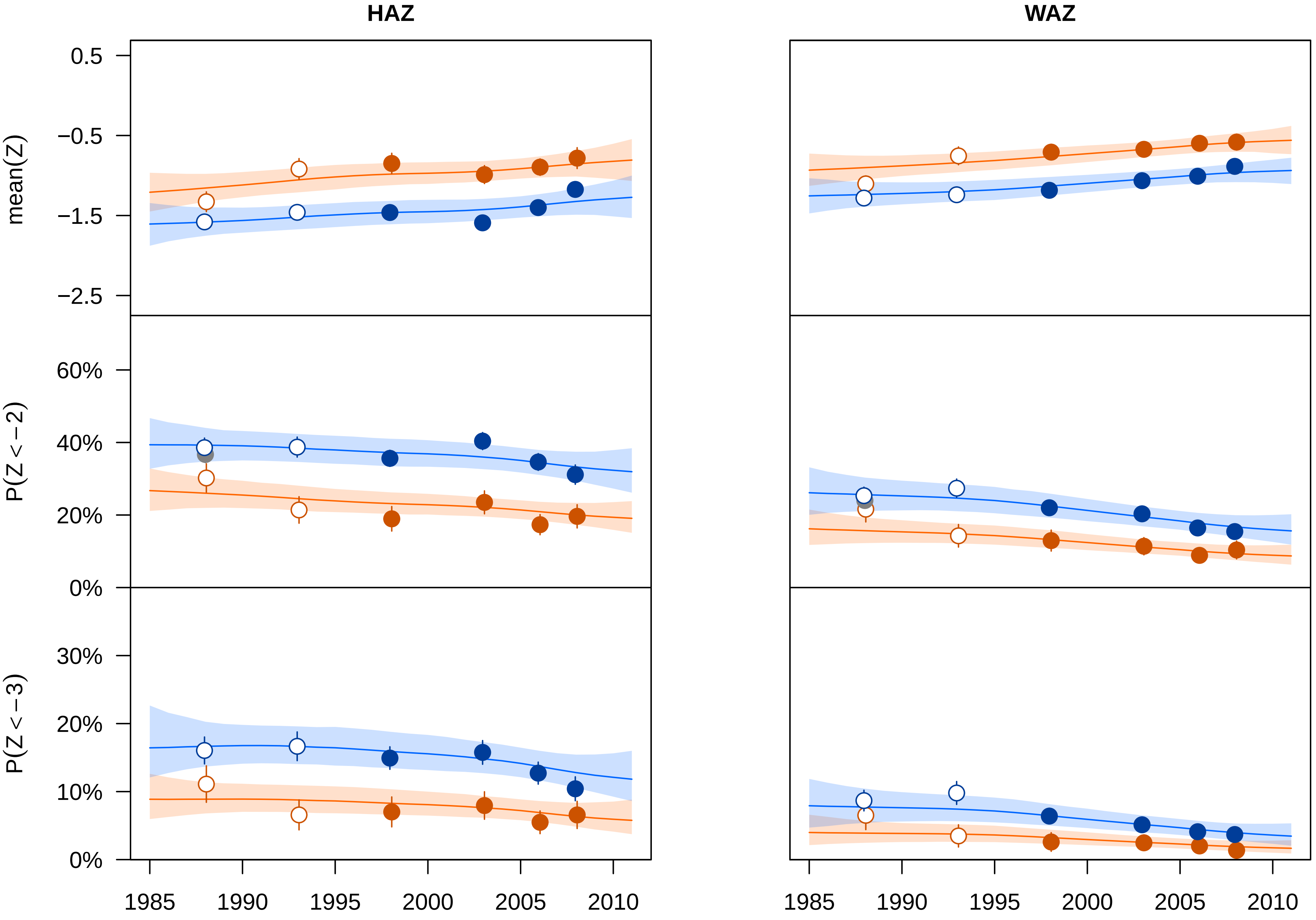

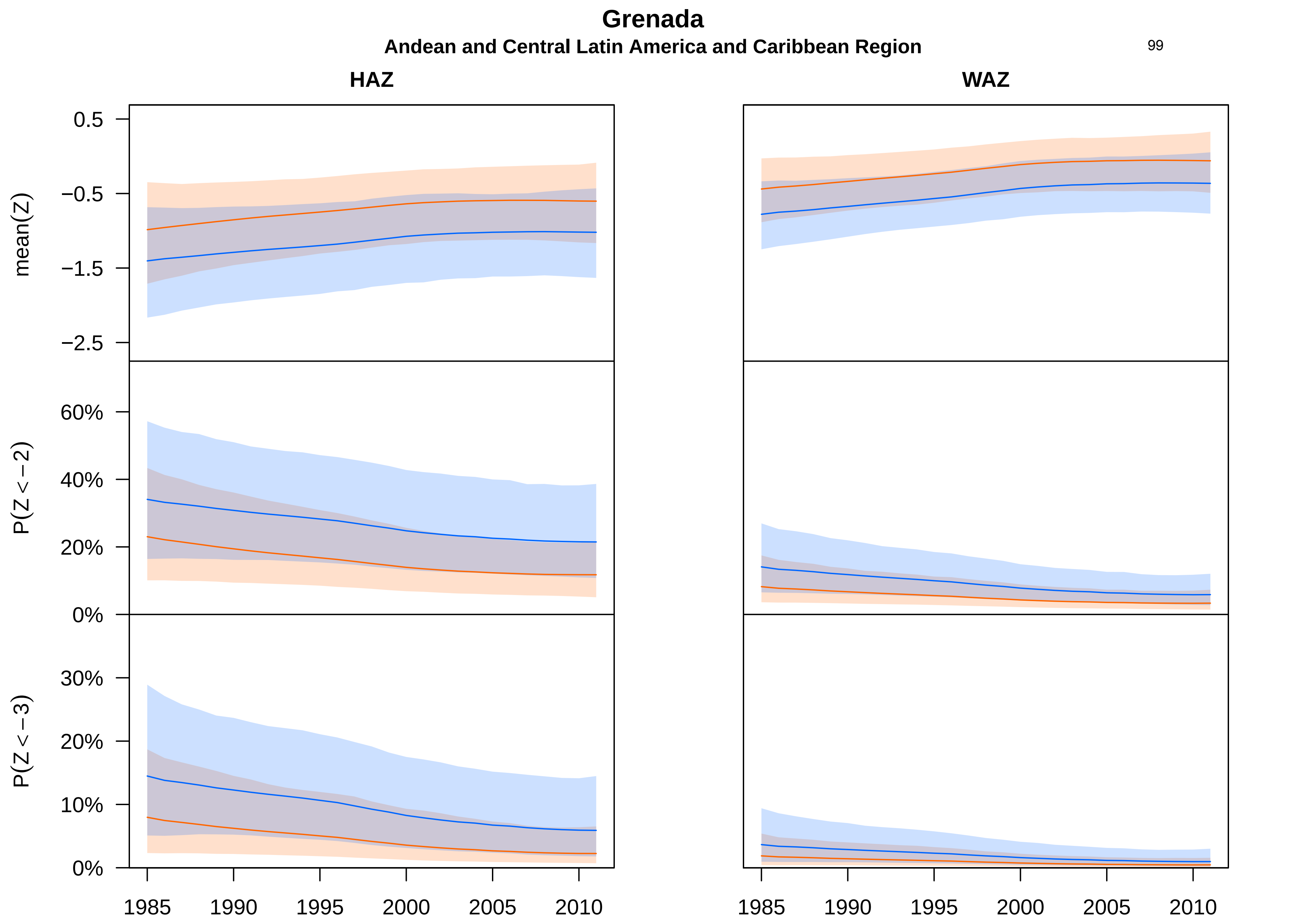
Grenada
Andean and Central Latin America and Caribbean Region
HAZ
WAZ
mean(Z)
P(Z < −2)
P(Z < −3)
0.5
−0.5
−1.5
−2.5
60%
40%
20%
0%
30%
20%
10%
0%
1985
1990
1995
2000
2005
2010

Guatemala

Andean and Central Latin America and Caribbean Region

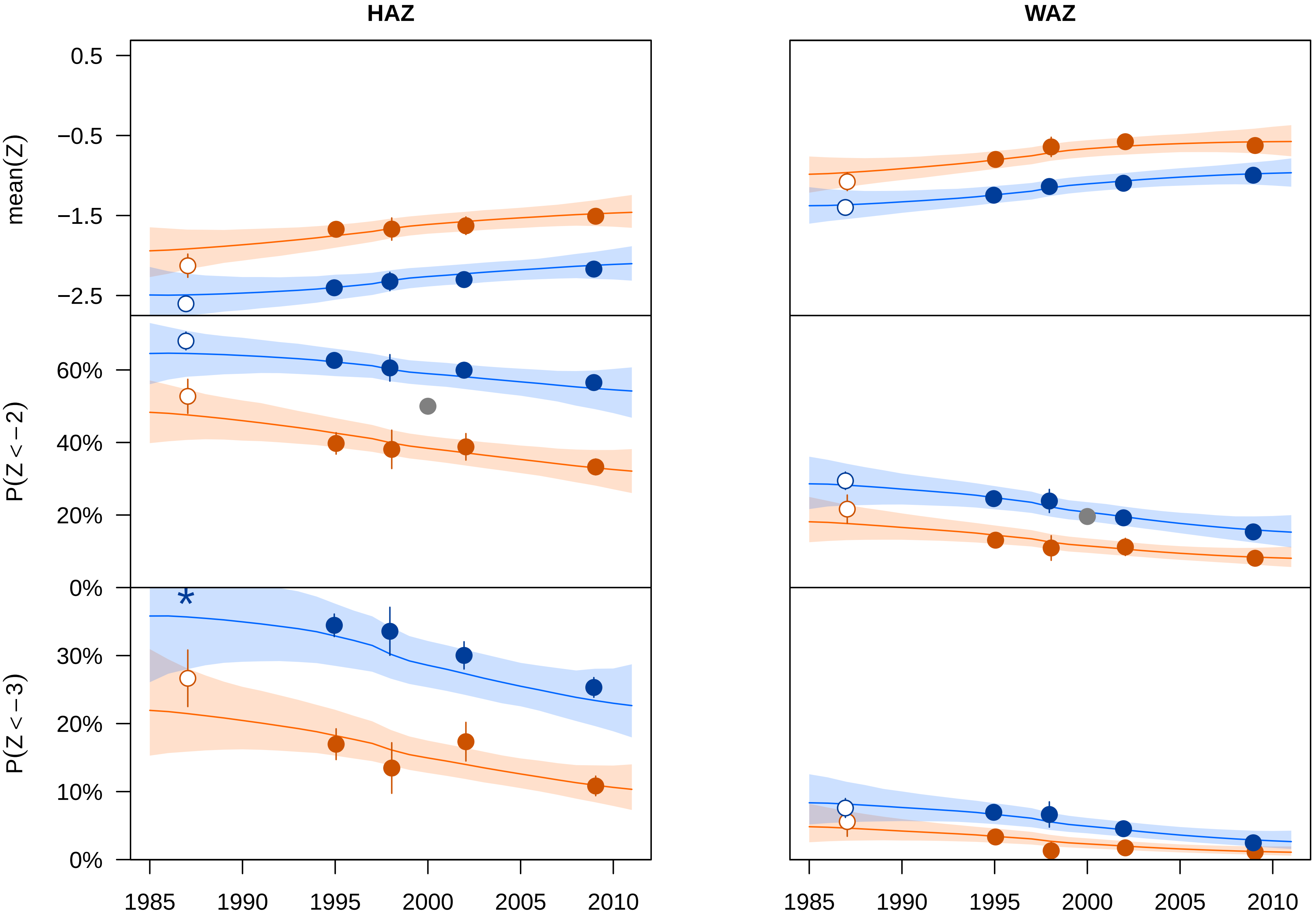

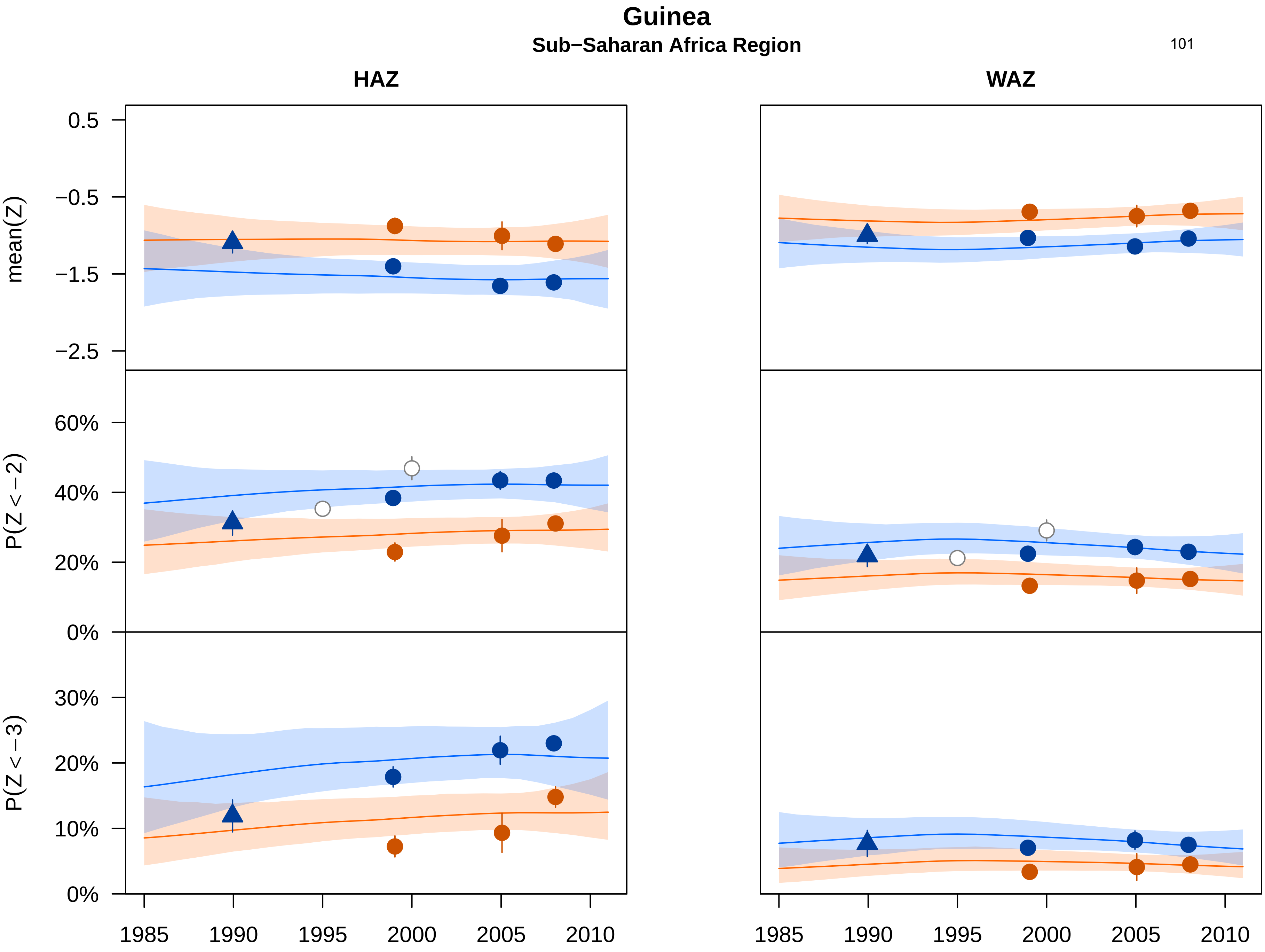
Guinea
Sub−Saharan Africa Region
HAZ
WAZ
mean(Z)
P(Z < −2)
P(Z < −3)
0.5
−0.5
−1.5
−2.5
60%
40%
20%
0%
30%
20%
10%
0%
1985
1990
1995
2000
2005
2010

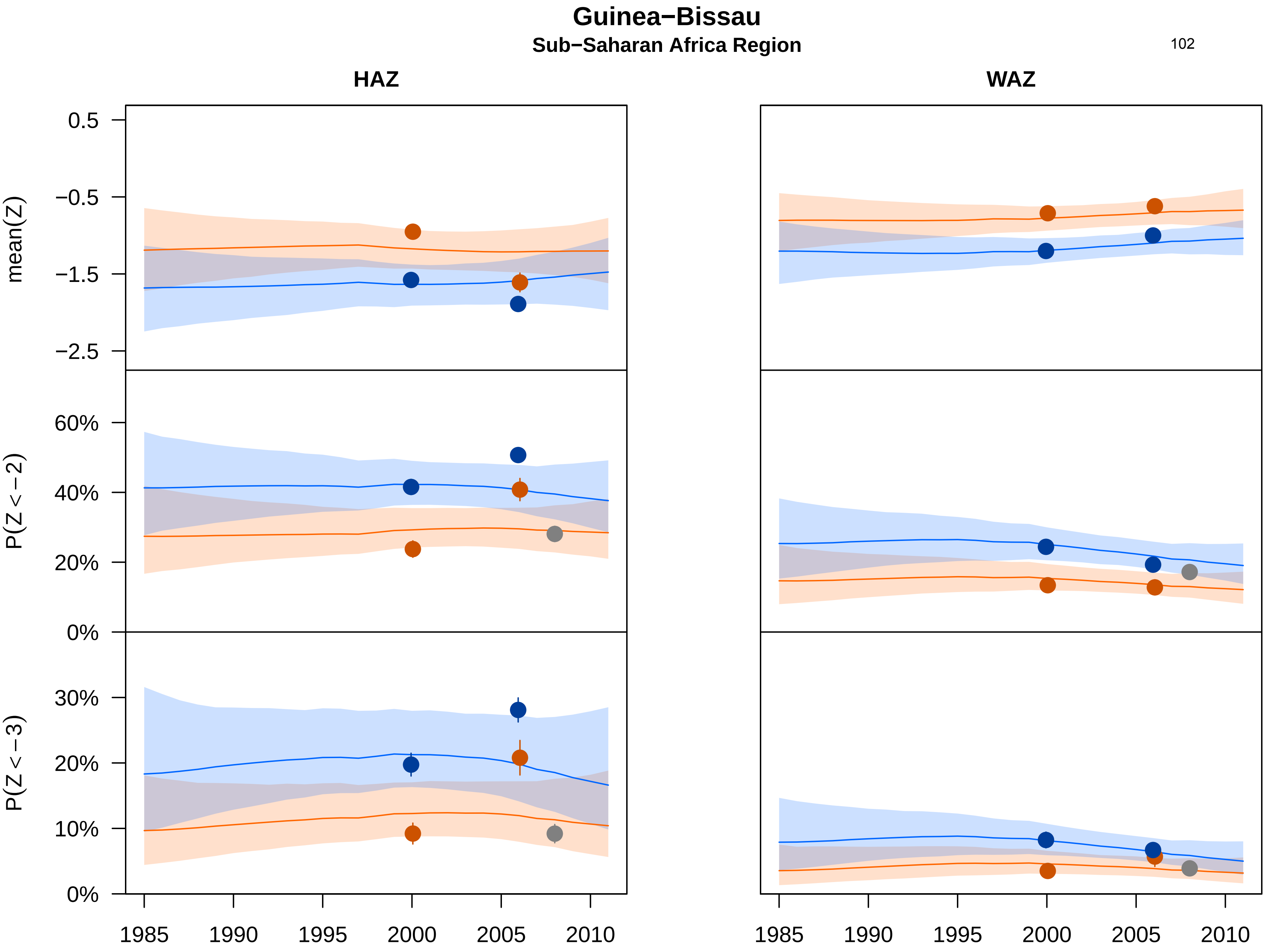
Guinea−Bissau
Sub−Saharan Africa Region
102
HAZ
WAZ
mean(Z)
P(Z < −2)
P(Z < −3)
0.5
−0.5
−1.5
−2.5
60%
40%
20%
0%
30%
20%
10%
0%
1985
1990
1995
2000
2005
2010

Guyana

Andean and Central Latin America and Caribbean Region

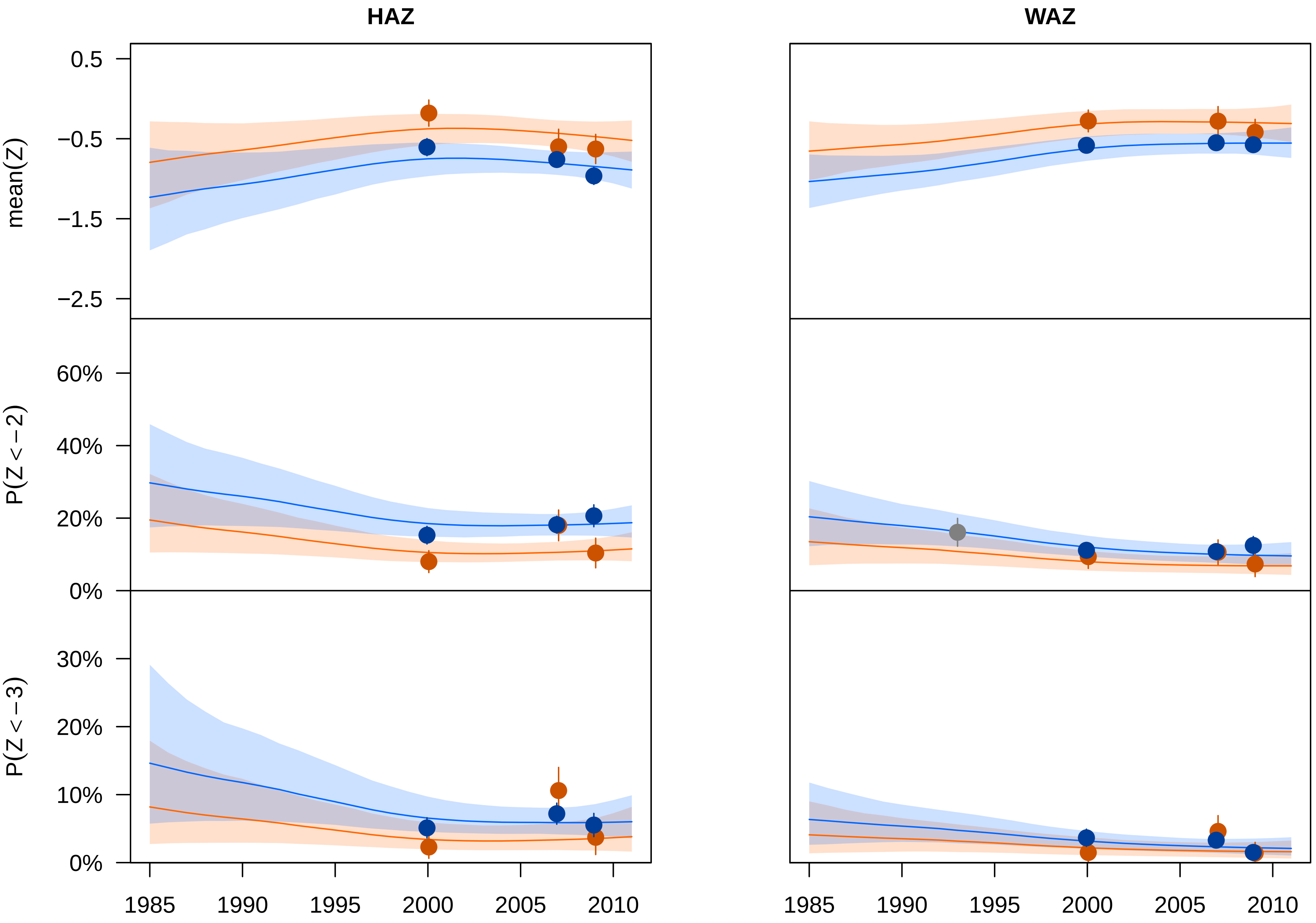

Haiti

Andean and Central Latin America and Caribbean Region

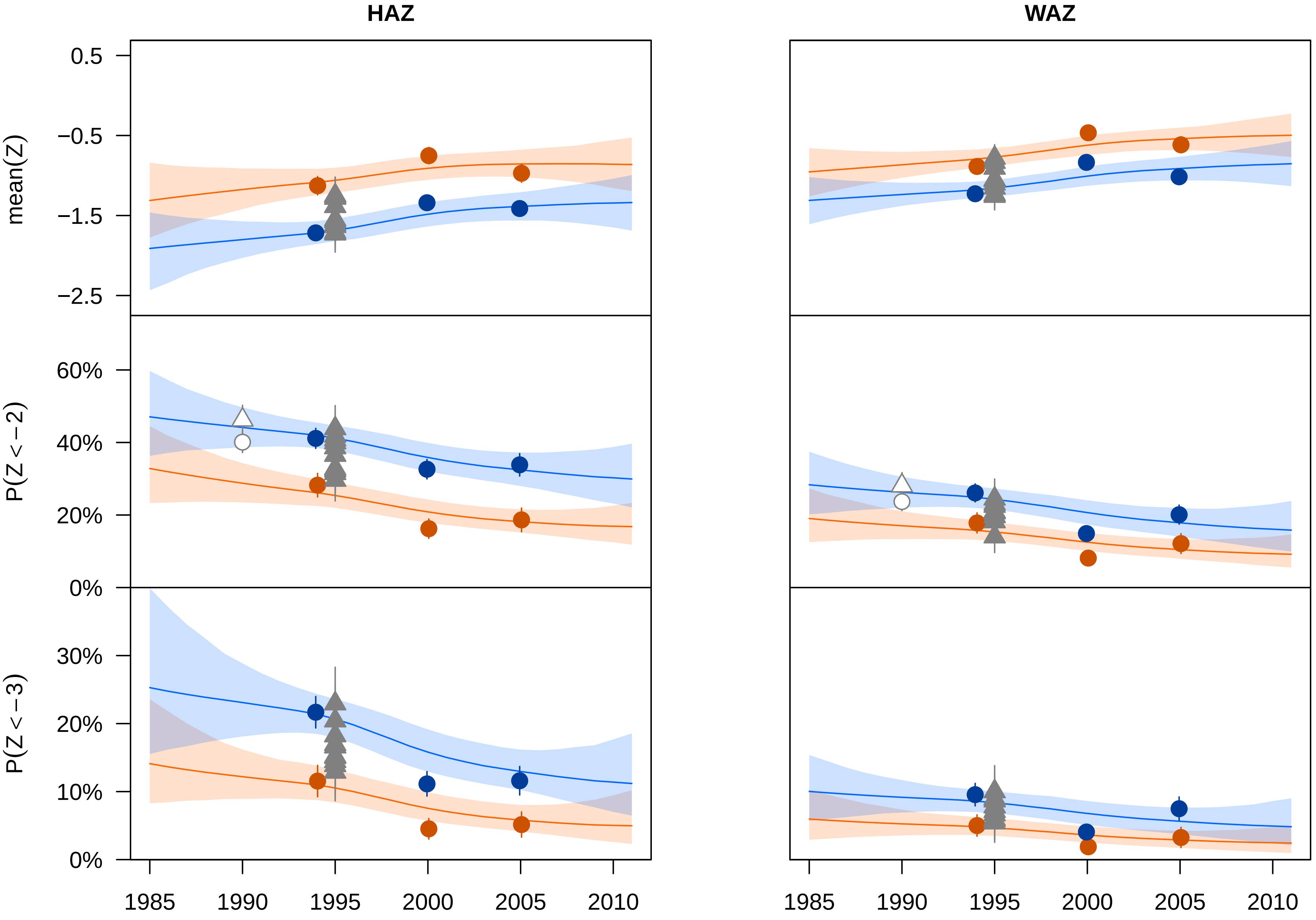

Honduras

Andean and Central Latin America and Caribbean Region

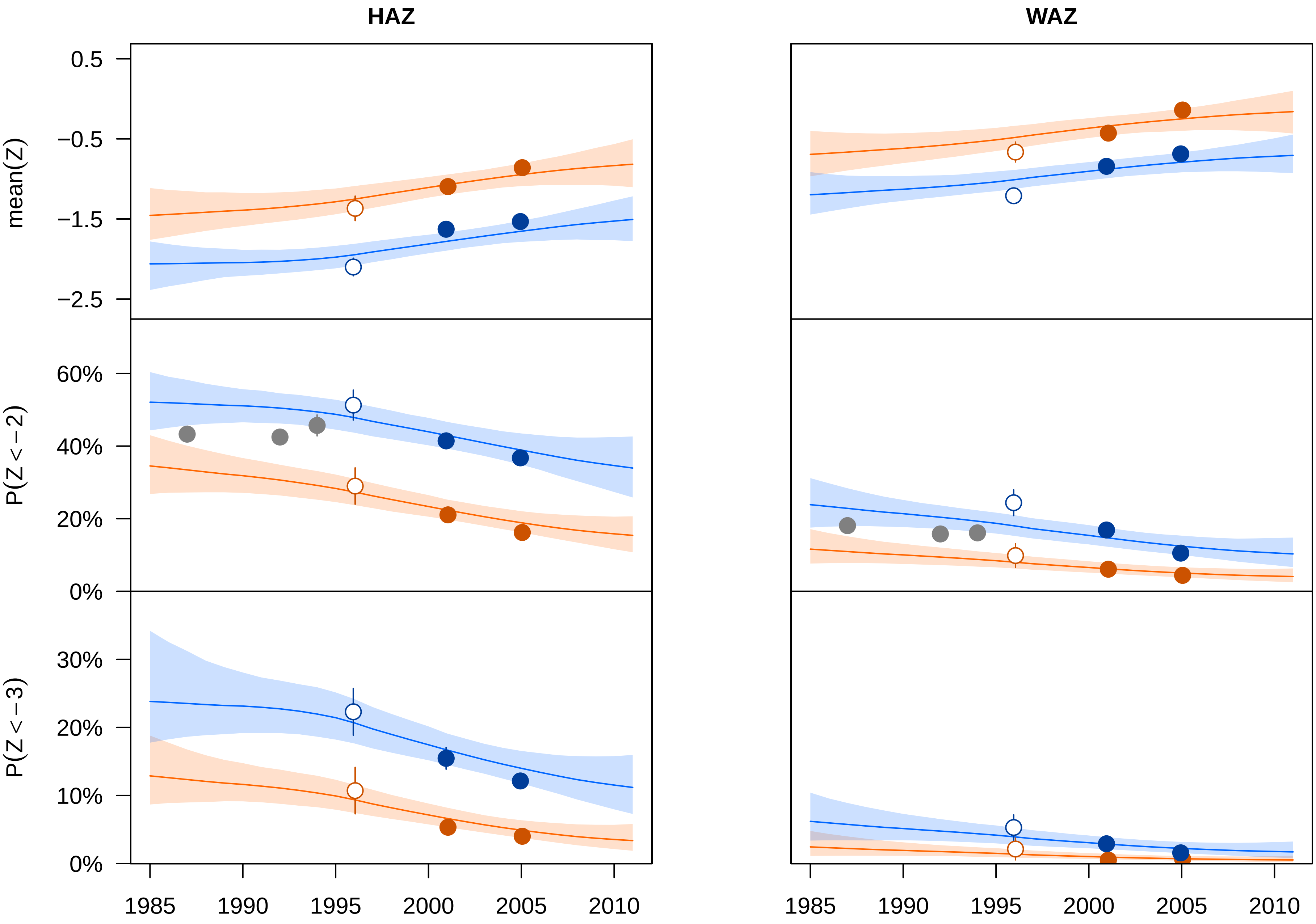

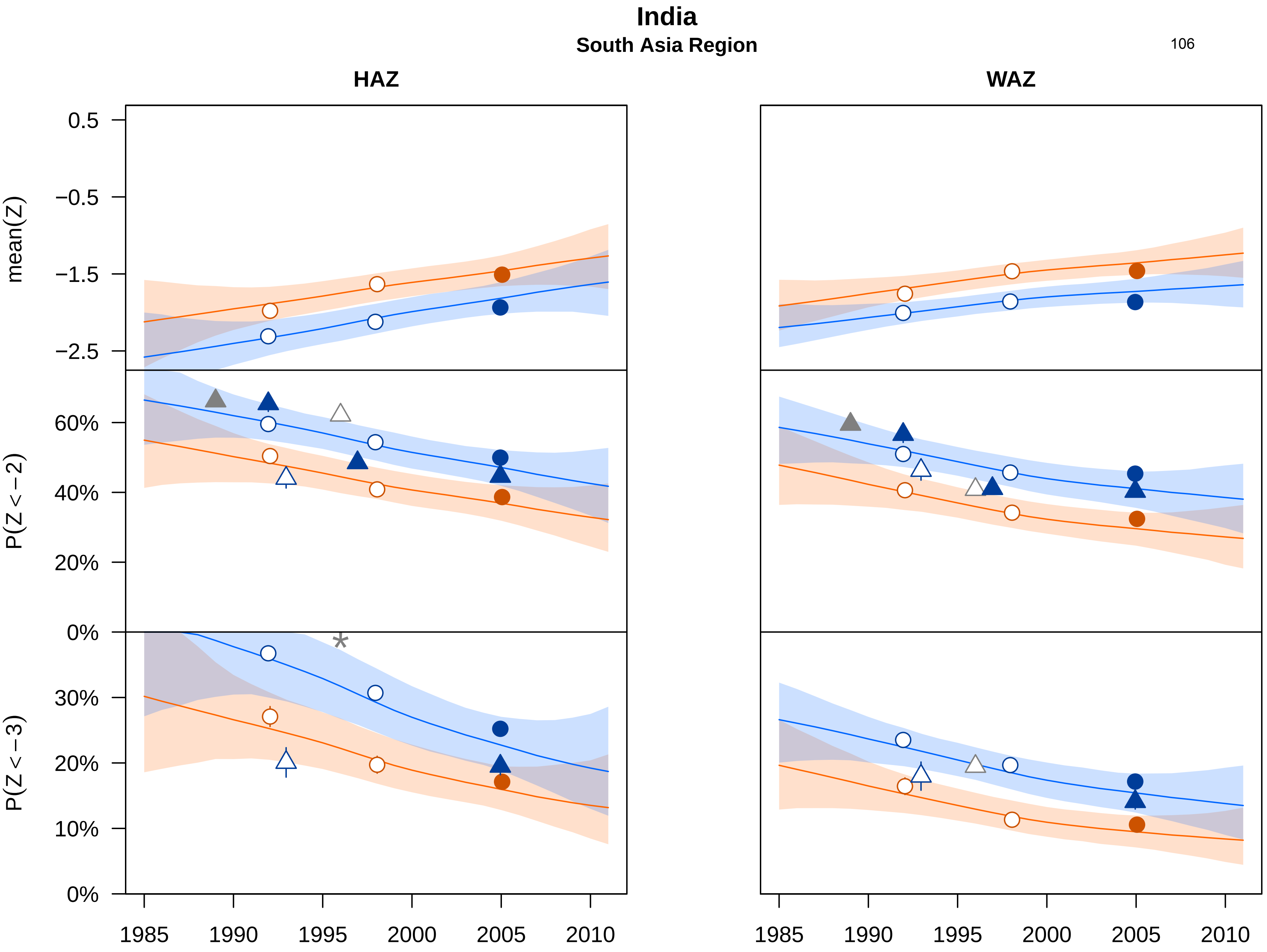
India
South Asia Region
HAZ
WAZ
mean(Z)
P(Z < −2)
P(Z < −3)
0.5
−0.5
−1.5
−2.5
60%
40%
20%
0%
30%
20%
10%
0%
1985
1990
1995
2000
2005
2010
1985
1990
1995
2000
2005
2010

Indonesia

East and Southeast Asia Region

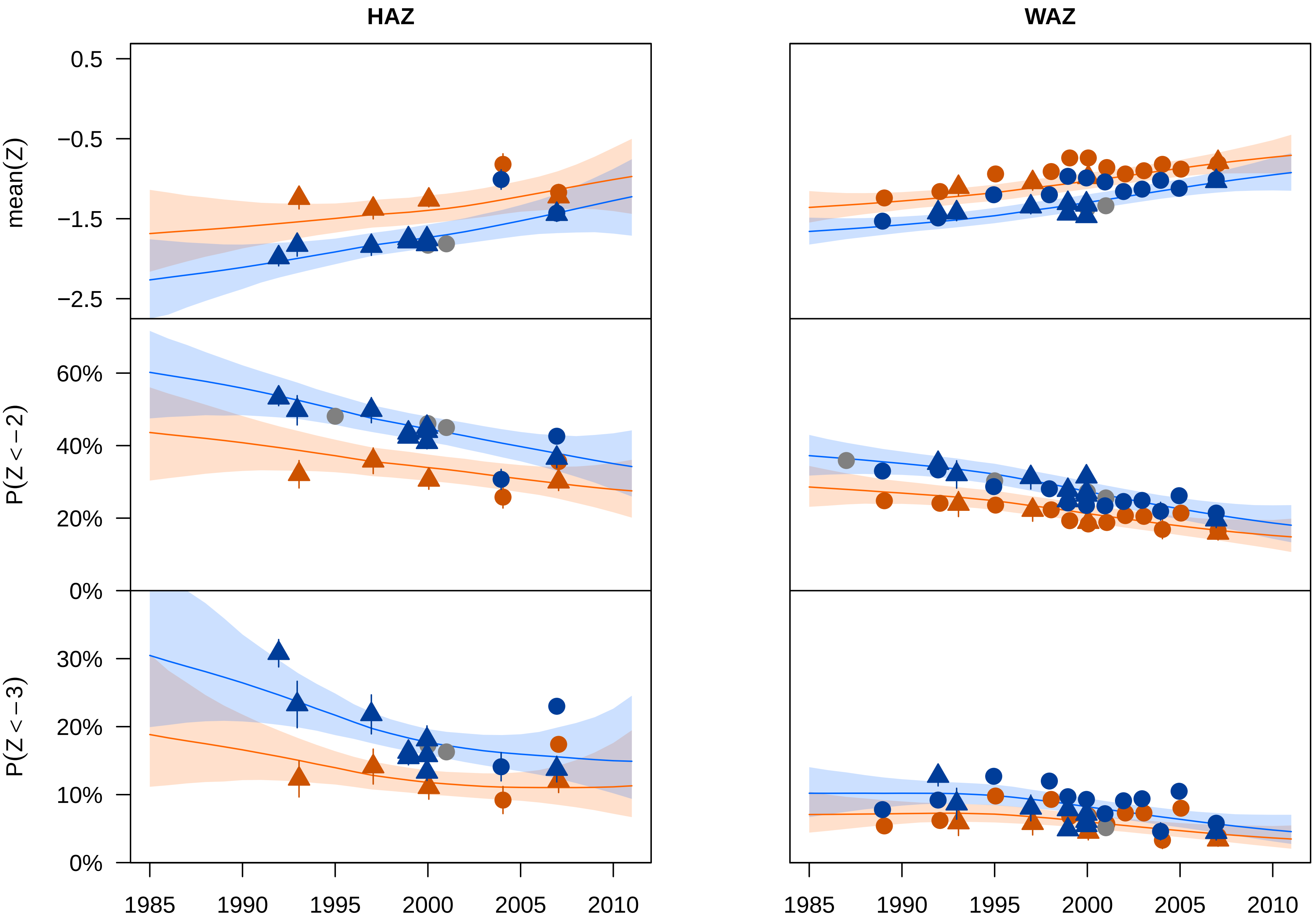

Iran (Islamic Republic of)

Central Asia, Middle East, and North Africa Region

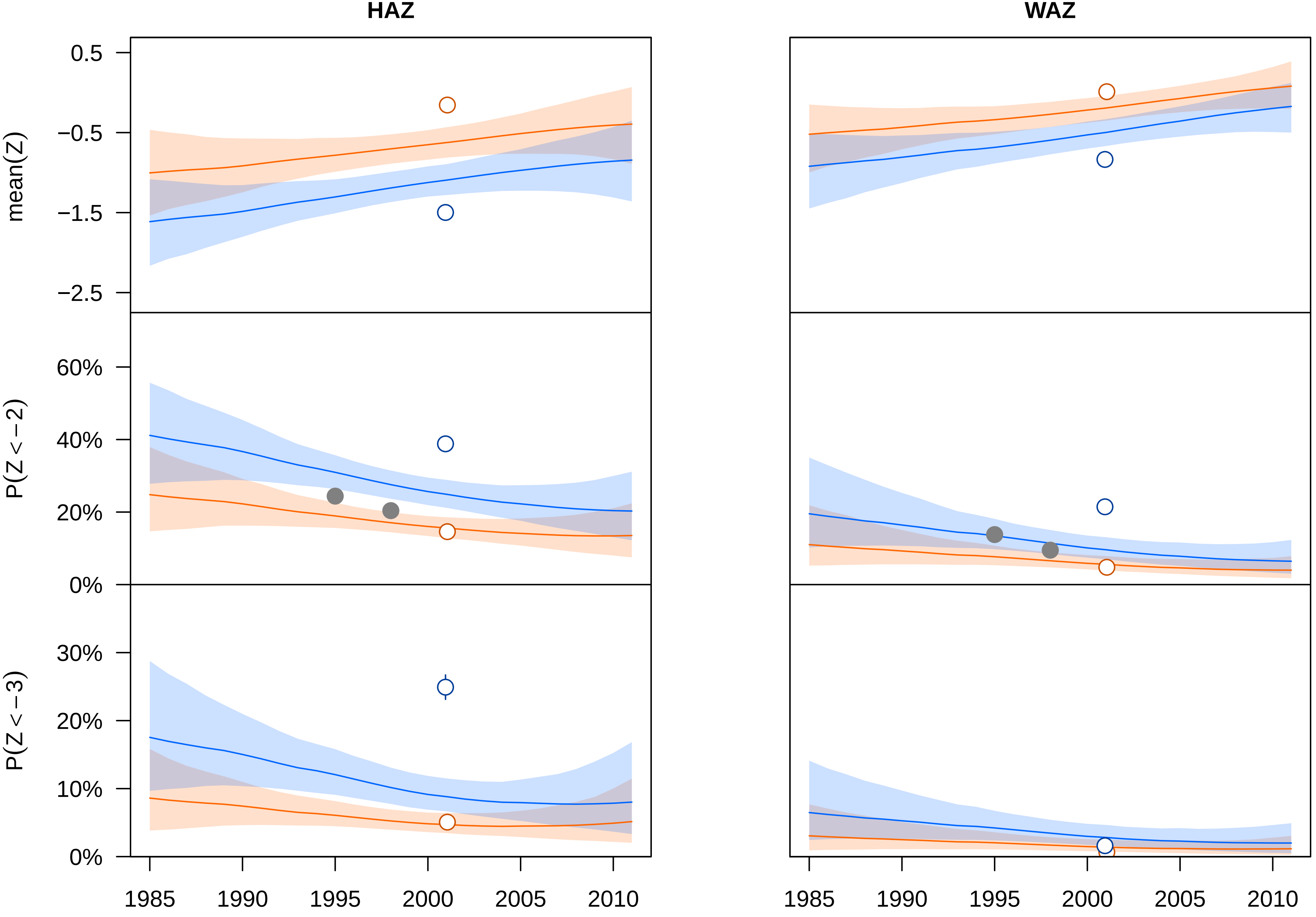

Iraq

Central Asia, Middle East, and North Africa Region

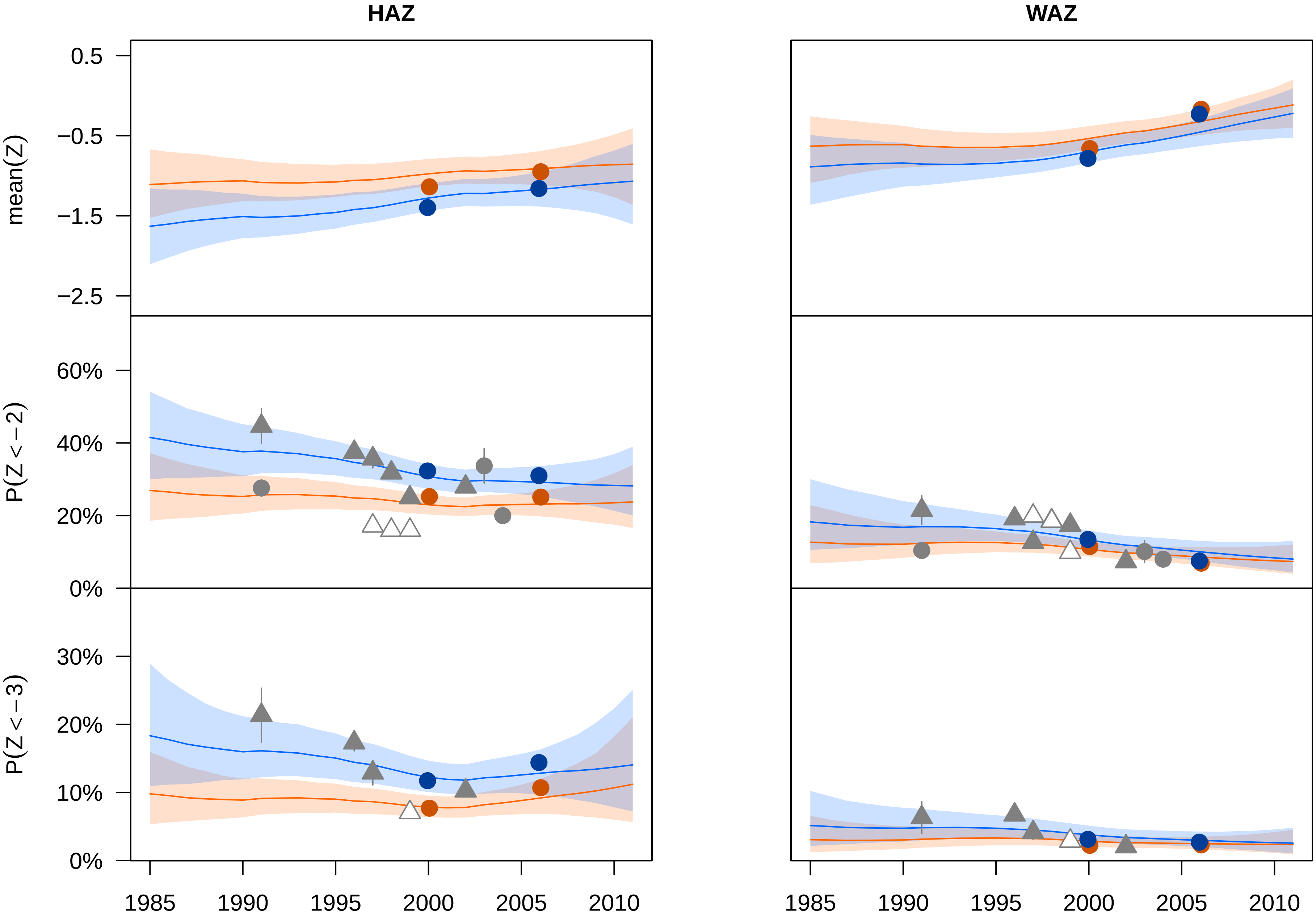

Jamaica

Andean and Central Latin America and Caribbean Region

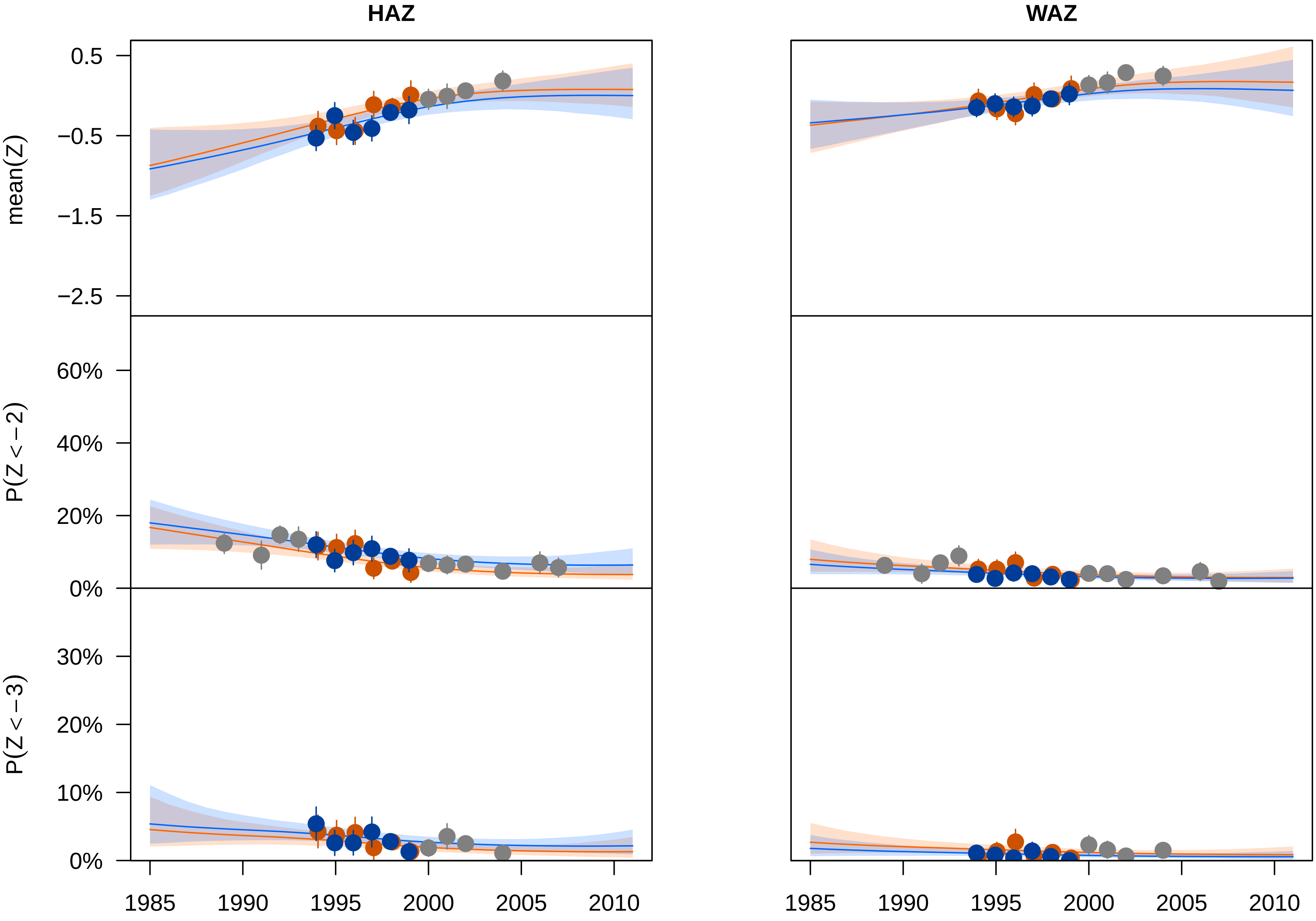

Jordan

Central Asia, Middle East, and North Africa Region

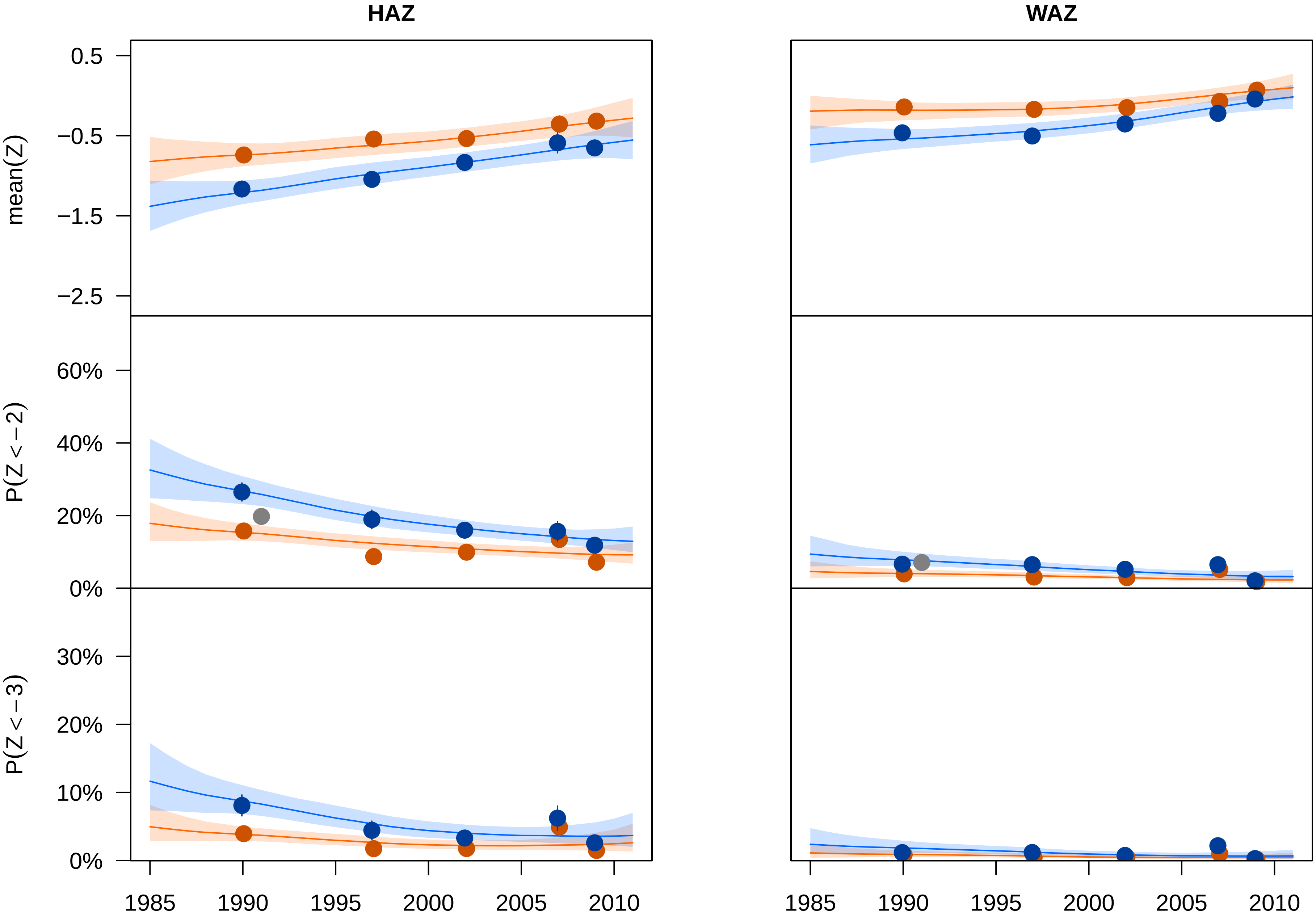

Kazakhstan

Central Asia, Middle East, and North Africa Region

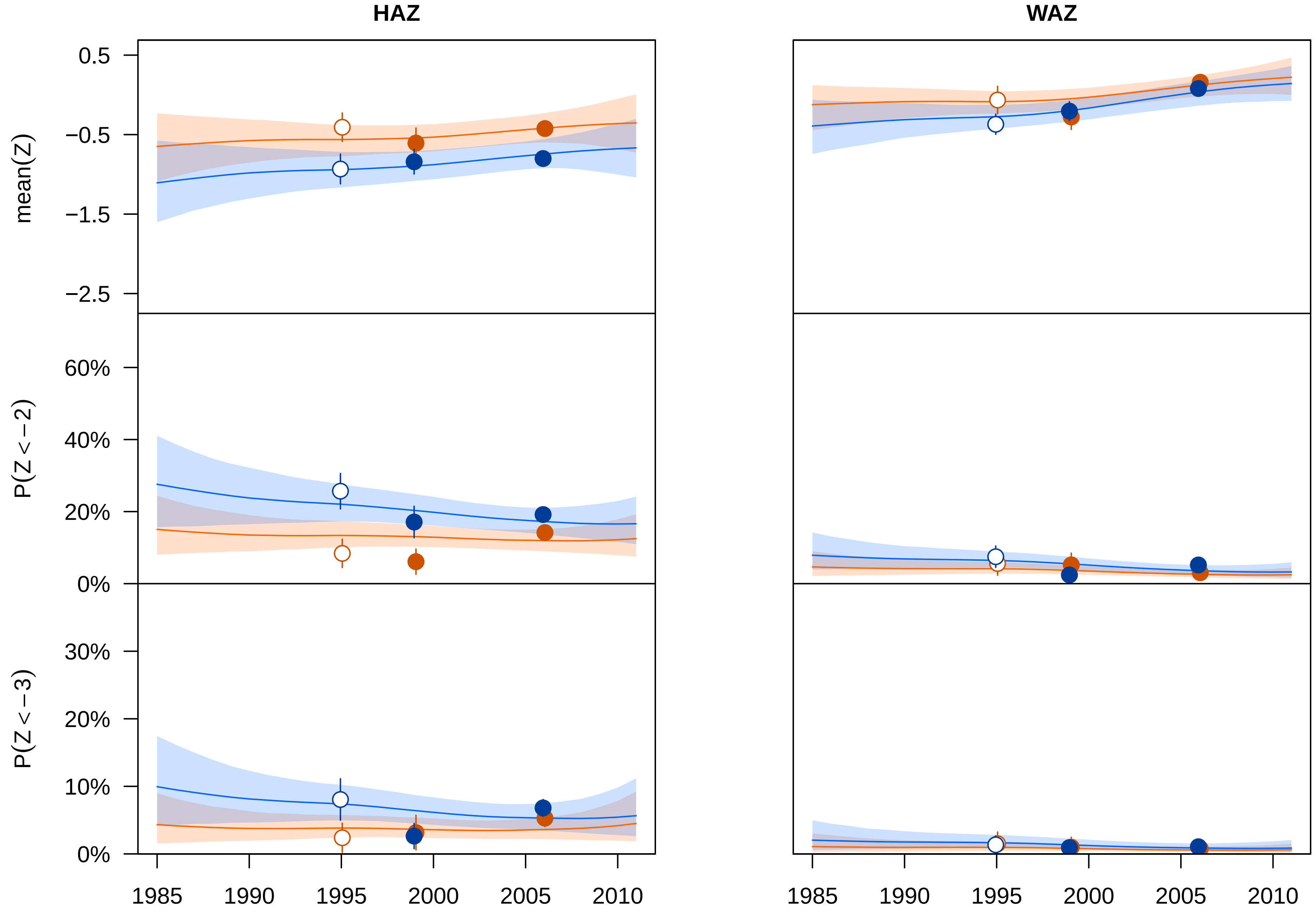

Kenya

Sub−Saharan Africa Region

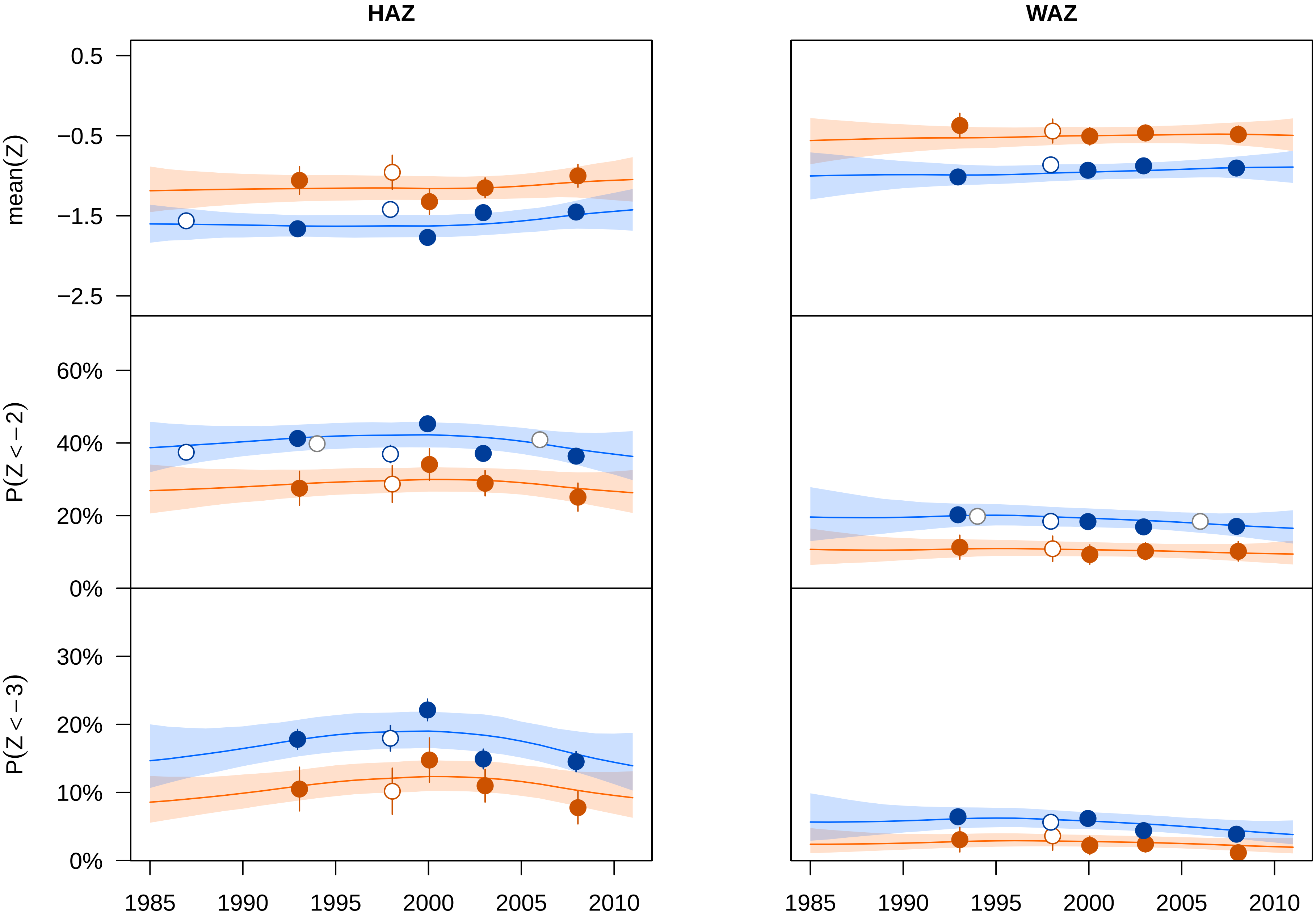

Kiribati

Oceania Region

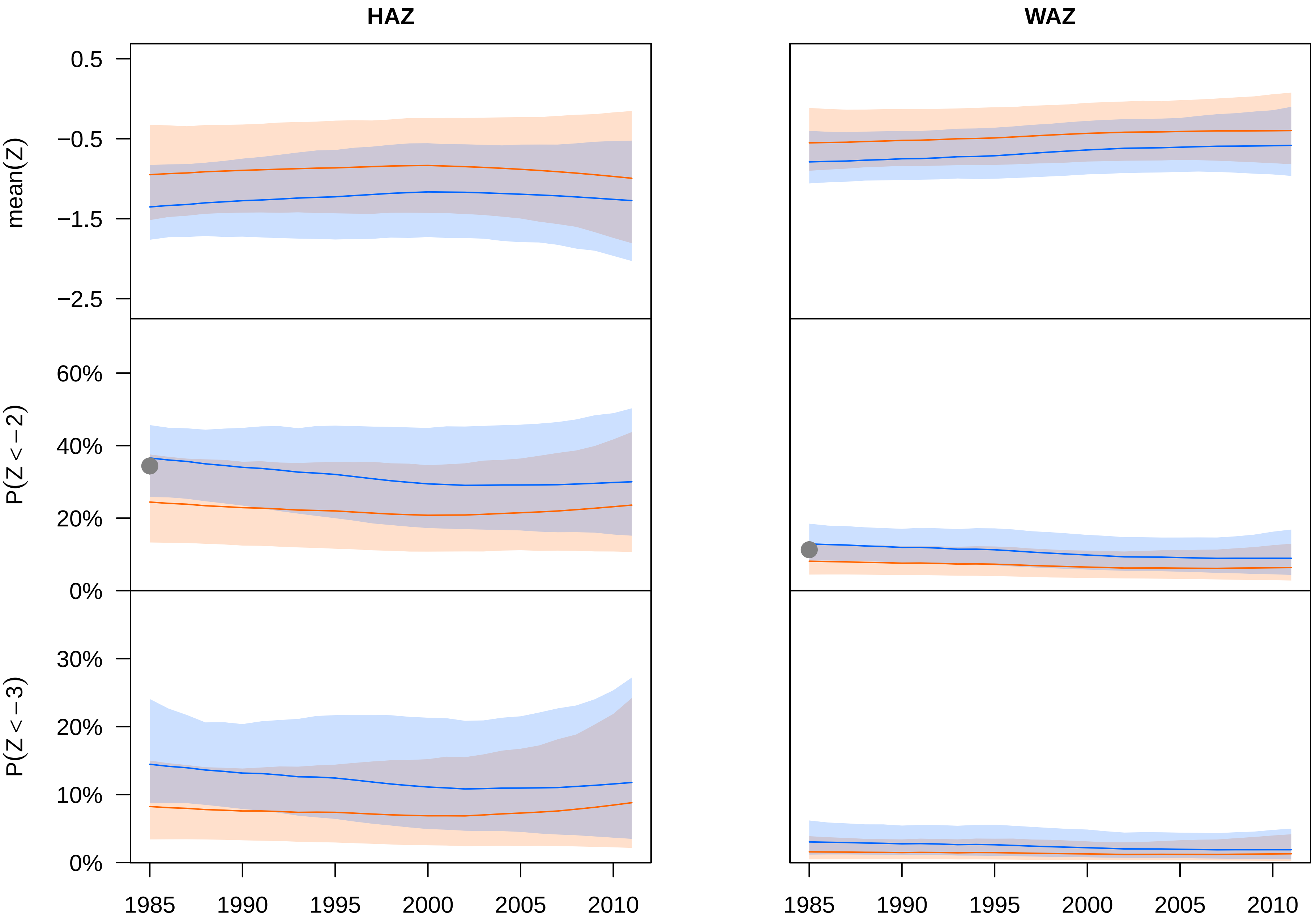

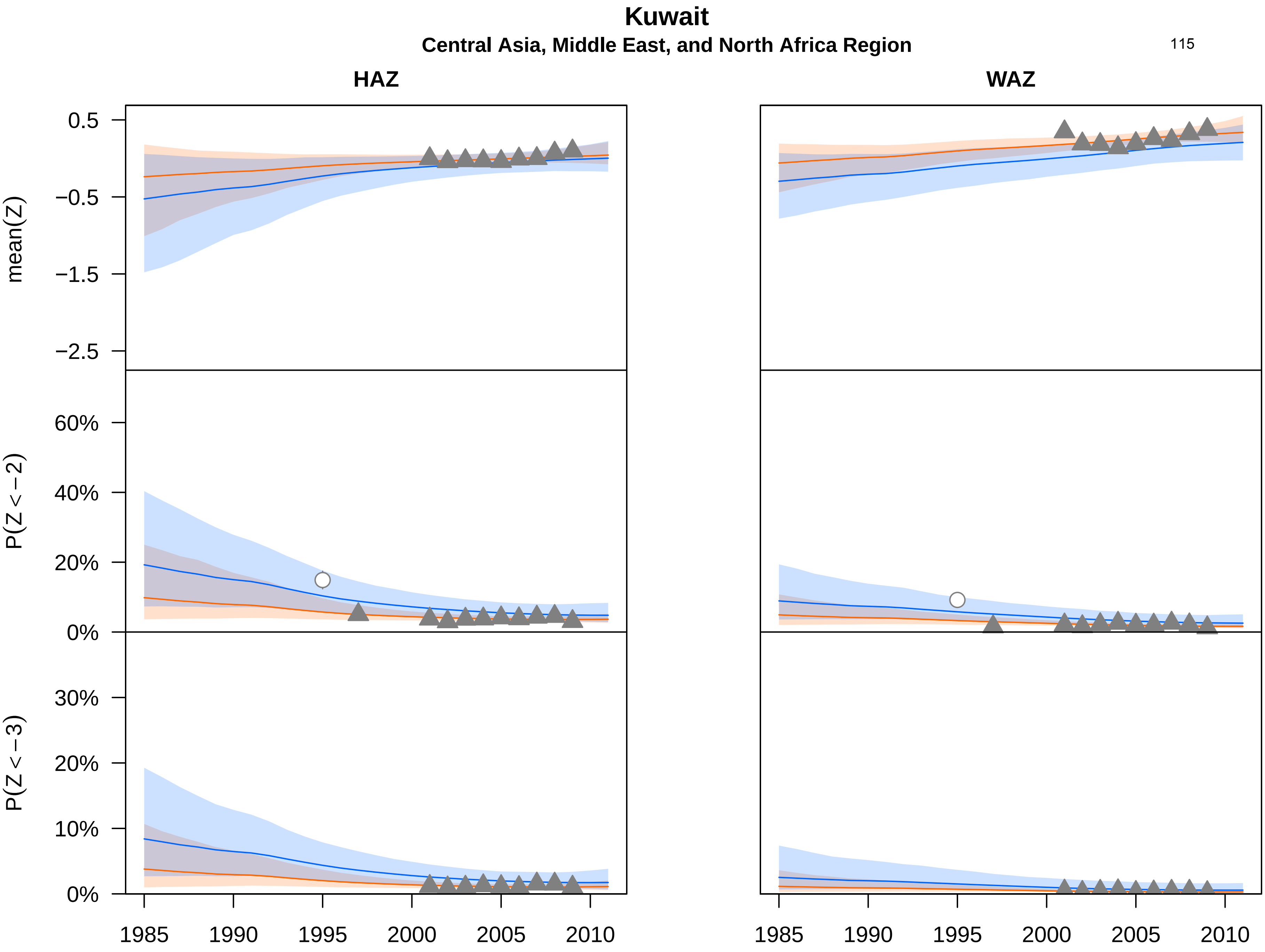
Kuwait
Central Asia, Middle East, and North Africa Region
HAZ
WAZ
mean(Z)
P(Z < −2)
P(Z < −3)
0.5
−0.5
−1.5
−2.5
60%
40%
20%
0%
30%
20%
10%
0%
1985
1990
1995
2000
2005
2010

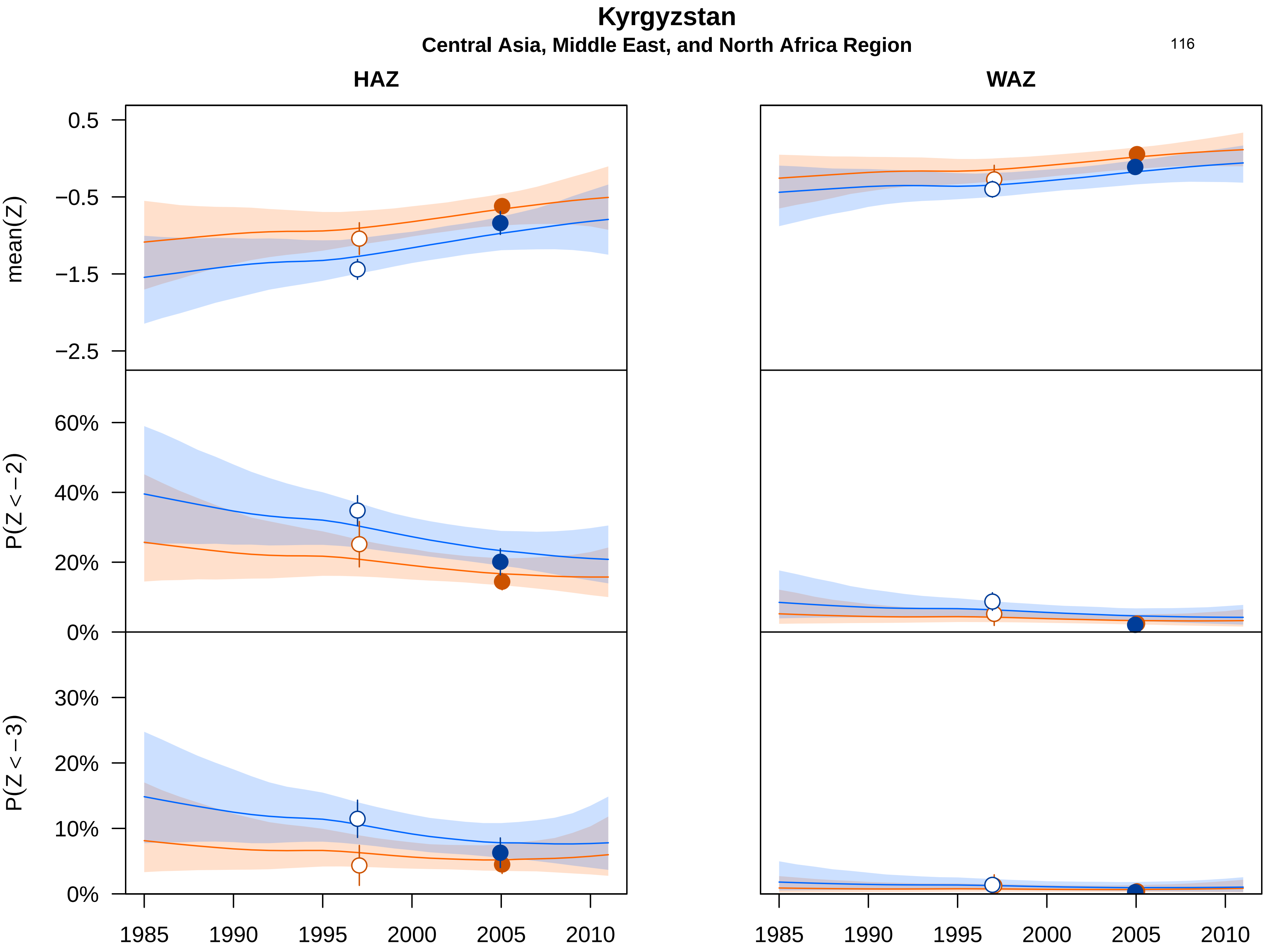
Kyrgyzstan
Central Asia, Middle East, and North Africa Region
HAZ
WAZ
mean(Z)
P(Z < −2)
P(Z < −3)
0.5
−0.5
−1.5
−2.5
60%
40%
20%
0%
30%
20%
10%
0%
1985
1990
1995
2000
2005
2010
1985
1990
1995
2000
2005
2010

Lao People's Democratic Republic

East and Southeast Asia Region

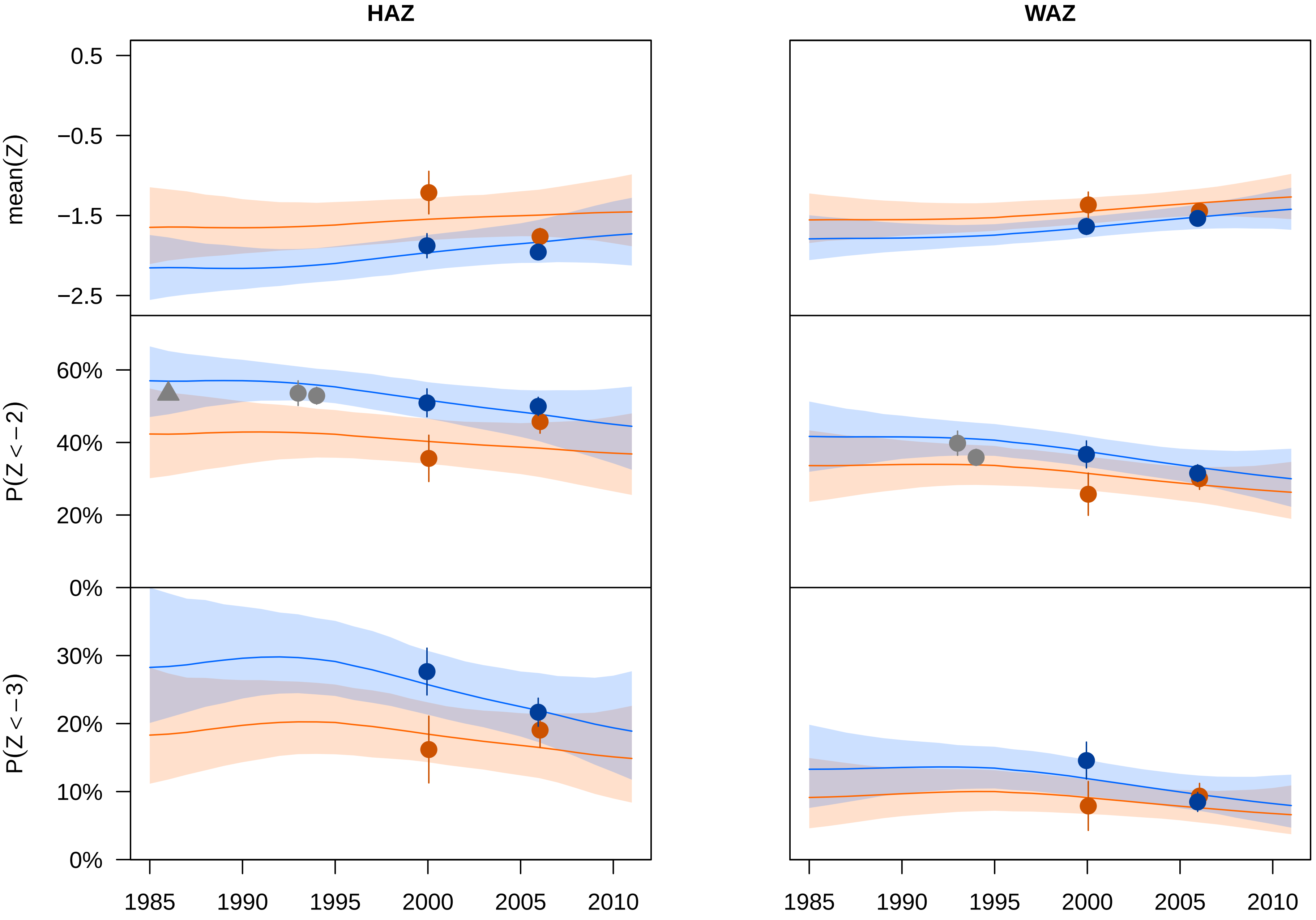

Lebanon

Central Asia, Middle East, and North Africa Region

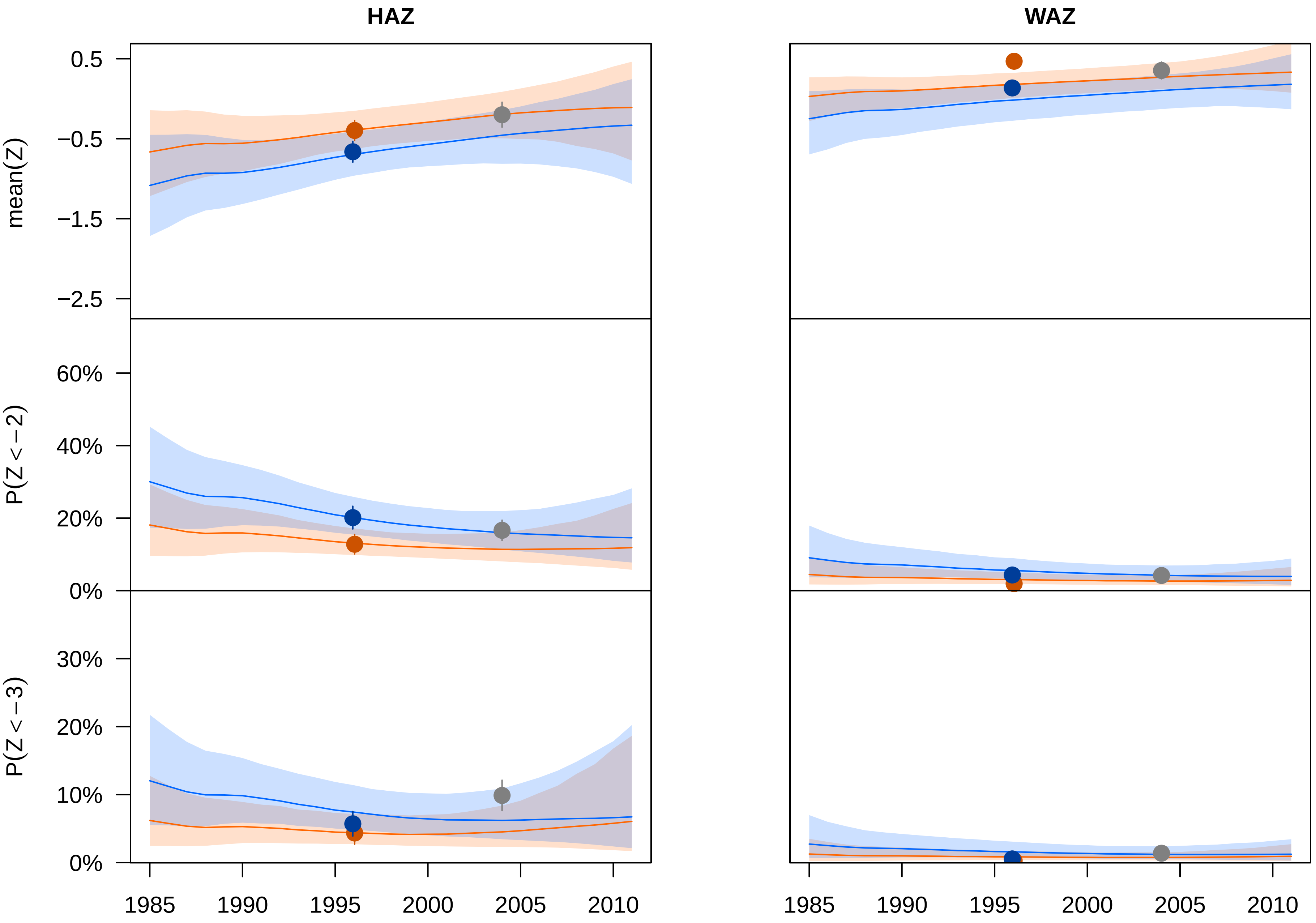

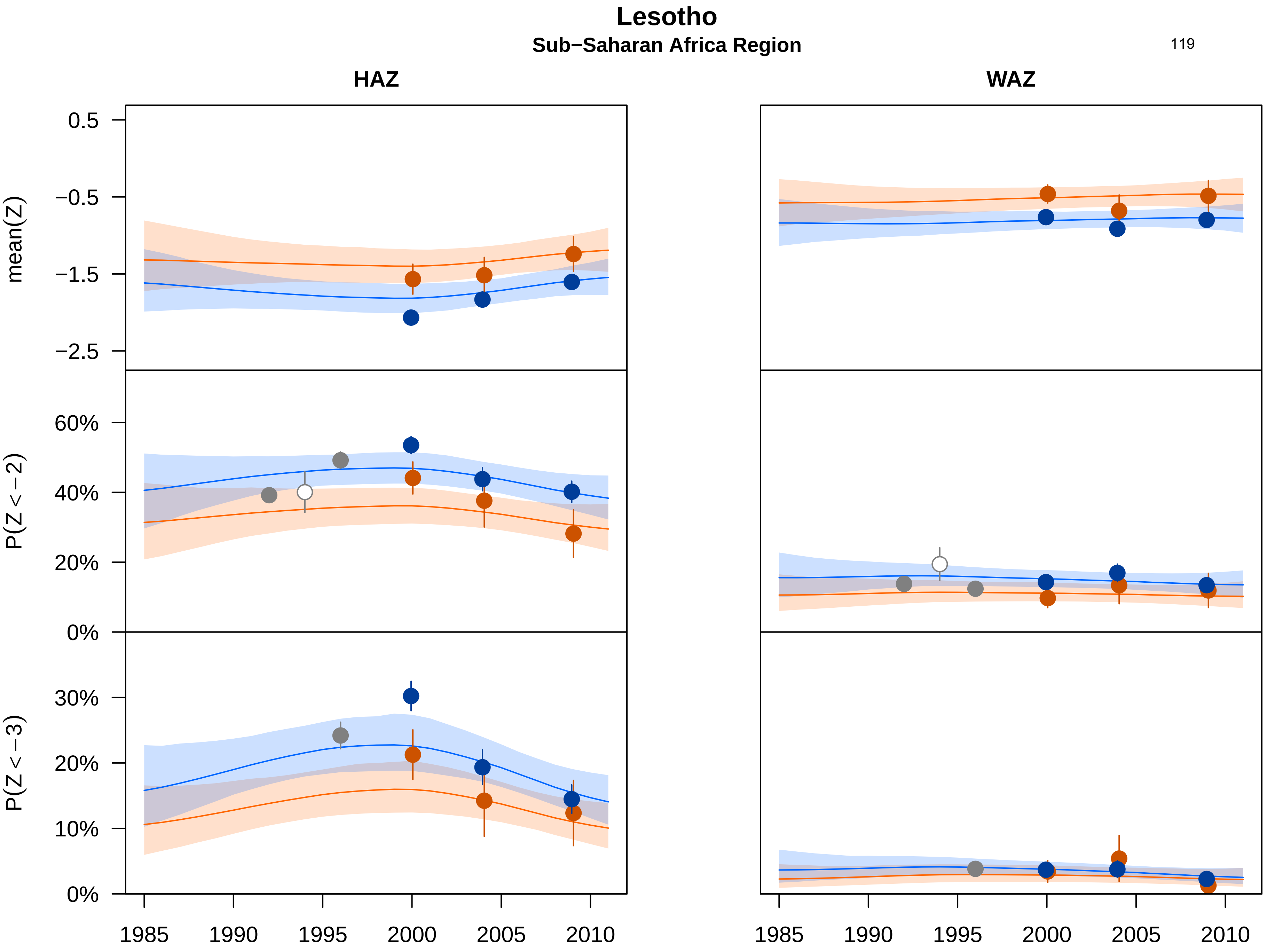
Lesotho
Sub−Saharan Africa Region
HAZ
WAZ
mean(Z)
P(Z < −2)
P(Z < −3)
0.5
−0.5
−1.5
−2.5
60%
40%
20%
0%
30%
20%
10%
0%
1985
1990
1995
2000
2005
2010

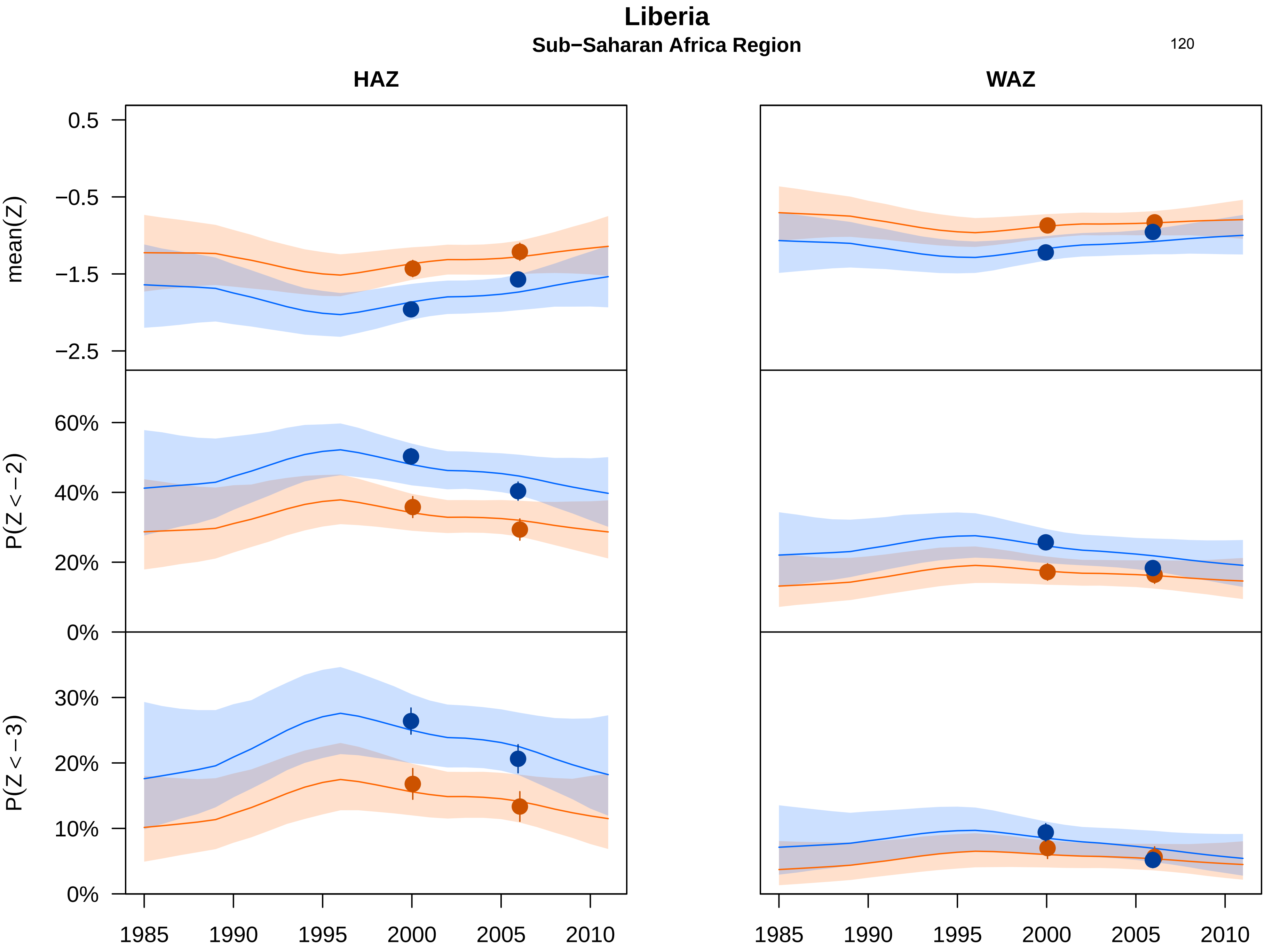
Liberia
Sub−Saharan Africa Region
HAZ
WAZ
mean(Z)
P(Z < −2)
P(Z < −3)
0.5
−0.5
−1.5
−2.5
60%
40%
20%
0%
30%
20%
10%
0%
1985
1990
1995
2000
2005
2010

Libyan Arab Jamahiriya

Central Asia, Middle East, and North Africa Region

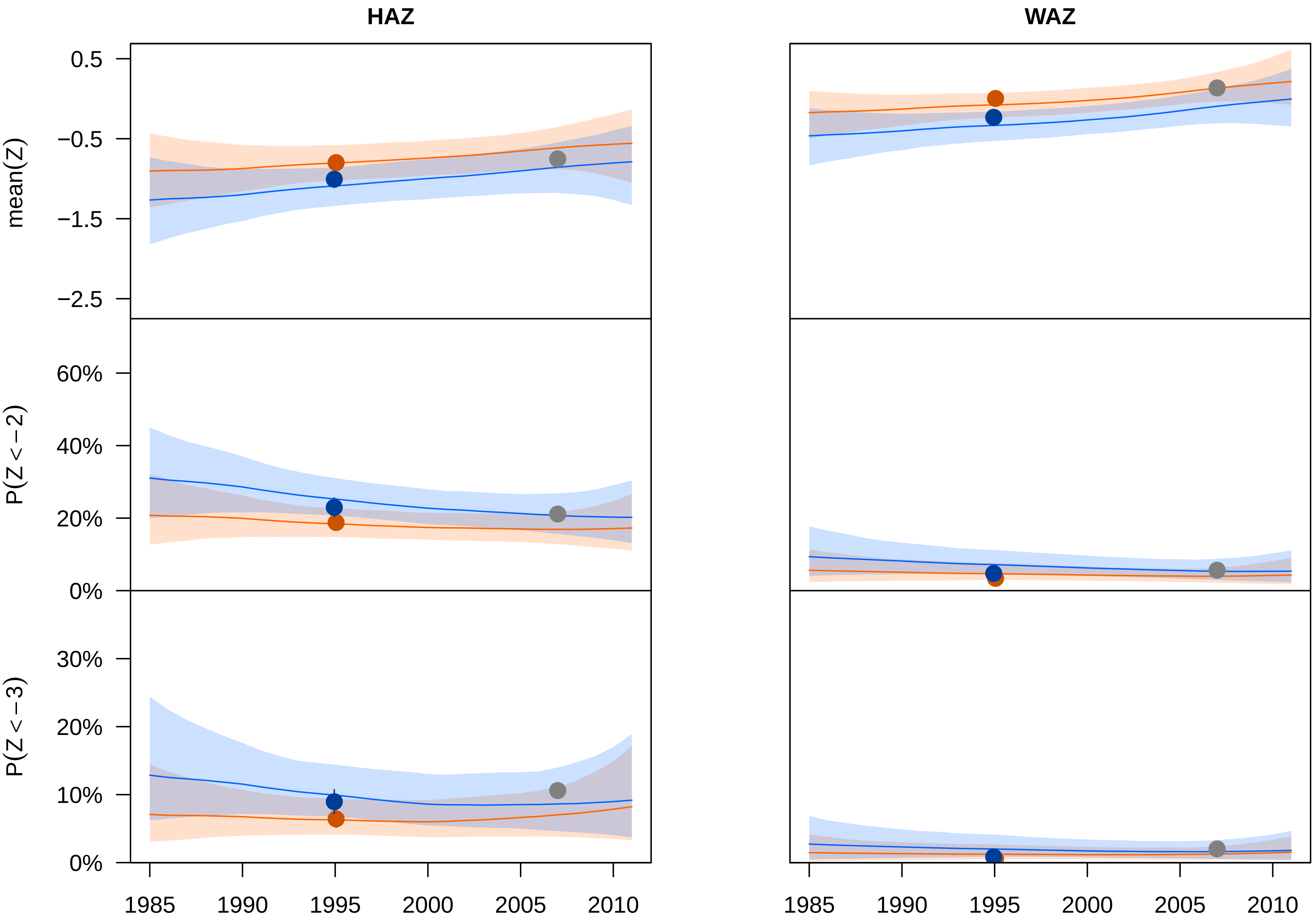

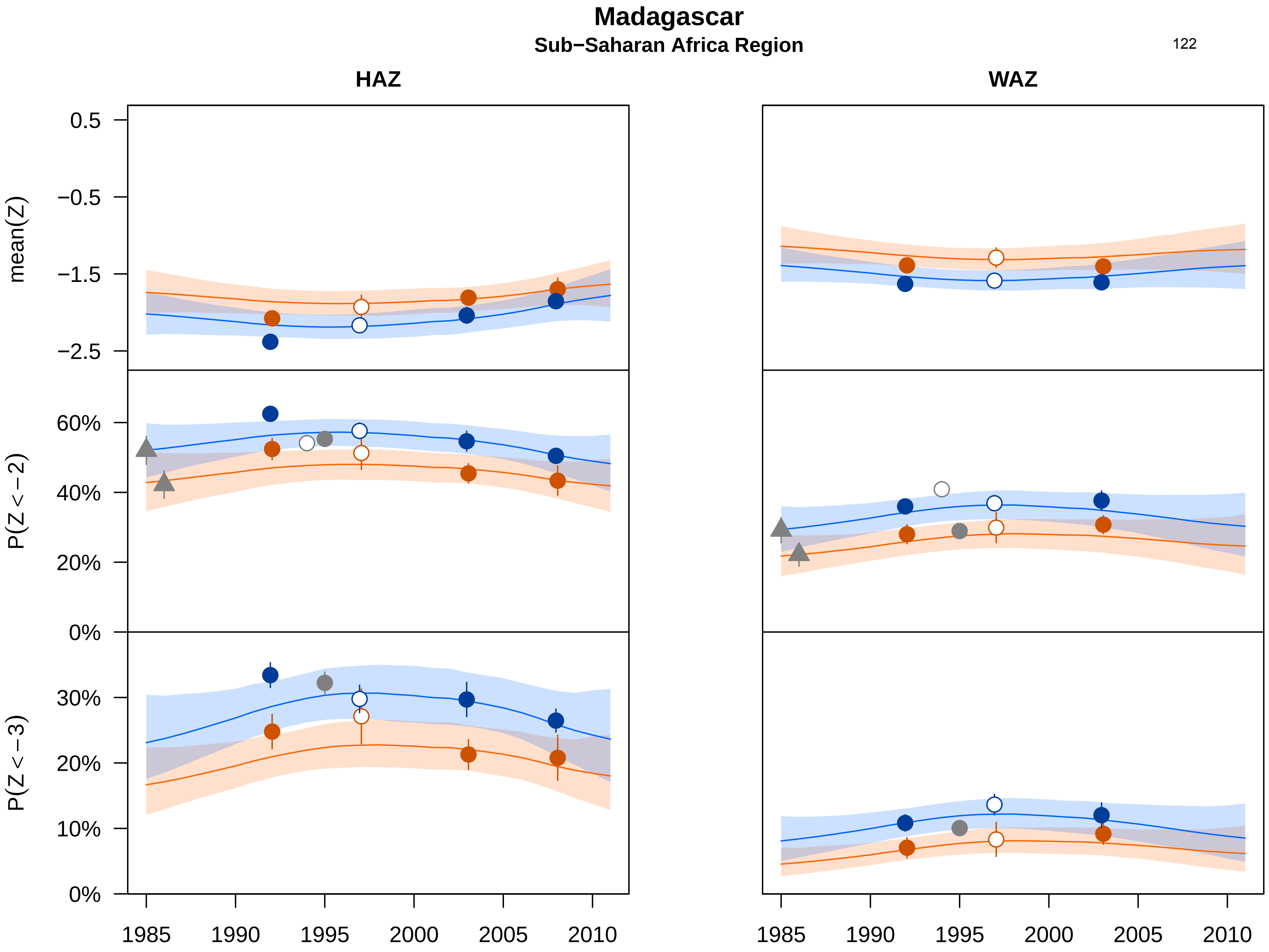
Madagascar
Sub−Saharan Africa Region
HAZ
WAZ
mean(Z)
P(Z < −2)
P(Z < −3)
0.5
−0.5
−1.5
−2.5
60%
40%
20%
0%
30%
20%
10%
0%
1985
1990
1995
2000
2005
2010

Malawi

Sub−Saharan Africa Region

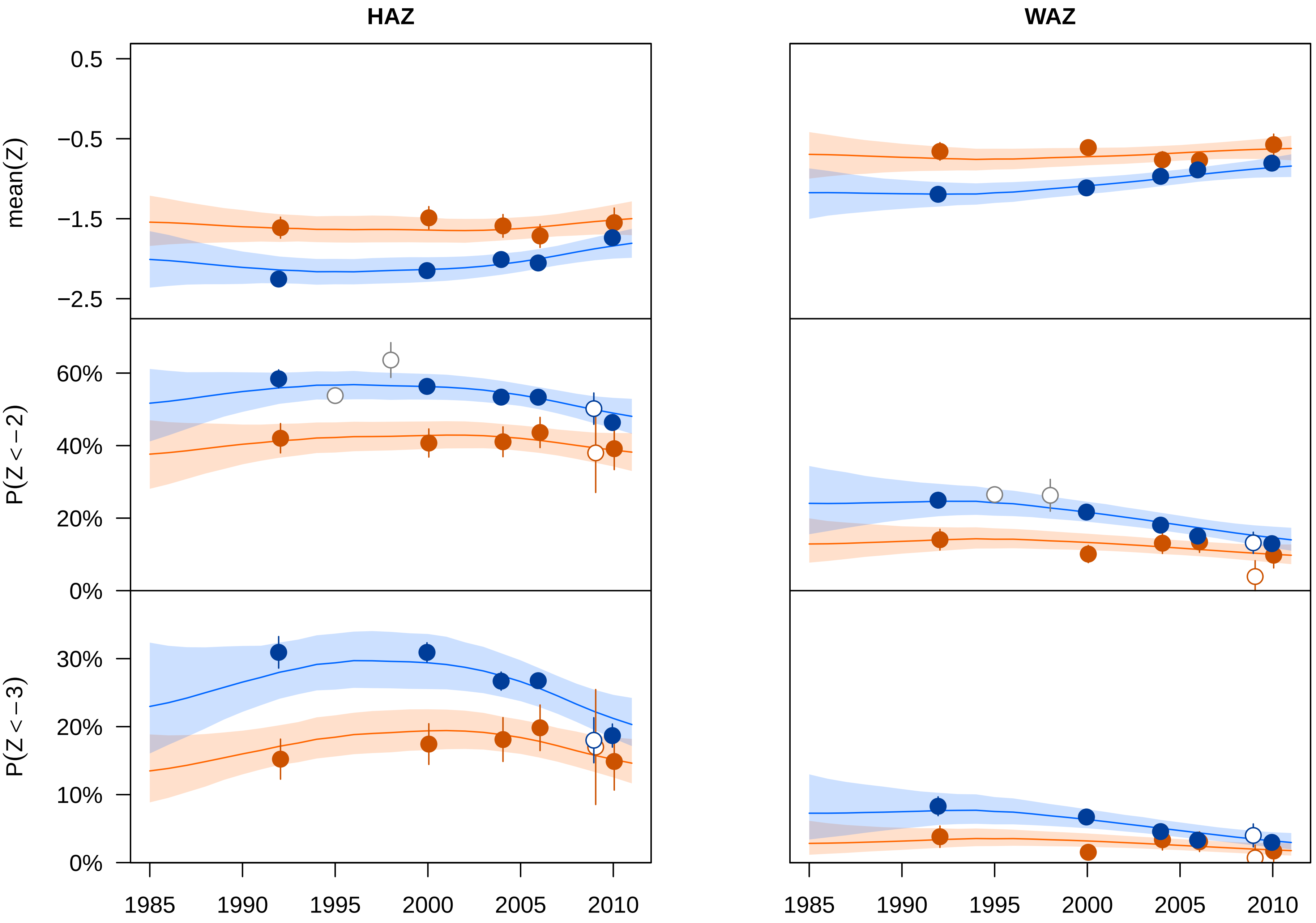

Malaysia

East and Southeast Asia Region

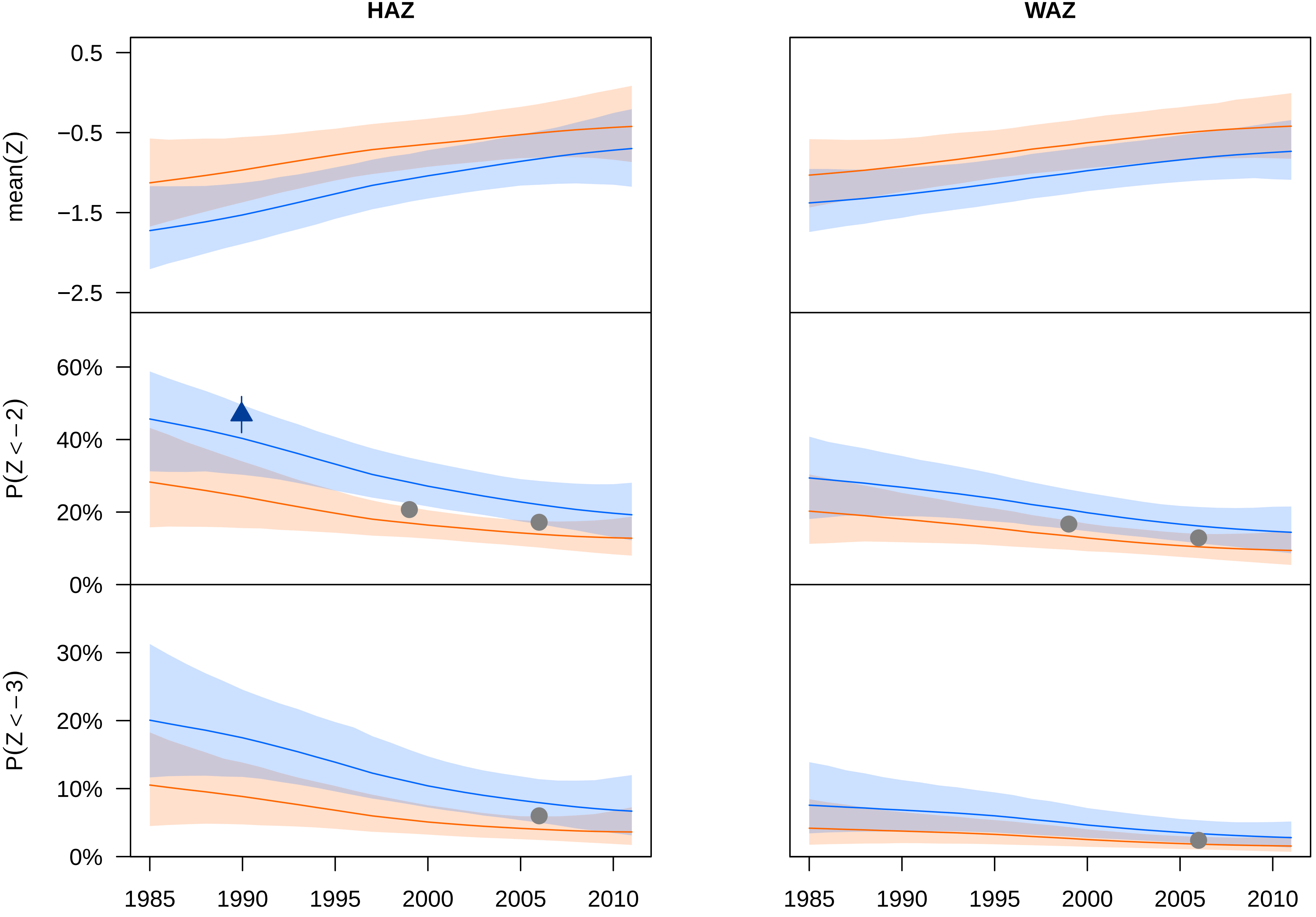

Maldives
East and Southeast Asia Region

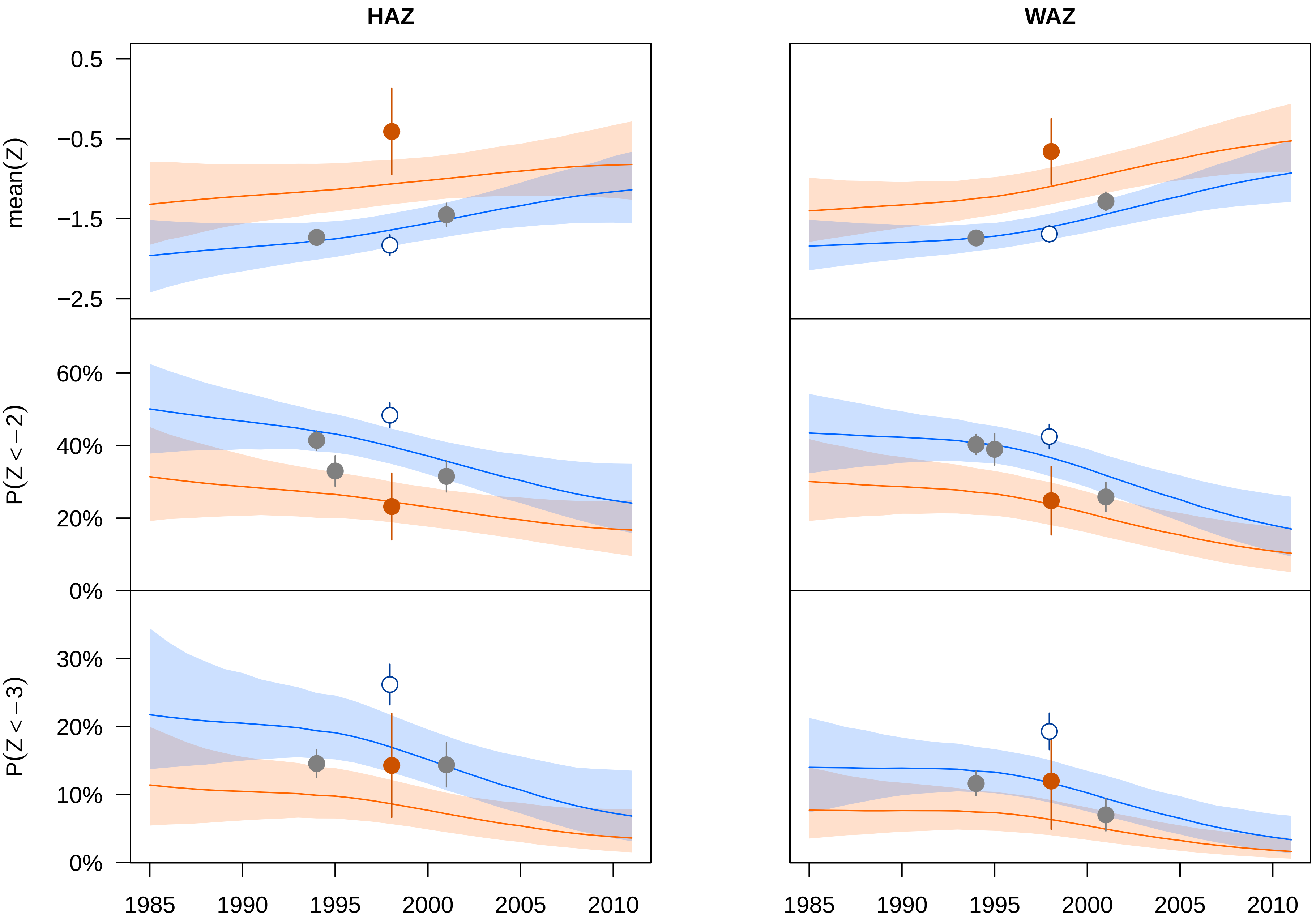

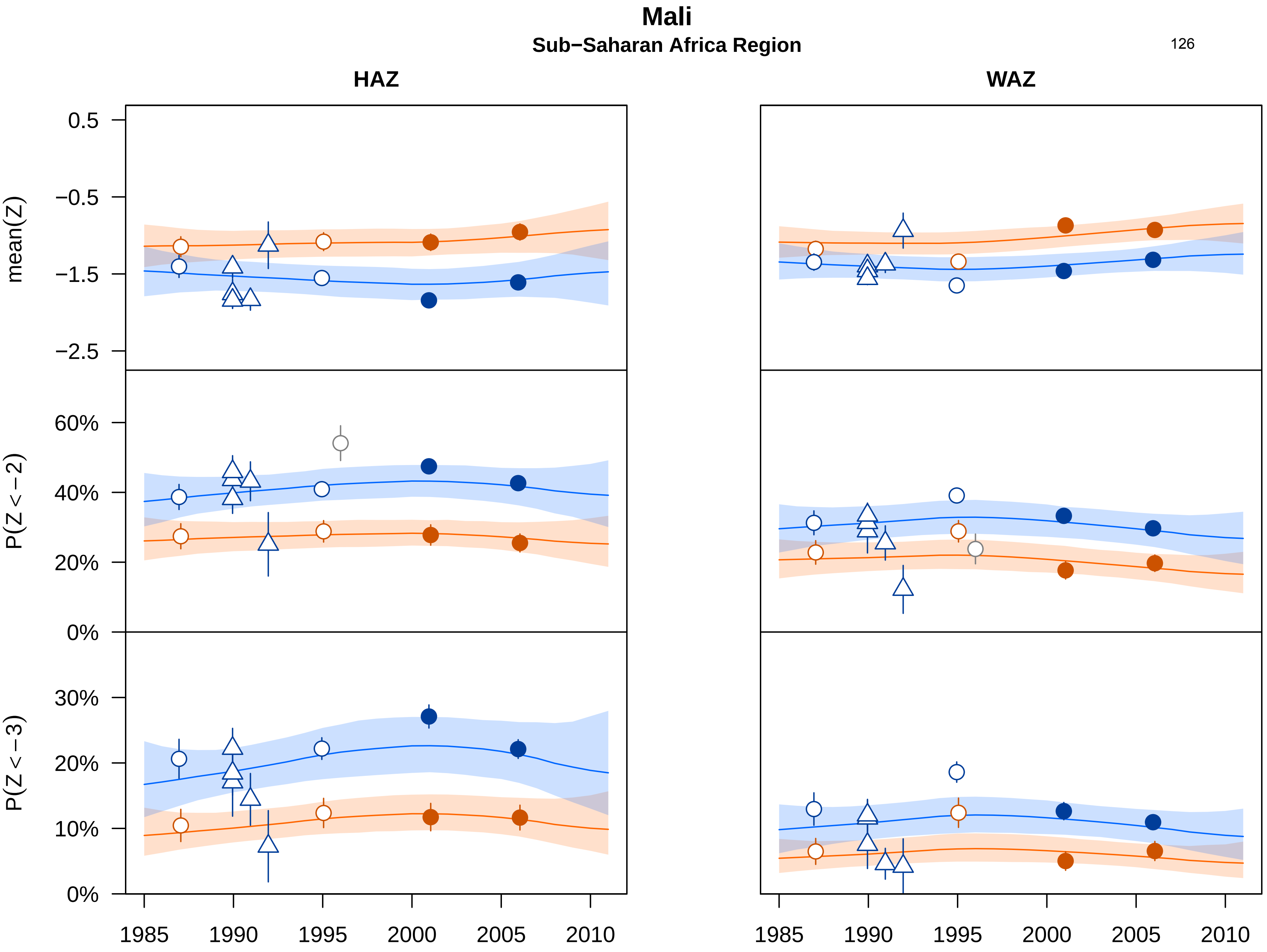
Mali
Sub−Saharan Africa Region
HAZ
WAZ
mean(Z)
P(Z < −2)
P(Z < −3)
0.5
−0.5
−1.5
−2.5
60%
40%
20%
0%
30%
20%
10%
0%
1985
1990
1995
2000
2005
2010

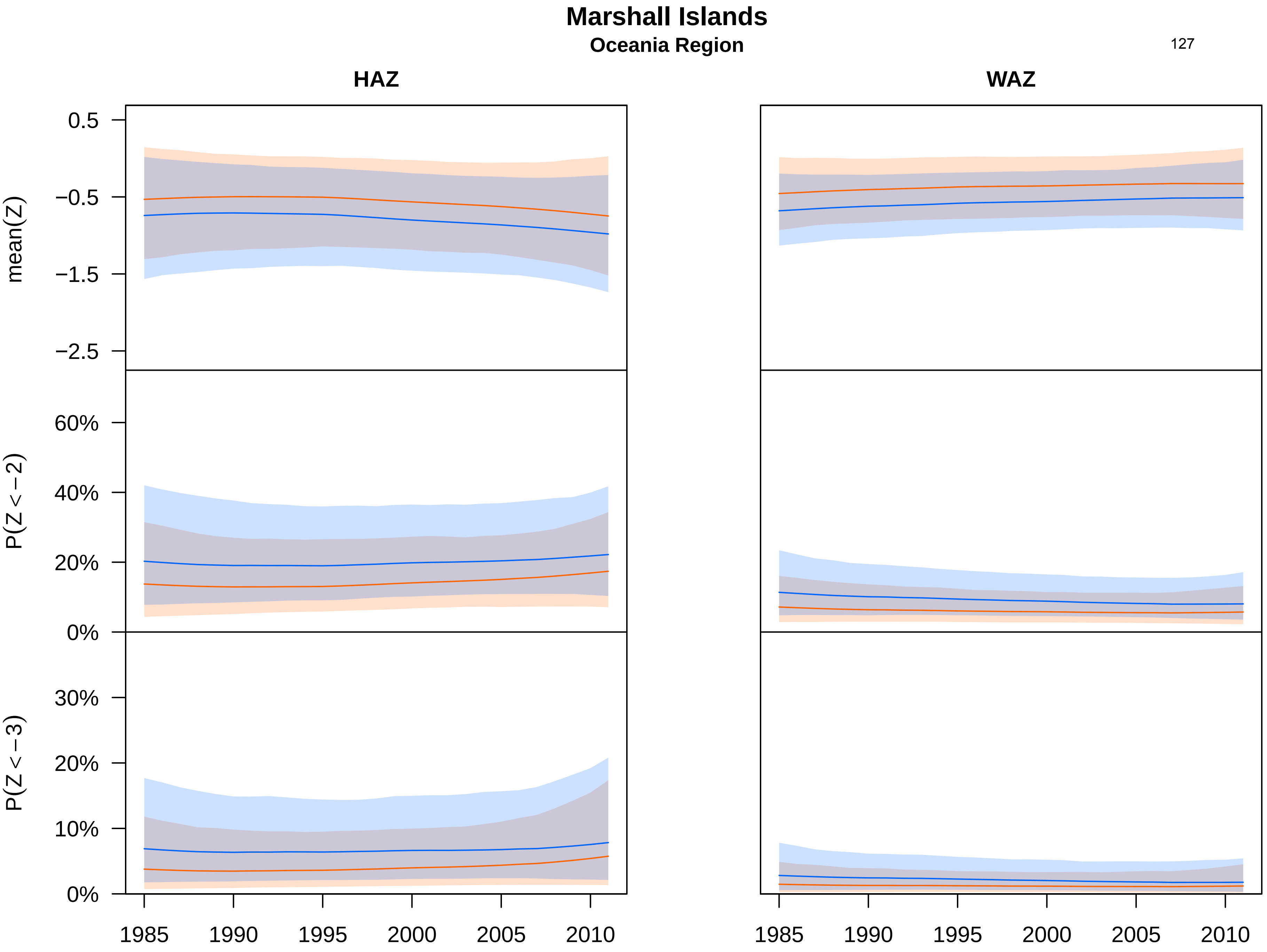
Marshall Islands
Oceania Region
HAZ
WAZ
mean(Z)
P(Z < −2)
P(Z < −3)
0.5
−0.5
−1.5
−2.5
60%
40%
20%
0%
30%
20%
10%
0%
1985
1990
1995
2000
2005
2010

Mauritania

Sub−Saharan Africa Region

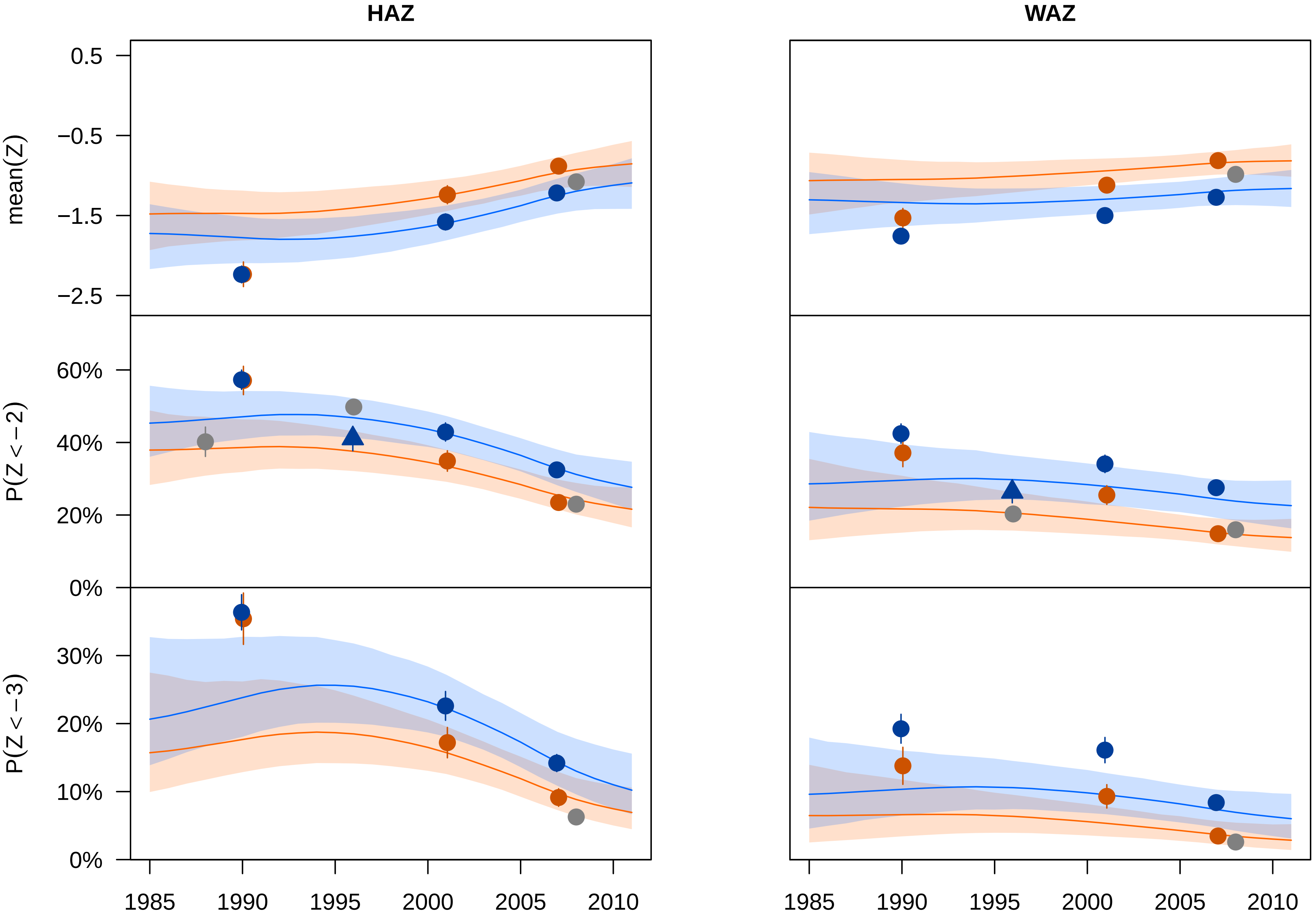

Mauritius

Sub−Saharan Africa Region

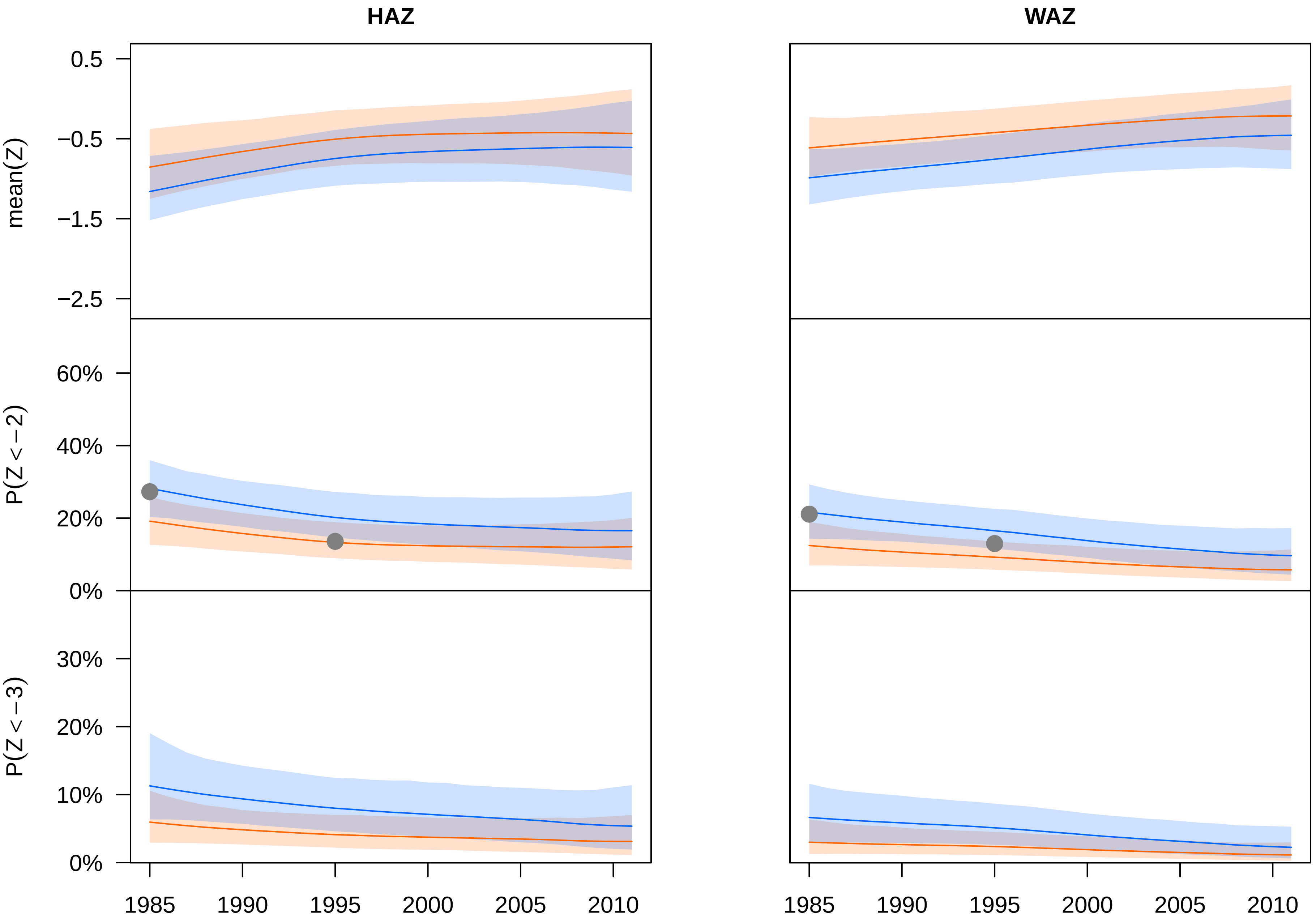

Mexico

Andean and Central Latin America and Caribbean Region

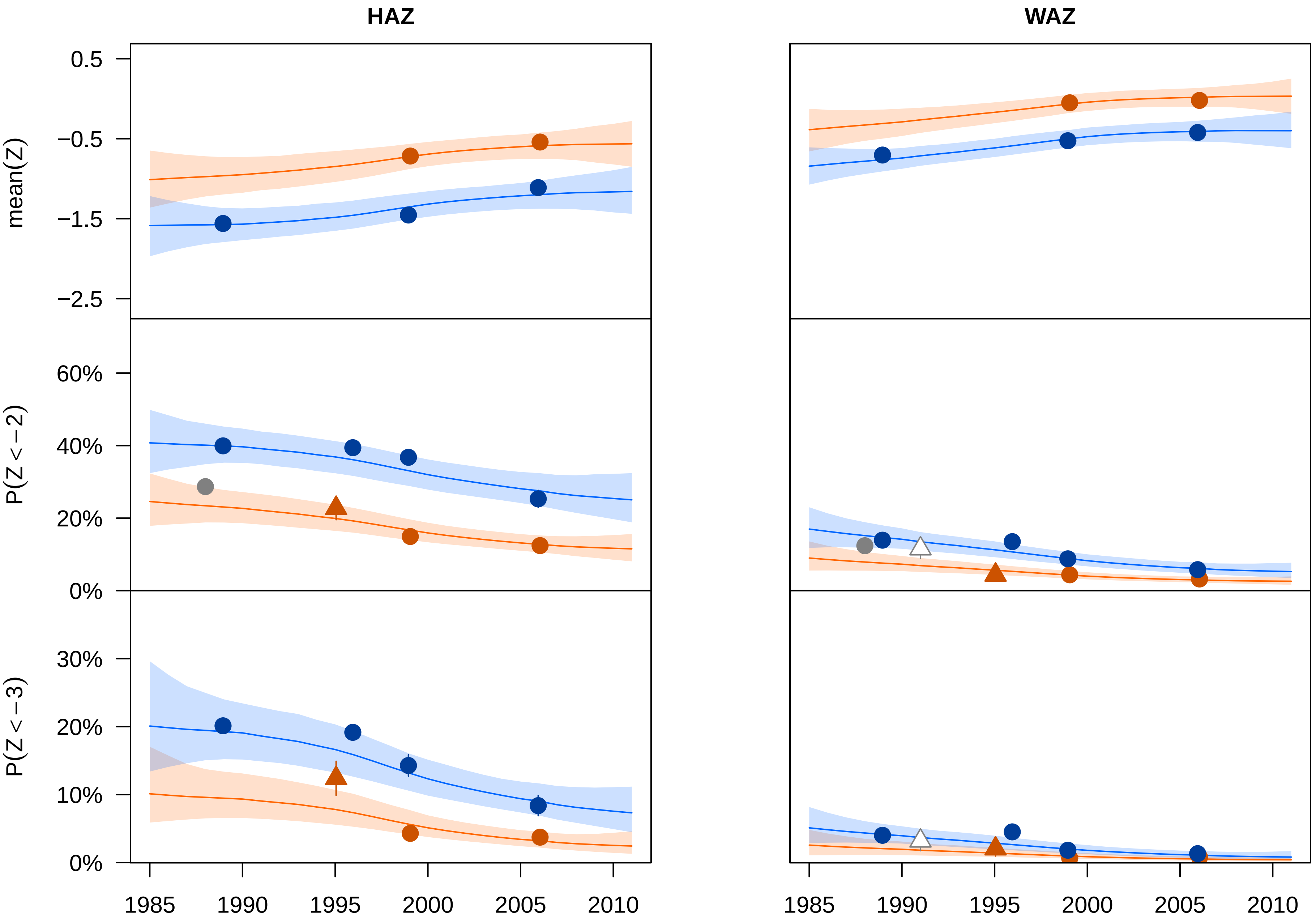

Micronesia (Federated States of)

Oceania Region

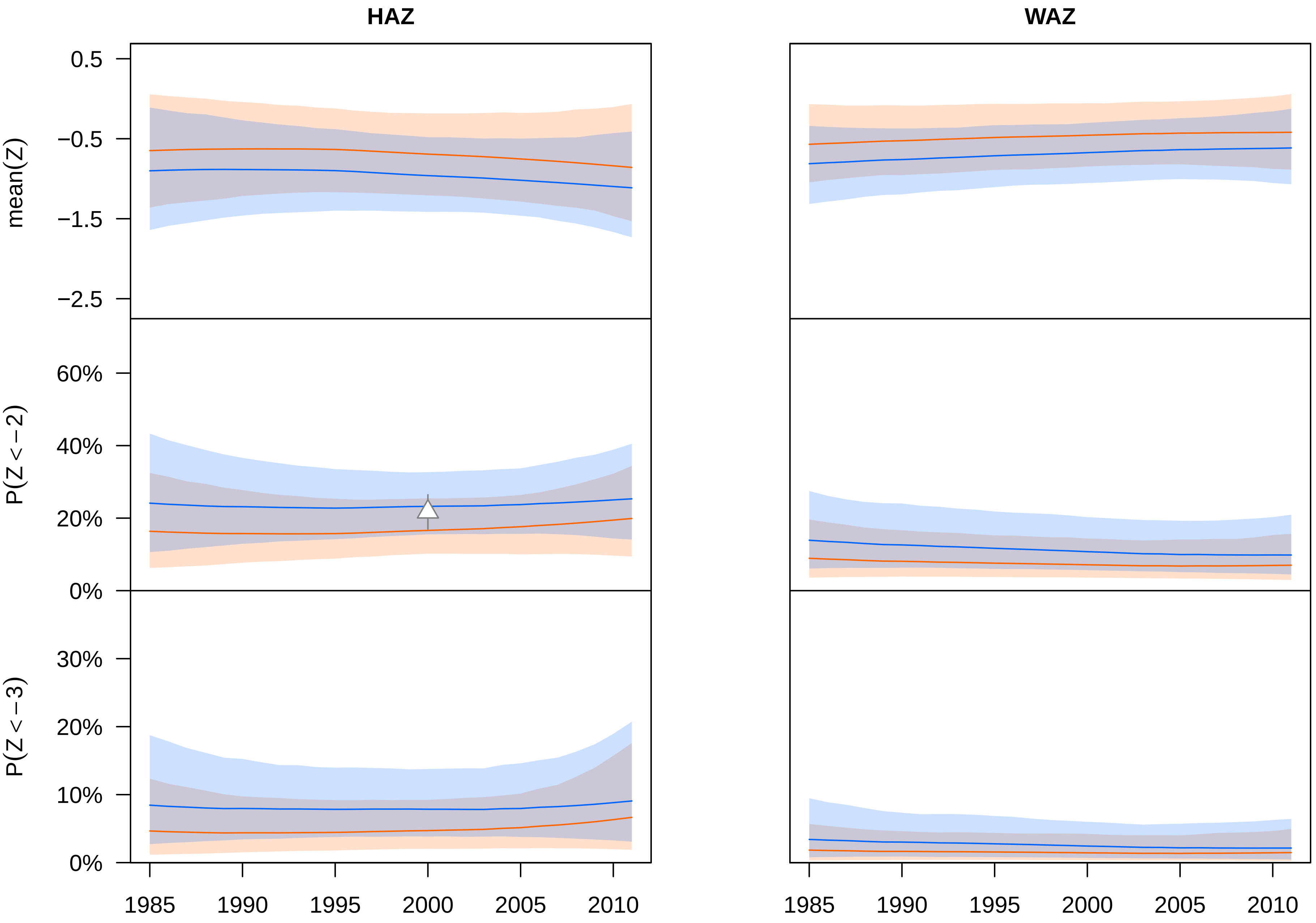

Mongolia

Central Asia, Middle East, and North Africa Region

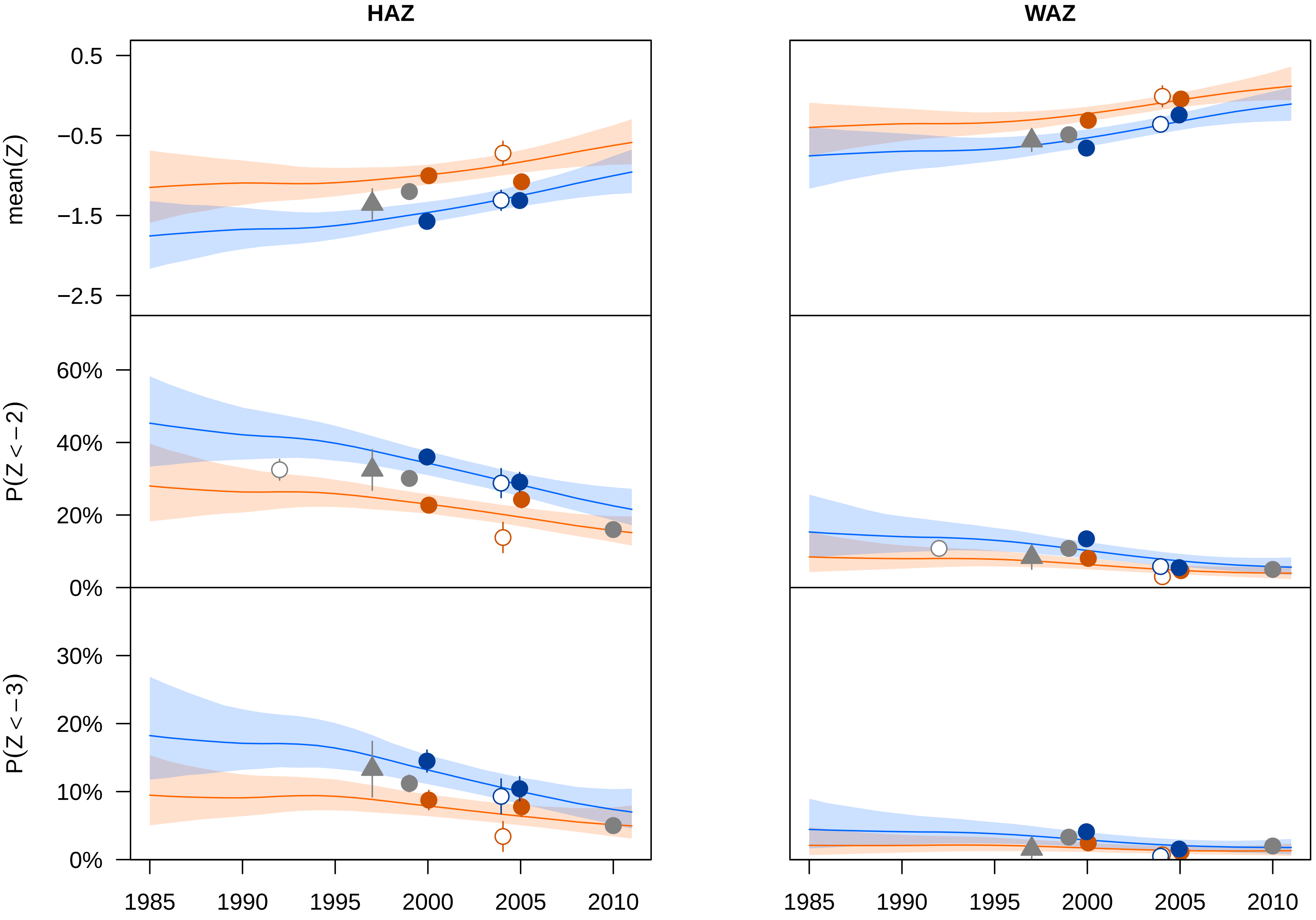

Morocco

Central Asia, Middle East, and North Africa Region

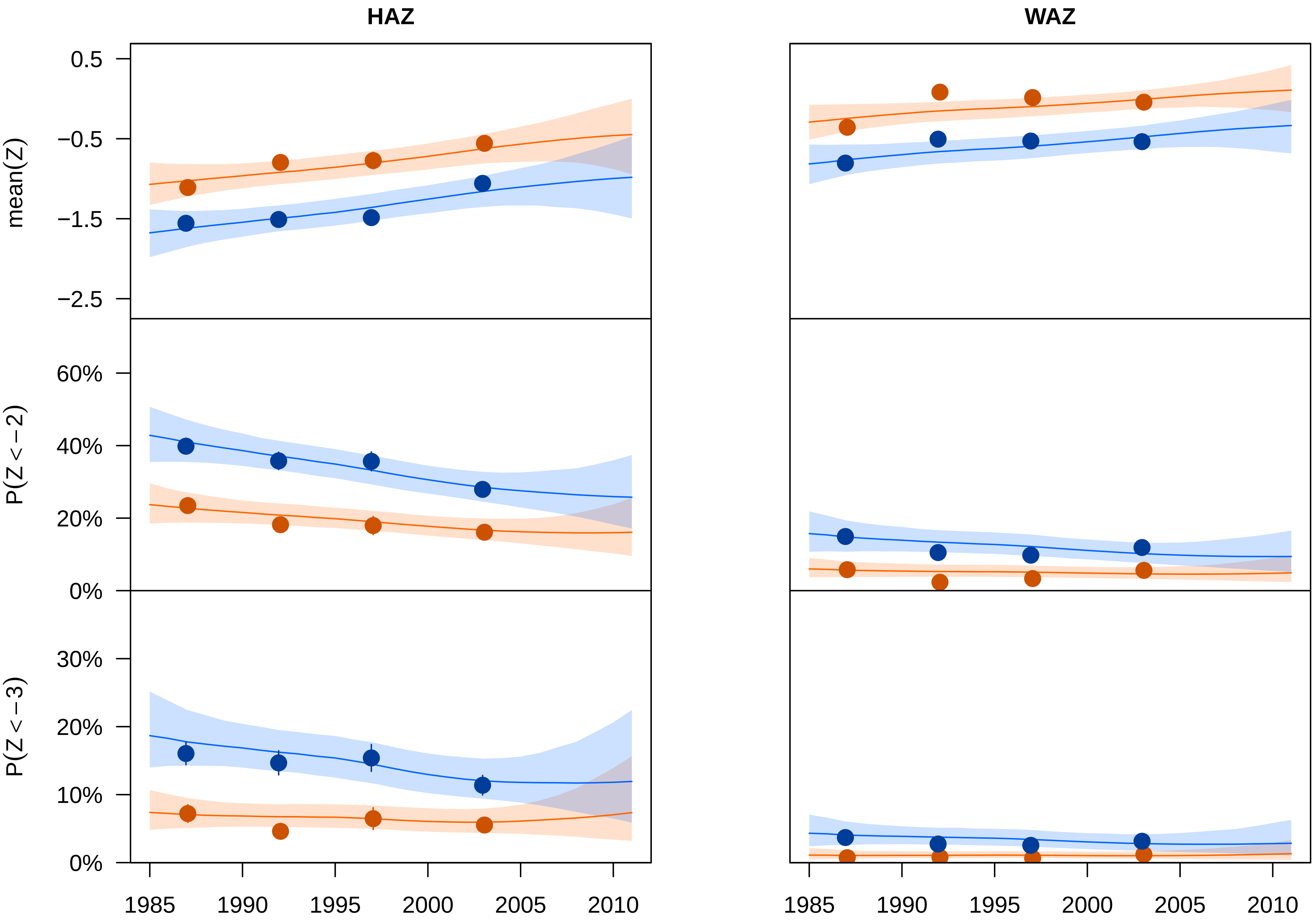

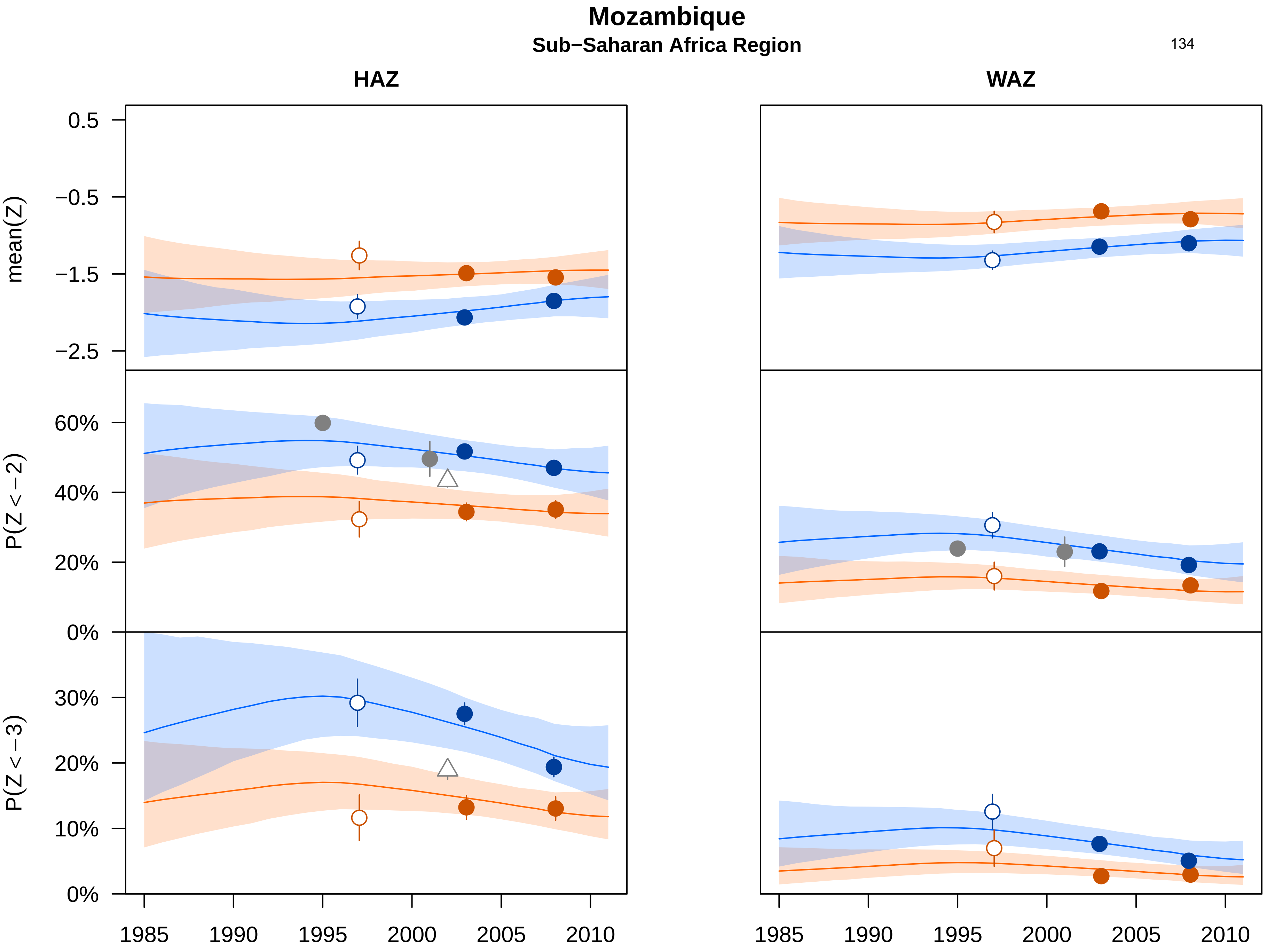
Mozambique
Sub−Saharan Africa Region
HAZ
WAZ
mean(Z)
P(Z < −2)
P(Z < −3)
0.5
−0.5
−1.5
−2.5
60%
40%
20%
0%
30%
20%
10%
0%
1985
1990
1995
2000
2005
2010

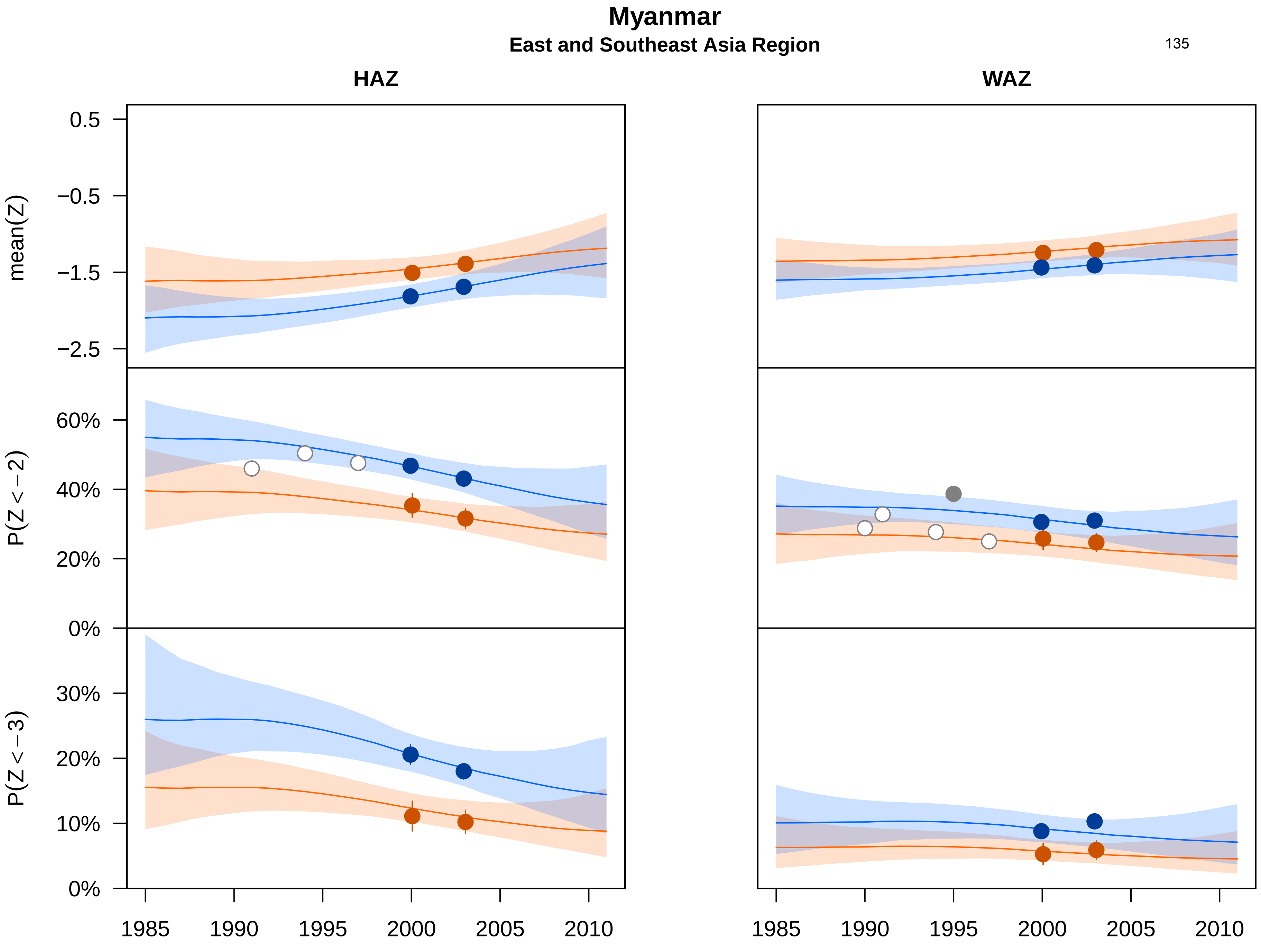
Myanmar
East and Southeast Asia Region
HAZ
WAZ
mean(Z)
P(Z < −2)
P(Z < −3)
0.5
−0.5
−1.5
−2.5
60%
40%
20%
0%
30%
20%
10%
0%
1985
1990
1995
2000
2005
2010

Namibia

Sub−Saharan Africa Region

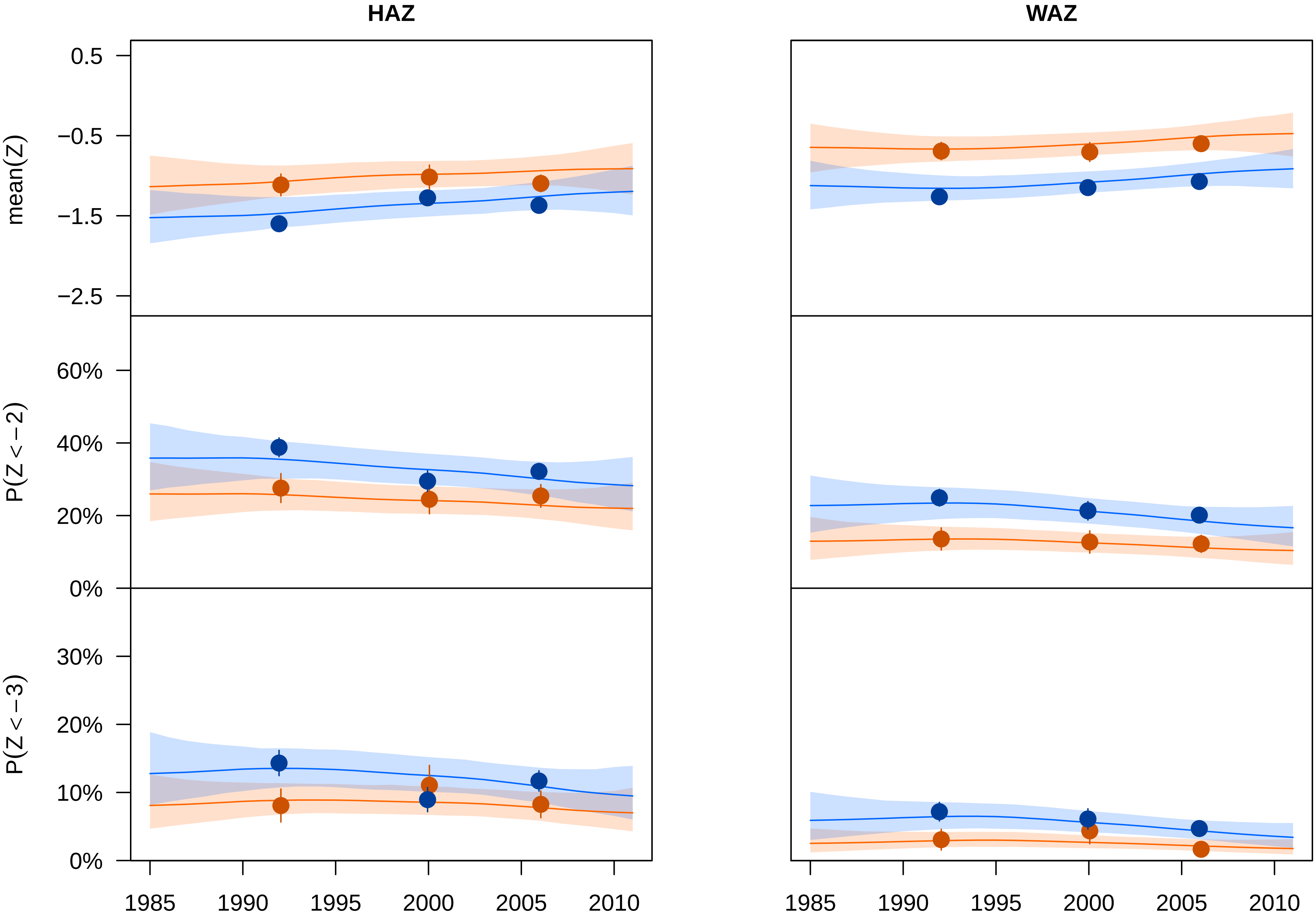

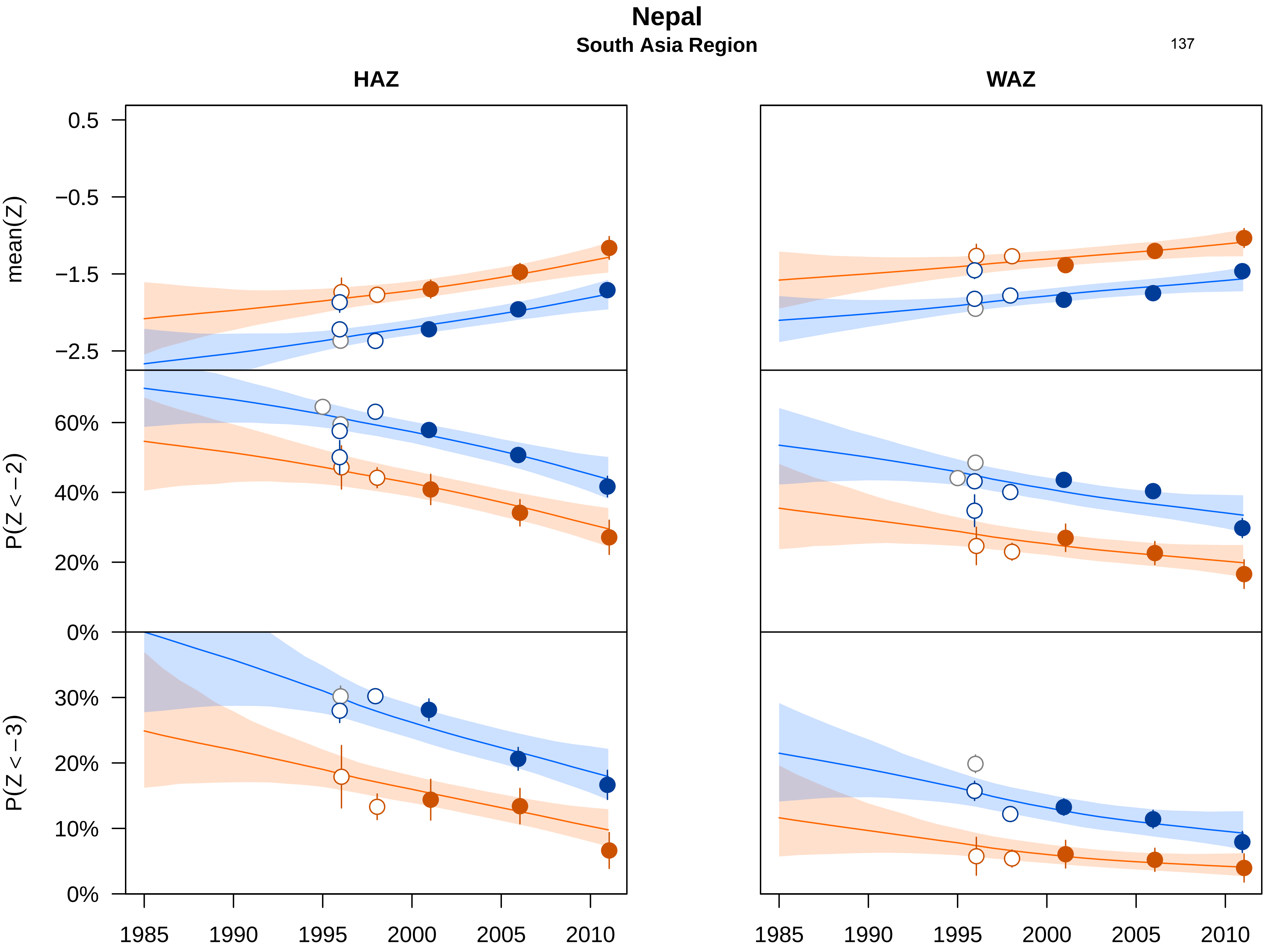
Nepal
South Asia Region
HAZ
WAZ
mean(Z)
P(Z < −2)
P(Z < −3)
0.5
−0.5
−1.5
−2.5
60%
40%
20%
0%
30%
20%
10%
0%
1985
1990
1995
2000
2005
2010

Nicaragua

Andean and Central Latin America and Caribbean Region

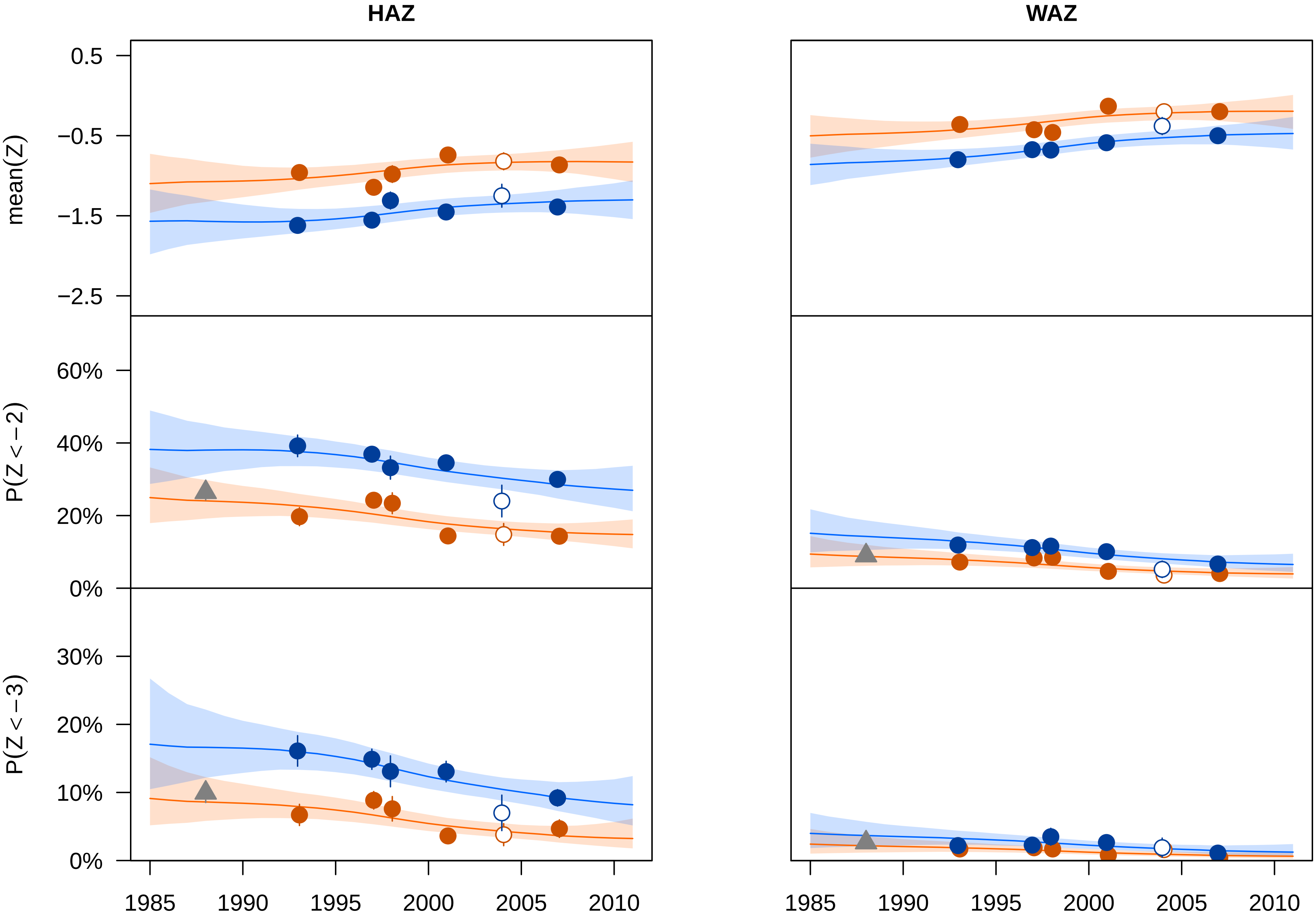

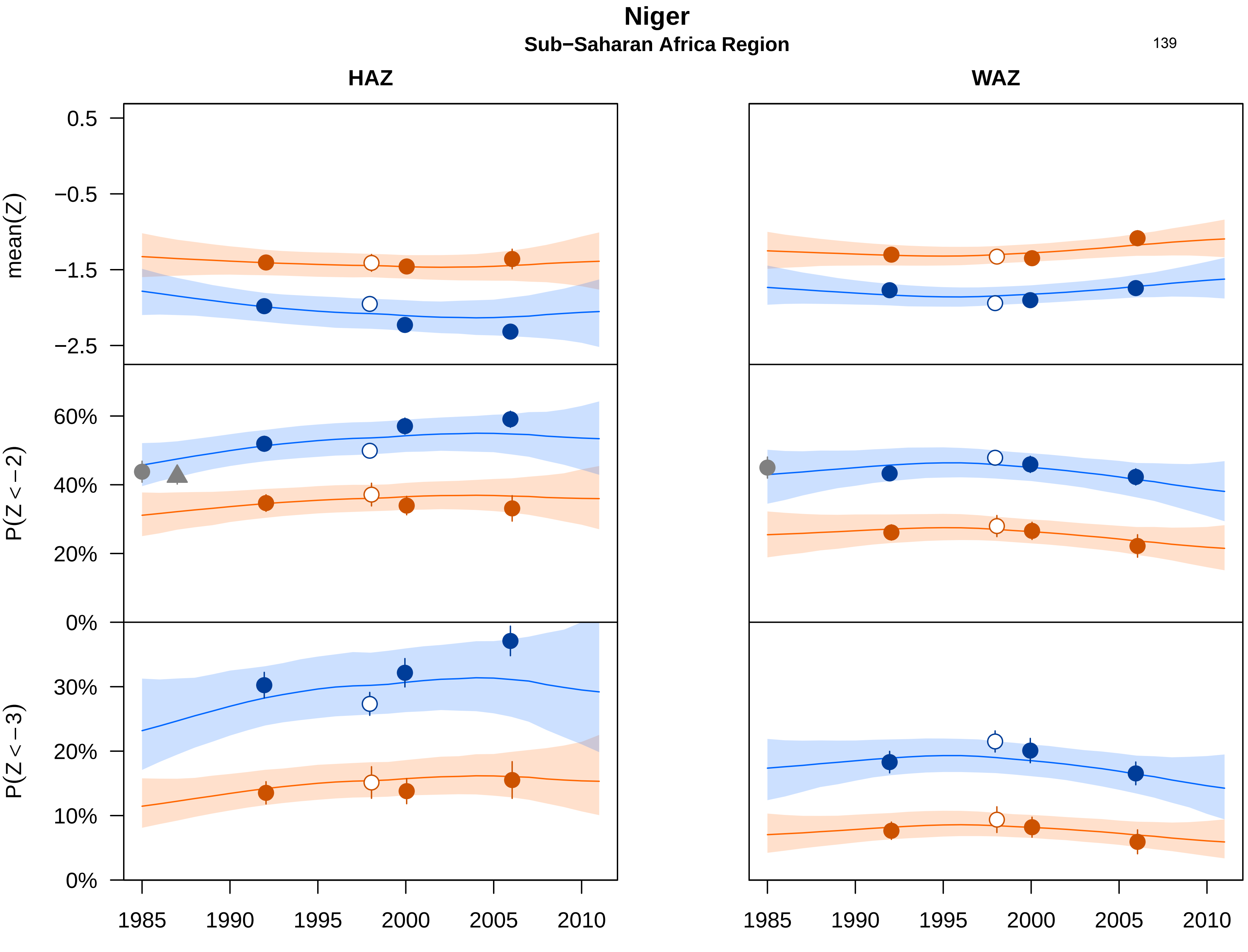
Niger
Sub−Saharan Africa Region
HAZ
WAZ
mean(Z)
P(Z < −2)
P(Z < −3)
0.5
−0.5
−1.5
−2.5
60%
40%
20%
0%
30%
20%
10%
0%
1985
1990
1995
2000
2005
2010

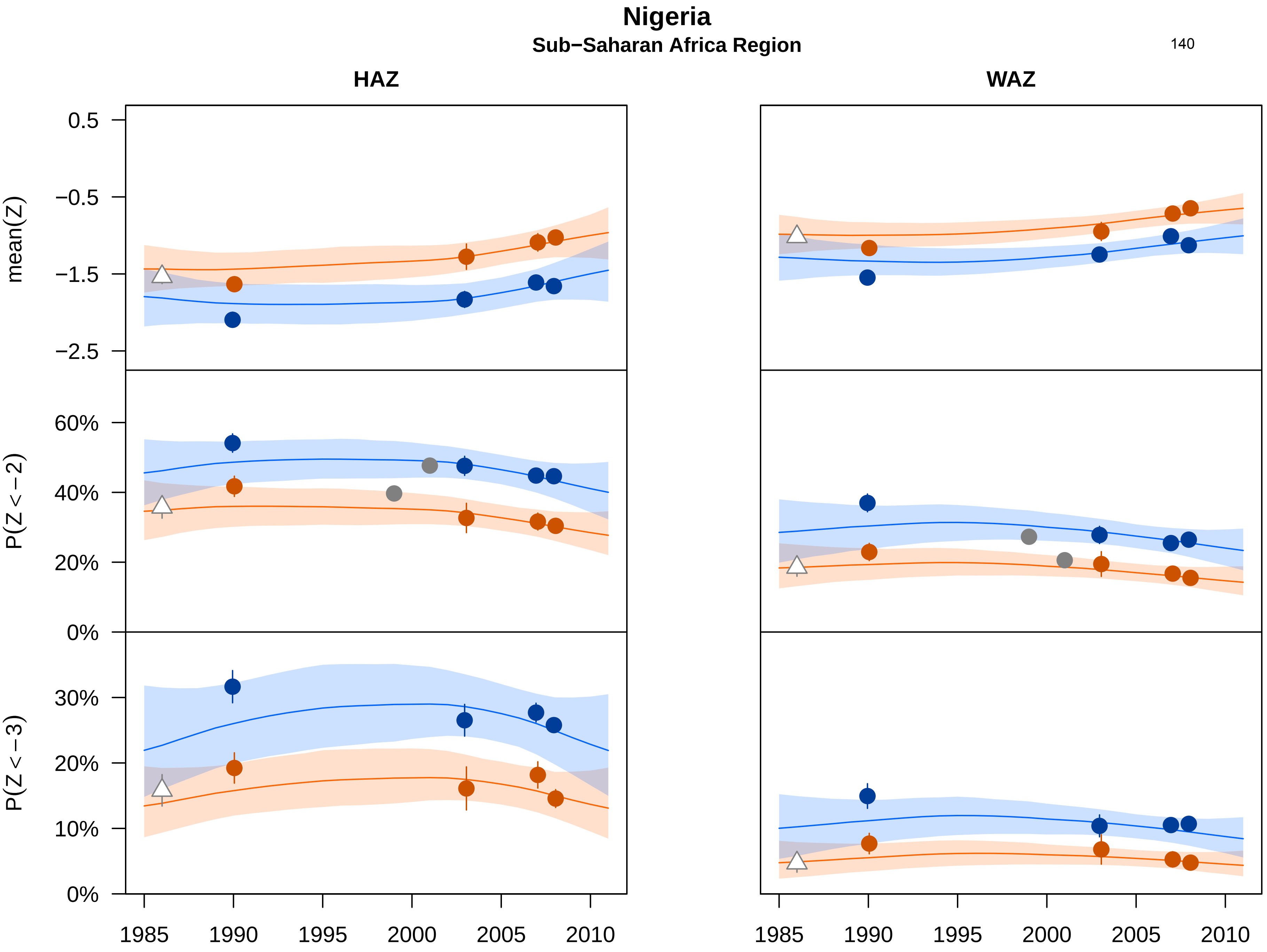

Nigeria
Sub−Saharan Africa Region
HAZ
WAZ
mean(Z)
P(Z < −2)
P(Z < −3)
0.5
−0.5
−1.5
−2.5
60%
40%
20%
0%
30%
20%
10%
0%
1985
1990
1995
2000
2005
2010

Occupied Palestinian Territory

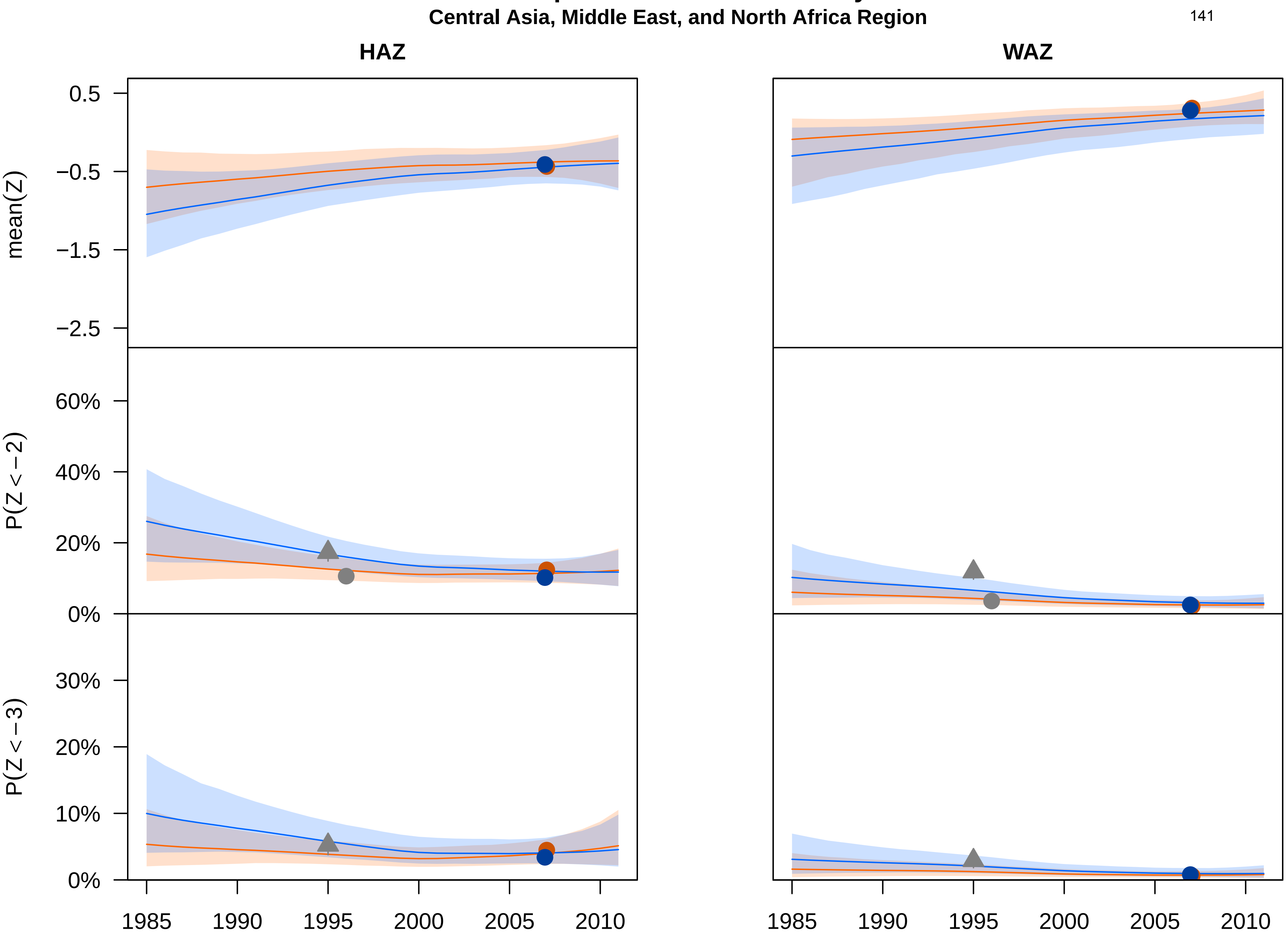

Oman

Central Asia, Middle East, and North Africa Region

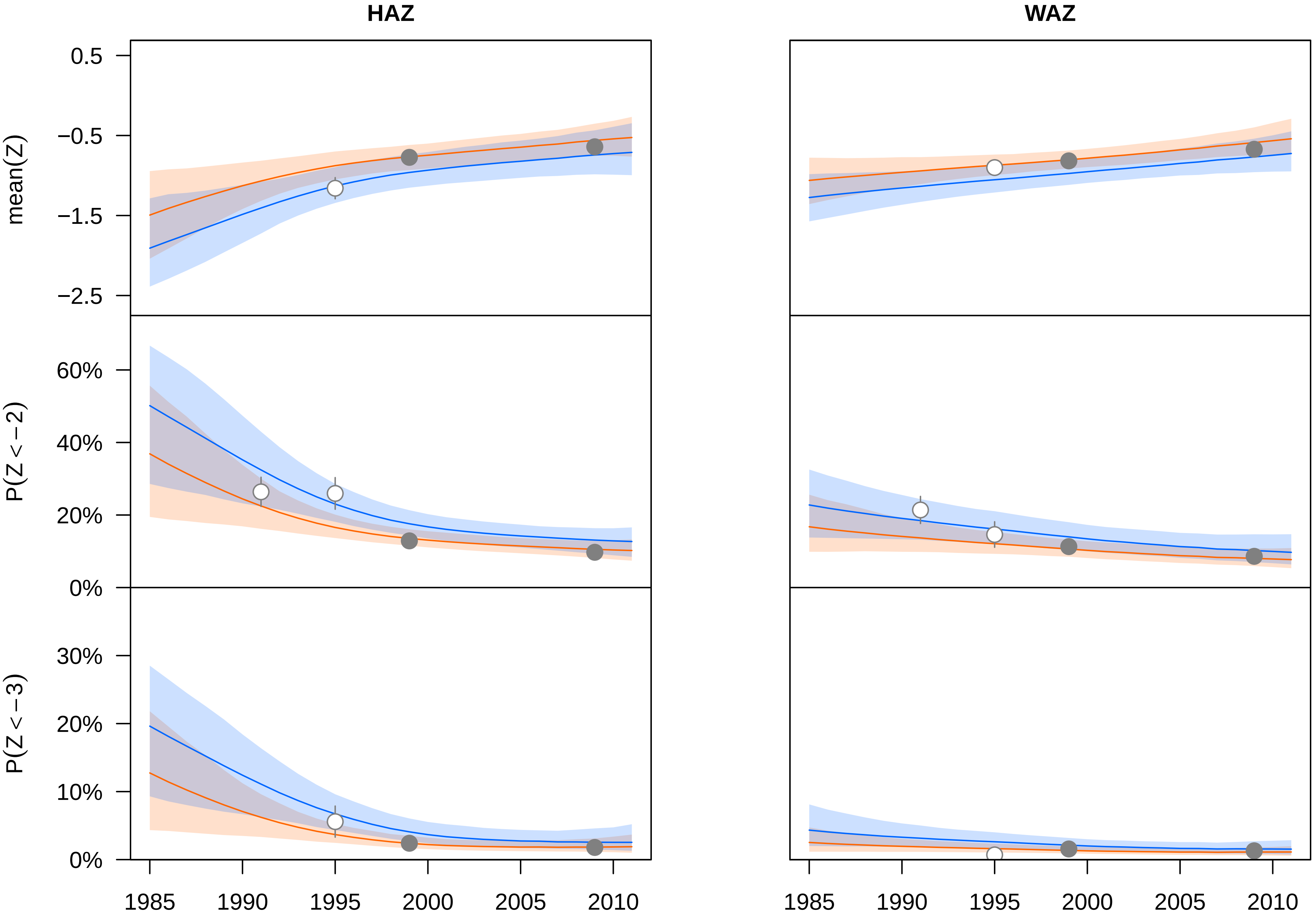

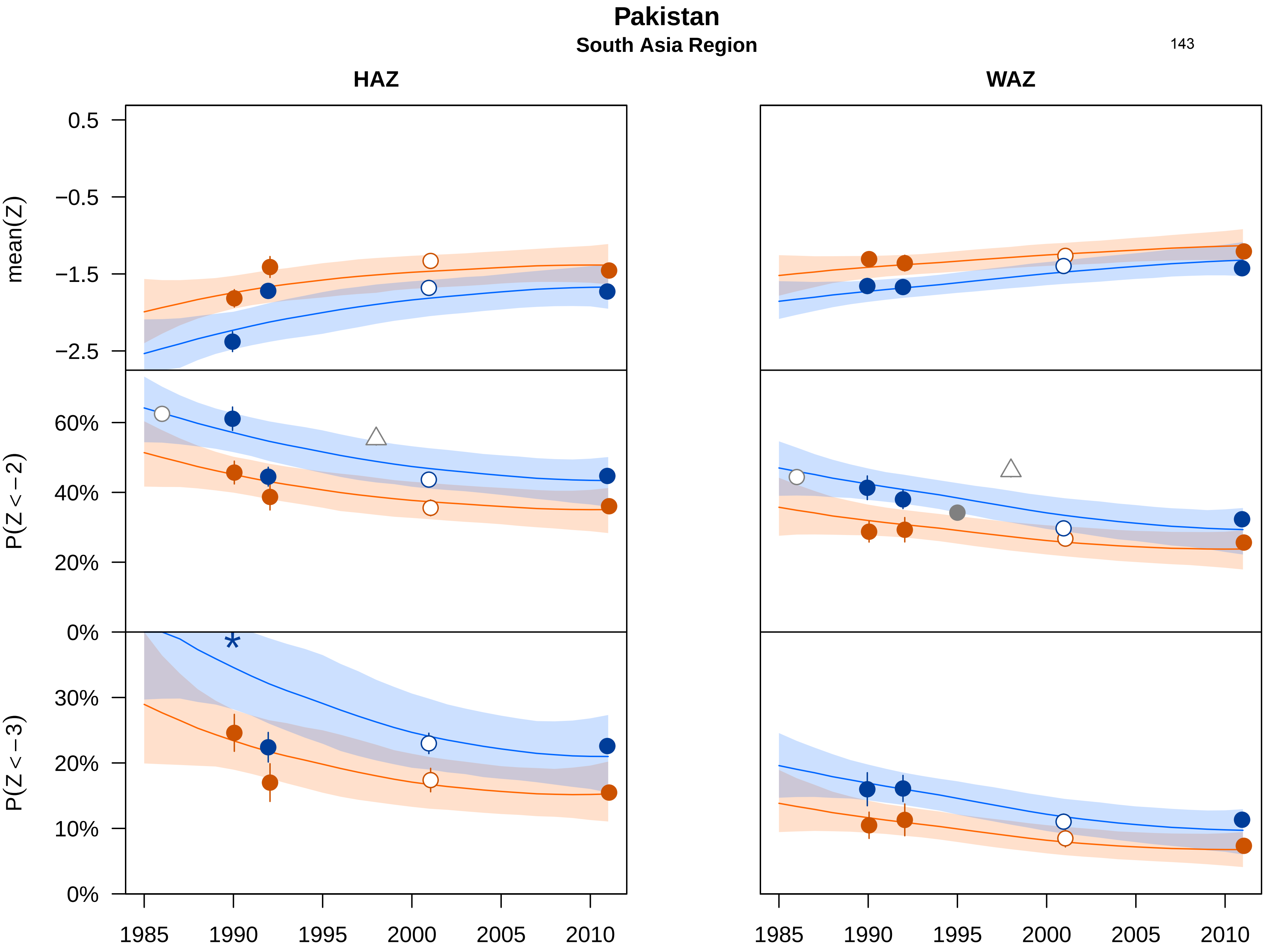
Pakistan
South Asia Region
HAZ
WAZ
mean(Z)
P(Z < −2)
P(Z < −3)
0.5
−0.5
−1.5
−2.5
60%
40%
20%
0%
30%
20%
10%
0%
1985
1990
1995
2000
2005
2010

Panama

Andean and Central Latin America and Caribbean Region

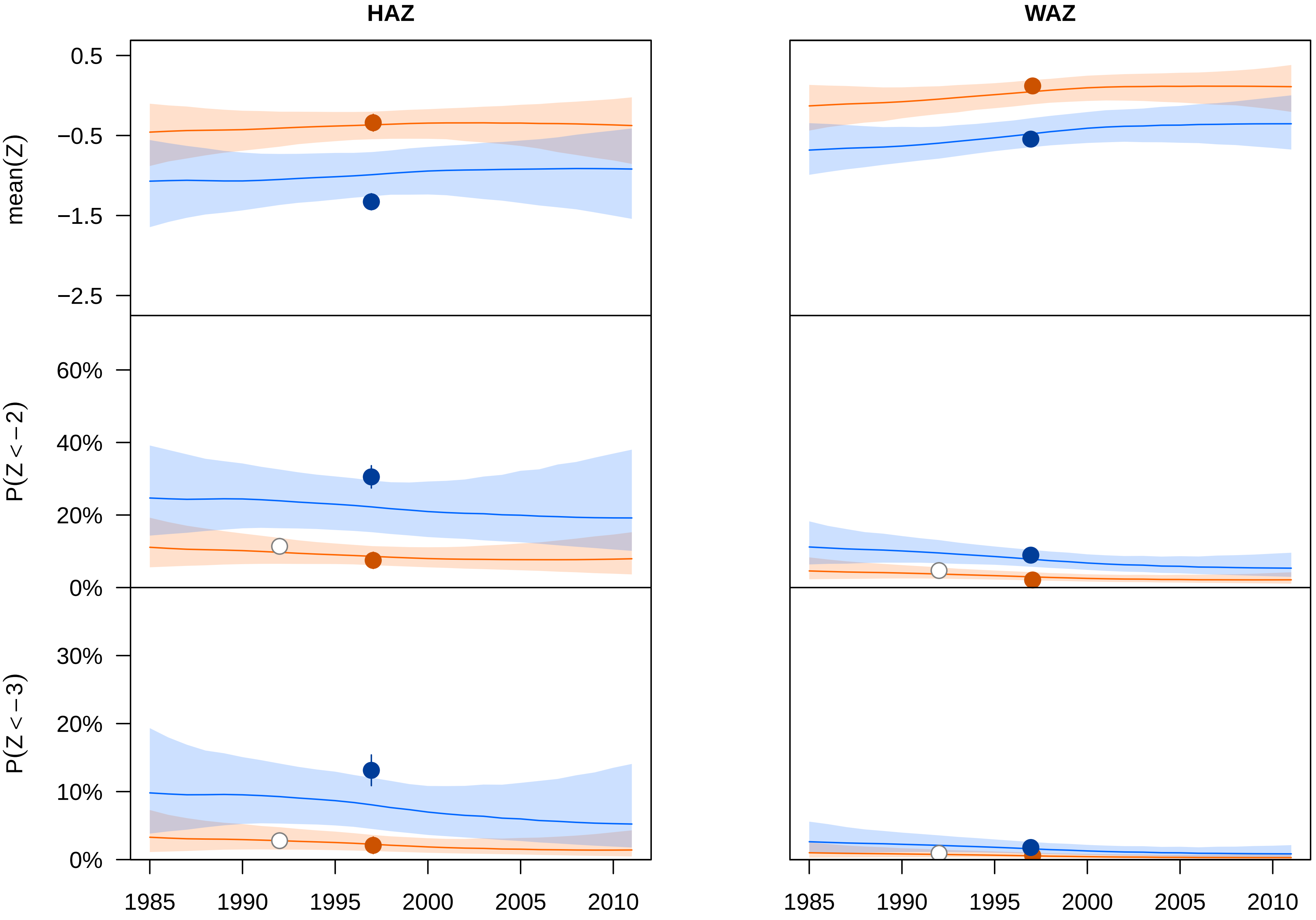

Papua New Guinea

Oceania Region

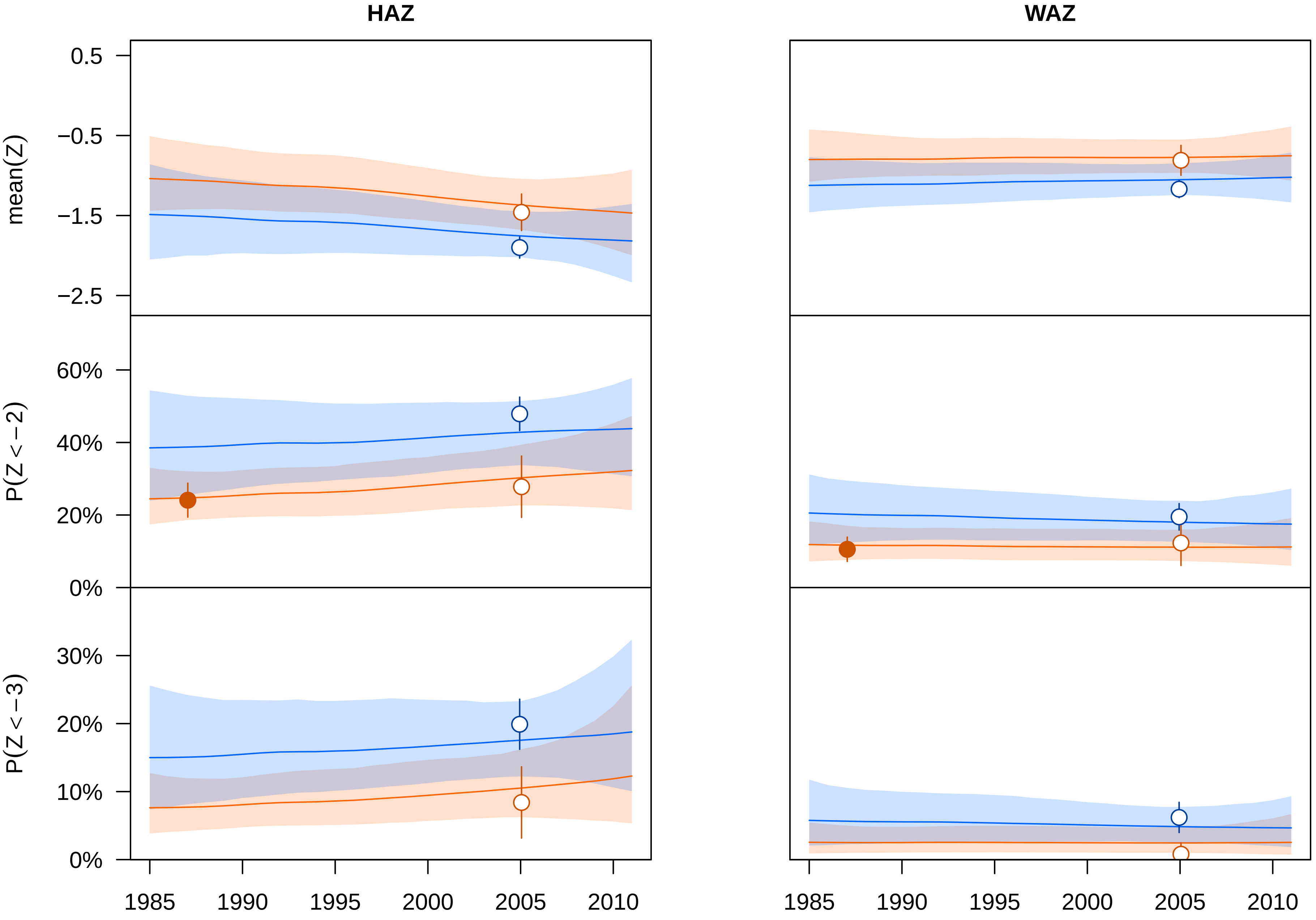

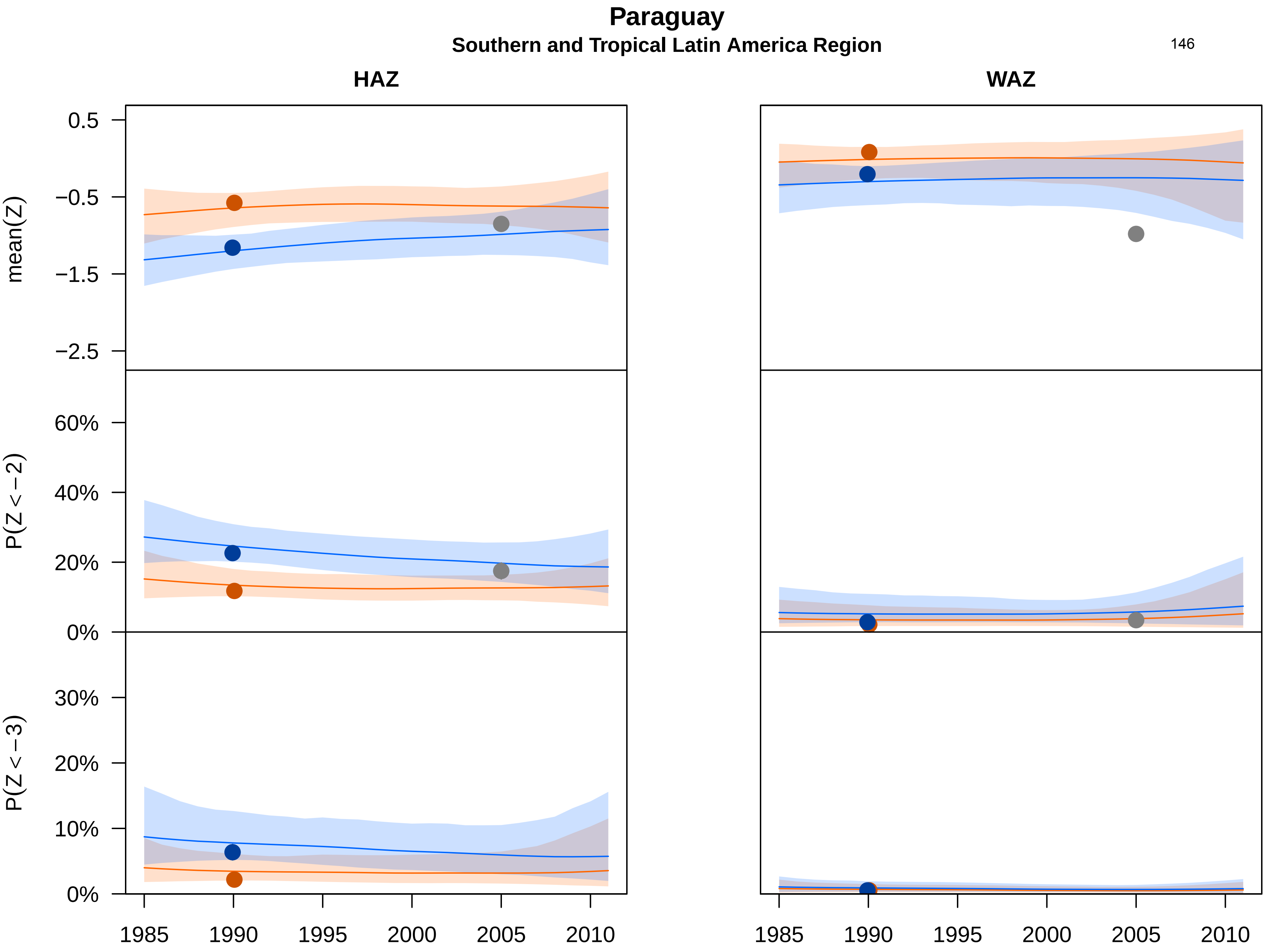
Paraguay
Southern and Tropical Latin America Region
HAZ
WAZ
mean(Z)
P(Z < −2)
P(Z < −3)
0.5
−0.5
−1.5
−2.5
60%
40%
20%
0%
30%
20%
10%
0%
1985
1990
1995
2000
2005
2010

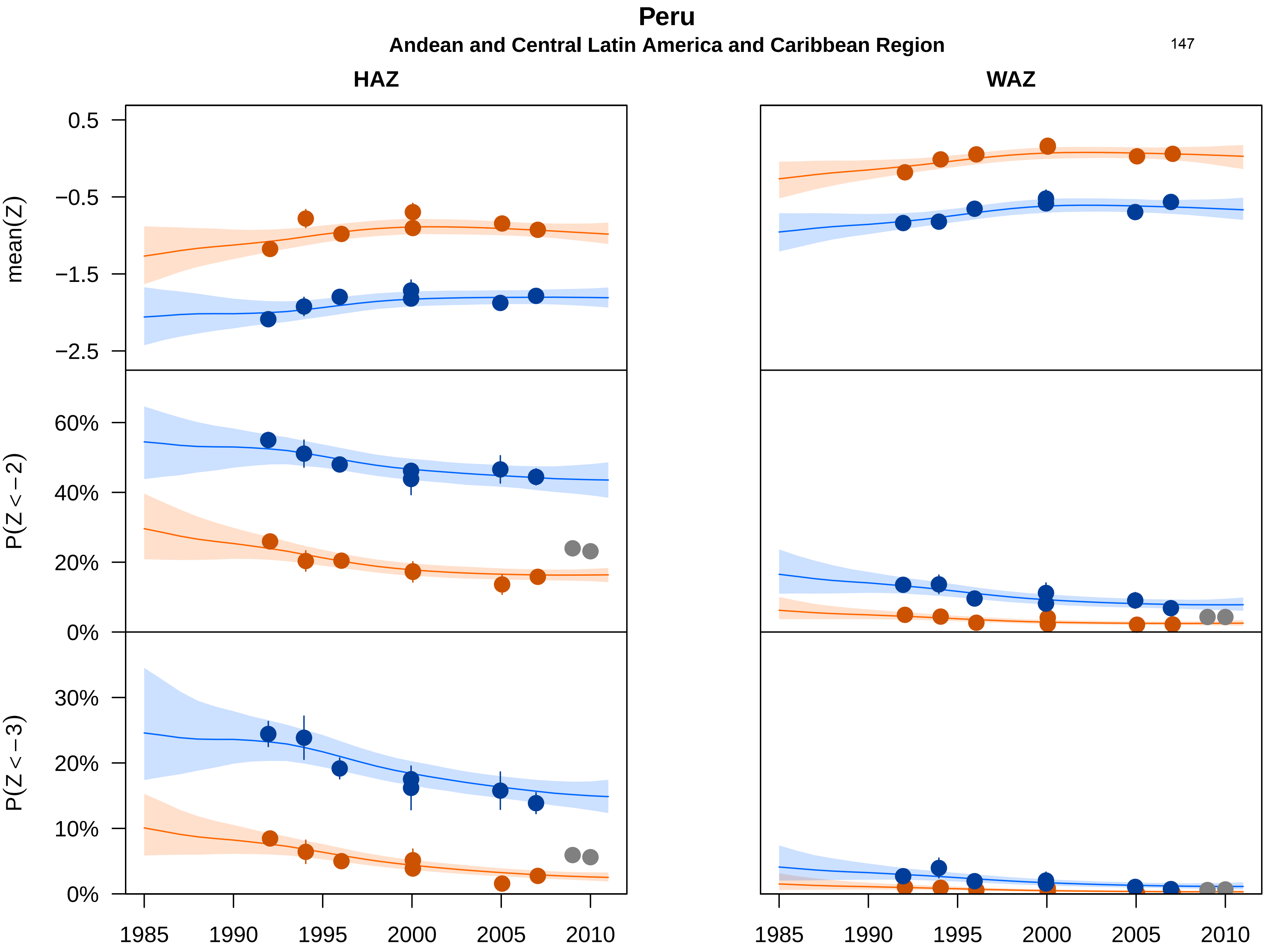
Peru
Andean and Central Latin America and Caribbean Region
HAZ
WAZ
mean(Z)
P(Z < −2)
P(Z < −3)
0.5
−0.5
−1.5
−2.5
60%
40%
20%
0%
30%
20%
10%
0%
1985
1990
1995
2000
2005
2010

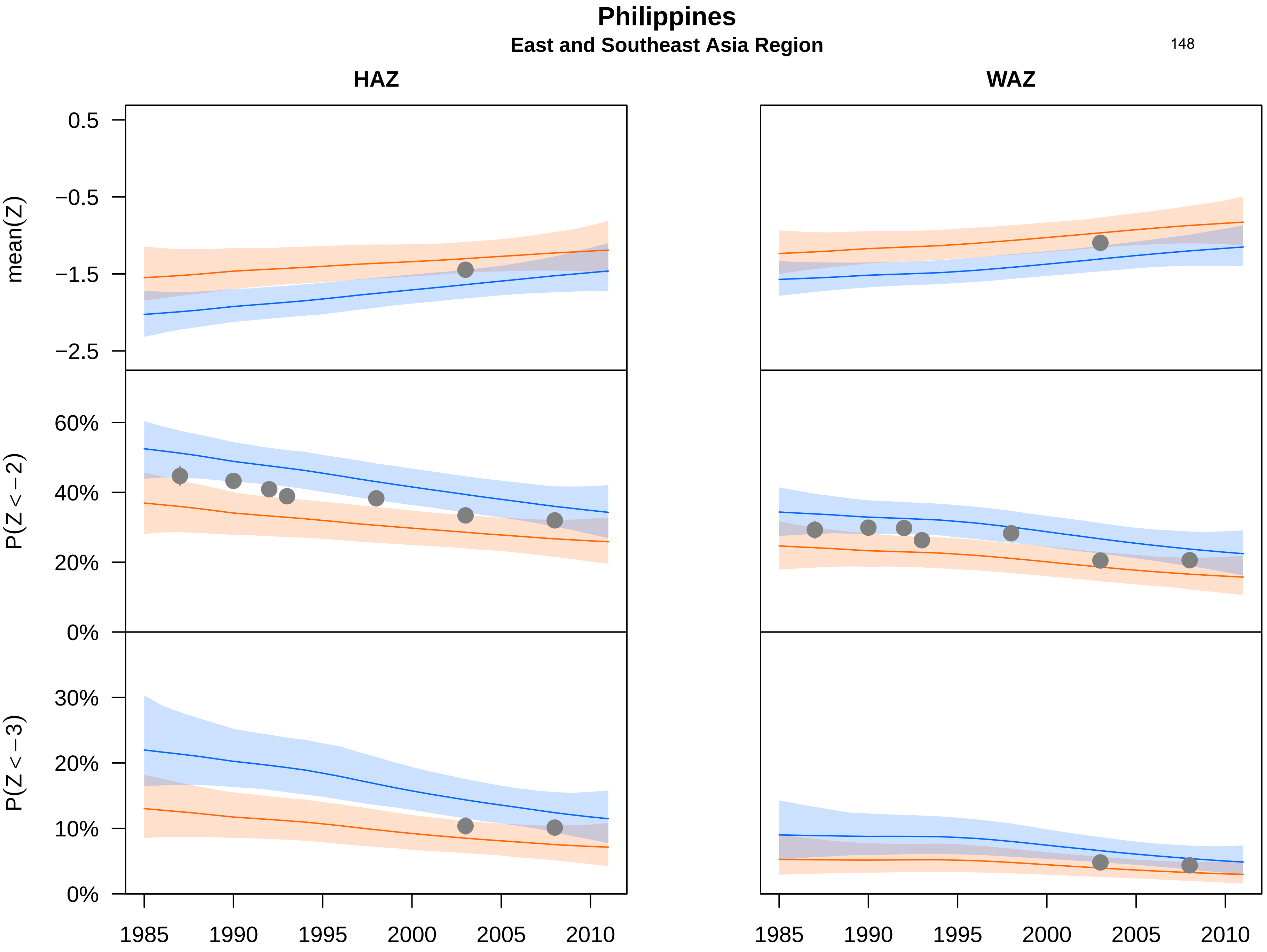

Philippines
East and Southeast Asia Region
HAZ
WAZ
mean(Z)
P(Z < −2)
P(Z < −3)
0.5
−0.5
−1.5
−2.5
60%
40%
20%
0%
30%
20%
10%
0%
1985
1990
1995
2000
2005
2010

Puerto Rico

Andean and Central Latin America and Caribbean Region

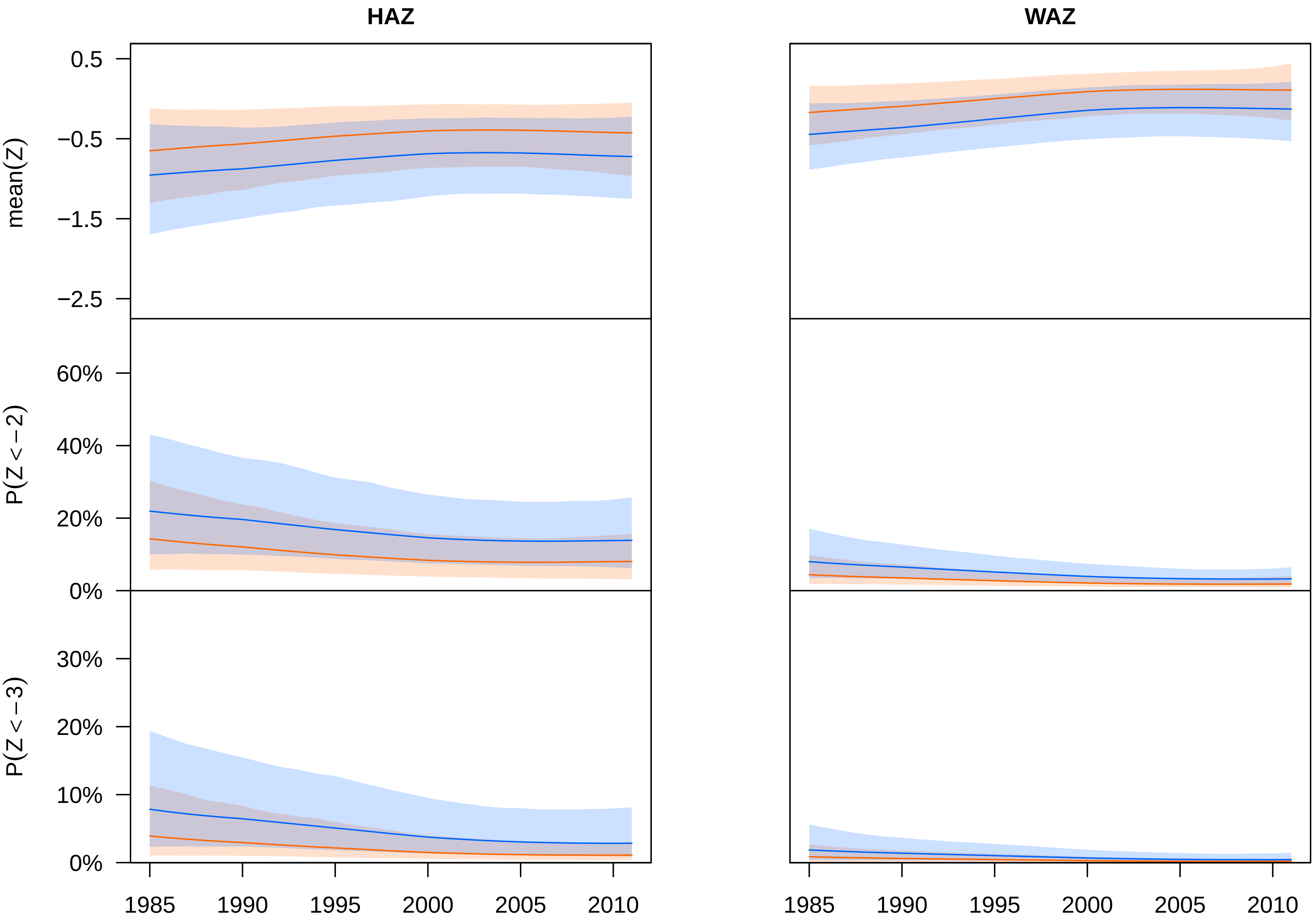

Qatar

Central Asia, Middle East, and North Africa Region

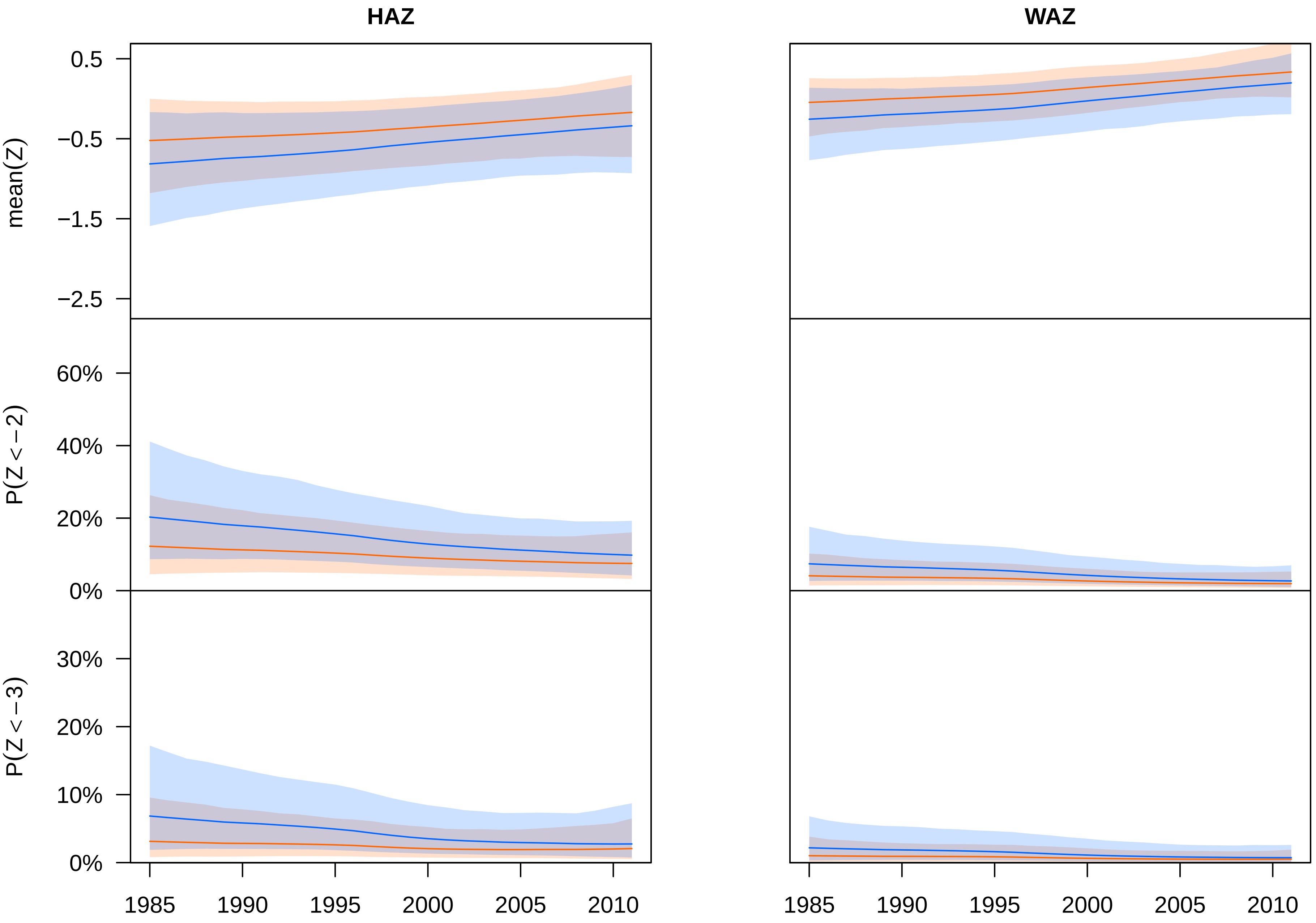

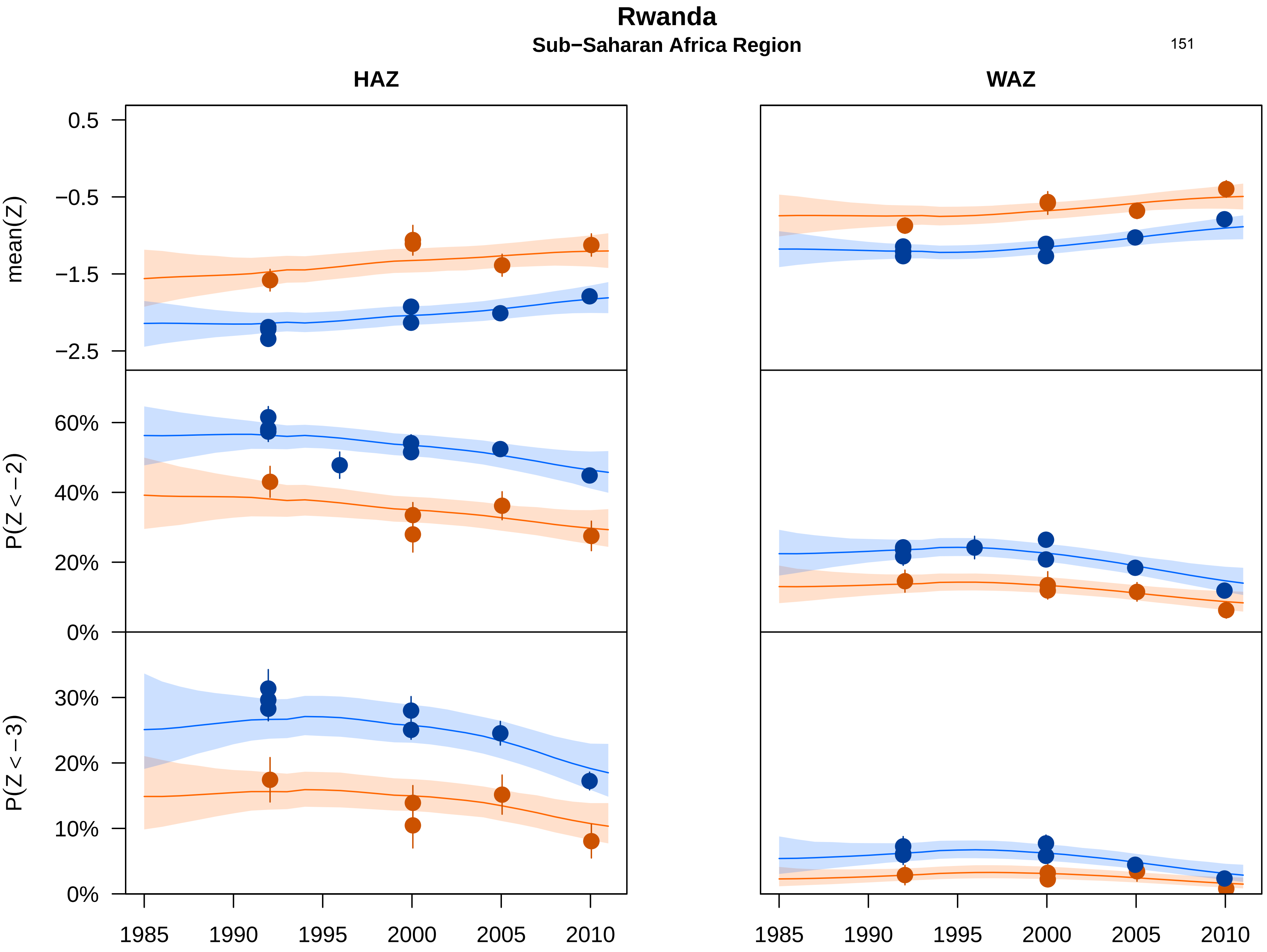
Rwanda
Sub−Saharan Africa Region
HAZ
WAZ
mean(Z)
P(Z < −2)
P(Z < −3)
0.5
−0.5
−1.5
−2.5
60%
40%
20%
0%
30%
20%
10%
0%
1985
1990
1995
2000
2005
2010

Saint Lucia

Andean and Central Latin America and Caribbean Region

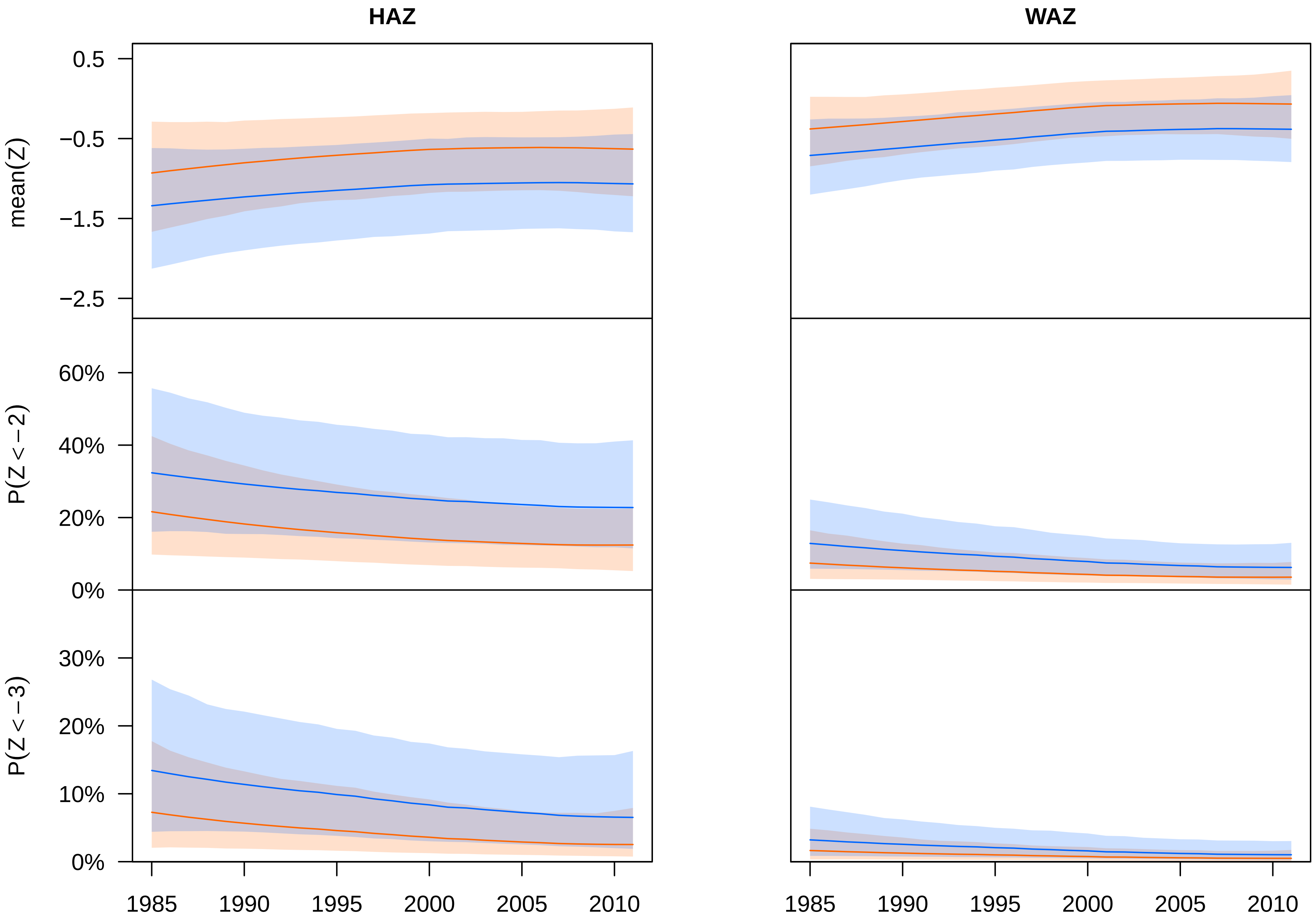

Saint Vincent and the Grenadines

Andean and Central Latin America and Caribbean Region

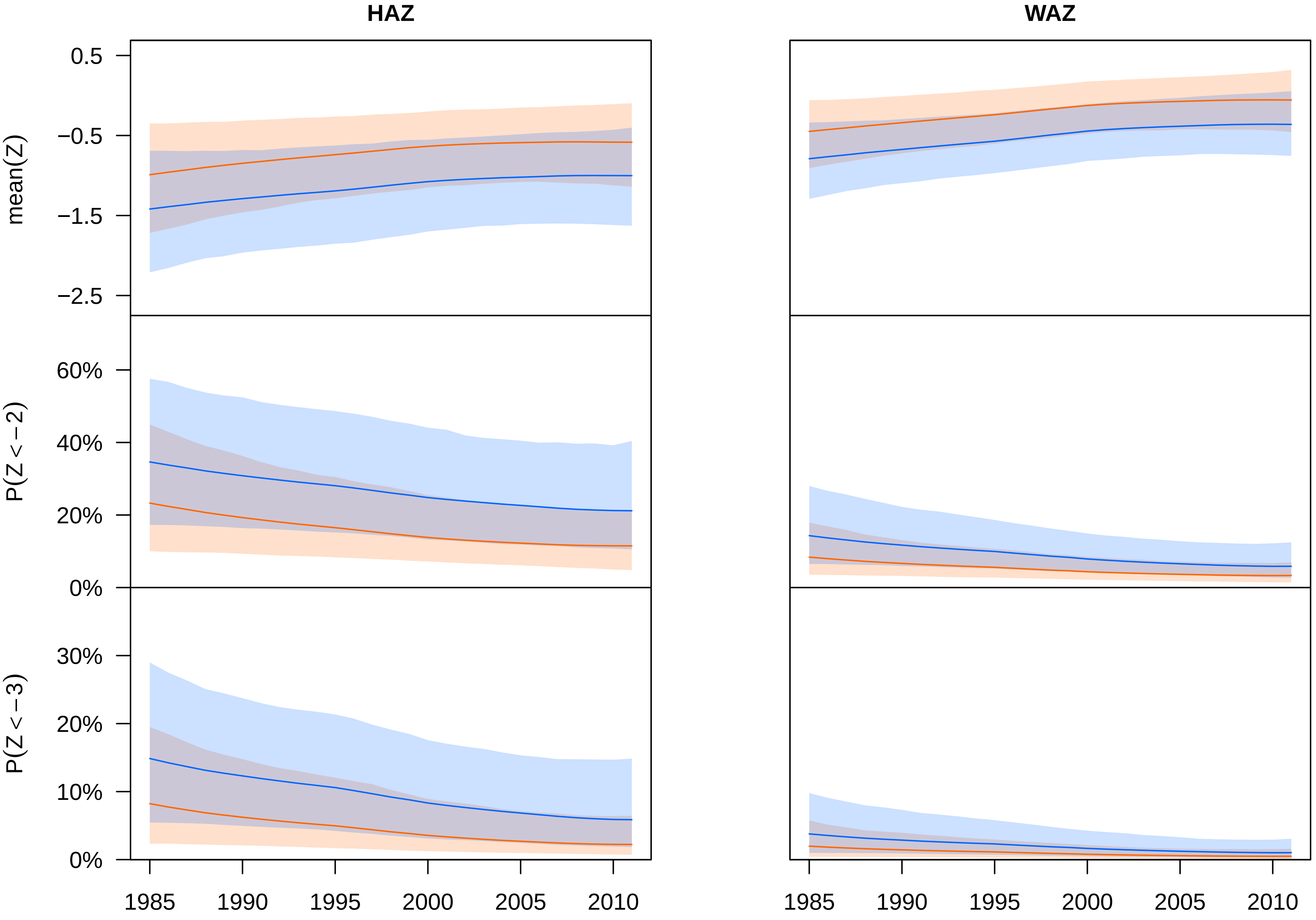

Samoa
Oceania Region

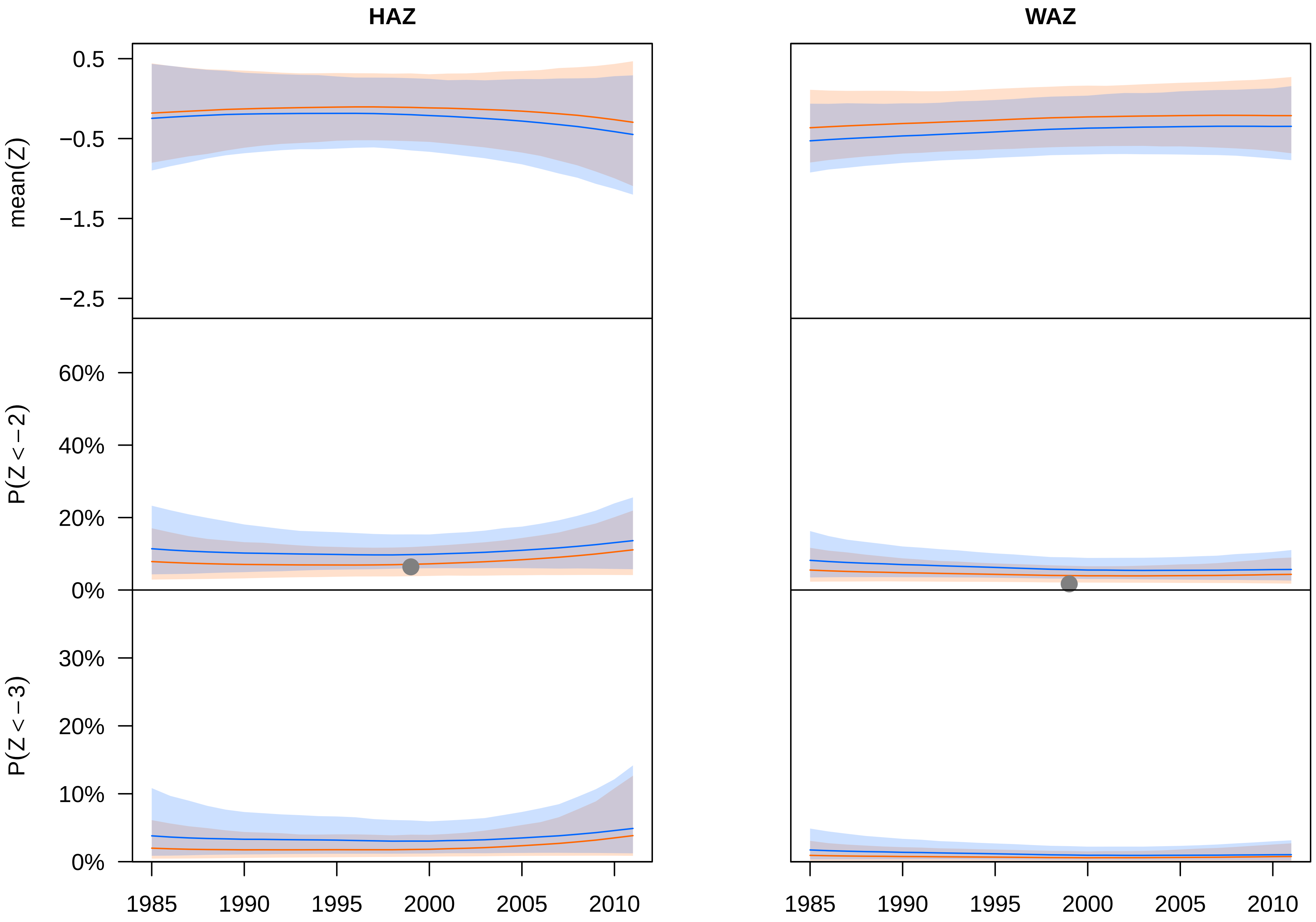

São Tomé and Príncipe
Sub−Saharan Africa Region

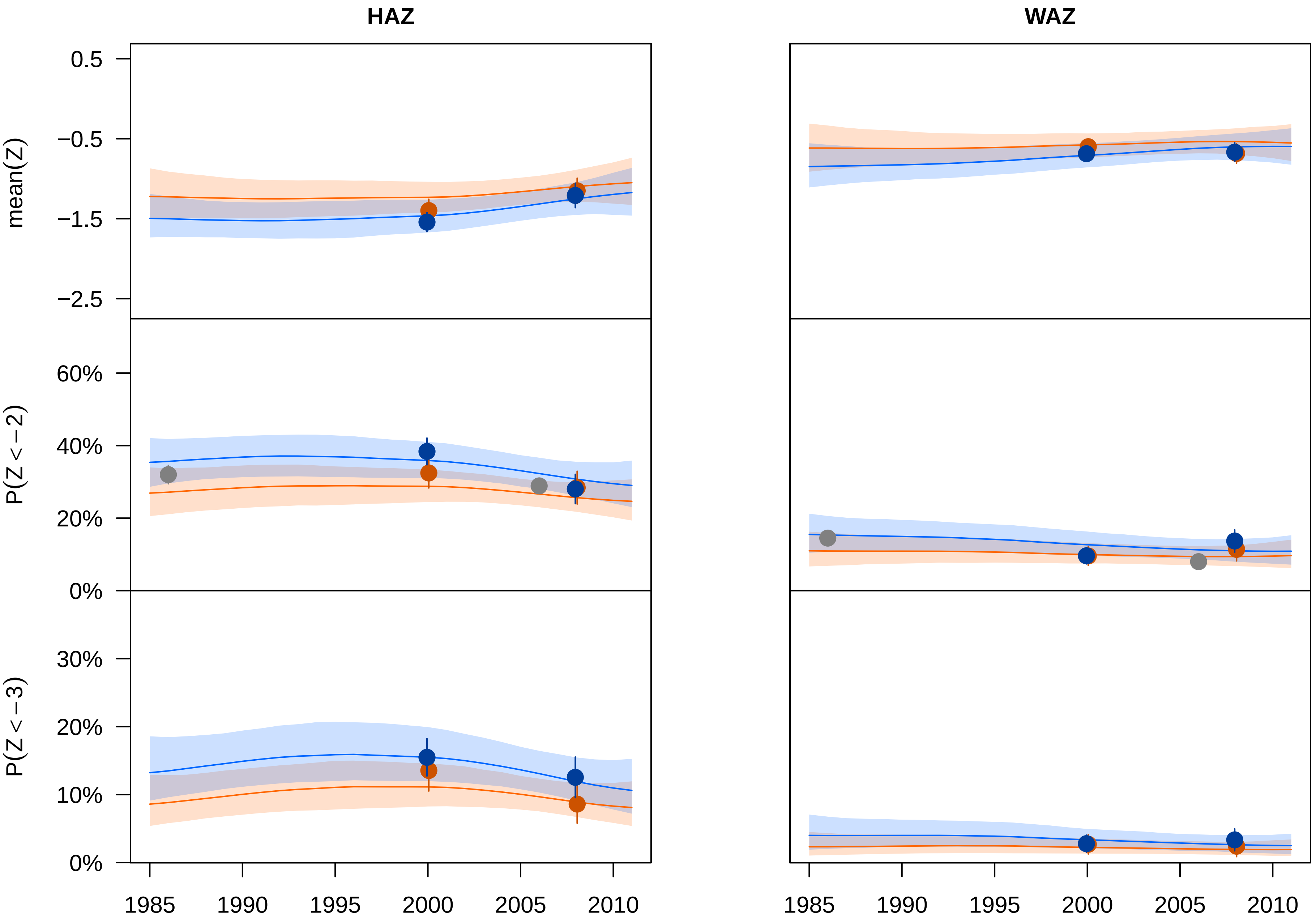

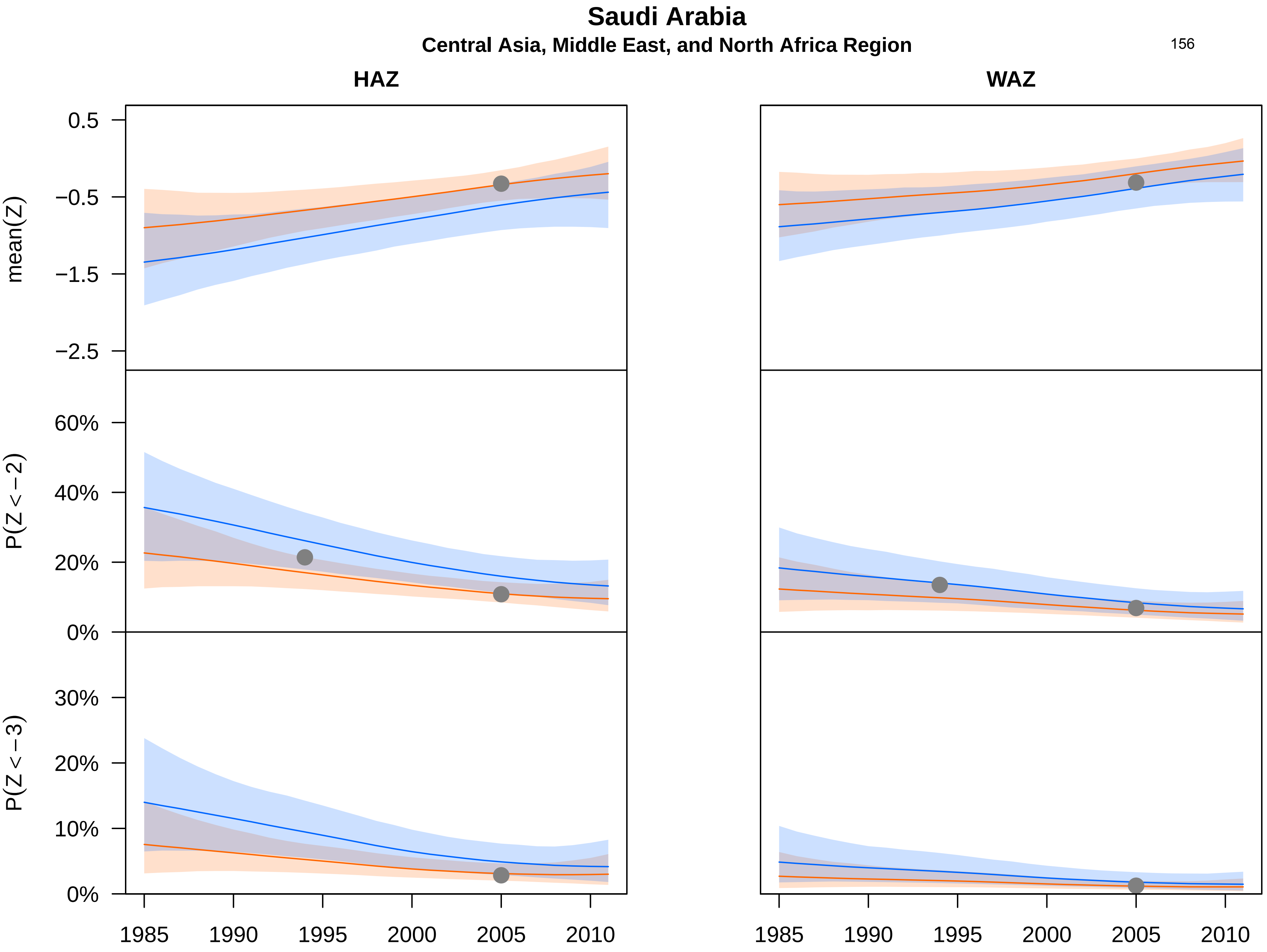
Saudi Arabia
Central Asia, Middle East, and North Africa Region
HAZ
WAZ
mean(Z)
P(Z < −2)
P(Z < −3)
0.5
−0.5
−1.5
−2.5
60%
40%
20%
0%
30%
20%
10%
0%
1985
1990
1995
2000
2005
2010

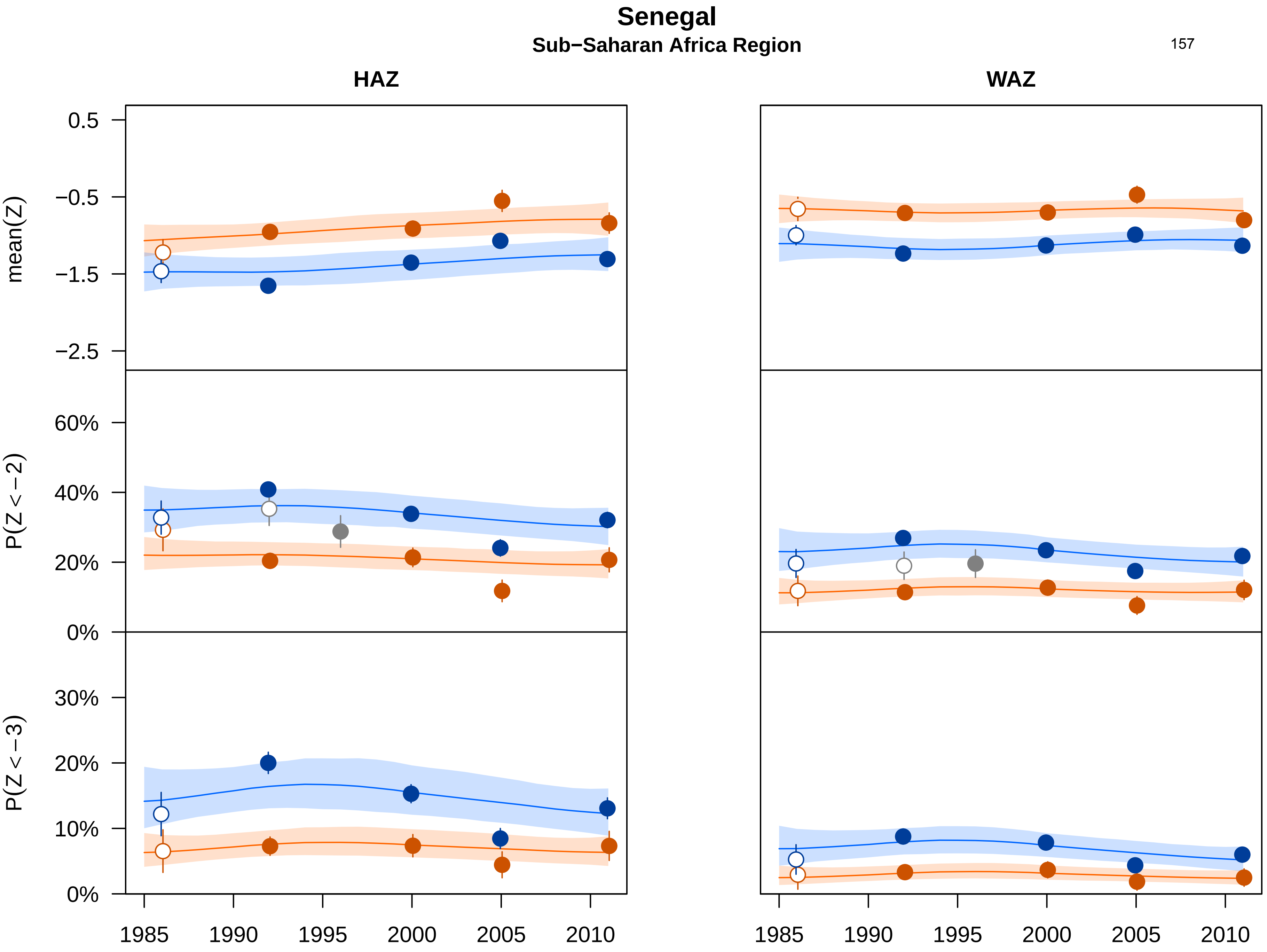
Senegal
Sub−Saharan Africa Region
HAZ
WAZ
mean(Z)
P(Z < −2)
P(Z < −3)
0.5
−0.5
−1.5
−2.5
60%
40%
20%
0%
30%
20%
10%
0%
1985
1990
1995
2000
2005
2010
1985
1990
1995
2000
2005
2010

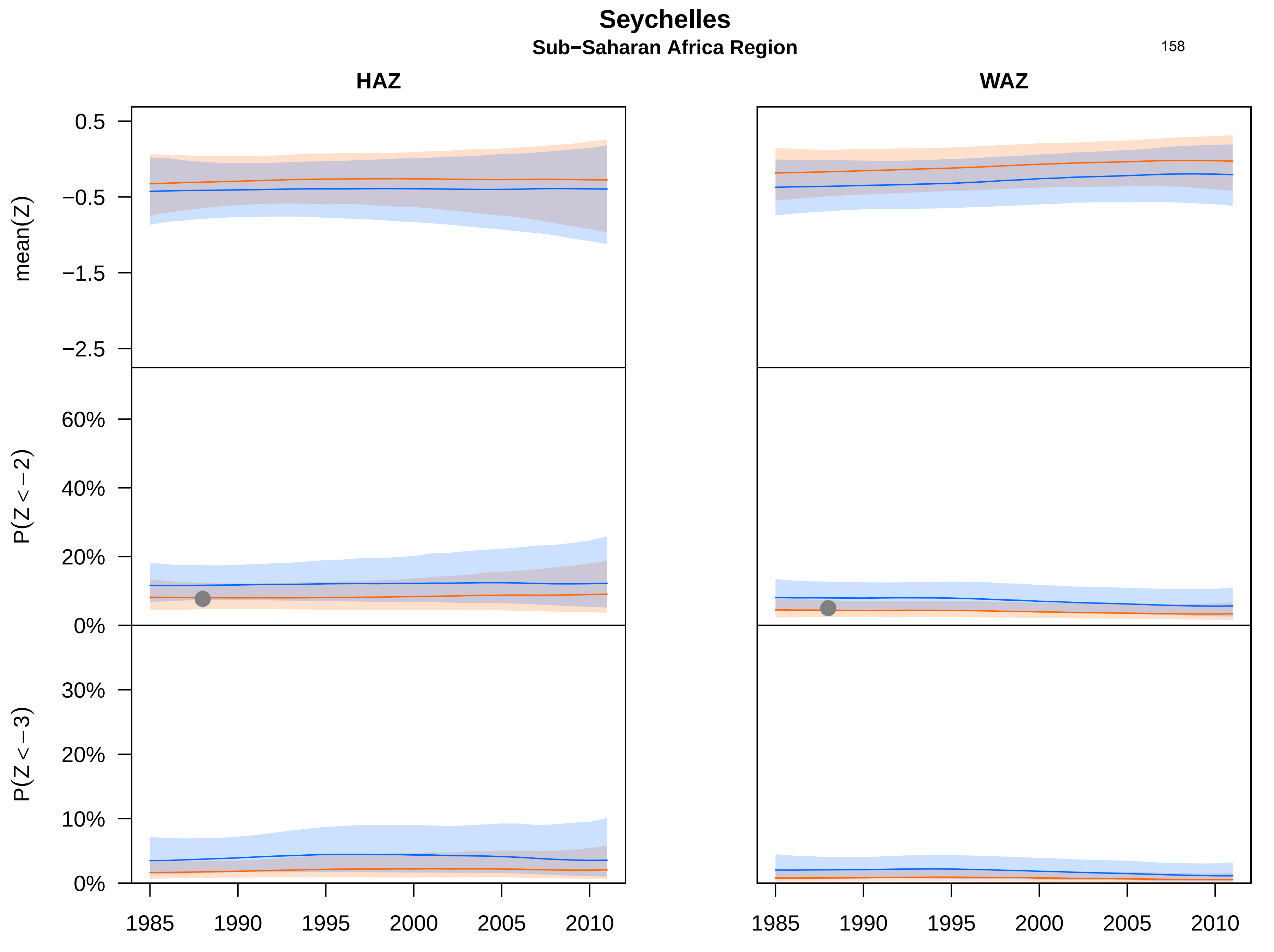
Seychelles
Sub−Saharan Africa Region
HAZ
WAZ
mean(Z)
P(Z < −2)
P(Z < −3)
0.5
−0.5
−1.5
−2.5
60%
40%
20%
0%
30%
20%
10%
0%
1985
1990
1995
2000
2005
2010

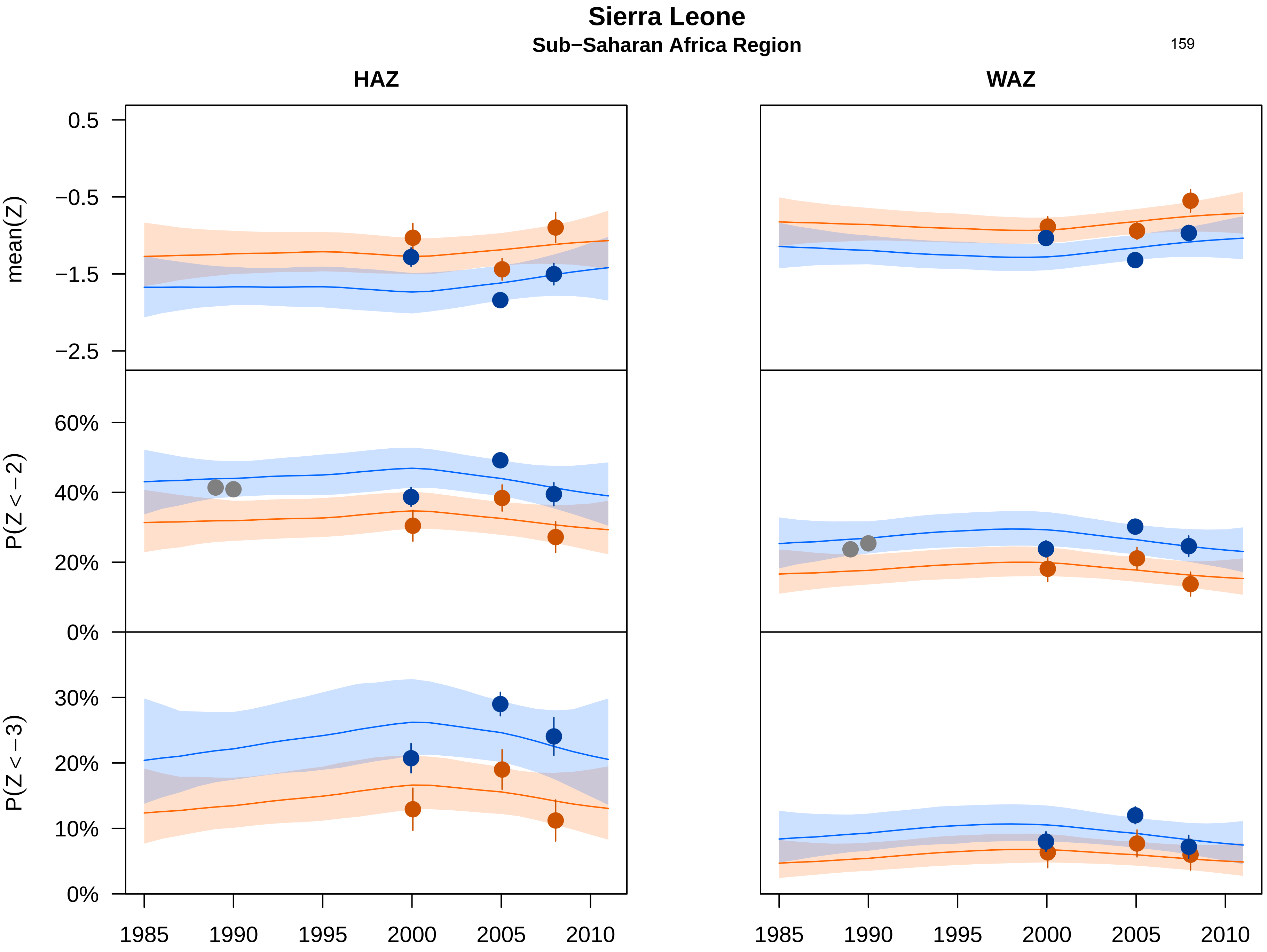
Sierra Leone
Sub−Saharan Africa Region
HAZ
WAZ
mean(Z)
P(Z < −2)
P(Z < −3)
0.5
−0.5
−1.5
−2.5
60%
40%
20%
0%
30%
20%
10%
0%
1985
1990
1995
2000
2005
2010

Solomon Islands

Oceania Region

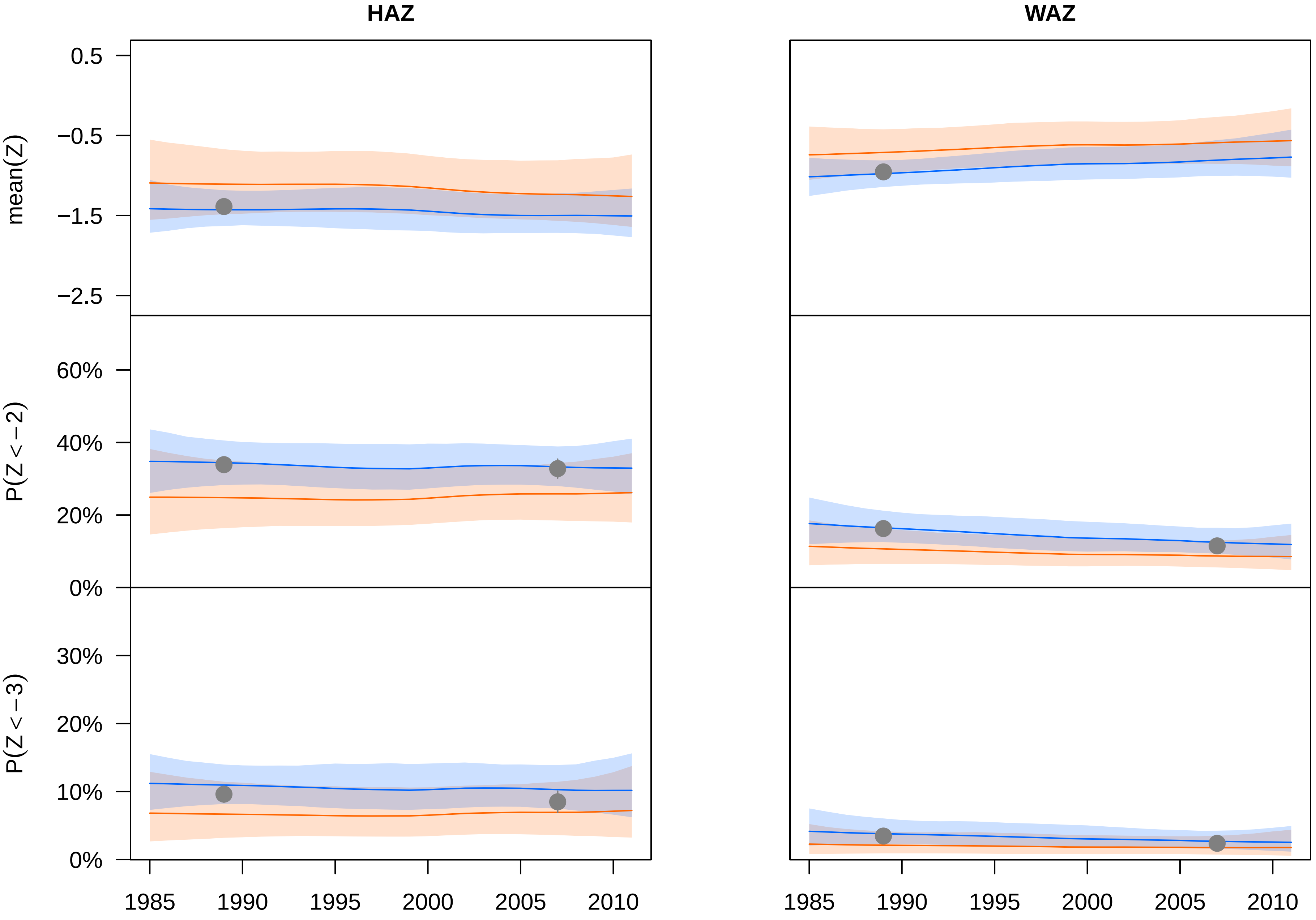

Somalia

Sub−Saharan Africa Region

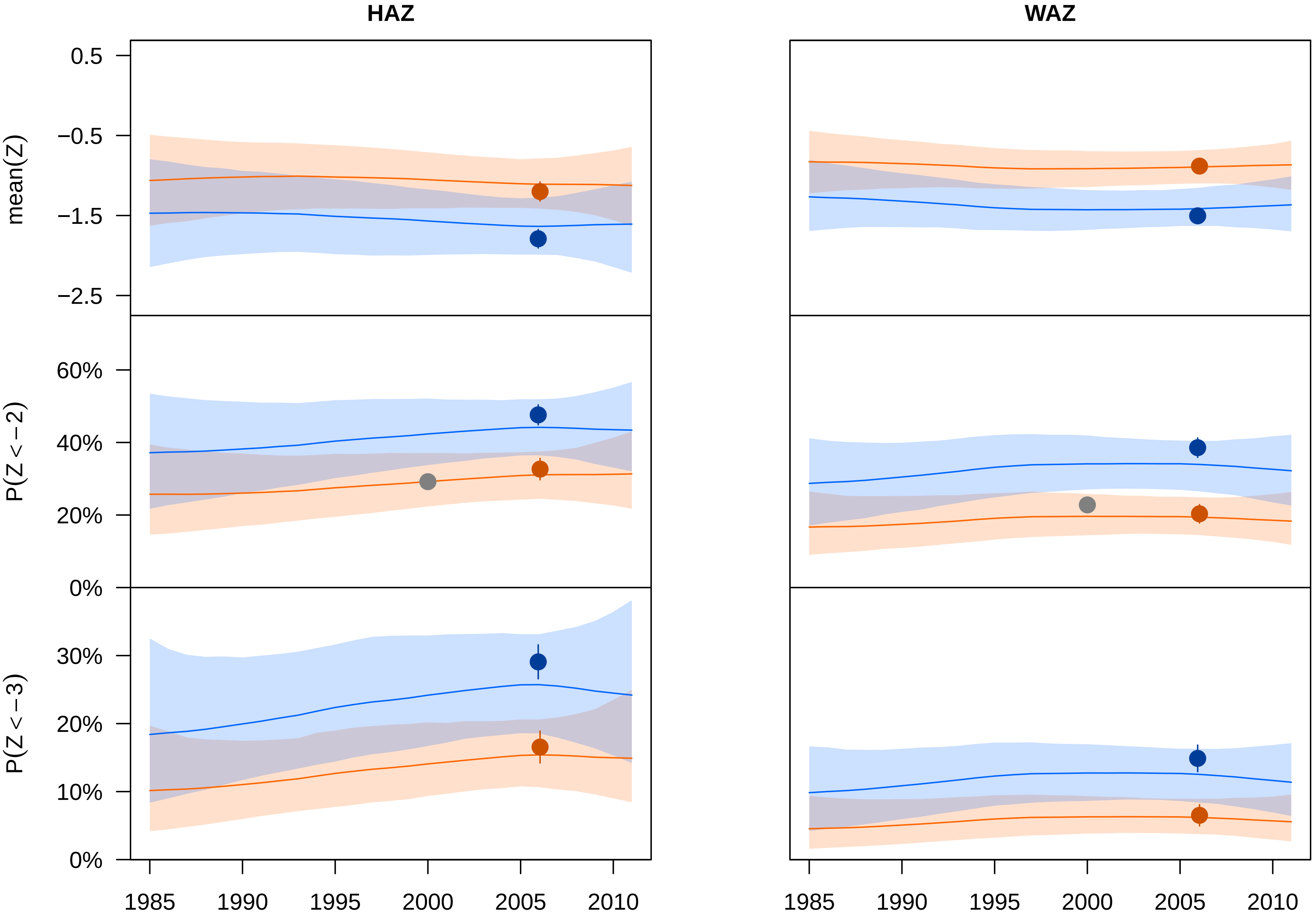

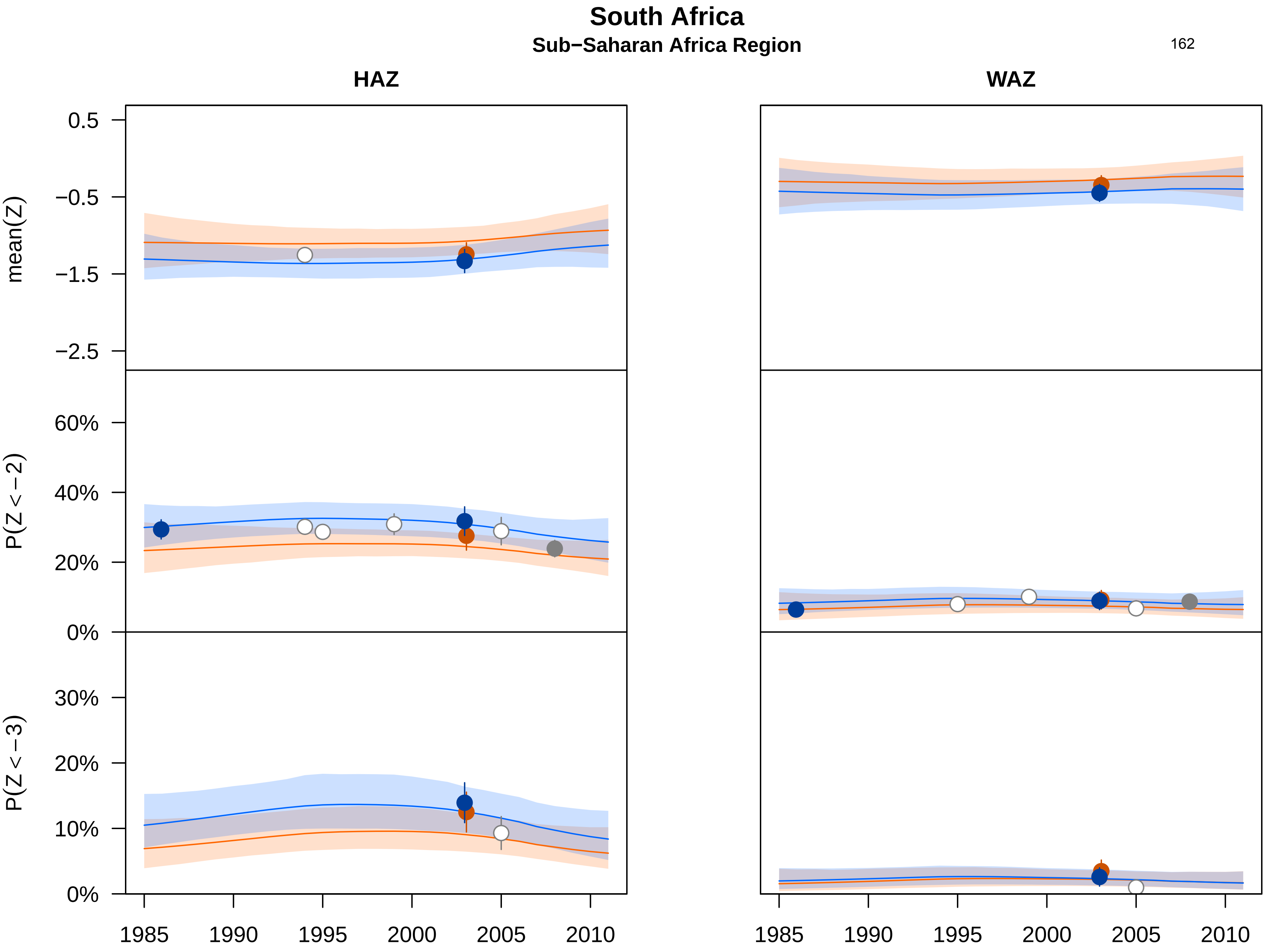
South Africa
Sub−Saharan Africa Region
HAZ
WAZ
mean(Z)
P(Z < −2)
P(Z < −3)
0.5
−0.5
−1.5
−2.5
60%
40%
20%
0%
30%
20%
10%
0%
1985
1990
1995
2000
2005
2010

Sri Lanka

East and Southeast Asia Region

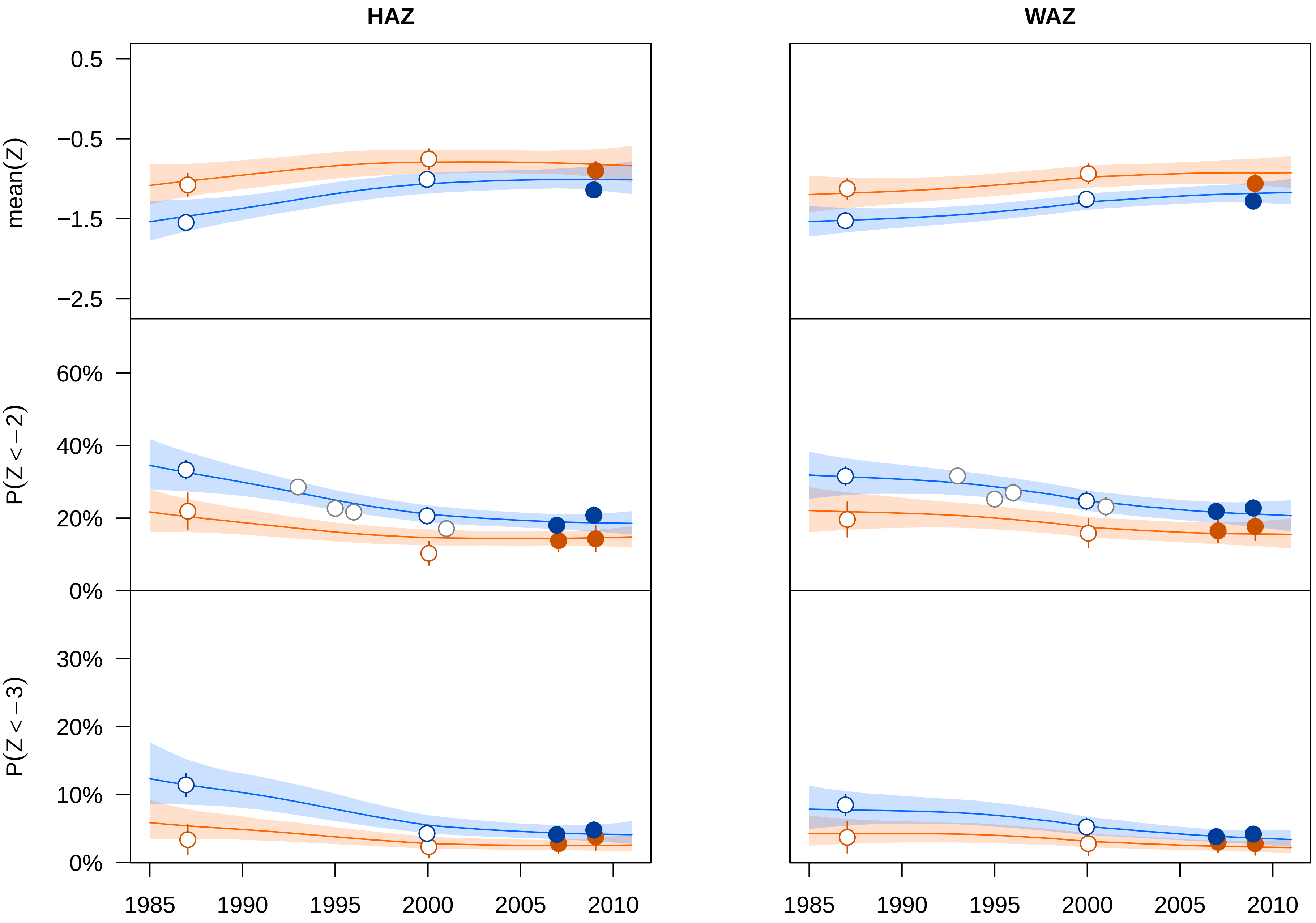

Sudan

Sub−Saharan Africa Region

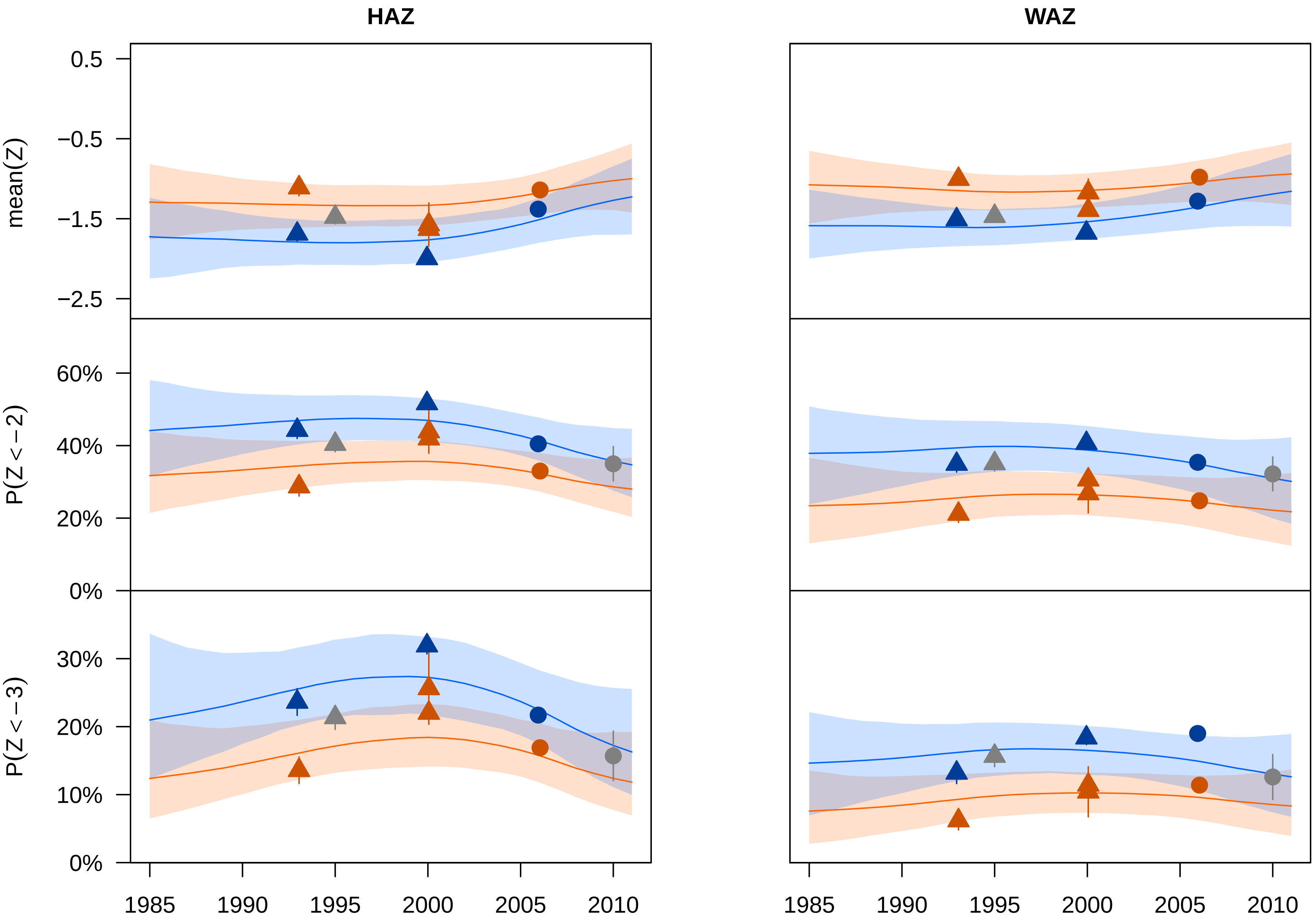

Suriname

Andean and Central Latin America and Caribbean Region

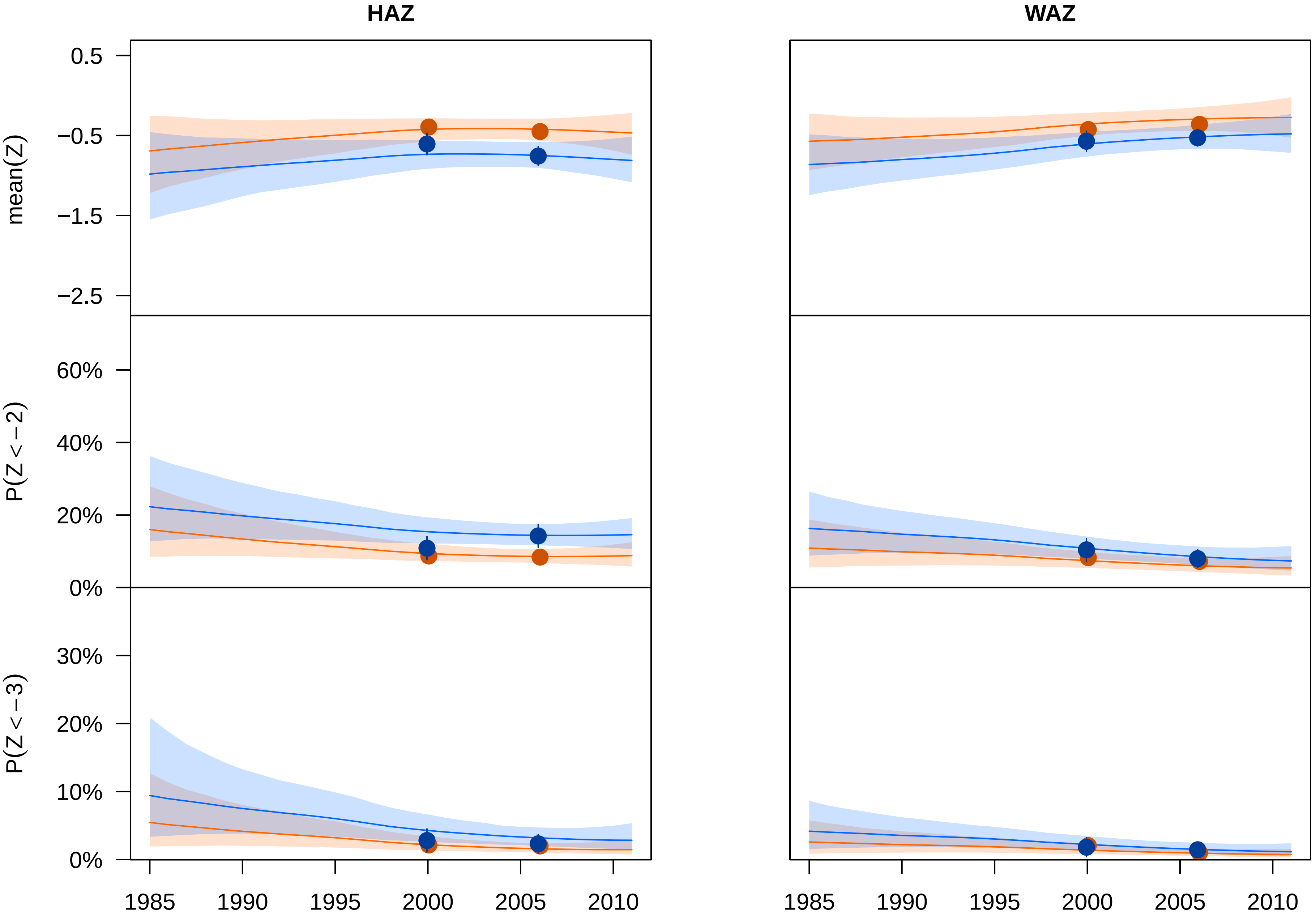

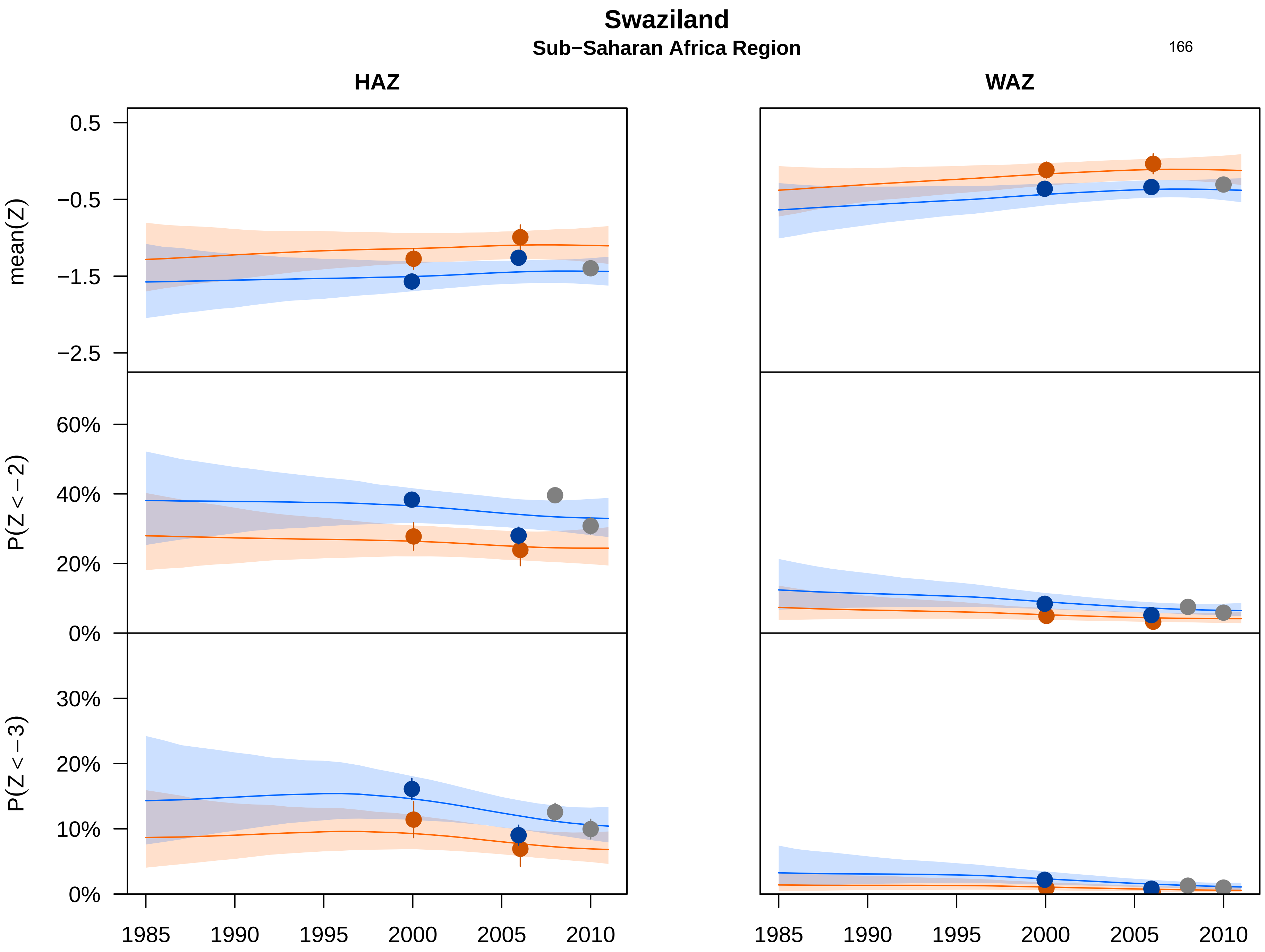
Swaziland
Sub−Saharan Africa Region
HAZ
WAZ
mean(Z)
P(Z < −2)
P(Z < −3)
0.5
−0.5
−1.5
−2.5
60%
40%
20%
0%
30%
20%
10%
0%
1985
1990
1995
2000
2005
2010

Syrian Arab Republic

Central Asia, Middle East, and North Africa Region

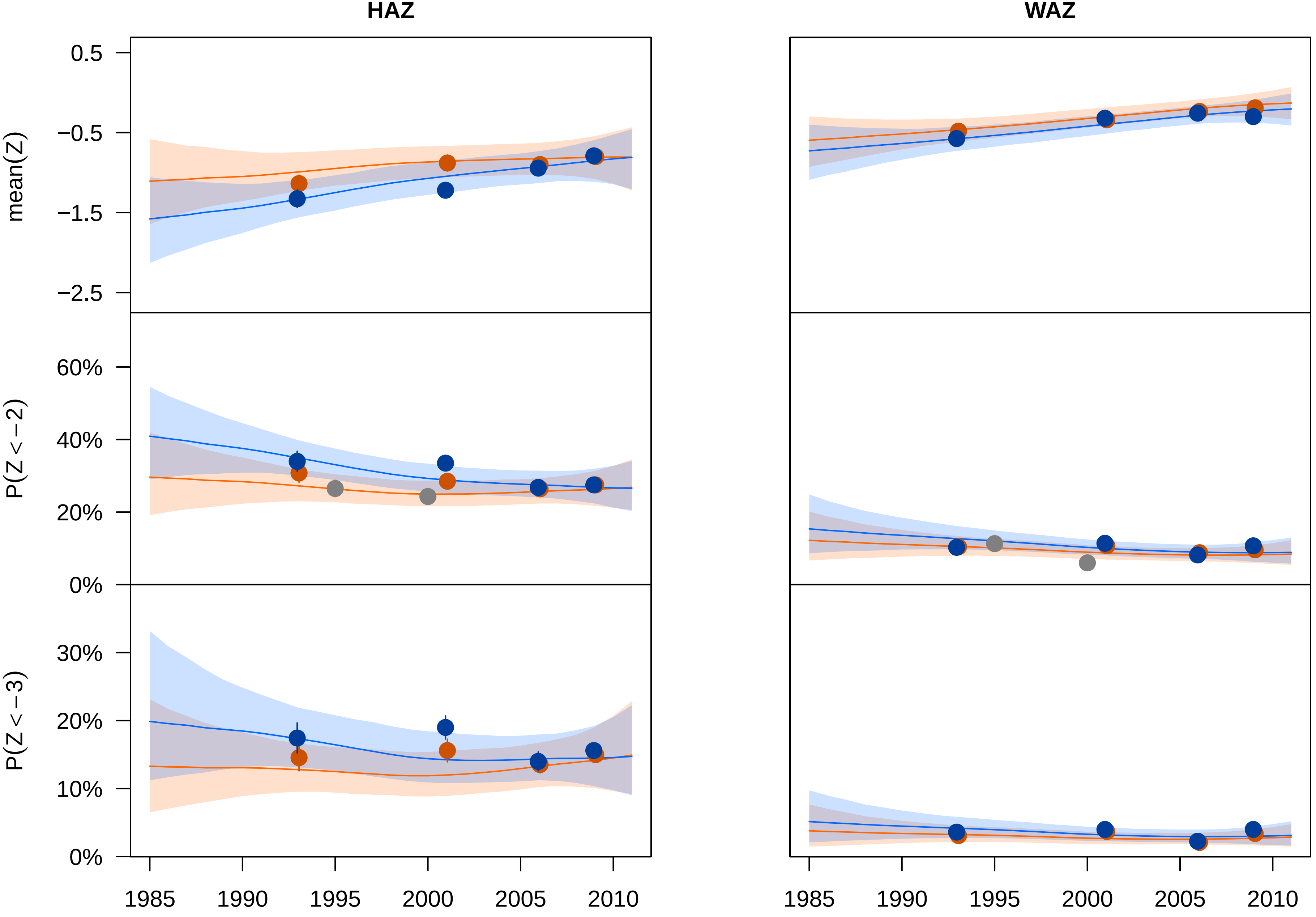

Taiwan

East and Southeast Asia Region

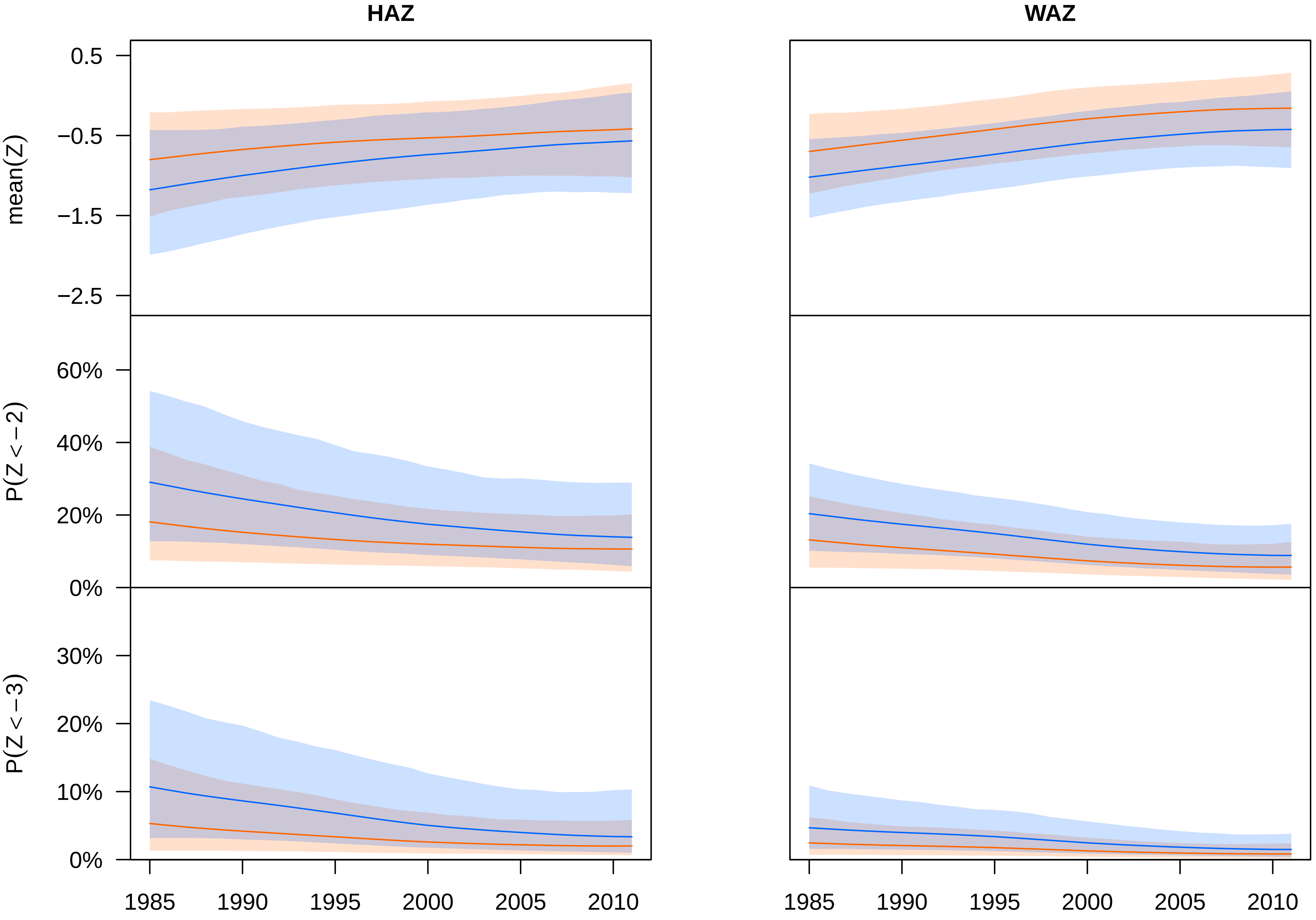

Tajikistan

Central Asia, Middle East, and North Africa Region

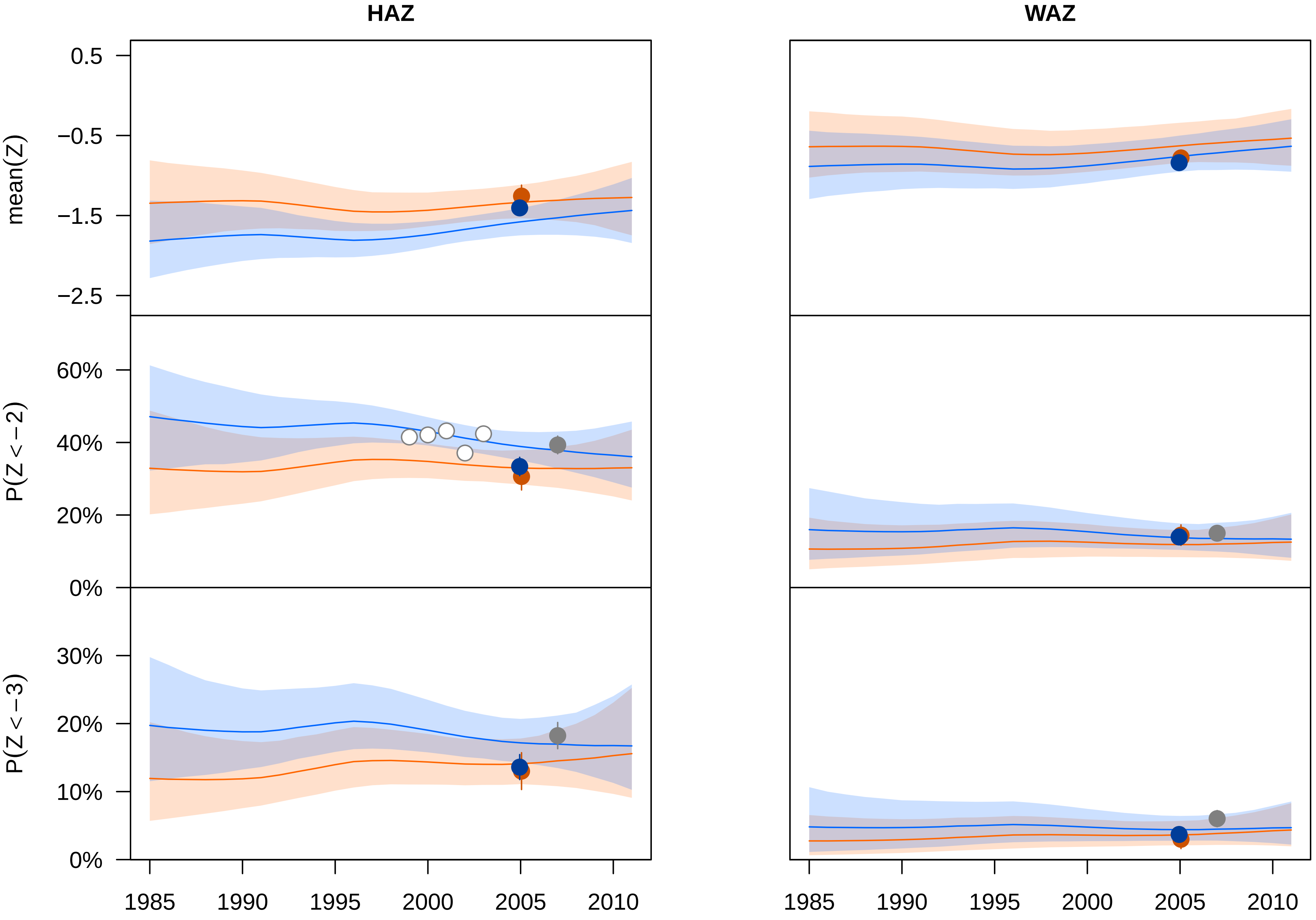

Thailand

East and Southeast Asia Region

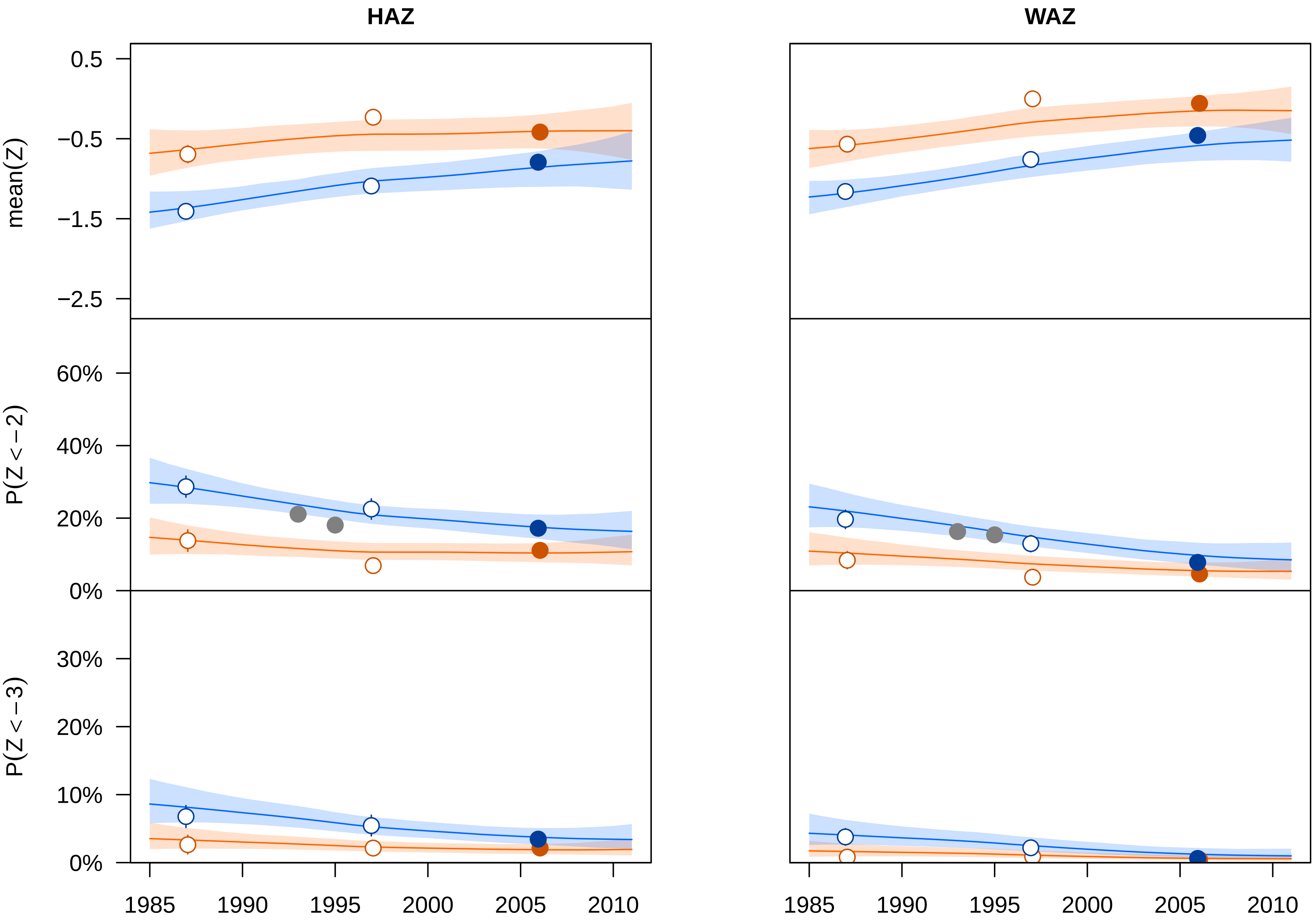

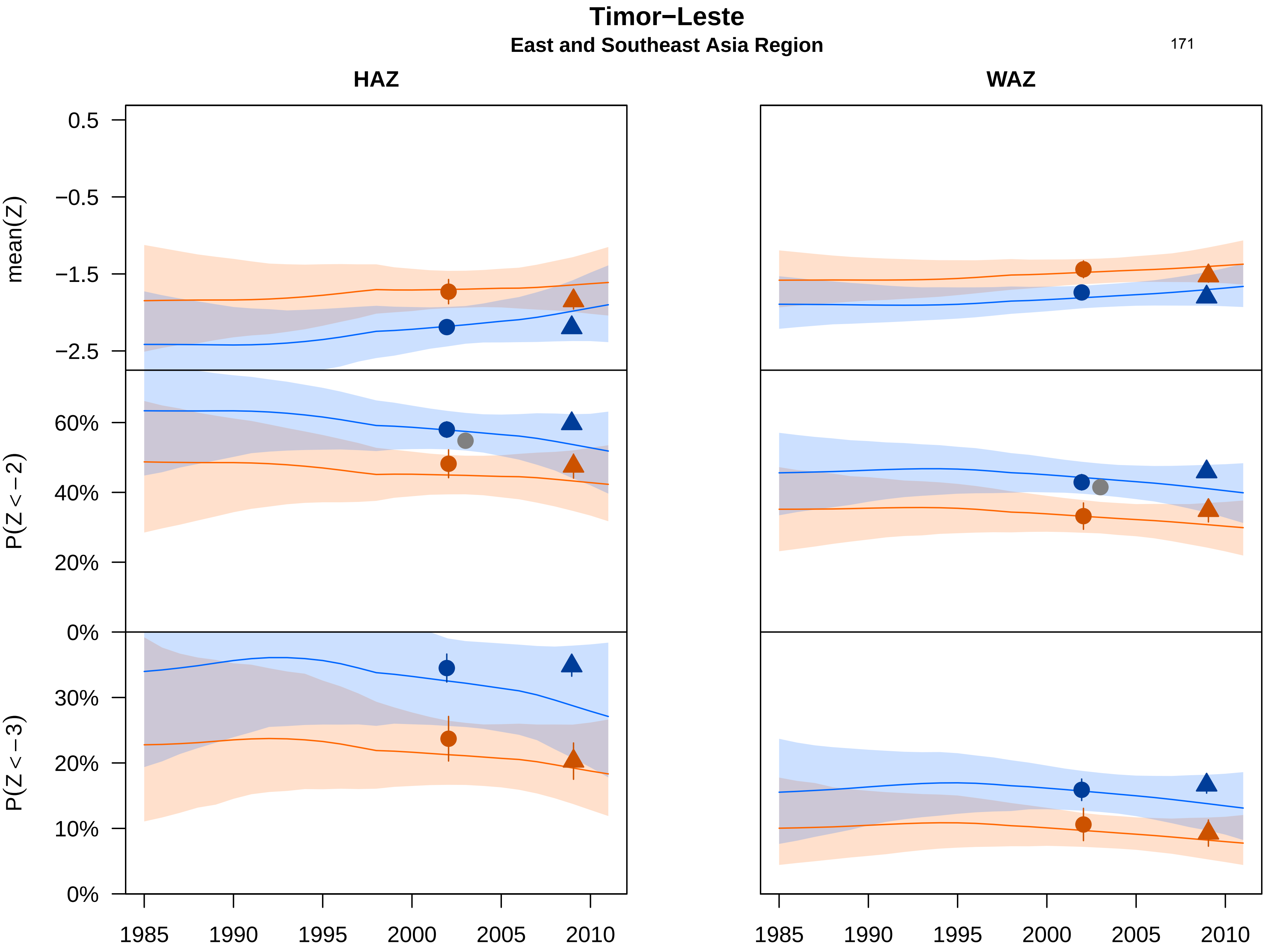
Timor−Leste
East and Southeast Asia Region
HAZ
WAZ
mean(Z)
P(Z < −2)
P(Z < −3)
0.5
−0.5
−1.5
−2.5
60%
40%
20%
0%
30%
20%
10%
0%
1985
1990
1995
2000
2005
2010

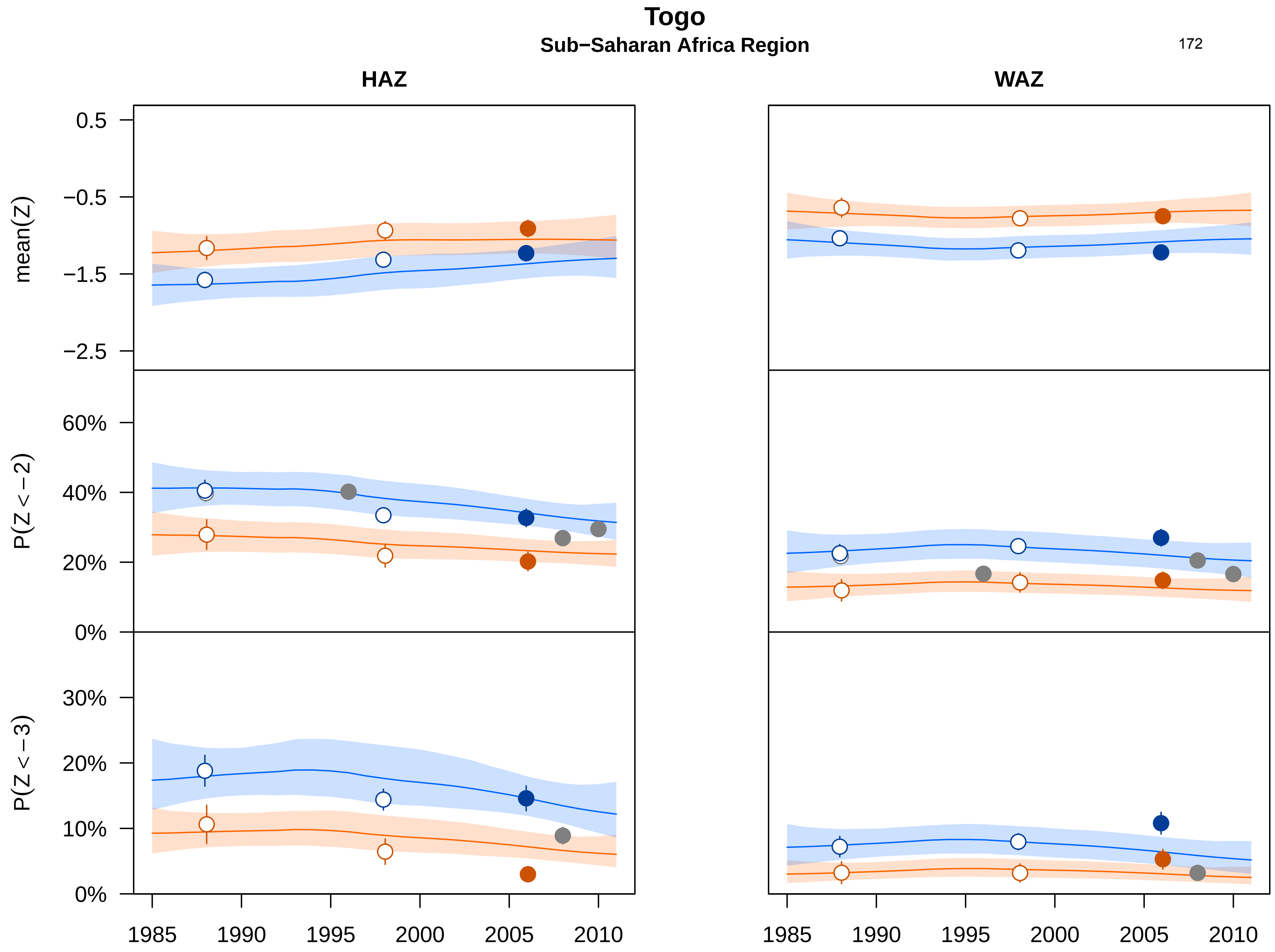
Togo
Sub−Saharan Africa Region
HAZ
WAZ
mean(Z)
P(Z < −2)
P(Z < −3)
0.5
−0.5
−1.5
−2.5
60%
40%
20%
0%
30%
20%
10%
0%
1985
1990
1995
2000
2005
2010

Tonga
Oceania Region

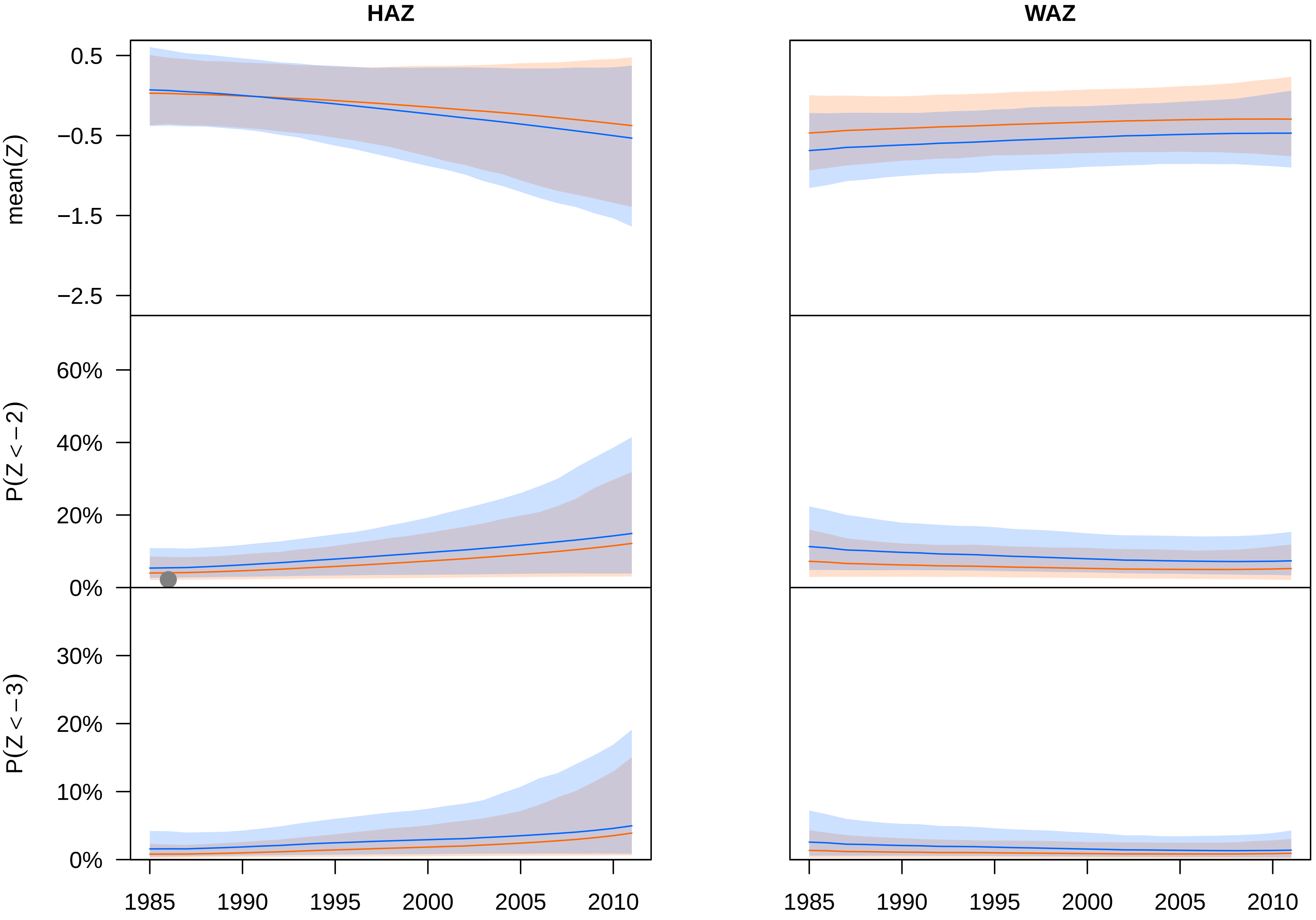

Trinidad and Tobago

Andean and Central Latin America and Caribbean Region

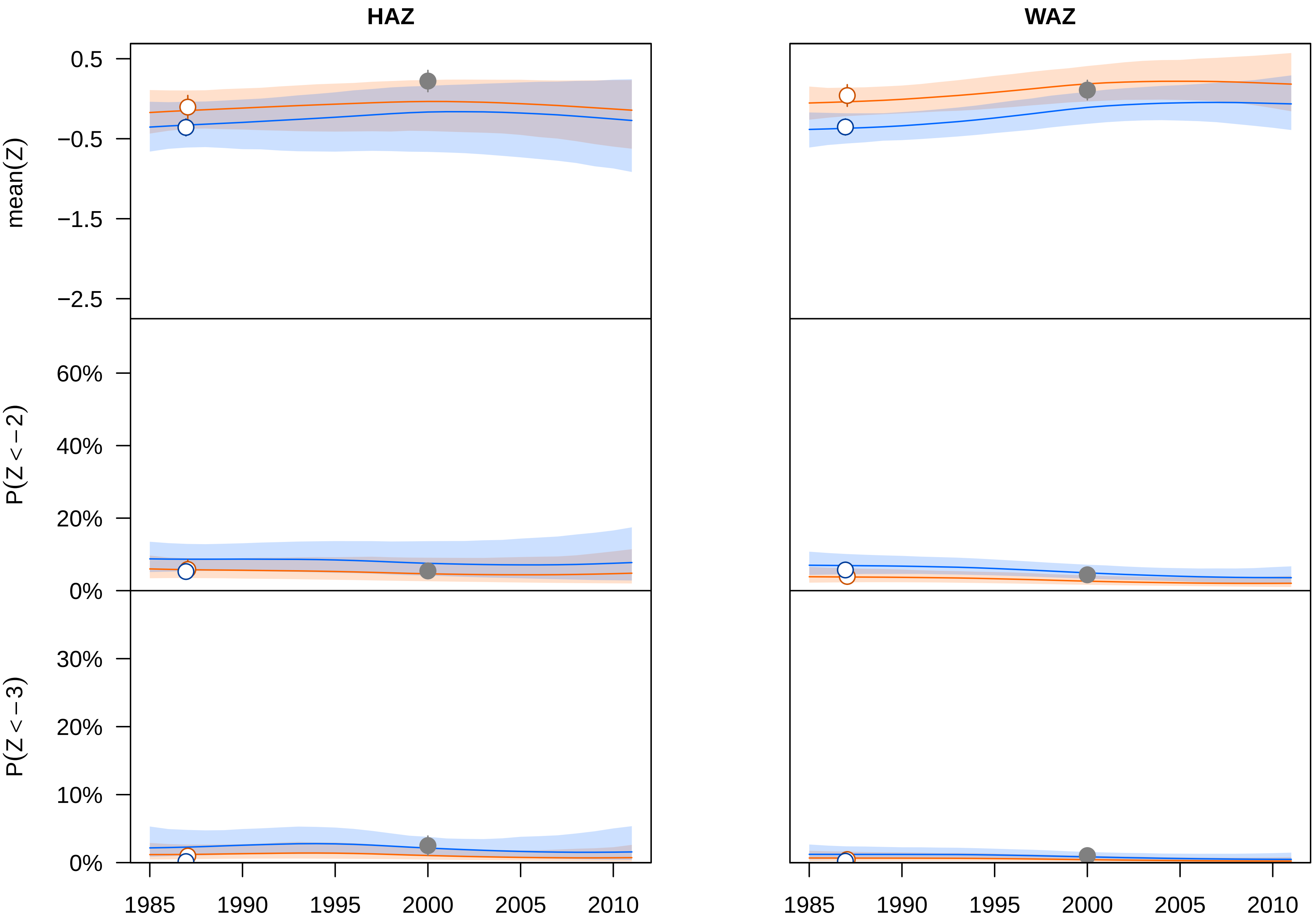

Tunisia

Central Asia, Middle East, and North Africa Region

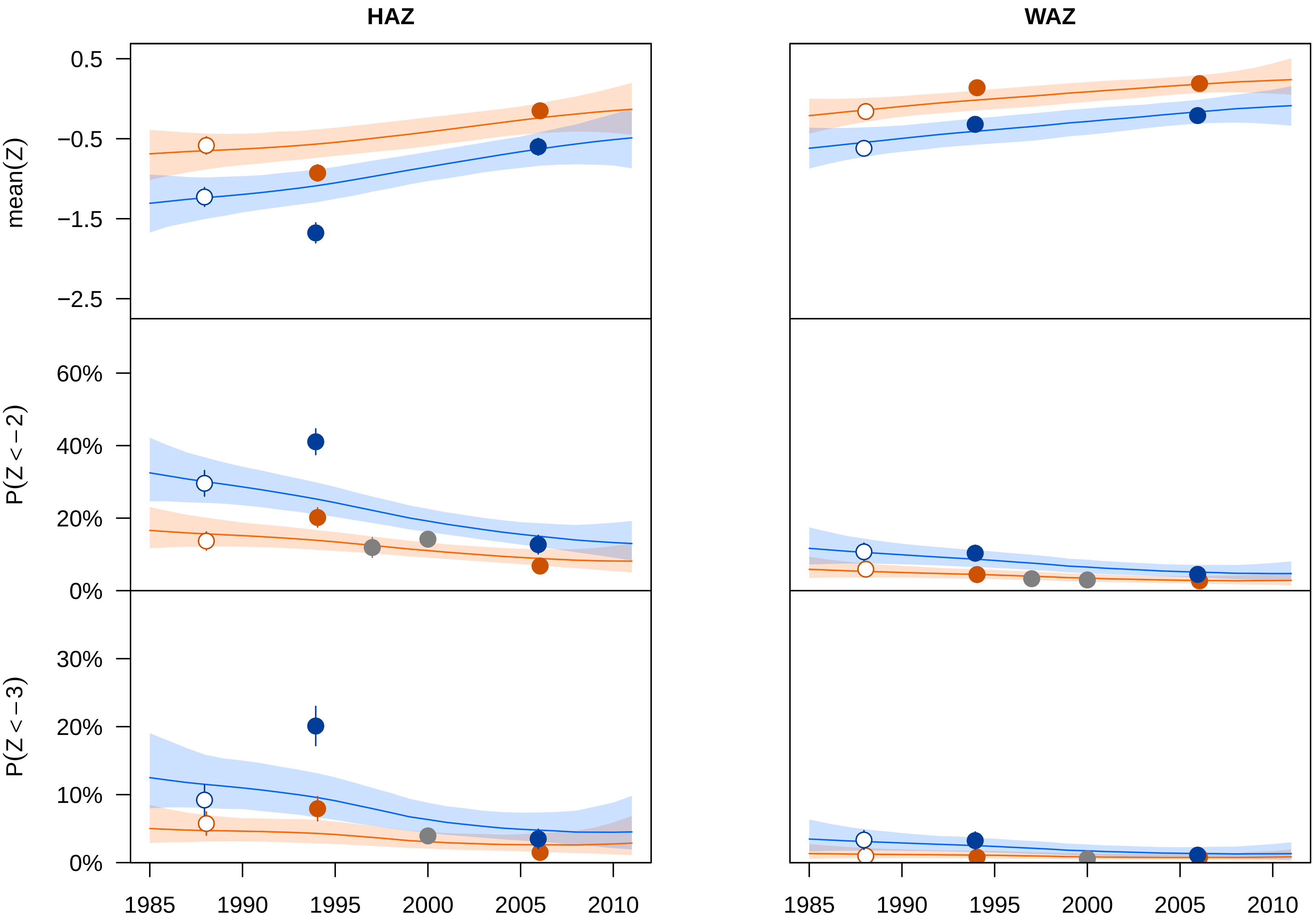

Turkey

Central Asia, Middle East, and North Africa Region

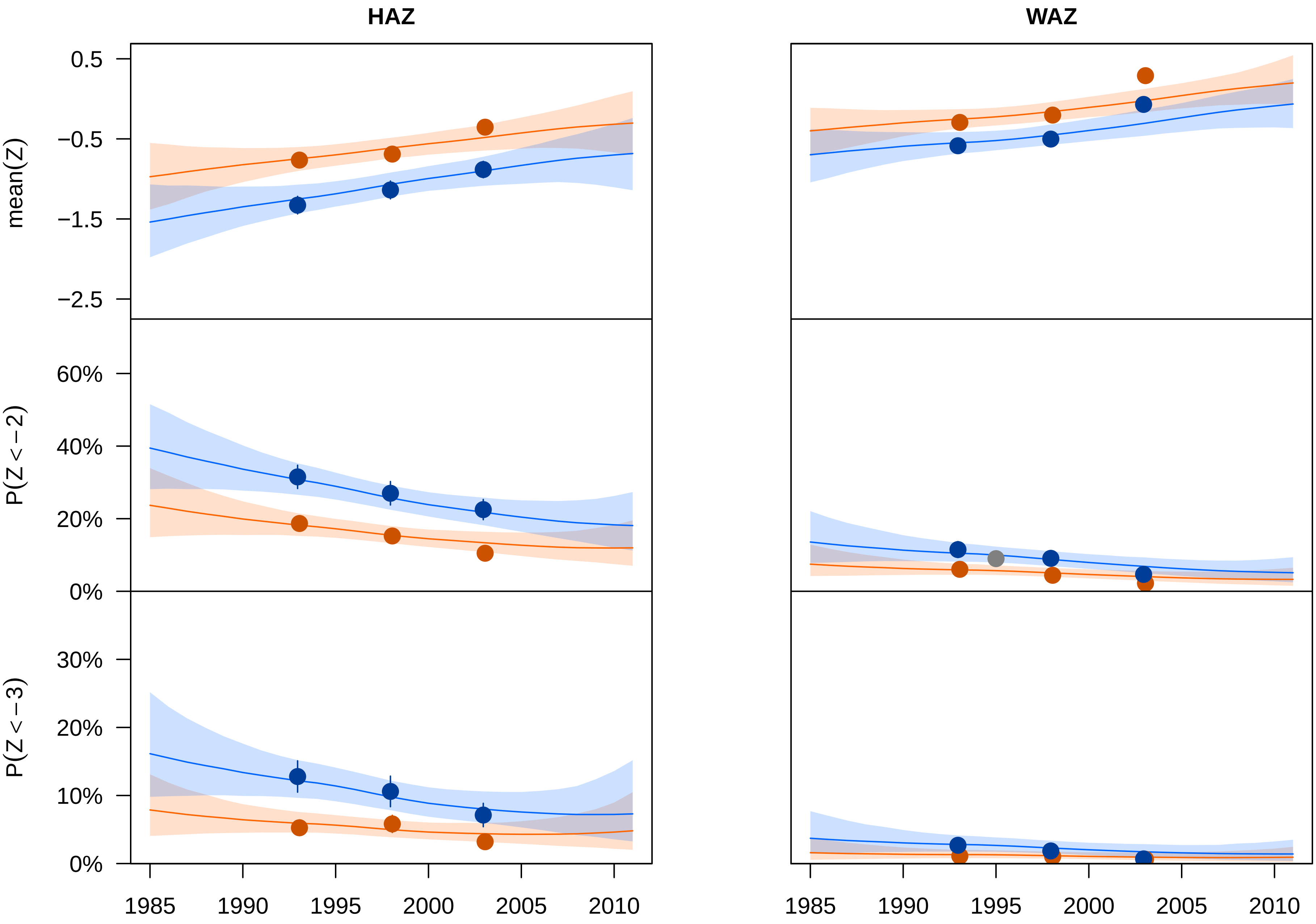

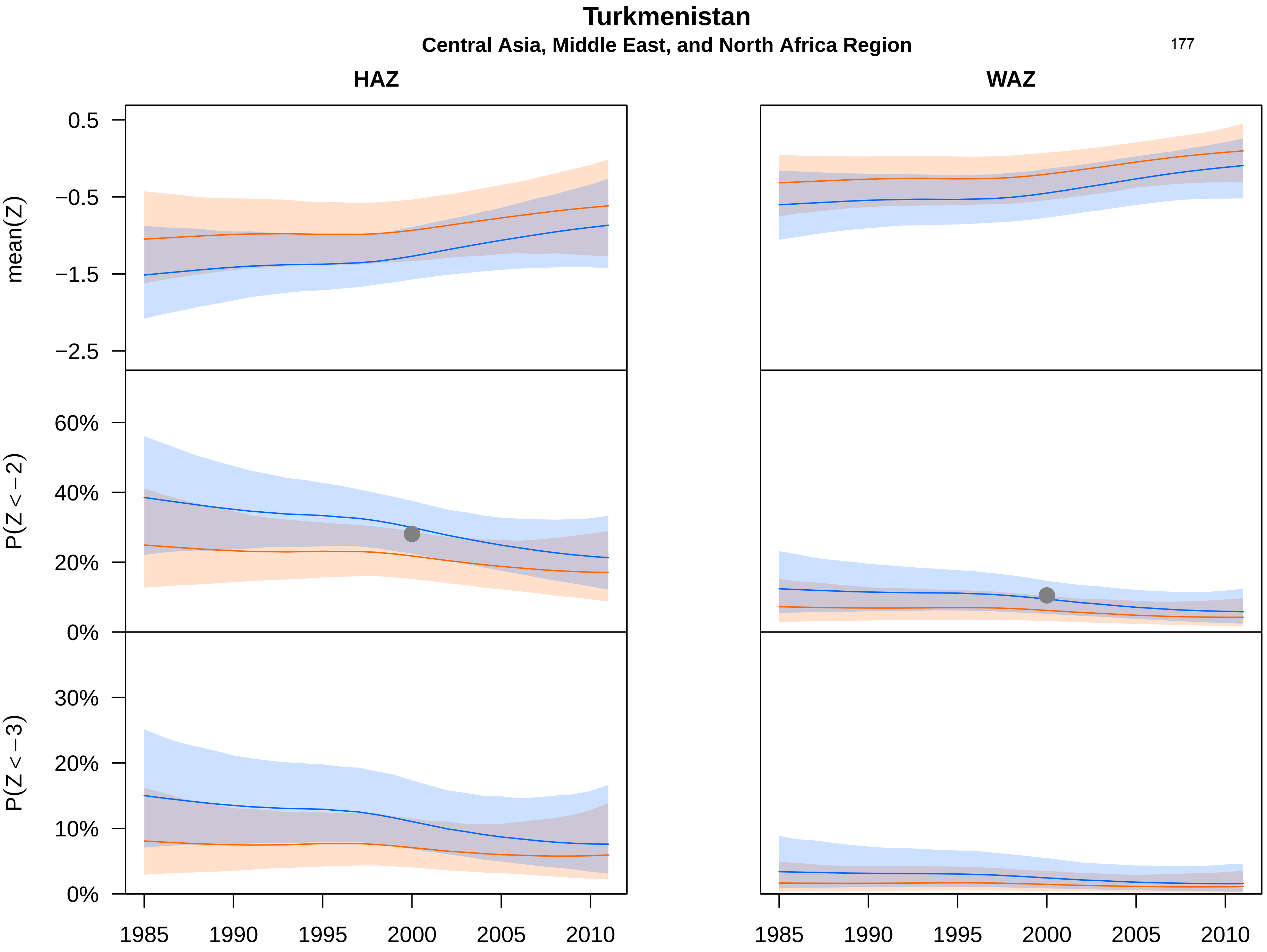
Turkmenistan
Central Asia, Middle East, and North Africa Region
HAZ
WAZ
mean(Z)
P(Z < −2)
P(Z < −3)
0.5
−0.5
−1.5
−2.5
60%
40%
20%
0%
30%
20%
10%
0%
1985
1990
1995
2000
2005
2010
1985
1990
1995
2000
2005
2010

Uganda

Sub−Saharan Africa Region

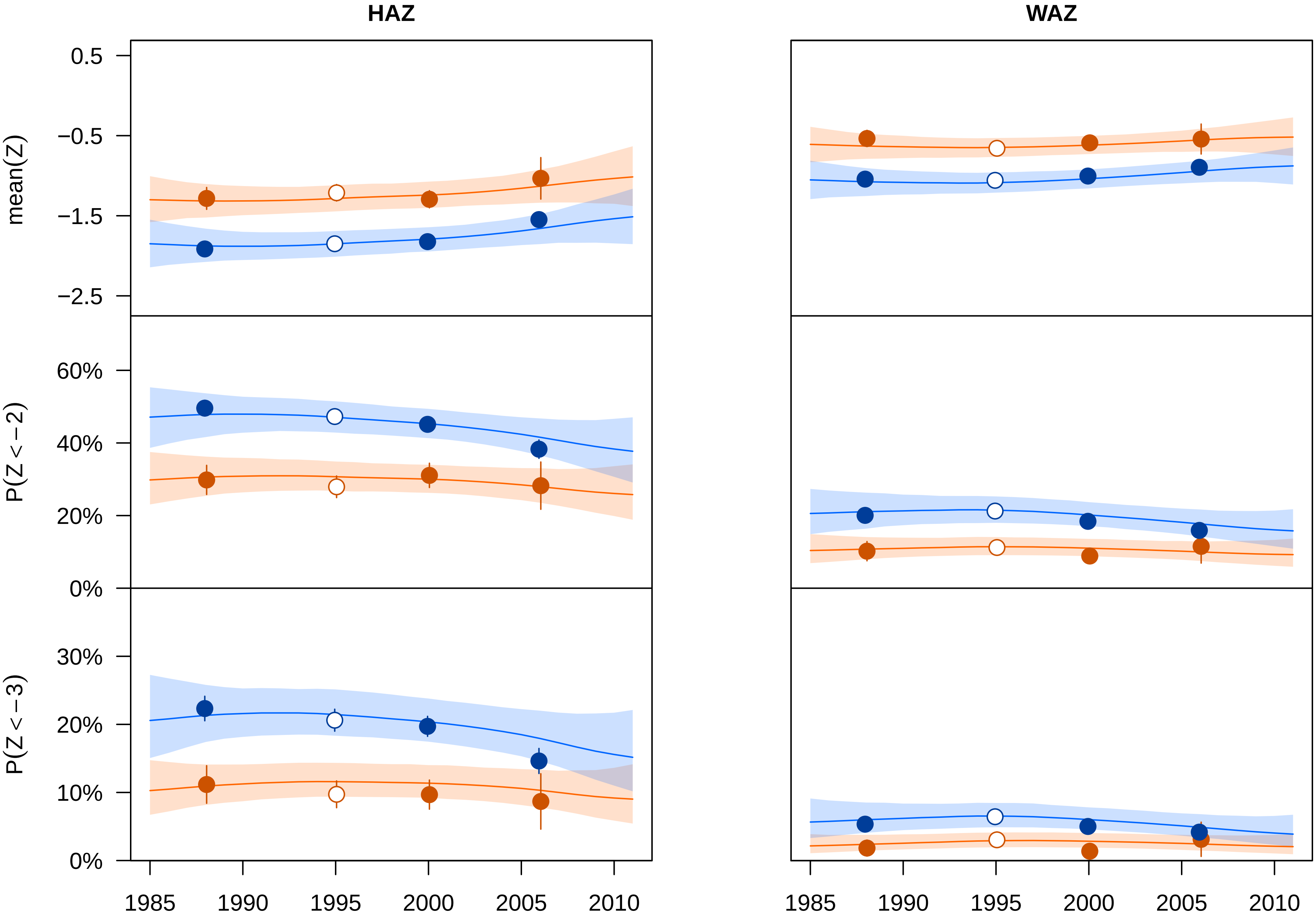

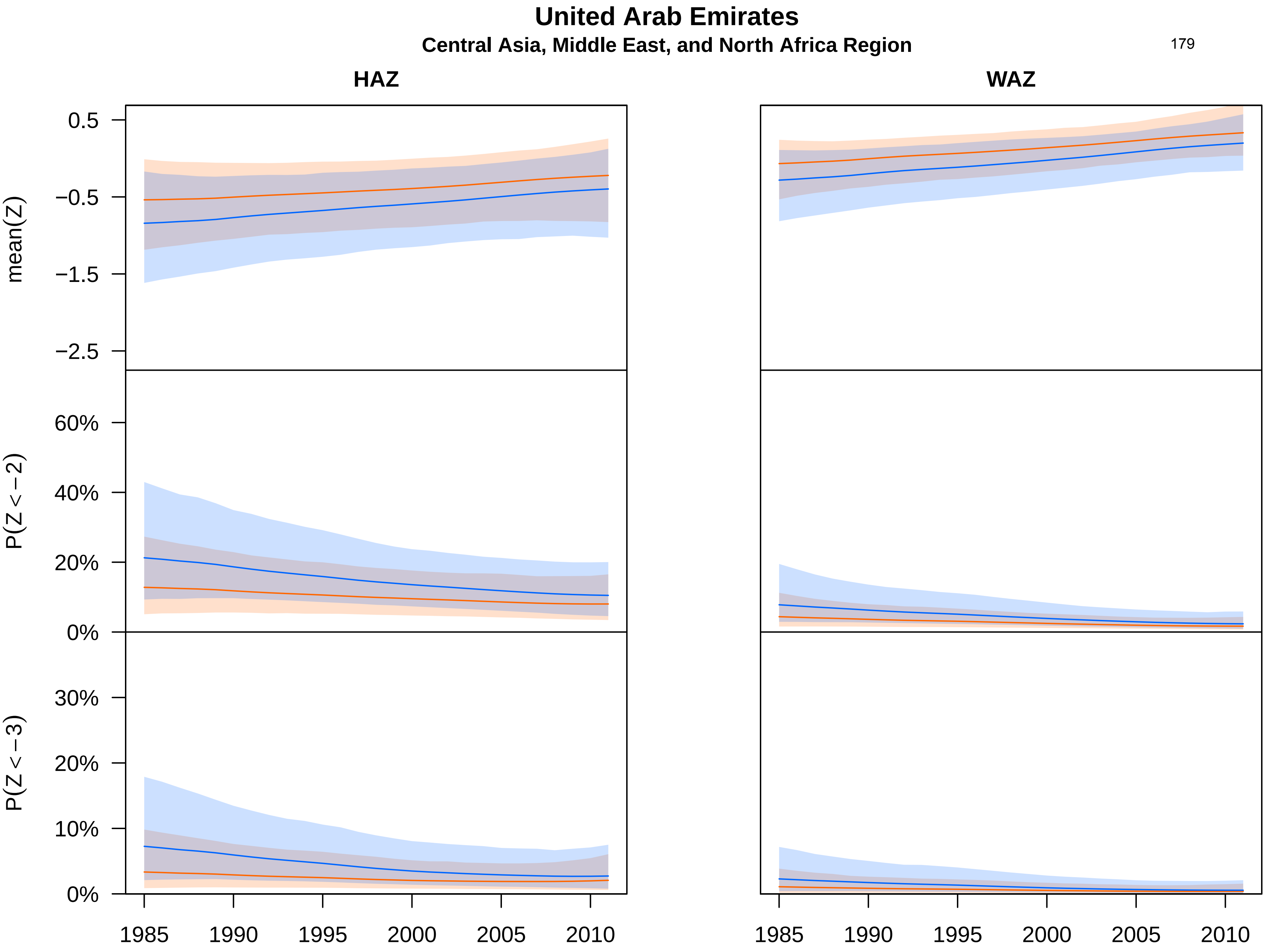
United Arab Emirates
Central Asia, Middle East, and North Africa Region
HAZ
WAZ
mean(Z)
P(Z < −2)
P(Z < −3)
0.5
−0.5
−1.5
−2.5
60%
40%
20%
0%
30%
20%
10%
0%
1985
1990
1995
2000
2005
2010

United Republic of Tanzania

Sub−Saharan Africa Region

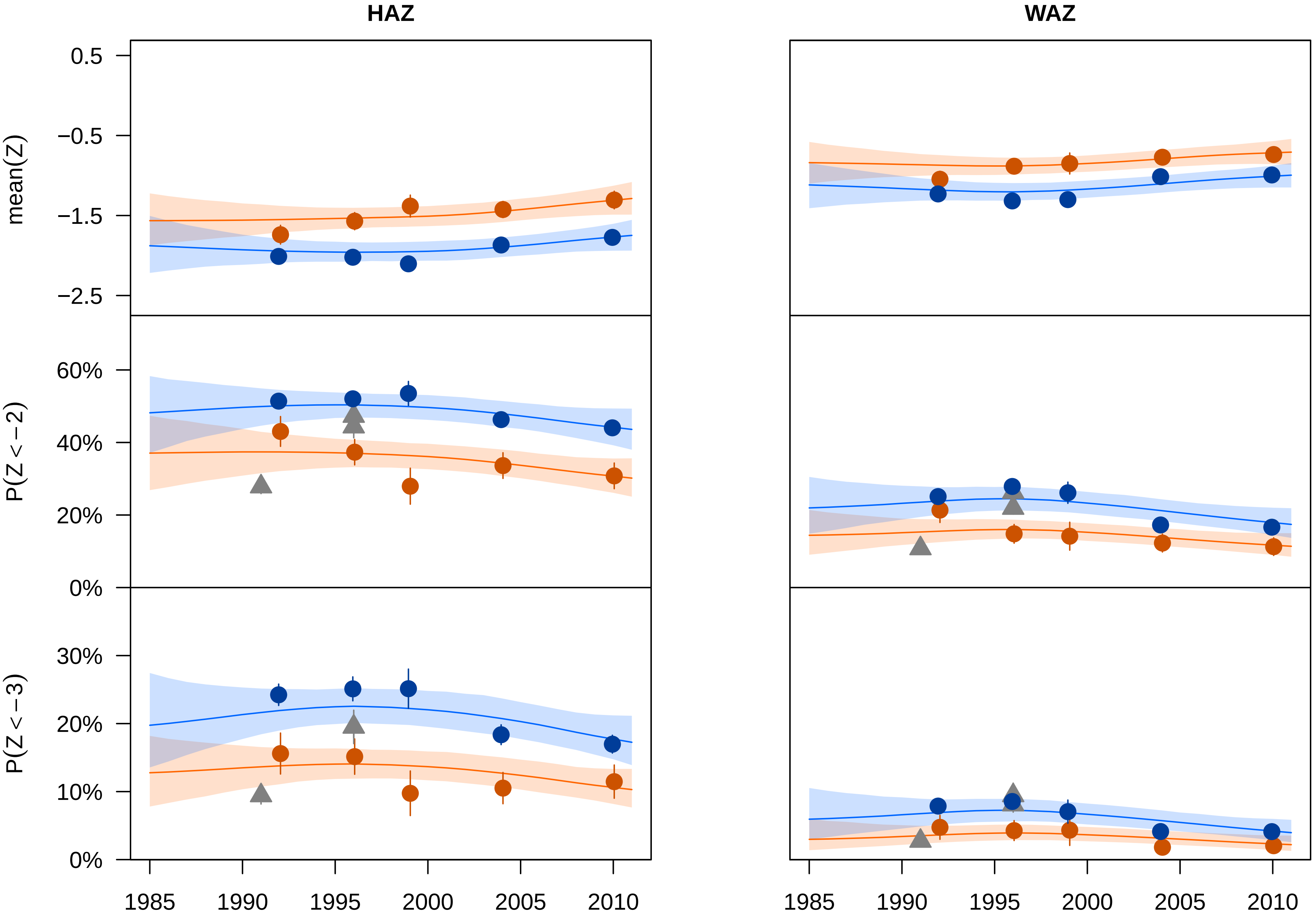

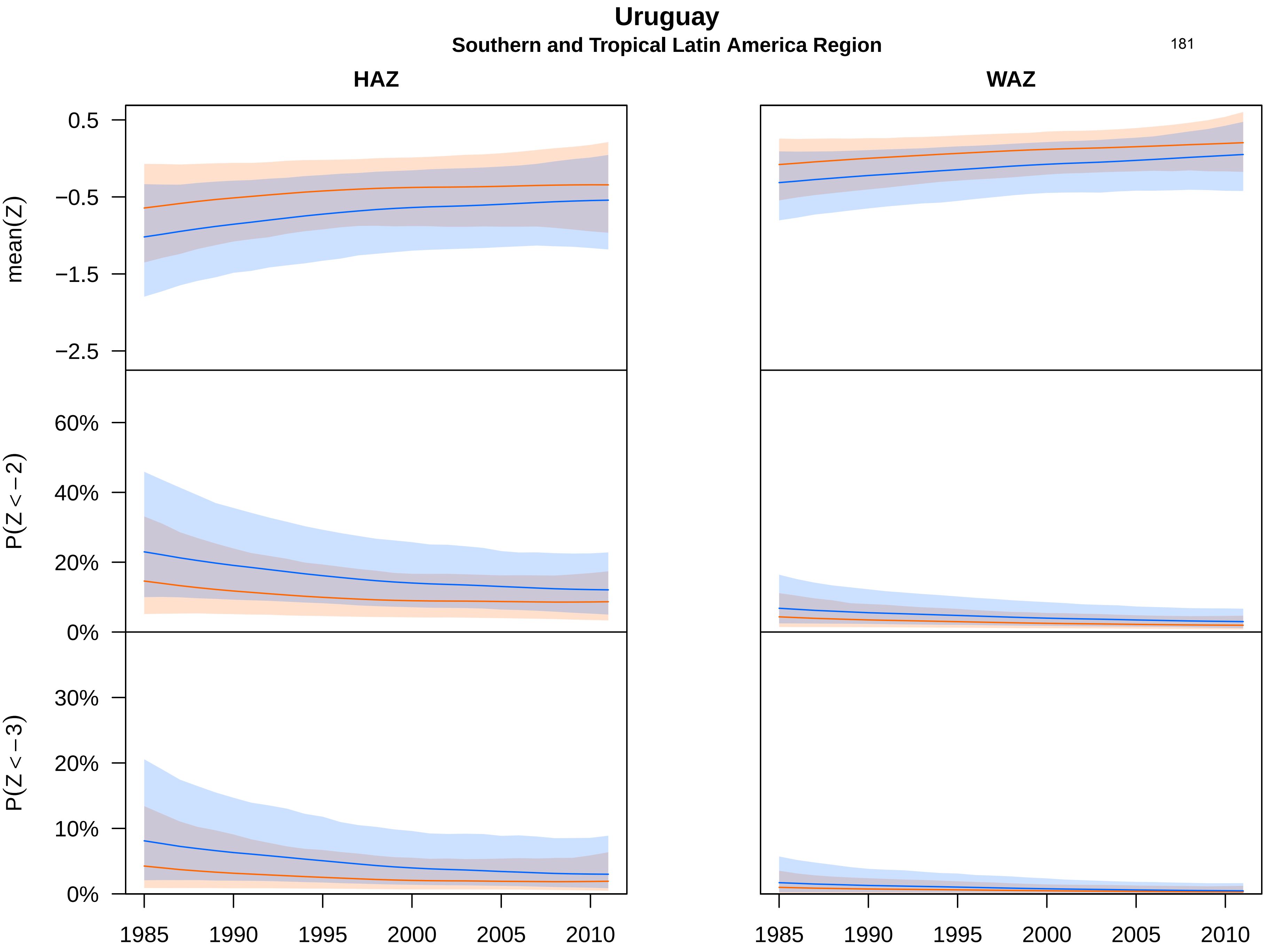
Uruguay
Southern and Tropical Latin America Region
HAZ
WAZ
mean(Z)
P(Z < −2)
P(Z < −3)
0.5
−0.5
−1.5
−2.5
60%
40%
20%
0%
30%
20%
10%
0%
1985
1990
1995
2000
2005
2010

Uzbekistan

Central Asia, Middle East, and North Africa Region

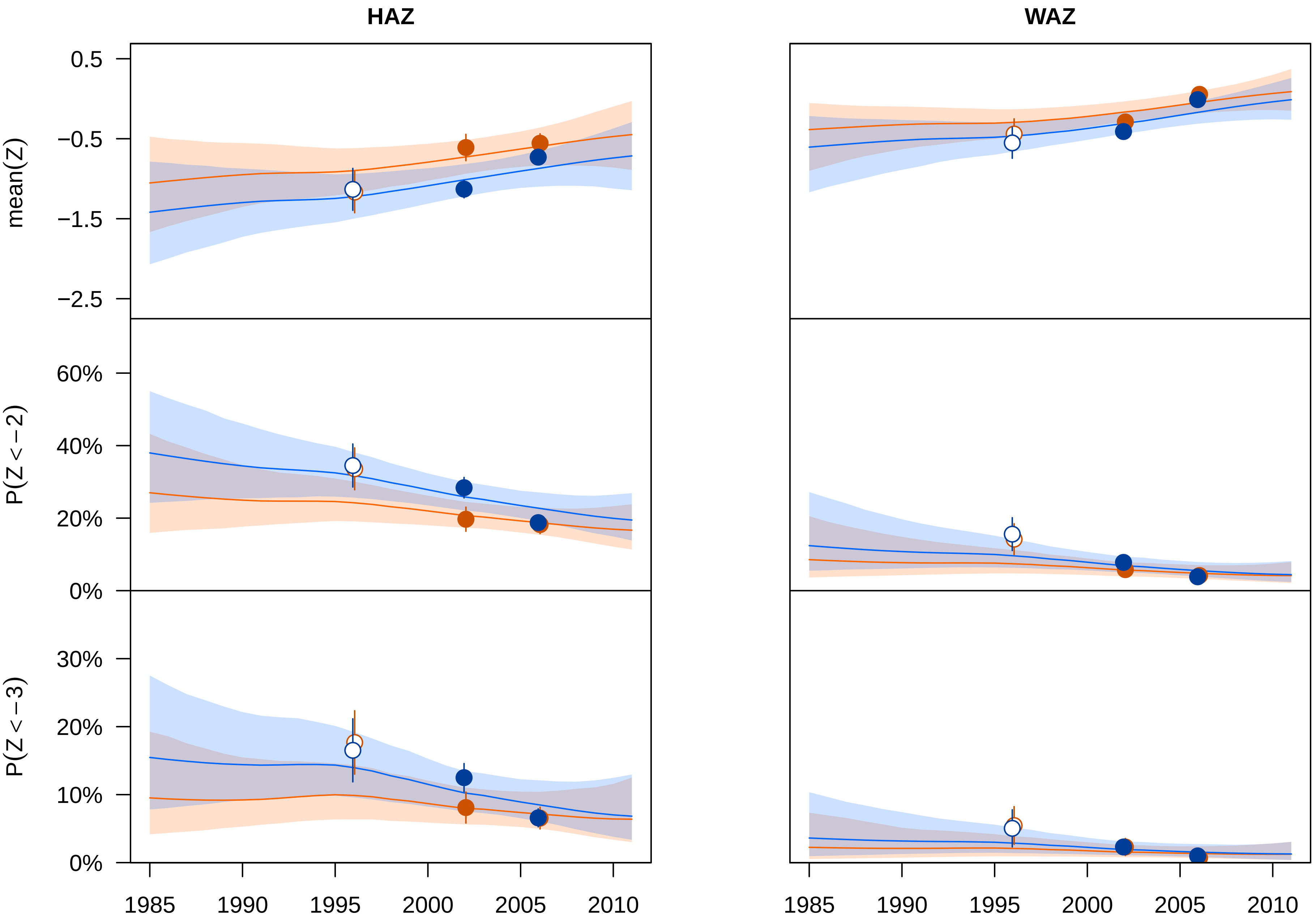

Vanuatu

Oceania Region

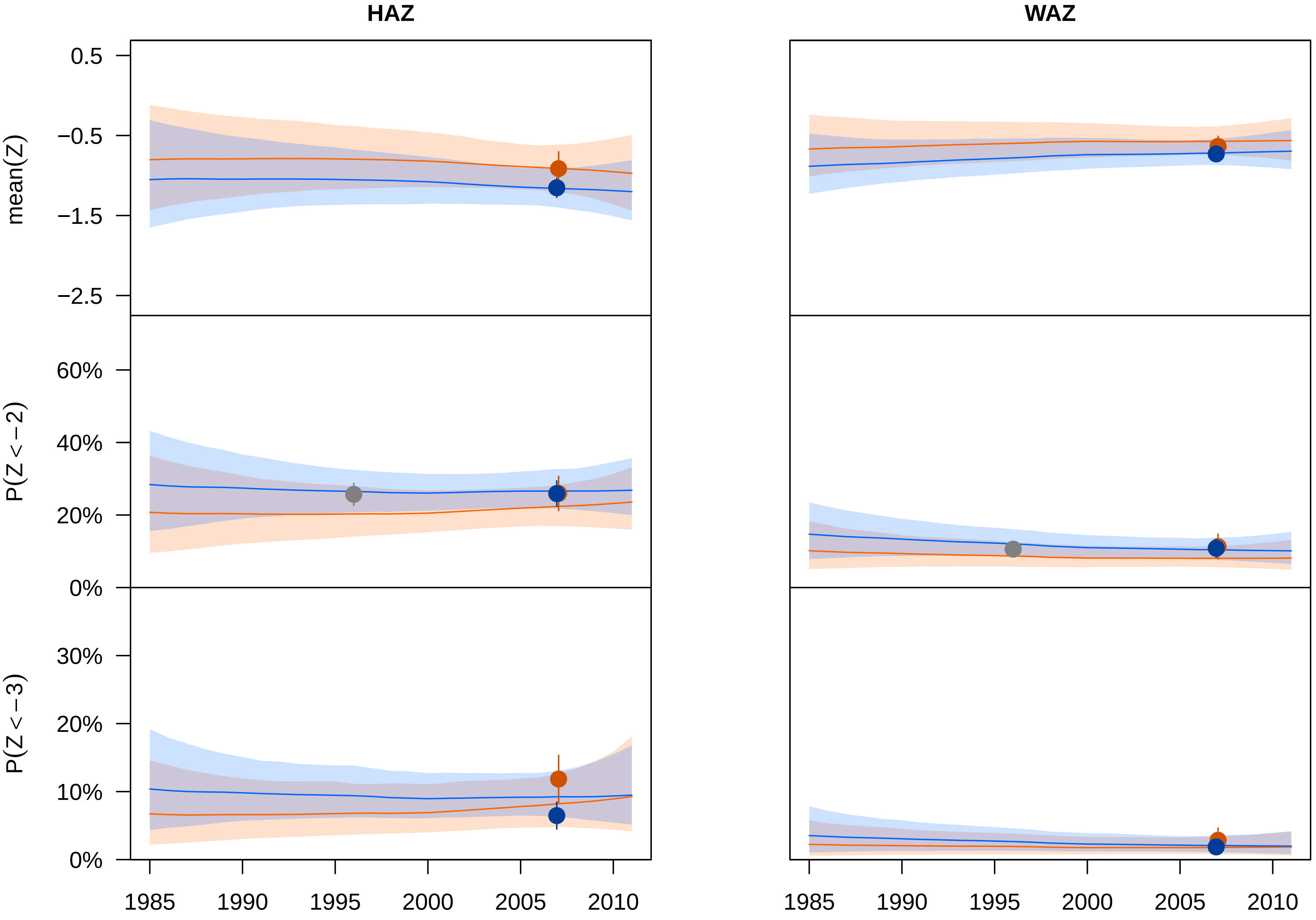

Venezuela (Bolivarian Republic of)

Andean and Central Latin America and Caribbean Region

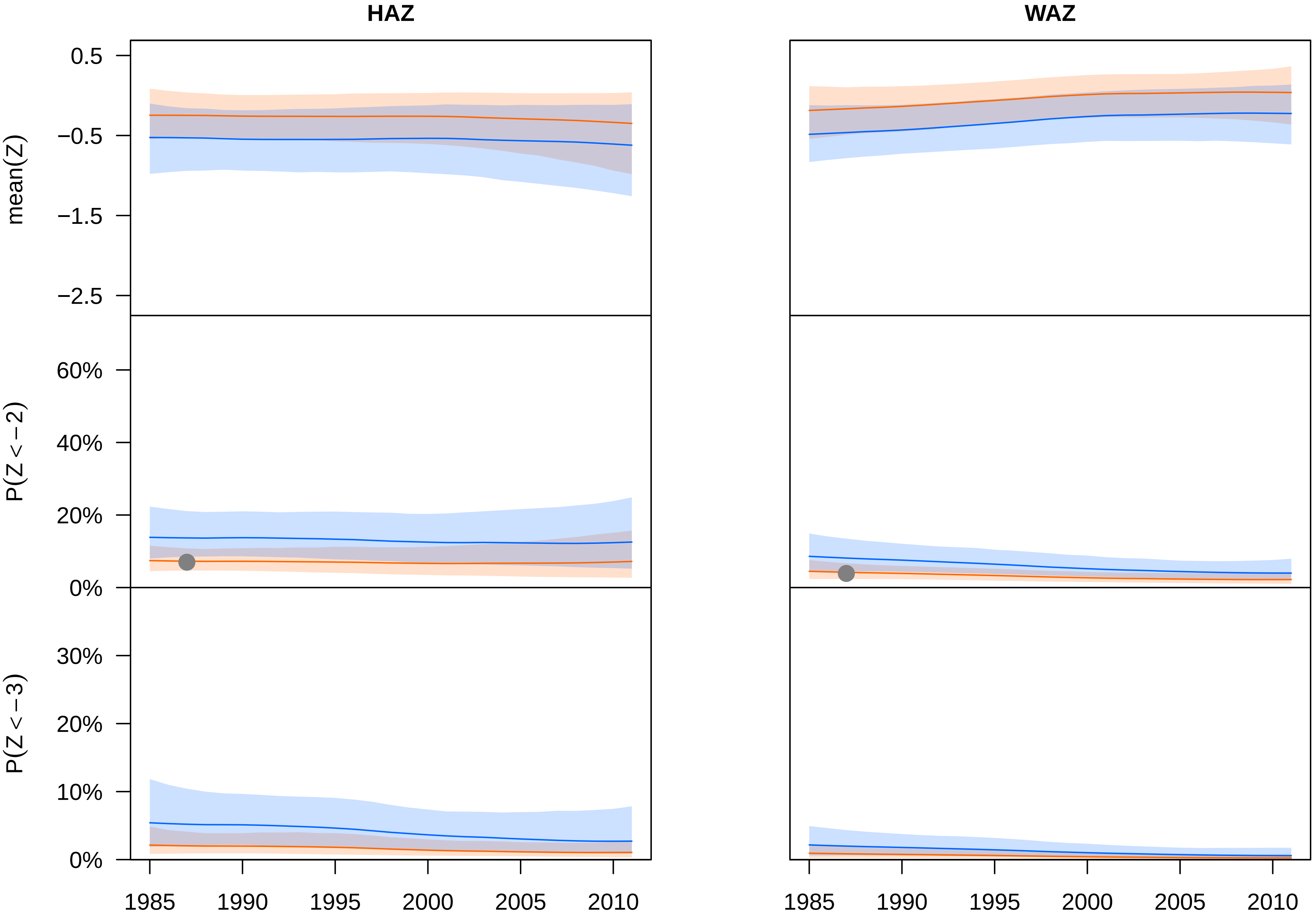

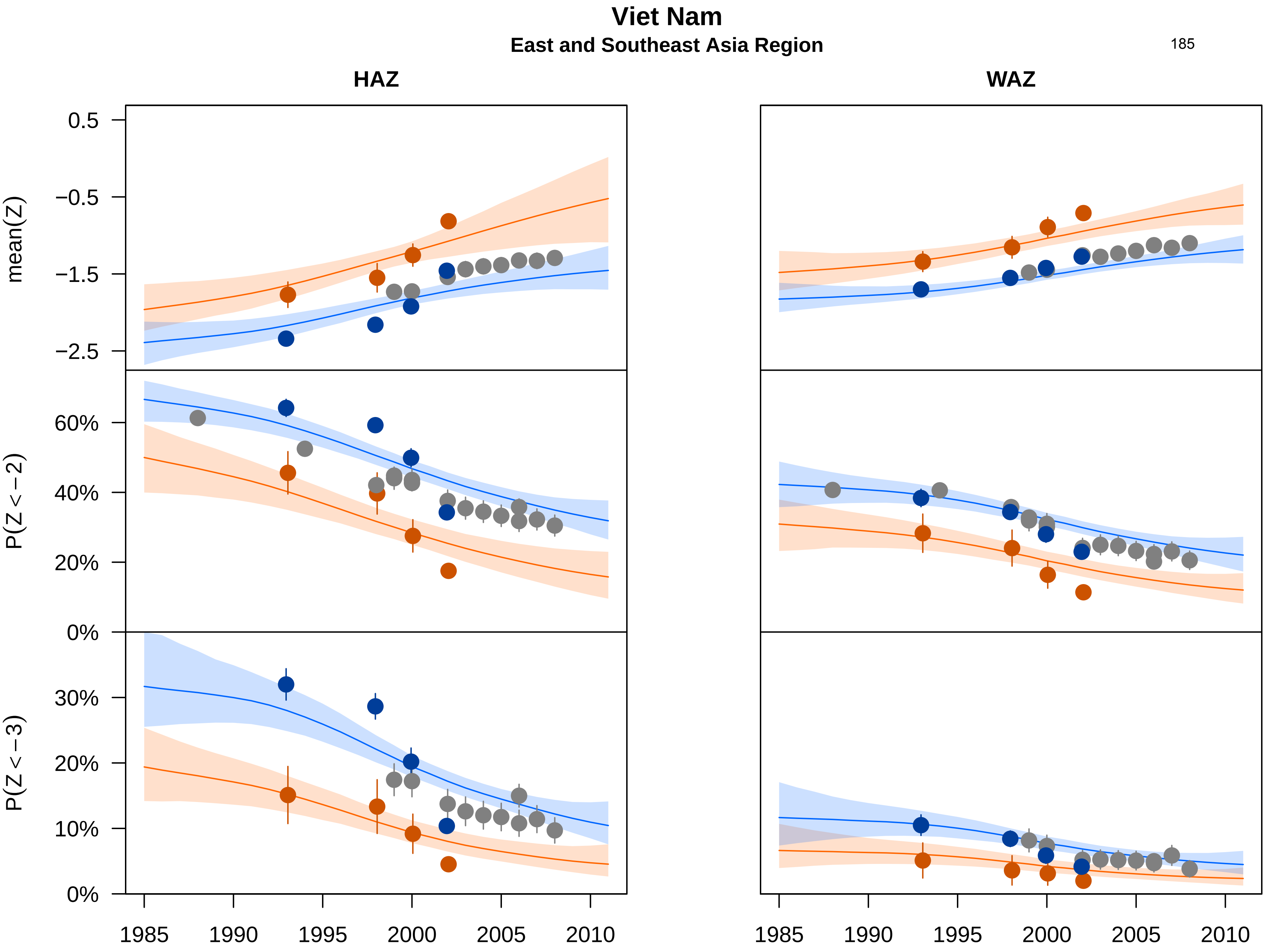
Viet Nam
East and Southeast Asia Region
HAZ
WAZ
mean(Z)
P(Z < −2)
P(Z < −3)
0.5
−0.5
−1.5
−2.5
60%
40%
20%
0%
30%
20%
10%
0%
1985
1990
1995
2000
2005
2010

Yemen

Central Asia, Middle East, and North Africa Region

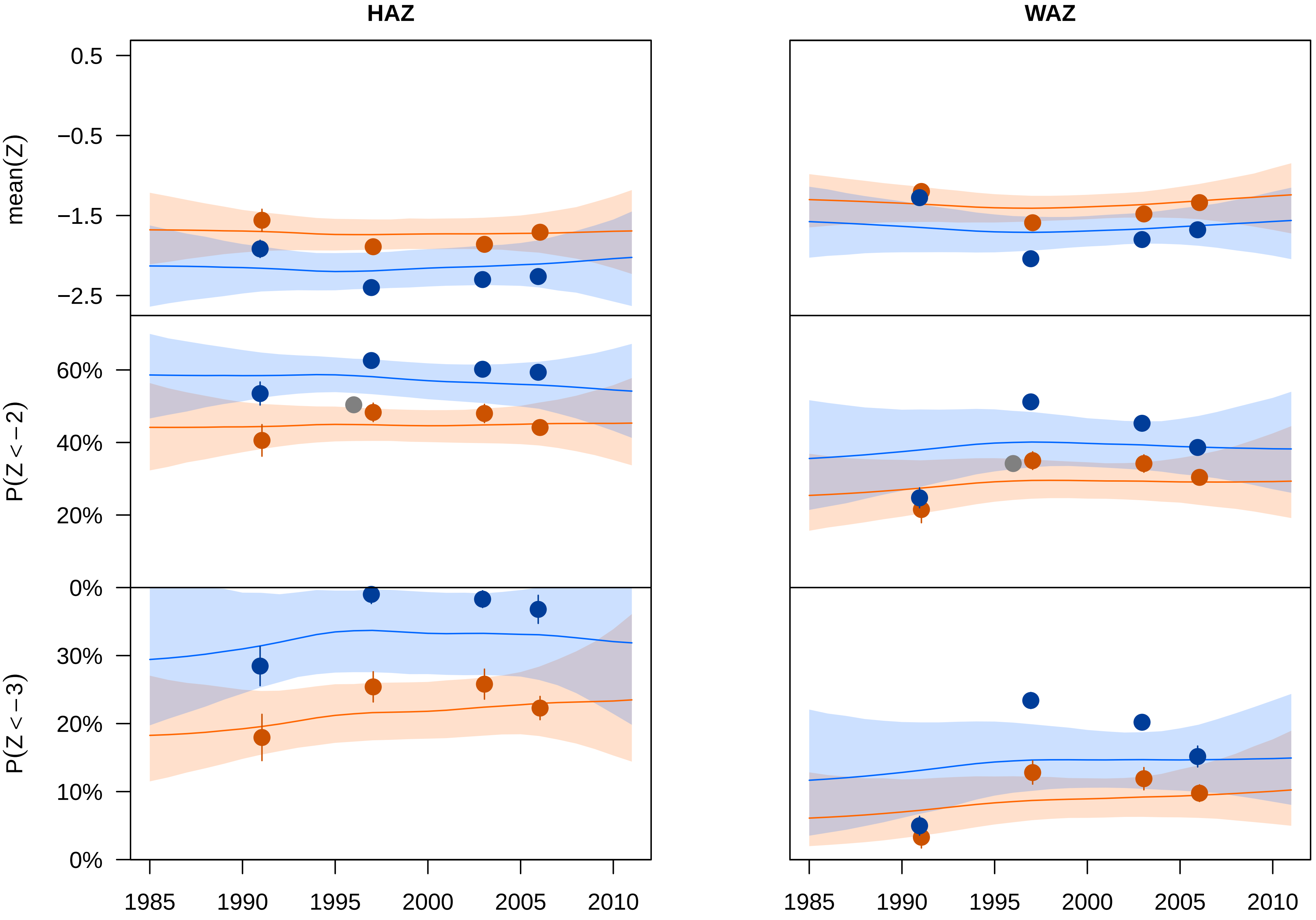

Zambia

Sub−Saharan Africa Region

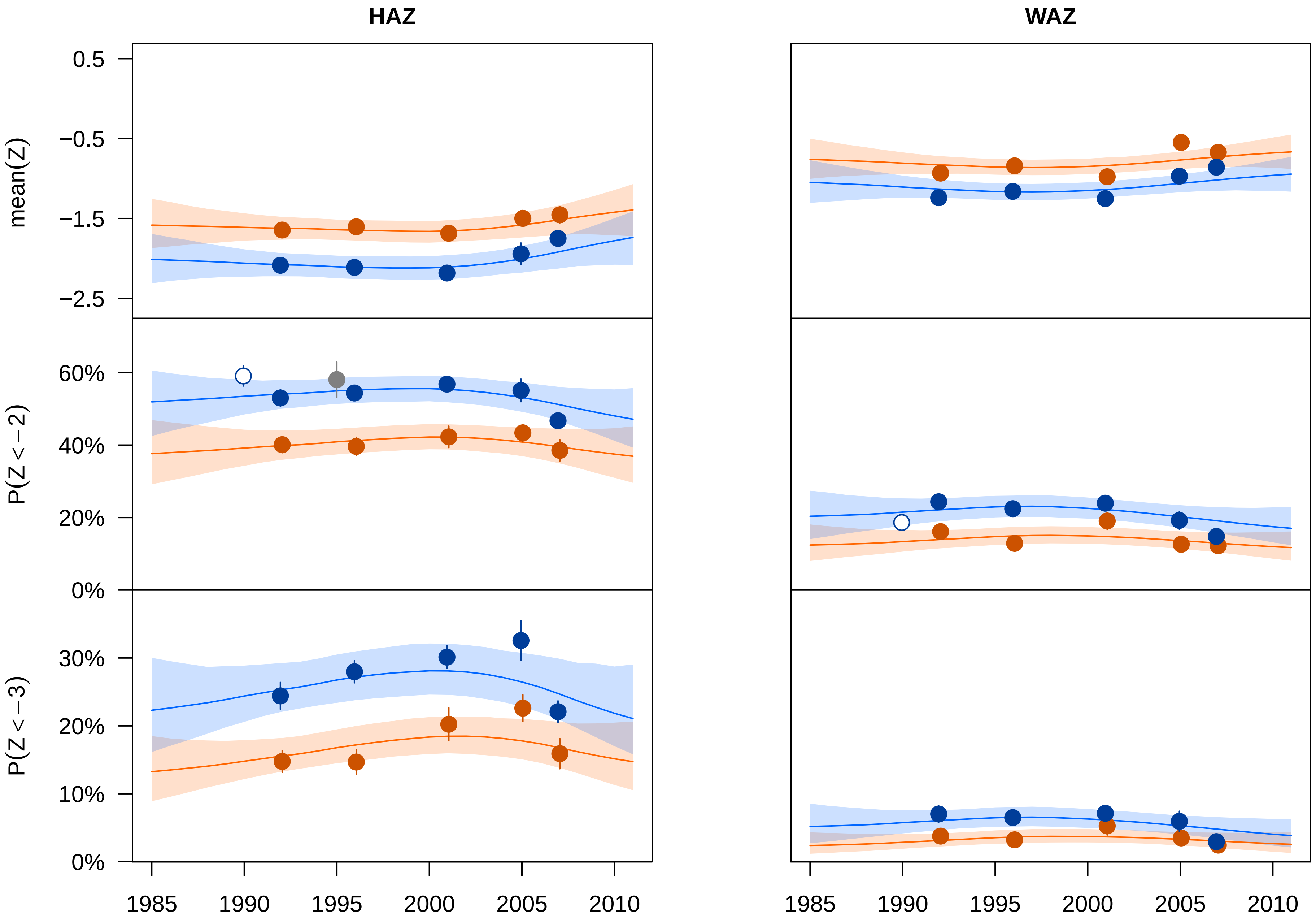

Zimbabwe

Sub−Saharan Africa Region

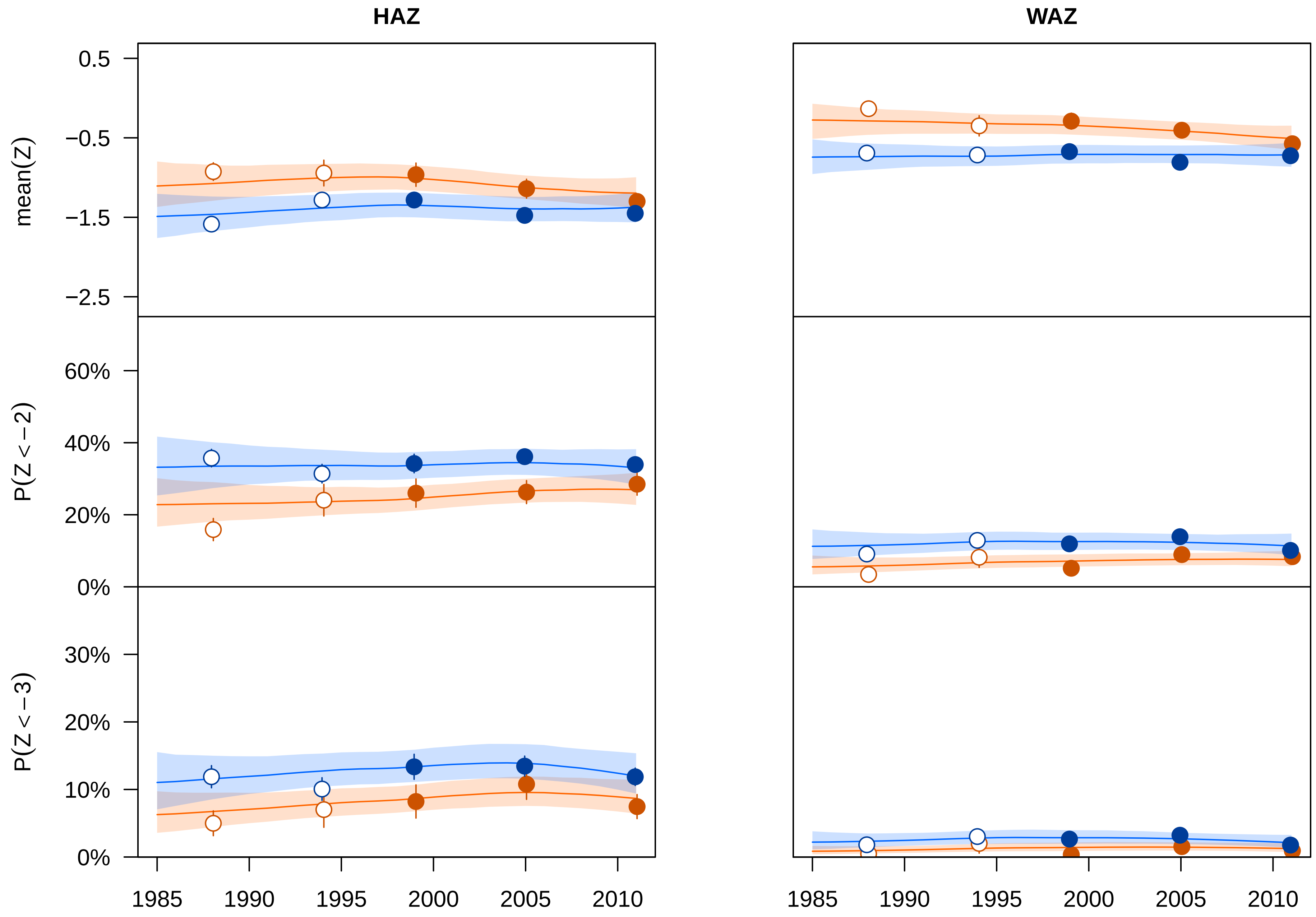

Supplement: Supplementary appendix [file mmc1.pdf]
